# Comprehensive RNA-Seq Profiling Reveals Temporal and Tissue-Specific Changes in Gene Expression in Sprague–Dawley Rats as Response to Heat Stress Challenges

**DOI:** 10.3389/fgene.2021.651979

**Published:** 2021-04-09

**Authors:** Jinhuan Dou, Angela Cánovas, Luiz F. Brito, Ying Yu, Flavio S. Schenkel, Yachun Wang

**Affiliations:** ^1^Key Laboratory of Animal Genetics, Breeding and Reproduction, MARA, National Engineering Laboratory for Animal Breeding, College of Animal Science and Technology, China Agricultural University, Beijing, China; ^2^Department of Animal Biosciences, Centre for Genetic Improvement of Livestock, University of Guelph, Guelph, ON, Canada; ^3^Department of Animal Sciences, Purdue University, West Lafayette, IN, United States

**Keywords:** heat stress response, RNA-sequencing, thermal tolerance, rats' transcriptome, mammals

## Abstract

Understanding heat stress physiology and identifying reliable biomarkers are paramount for developing effective management and mitigation strategies. However, little is known about the molecular mechanisms underlying thermal tolerance in animals. In an experimental model of Sprague–Dawley rats subjected to temperatures of 22 ± 1°C (control group; CT) and 42°C for 30 min (H30), 60 min (H60), and 120 min (H120), RNA-sequencing (RNA-Seq) assays were performed for blood (CT and H120), liver (CT, H30, H60, and H120), and adrenal glands (CT, H30, H60, and H120). A total of 53, 1,310, and 1,501 differentially expressed genes (DEGs) were significantly identified in the blood (*P* < 0.05 and |fold change (FC)| >2), liver (*P* < 0.01, false discovery rate (FDR)–adjusted *P* = 0.05 and |FC| >2) and adrenal glands (*P* < 0.01, FDR-adjusted *P* = 0.05 and |FC| >2), respectively. Of these, four DEGs, namely *Junb, P4ha1, Chordc1*, and *RT1-Bb*, were shared among the three tissues in CT vs. H120 comparison. Functional enrichment analyses of the DEGs identified in the blood (CT vs. H120) revealed 12 biological processes (BPs) and 25 metabolic pathways significantly enriched (FDR = 0.05). In the liver, 133 BPs and three metabolic pathways were significantly detected by comparing CT vs. H30, H60, and H120. Furthermore, 237 BPs were significantly (FDR = 0.05) enriched in the adrenal glands, and no shared metabolic pathways were detected among the different heat-stressed groups of rats. Five and four expression patterns (*P* < 0.05) were uncovered by 73 and 91 shared DEGs in the liver and adrenal glands, respectively, over the different comparisons. Among these, 69 and 73 genes, respectively, were proposed as candidates for regulating heat stress response in rats. Finally, together with genome-wide association study (GWAS) results in cattle and phenome-wide association studies (PheWAS) analysis in humans, five genes (*Slco1b2, Clu, Arntl, Fads1*, and *Npas2*) were considered as being associated with heat stress response across mammal species. The datasets and findings of this study will contribute to a better understanding of heat stress response in mammals and to the development of effective approaches to mitigate heat stress response in livestock through breeding.

## Introduction

Heat stress refers to the sum of the nonspecific physiological responses of the body to high ambient temperature (Dikmen and Hansen, 2009). Heat stress is a well-recognized environmental factor that threatens human health (Cheshire, 2016), animal welfare, and productive efficiency (Belhadj-Slimen et al., 2016; Polsky and von Keyserlingk, 2017). Significant increases in mortality and morbidity in humans and livestock exposed to severe heat stress have been documented in the literature (Mader et al., 2006; Belhadj-Slimen et al., 2016; Greene et al., 2016). Heat stress is also a major factor jeopardizing animal production in tropical, subtropical, and arid regions around the globe, through decreasing productive performance, compromised reproduction and welfare, and increasing heat-related diseases (Belhadj-Slimen et al., 2016; Saeed et al., 2019).

Heat stress poses great research challenges because it involves complex (and partially unknown) regulatory mechanisms in animals. The magnitude of the body's response to heat stress is influenced by numerous factors, such as the heat intensity (Thompson et al., 2018; Khan et al., 2020), heat duration (Volodina et al., 2017; Wang et al., 2019), genetic background of animals (e.g., species, breeds; Lees et al., 2018; Xu et al., 2018), and physiological state (Lucy and Safranski, 2017; Tamura et al., 2017). Therefore, it is difficult to evaluate and quantify the degree of heat stress. The temperature–humidity index (THI) is often used to represent the environmental conditions that drive heat stress (De Rensis et al., 2015). However, the definitions (Armstrong, 1994; De Rensis et al., 2015) and THI values (Bohmanova et al., 2007) tend to vary across studies and environmental conditions (e.g., geographic location, physical size, and the different equations), and therefore, THI can only be used as a rough indicator for assessing heat stress (Polsky and von Keyserlingk, 2017). In this context, various studies have focused on evaluating the effects of heat stress in humans and other animals (e.g., cattle, pig, and sheep) from the perspective of physiological measures to assess body coping mechanisms when exposed to different environmental challenges. For instance, rectal temperature (RT) (Leon et al., 2005) and respiratory rate (RR) (Kadzere et al., 2002) are common physiological indicators of heat stress response (Brito et al., 2020; Li et al., 2020). In addition, drooling score (DS) can also be as a good indicator for heat stress, because it is highly correlated with RR when heat stress occurs (Mader et al., 2006). However, although physiological measures can describe the health and biological status at a given environmental condition, they fail to constitute a gold standard due to their susceptibility to different detection methods and physiological conditions.

With the development of next-generation sequencing, including RNA-sequencing (RNA-Seq; Wickramasinghe et al., 2014), the accuracy and speed of identifying environmentally responsive genes at a large scale have increased substantially, thus contributing to a better understanding of the molecular mechanisms underlying heat stress response (Han et al., 2019; Li et al., 2019a). However, results remain limited by distinct heat stress conditions (intensity or time of heat stress), tissue sampling, and individual differences. Blood is a good model for medical and physiology research (Cohn, 2015) and is widely used in clinical disease diagnosis because of its advantages of noninvasive collection (Nisenblat et al., 2016; Kiddle et al., 2018). Previous studies have shown that mild/severe heat stress can affect blood physiological, biochemical, and immunological indicators (Bagath et al., 2019; Johnson et al., 2019), as well as the expression of certain genes [e.g., heat shock protein (HSP)] (Garner et al., 2020; Lee et al., 2020). Meanwhile, our previous studies have also proven that blood biomarkers can reflect the degree of acute and short-term heat stress (Dou et al., 2019, 2020). Liver is an important regulator of metabolism, controlling many of the physiological processes influenced by heat stress (Hubbard et al., 2019), and adrenal glands are the main tissues involved in production and release of heat stress–responsive hormones (e.g., glucocorticoids) to maintain animal homeostasis during heat stress (Li et al., 2019a,b). Moreover, heat stress induces transcriptomics, metabolic, and proteomic changes in the liver (Hubbard et al., 2019; Ma et al., 2019) and adrenal glands (Koko et al., 2004; Li et al., 2019a). In short, blood, liver, and adrenal glands are the three main tissues for studying the heat stress regulatory mechanisms. In this context, the main hypothesis of the present study is that the regulation of heat stress–related genes has temporal and tissue-specific differences. The main objectives of this study were to (1) identify heat stress–responsive genes in different key tissues (blood, liver, and adrenal glands) using the RNA-Seq technology and (2) investigate the dynamic expression pattern of differentially expressed genes (DEGs) in liver and adrenal glands under various heat stress conditions.

## Materials and Methods

### Ethics Approval

The animal protocol was reviewed and approved by the Institutional Animal Care and Use Committee from the China Agricultural University (Beijing, China). The *in vivo* rat experiments were performed at the College of Animal Science and Technology (China Agricultural University, Beijing, China). The Institutional Animal Care and Use Committee approved all the experimental procedures, which complied with the China Physiological Society's guiding principles for research involving animals and adhered to the high standard (best practice) of veterinary care as stipulated in the Guide for Care and Use of Laboratory Animals.

### Animals and Sample Collection

Twenty 8-week-old female Sprague–Dawley rats (Beijing Vital River Laboratory, Animal Technology Co. Ltd., Beijing, China), weighing 205 ± 7.16 g, were used as heat-stressed rat models under different climatic conditions: Control (22 ± 1°C and relative humidity of 50%, *n* = 5) or at three heat-stress conditions (42°C for 30 min (H30, *n* = 5), 60 min (H60, *n* = 5), or 120 min (H120, *n* = 5), following Dou et al. (2019). The changes in body temperature and biochemical indicators in blood (*n* = 4), liver (*n* = 5), and adrenal glands (*n* = 5) in the control group (CT) and H120 groups were investigated using RNA-Seq technology (Dou et al., 2020). In addition, liver (*n* = 5) and adrenal gland (*n* = 5) tissues from the H30 and H60 groups were also collected as described by Dou et al. (2020) and used for RNA-Seq analyses. Briefly, the experimental rats were anesthetized with an injection of 1.2 mL 1% pentobarbital sodium (0.6 mL/100 g body weight; Jeon et al., 2011), sacrificed, and quickly dissected by sterile surgical scissors and forceps (Shinva Medical Instrument Co. Ltd., Shandong, China). Liver and adrenal glands were collected, washed in ice-cold phosphate-buffered solution, and snap-frozen immediately in liquid nitrogen (−196°C).

### RNA Isolation and Library Construction

RNA reagents were used to extract the total RNA from the samples of Sprague–Dawley rats used in this study, according to the manufacturer's protocol (HUAYUEYANG Biotechnology Co., Ltd., Beijing, China). The purity of the RNA samples was assessed by A260/230 nm and A260/280 nm ratios using NanoDrop 2100 (Thermo Fisher Scientific, Waltham, MA, USA). The integrity of the RNA samples was determined using the RNA Nano 6000 Assay Kit of the Agilent Bioanalyzer 2100 system (Agilent Technologies, CA, USA), and the RNA concentration and genomic DNA contamination were determined using the Qubit^®^ 2.0 Fluorometer (Invitrogen, Carlsbad, CA, USA). The 260/280 ratio of all samples ranged from 1.8 to 2.0, and the integrity number (RIN) values were > 7.0, indicating good RNA quality (Cánovas et al., 2013). A total of 3 μg RNA from each sample was used to prepare the cDNA libraries using the NEBNext^®^ Ultra™ Directional RNA Library Prep Kit from Illumina^®^ (NEB, San Diego, CA, USA), following the manufacturer's recommendation. Paired-end (2 × 150 bp) reads of samples in H30 and H60 were sequenced using the HiSeq 2500 sequencer (Illumina, San Diego, CA, USA).

### RNA Reads Mapping and Candidate Gene Identification

A total of 48 RNA-Seq samples from 20 rats were analyzed. First, a quality control analysis was performed using the CLC Genomics Workbench software 20.0 (CLC Bio, Aarhus, Denmark, https://www.qiagenbioinformatics.com), following the parameters described by Cánovas et al. (2014a). All the samples analyzed passed all the quality control parameters having the same length (100 bp); 100% coverage in all bases; 25% of A, T, G, and C nucleotide contributions; 50% GC on base content; and < 0.1% overrepresented sequences, indicating good quality of sequencing outputs. The paired-end sequence reads were mapped to the reference genome *Rattus norvegicus* 6.0 (ftp://ftp.ensembl.org/pub/release-95/genbank/rattus_norvegicus/) using the CLC Genomics Workbench software 20.0 (Cánovas et al., 2014a). The detailed mapping parameters were as follows: two mismatches, three insertions, and three deletions per read were allowed; end-to-end with 0.9 length fraction (the minimum percentage of the total alignment length that must match the reference sequence at the selected similarity fraction); and 0.9 similarity fraction (the minimum percentage identity between the aligned region of the read and the reference sequence). Expression data were normalized by calculating the reads per kilobase per million mapped reads (RPKM = total exon reads/mapped reads in millions × exon length in kb) for each gene (Allison et al., 2006; Mortazavi et al., 2008; Cánovas et al., 2014b; Medici et al., 2016) and log_10_ transformed. Only transcripts with RPKM ≥ 0.2 were considered expressed genes; otherwise, they were discarded from further analyses (Cardoso et al., 2018). Principal component analysis (PCA) between RPKM expression levels for samples in different comparisons were performed using the R 3.6.3 software (https://cran.r-project.org/src/base/R-3/).

### Differential Gene Expression

Differential gene expression analyses were performed using the CLC Genomics Workbench software 20.0 as described by Asselstine et al. (2019) using a negative binomial generalized linear model (GLM). The GLM assumes that the expression levels follow a negative binomial distribution. This distribution has a free parameter, the dispersion. The greater the dispersion, the greater the variation in expression levels for a gene. The most likely values of the dispersion and coefficients are determined simultaneously by fitting the GLM to the data. Therefore, the Cox–Reid adjusted likelihood was used to correct the GLM dispersion for a gene (Robinson et al., 2010). Differential expression analysis was performed using *t*-test [*P* ≤ 0.01, false discovery rate (FDR)–adjusted *P* = 0.05 and |fold change (FC)| >2] on transformed data to identify the DEGs between the groups of samples under different heat-stressed conditions, with the exception of blood samples *P* < 0.05 and |FC| >2, considered to be DEGs (Medici et al., 2016). Short Time-series Expression Miner (STEM) was used for the dynamic expression pattern analyses of the shared DEGs identified among the four groups (Ernst and Bar-Joseph, 2006). The parameters of STEM analysis were set as follows: maximum number of model profiles equal to 20 and minimum FC of genes in different samples were >2, and the log_2_ normalization was used for the FC of gene expression between the heat treatment group and CT group (Ernst and Bar-Joseph, 2006). Significant patterns were considered at *P* < 0.05 and shown in color.

### Functional Enrichment Analysis and Gene Annotation

Functional annotation was performed using the Gene Ontology (GO) (http://geneontology.org) and Kyoto Encyclopedia of Genes and Genomes (KEGG) (http://www.genome.ad.jp/kegg/) databases based on the GO biological processes (BPs) and pathway analysis. The analyses were performed based on DEGs identified in each tissue under various heat stress conditions, and those DEGs significantly enriched in dynamic expression patterns. The BPs and metabolic pathways significantly associated with the gene lists were determined based on their FDR (Ashburner et al., 2000). Functional evidence of the relationship between the significant GO terms (FDR = 0.05) and the DEGs was identified (Sweett et al., 2020). The FDR thresholds of 0.05 for genes involved in the BPs or metabolic pathways were applied to filter significant candidate genes by function, pathway, and network effects (Cardoso et al., 2017). The Protein Analysis Through Evolutionary Relationships (PANTHER; Mi et al., 2019) and Database for Annotation, Visualization, and Integrated Discovery (DAVID) 6.8 (https://david.ncifcrf.gov/tools.jsp) were used to perform functional annotation for the genes in the liver and adrenal glands that were significantly (*P* < 0.05) enriched in the expression patterns (Cánovas et al., 2012).

### Cross-Species Analysis of Genes With Significant Expression Patterns in the Liver and Adrenal Glands

In another of our recent studies, a multiple-trait animal model was used to estimate genetic parameters using 59,265 RT, 30,290 RR, and 30,421 DS records from 13,592 lactating Holstein cows (Luo et al., 2020). A total of 2,627 cows were genotyped using the Illumina 150K Bovine Bead chip (Illumina, San Diego, CA, USA). After quality control of the chip data, a total of 116,594 single-nucleotide polymorphisms (SNPs) were used in the analysis (Luo et al., 2020). Subsequently, the LiftOver software (https://genome.ucsc.edu/cgi-bin/hgLiftOver) was used to convert the 140 unique DEGs of rats to the cattle genes based on the physical locations and UMD 3.1 bovine reference genome (Bos_taurus_UMD 3.1.1/bos Tau8) information. After the LiftOver analysis, the intersect function of BEDTools software (Quinlan and Hall, 2010) was used to perform overlap analysis between the genes converted to the cow with SNPs. Moreover, only the SNP with the smallest *P-*value that overlapped with each gene was retained and stored into a new candidate list. Finally, a phenome-wide association studies (PheWAS) analysis was performed based on the new candidate gene list using the GWASATLAS software (Watanabe et al., 2020), which provides annotation information on human genome for candidate SNPs and genes related to complex traits. The human complex traits were mainly summarized based on six key groups, namely, cardiovascular, endocrine, environmental, immunological, metabolic, and others (e.g., neurological, psychiatric, and respiratory).

## Results and Discussion

Through the exploration of the transcriptome profile, further understanding of the coping mechanisms under heat stress challenges from the perspective of genetic regulation is essential for in-depth explaining of thermo-tolerance and breeding of heat-tolerant varieties. In the present study, a well-established heat-stressed rat model was used to investigate the effects of various heat stress conditions (H30, H60, and H120) on the gene expression levels in multiple tissues (blood, liver, and adrenal glands), thereby providing a theoretical reference for the research on the temporal and tissue-specific expression of heat stress genes.

### Sequencing and Profiling of Transcriptomes in Response to Heat Stress

For the liver and adrenal gland tissues in the H30 and H60 groups, the 20 RNA-Seq libraries generated an average of 10.94 Gb paired-end clean reads, for which the percentages of Q20 and Q30 were > 96 and 92%, respectively, with GC content of < 50% (Supplementary Table 1). The quality of the 28 RNA-Seq data generated from blood, liver, and adrenal gland samples in the CT and H120 groups was reported by Dou et al. (2020) and showed an average of 9.46 Gb paired-end clean reads for each sample, with high quality (GC content: 50.81%, Q20: 95.06%, and Q30: 89.10%). In the current study, an average of 87.47% reads for the 48 samples was mapped in pairs (Supplementary Table 2). Furthermore, 92.46% and 7.54% of the reads were mapped in genic and intergenic regions, respectively (Supplementary Table 2). The PCA analysis indicated that the samples seem to be more similar within treatment group and more divergent across treatments based on each principal component dimension (Supplementary Figure 1). In addition, under different heat stress conditions, the difference between the CT and heat treatment groups of the adrenal glands was clear (Supplementary Figure 1), which may be because adrenal glands can rapidly release the glucocorticoids into the bloodstream when heat stress occurs (Blake et al., 1991), and then the glucocorticoids act on a variety of tissues to yield their numerous homeostatic effects (Meaney et al., 1993).

### Differentially Gene Expression Analysis

In the blood (CT vs. H120), liver (CT vs. H30, CT vs. H60, and CT vs. H120), and adrenal gland (CT vs. H30, CT vs. H60, and CT vs. H120) tissues, a total of 53, 2,449, and 2,763 genes were identified based on *P* < 0.05 and |FC| >2, as well as 0, 1,310, and 1,501 genes were detected based on *P* < 0.05, FDR-adjusted *P* = 0.05, and |FC| >2, respectively (Table 1). The detailed information on the DEGs in blood, liver, and adrenal gland tissues is summarized in Supplementary Tables 3–9. Among the 53 DEGs in blood, 66.04% of genes were up-regulated in CT vs. H120 (Table 1 and Supplementary Table 3), and the *Actg1* (actin, gamma 1) gene was the most expressed with FC = 39.70. The *Actg1* has a function associated with response to mechanical stimulus and positive regulation of gene expression (Ogneva et al., 2014). The possible role of the *Actg1* gene in heat stress has not been reported yet. The number of DEGs in the liver and adrenal glands was 24.26 and 27.80 times higher, respectively, than that in blood. In the liver (Supplementary Tables 4–6), most DEGs were found when comparing CT vs. H60, which were 225 and 289 more than those for CT vs. H30 and CT vs. H120, respectively. However, most DEGs in the adrenal glands (Supplementary Tables 7–9) were identified at H120 (723), and the numbers of up-regulated DEGs were 126, 250, and 277 higher than down-regulated DEGs in CT vs. H30, CT vs. H60, and CT vs. H120, respectively. In all comparisons in the liver, *Hspa1b* [HSP family A (HSP70) member 1B] gene had the largest FC among all the DEGs identified in CT vs. H30 (FC = 101.69) and CT vs. H60 (FC = 1,281.27), and also the top 2 genes were expressed with FC = 315.32 in CT vs. H120. Furthermore, the top 1 expressed gene in CT vs. H120 was *LOC108348108* (heat shock 70-kDa protein 1A; FC = 461.79), which belongs to the HSP70 family together with *Hspa1b*. HSPs are ubiquitously expressed in eukaryotes (Locke and Tanguay, 1996) and play a critical role in the regulation of many fundamental cellular processes as molecular chaperones (Abravaya et al., 1992), such as heat stress response (Baler et al., 1996). Previous studies have confirmed that the expression of endogenous *Hspa1b* was modified by *Hsf2* (heat shock factor 2) (Le Masson and Christians, 2011). However, the specific mechanisms of the interaction between *Hsf2* and *Hsp70* during heat stress in rats remain unclear. Unlike the liver tissue, the *Hspa1b* in adrenal glands changed most in H60 (FC = 182.12). The *LOC108348130* [carbonyl reductase (NADPH) 1–like] and *Vom2r53* (vomeronasal 2 receptor, 53) genes were most expressed under H30 and H120 treatments, respectively. *LOC108348130* and *Vom2r53* play an important role in the oxidation–reduction process and G protein–coupled receptor signaling pathway, respectively, and no evidence has been reported of their roles in heat stress response yet.

**Table 1:** Summary of differentially expressed genes (DEGs) in each comparison.

**Tissue (criteria)**	**Comparisons**	**Total**	**Up-regulated**	**Down-regulated**
Blood (*P* < 0.05, |FC| >2)	CT vs. H120	53	35	18
Liver (*P* < 0.05, |FC| >2)	CT vs. H30	714	619	95
	CT vs. H60	1,081	971	110
	CT vs. H120	654	377	277
Adrenal glands (*P* < 0.05, |FC| >2)	CT vs. H30	543	376	167
	CT vs. H60	1,026	628	398
	CT vs. H120	1,194	670	524
Blood (*P* < 0.01, FDR-adjusted *P* = 0.05, |FC| >2)	CT vs. H120	0	0	0
Liver (*P* < 0.01, FDR-adjusted *P* = 0.05, |FC| >2)	CT vs. H30	383	115	268
	CT vs. H60	608	267	341
	CT vs. H120	319	178	141
Adrenal glands (*P* < 0.01, FDR-adjusted *P* = 0.05, |FC| >2)	CT vs. H30	226	176	50
	CT vs. H60	552	401	151
	CT vs. H120	723	500	223

*FDR, false discovery rate, adjusted P-value; FC, fold change*.

**Figure 1:**
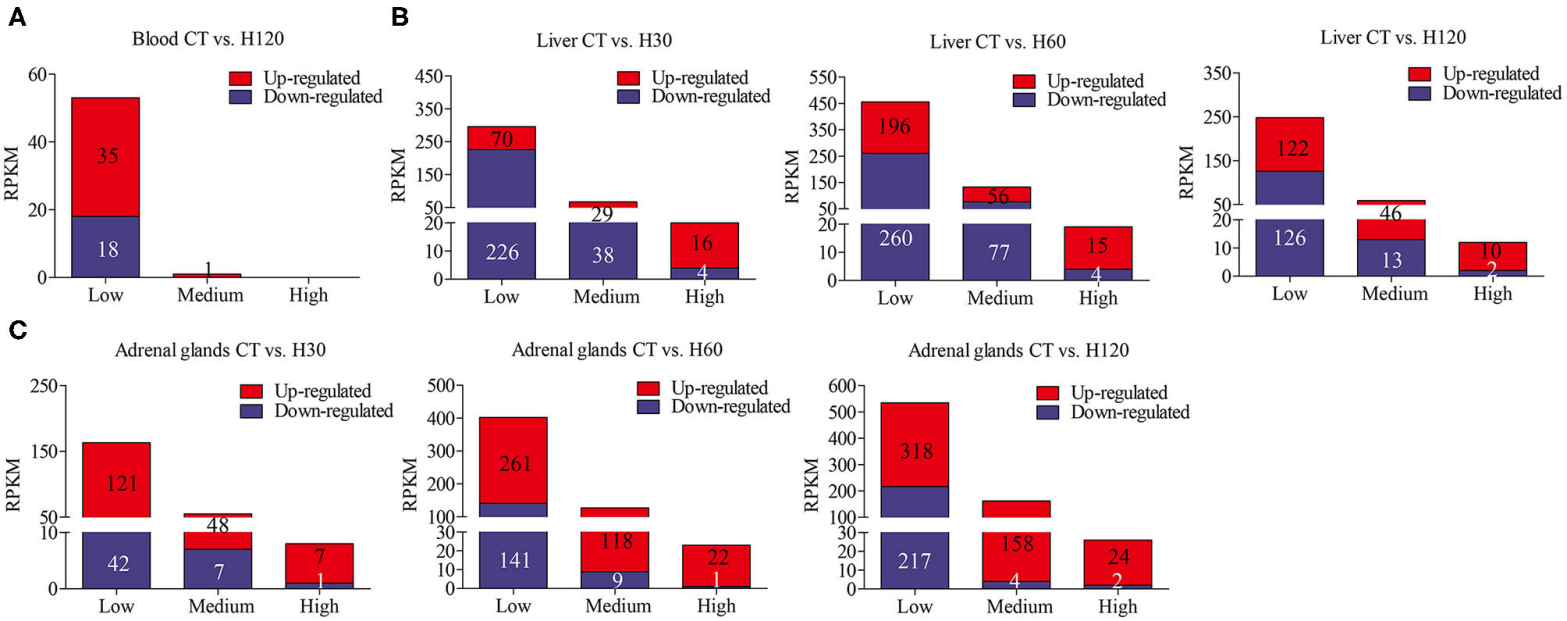
Number of lowly (RPKM ≤ 50), medium (50 < RPKM < 500), and highly (RPKM ≥ 500) expressed genes in each library. **(A–C)** refer to the numbers of lowly, medium and highly expressed genes identified in blood, liver and adrenal glands, respectively.

To further understand the expression levels of DEGs in blood, liver, and adrenal glands, the numbers of genes with low, medium, and high expression levels were summarized according to the criteria of RPKM ≤ 50, 50 < RPKM < 500, and RPKM ≥500 (as described by Cánovas et al., 2014a). Most DEGs belong to the low expression group (RPKM ≤ 50), followed by medium and highly DEG levels (Figure 1). Genes with the highest RPKM values in the liver were cytochrome c oxidase I, mitochondrial (*Mt-co1*, CT vs. H30) and cytochrome P450, family 2, subfamily c, and polypeptide 7 (*Cyp2c7*, CT vs. H60, and CT vs. H120), respectively. *Mt-col* is a mitochondrial component, which contributes to cytochrome c oxidase activity and is involved in the process of response to oxidative stress (García and Chávez, 2007; Gaudet et al., 2011). *Cyp2c7*, a gene in the cytochrome P450 family, encodes for cytochrome P450 2C7 precursor and participates in the epoxygenase P450 pathway (Gaudet et al., 2011). *Hspa1b* (CT vs. H30 and CT vs. H60) and scavenger receptor class B, member 1 (*Scarb1*, CT vs. H120), were highly expressed in adrenal glands. The function of *Hspa1b* was discussed above, and the gene *Scarb1* has the function of positive regulation of cholesterol storage and lipid transport (Rigotti et al., 1997; Azhar et al., 2002).

### Functional Annotation of the DEGs

**Figure 2:**
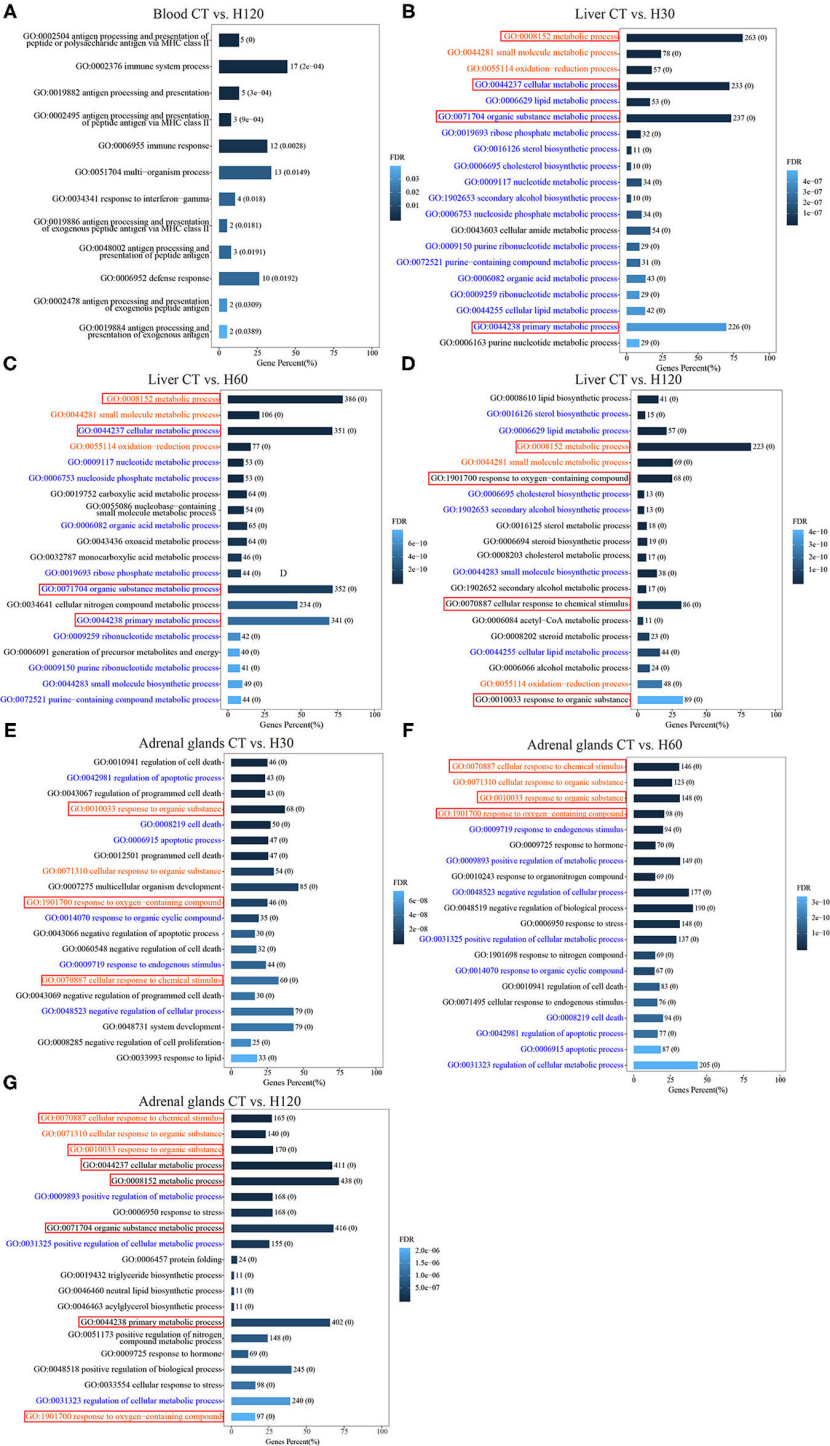
Significantly (FDR = 0.05) enriched biological processes (BPs) for differentially expressed genes (DEGs) in the blood, liver, and adrenal glands. CT means rats were kept at 22 ± 1°C and relative humidity 50%; H30, H60, and H120 mean rats were kept at 42°C for 30, 60, and 120 min, and the relative humidity 50% conditions. **(A)** All of the 12 significantly enriched BPs for DEGs in blood for CT vs. H120. **(B–D)** The top 20 significantly enriched BPs for DEGs in liver for CT vs. H30, CT vs. H60, and CT vs. H120, respectively. **(E–G)** The top 20 significantly enriched BPs for DEGs in adrenal glands for CT vs. H30, CT vs. H60, and CT vs. H120, respectively. The red words mean these BPs are significantly enriched in three comparisons of the same tissue; the blue words mean these BPs are significantly enriched in only two comparisons of the same tissue; the black words mean these BPs are specifically enriched in each comparison of the tissue. The red boxes mean these BPs are shared among different tissues.

To elucidate the biological functions related to heat stress at the molecular level, genes that were differentially expressed in blood, liver, and adrenal glands were included in a functional enrichment analysis performed using the GO and KEGG databases. In blood, 38 unique DEGs were significantly enriched (FDR = 0.05) in 12 unique BPs (Figure 2A and Supplementary Table 10). Among them, 5 of 38 unique genes were assigned to the most significant BPs (FDR = 7.99E^−07^) of antigen processing and presentation of peptide or polysaccharide antigen via major histocompatibility complex (MHC) class II (GO: 0002504). In addition, 17 genes were significantly enriched in BPs of the immune system process (GO: 0002376, FDR = 1.98E^−04^) (Supplementary Table 10). In the liver, 323, 492, and 271 unique genes from comparisons of CT vs. H30, CT vs. H60, and CT vs. H120, respectively, were assigned to GO analysis, and 133 unique BPs were significantly enriched across all comparisons, which included response to regulation of cold-induced thermogenesis (GO: 0120161), adaptive thermogenesis (GO:1990845), and temperature homeostasis (GO: 0001659) (Supplementary Table 10). In addition, 233 BPs were commonly found in at least two comparisons, and 29, 134, and 158 BPs were specifically enriched in comparisons of CT vs. H30, CT vs. H60, and CT vs. H120, respectively (Supplementary Table 10). The top 20 significantly enriched BPs in each comparison of liver tissues are shown in Figures 2B–D. In adrenal glands, 226, 552, and 723 DEGs were identified in comparisons of CT vs. H30, CT vs. H60, and CT vs. H120, respectively, and 183, 469, and 612 of these were commonly enriched (FDR = 0.05) in 237 BPs (Supplementary Table 10). The commonly identified BP terms included response to stress (GO:0006950), regulation of response to stress (GO:0080134), cellular response to stress (GO:0033554), regulation of cellular response to stress (GO:0080135), response to temperature stimulus (GO:0009266), and cellular response to heat (GO:0034605). Three hundred fifty-four BPs were identified in at least two comparisons. Furthermore, 178, 313, and 133 BPs were specifically enriched in CT vs. H30, CT vs. H60, and CT vs. H120, respectively (Supplementary Table 10). In addition, Figures 2E–G show the top 20 significantly enriched BPs terms for CT vs. H30, CT vs. H60, and CT vs. H120 (Figure 2E). Of these, the cellular response to chemical stimulus (GO:0070887) was the most significantly enriched term in the comparisons of CT vs. H60, and CT vs. H120, with FDR of 1.86E^−17^ and 3.63E^−13^, respectively, following cellular response to organic substance (GO:0071310) and response to organic substance (GO:0010033). In addition, Figure 2 also shows that seven BPs terms were commonly enriched in liver and adrenal glands, and no shared BP terms were detected among blood, liver, and adrenal glands.

For the KEGG pathway overrepresentation analysis, 25 significantly enriched pathways were detected (FDR = 0.05) in blood (Table 2), all of which are interconnected and directly engaged in innate inflammatory host defense, suggesting that multiple innate immunity and inflammation-related pathways in the blood at H120 can be completely mobilized to respond to acute and mild heat stress response (Bagath et al., 2019). Functional enrichment analysis of the 383, 605, and 319 DEGs in the liver of CT vs. H30, CT vs. H60, and CT vs. H120 comparisons found 15, 24, and 20 significant pathways, respectively (Table 3). Three significantly enriched pathways, namely, metabolism, fatty acid metabolism, and peroxisome proliferator-activated receptor (PPAR) signaling pathways, were shared among all the comparisons. Previous research has suggested that heat stress can cause abnormal liver function and lead to metabolic changes (i.e., carbohydrate metabolism, protein breakdown, and metabolism and nonesterified fatty acid levels in the plasma; Bagath et al., 2019; Li et al., 2019b). Heat stress can promote the synthesis and storage of fatty acids and reduce fat decomposition by increasing insulin concentrations (Ding et al., 2002). Fourteen significantly enriched pathways were detected in at least two comparisons of the liver tissue results, of which the oxidative phosphorylation and thermogenesis pathways (FDR = 1.74E^−05^ in CT vs. H30 and FDR = 2.35E^−06^ in CT vs. H60) were the most significantly enriched. During heat stress response process, hypothalamus plays a vital role in adaptive increases in body temperature by inducing thermogenesis (Dimicco and Zaretsky, 2007). In our study, 21 and 28 genes in the liver related to thermogenesis were enriched in the CT vs. H30 and CT vs. H60 comparisons, respectively, indicating that rats mobilize these genes to adapt to the heat stress environment. Among these, eight genes were shared in two comparisons in the liver, including *Mt-nd4l* (NADH dehdrogenase 4L, mitochondrial), *LOC679739* [NADH dehydrogenase (ubiquinone) Fe-S protein 6], *Atp5mc1* (ATP synthase membrane subunit c locus 1), *Mt-nd2* (NADH dehydrogenase 2, mitochondrial), *Acsl5* (acyl-CoA synthetase long-chain family member 5), *LOC100361457* (actin, gamma 1 propeptide-like), *Mt-nd5* (NADH dehydrogenase 5, mitochondrial), and *LOC1009125*. The specific mechanisms of these gene regulations of heat stress response need to be further investigated.

**Table 2:** Significantly enriched metabolic pathways (FDR = 0.05) using the list of DEGs identified in blood between CT vs. H120.

**Metabolic pathway**	Number^*^	**FDR**
Viral myocarditis	7	6.82E^−08^
Graft-vs.-host disease	6	1.52E^−07^
Allograft rejection	6	1.52E^−07^
Type I diabetes mellitus	6	1.99E^−07^
Autoimmune thyroid disease	6	1.99E^−07^
Asthma	4	1.27E^−05^
Antigen processing and presentation	5	2.66E^−05^
Influenza A	6	4.52E^−05^
Phagosome	6	5.20E^−05^
Intestinal immune network for immunoglobulin A production	4	5.20E^−05^
*Staphylococcus aureus* infection	4	8.71E^−05^
Herpes simplex infection	6	9.27E^−05^
Inflammatory bowel disease (IBD)	4	1.53E^−04^
Leishmaniasis	4	2.08E^−04^
Cell adhesion molecules	5	3.56E^−04^
Rheumatoid arthritis	4	4.93E^−04^
T_H_1 and T_H_2 cell differentiation	4	5.01E^−04^
Hematopoietic cell lineage	4	5.01E^−04^
T_H_17 cell differentiation	4	9.08E^−04^
Toxoplasmosis	4	9.98E^−04^
Systemic lupus erythematosus	4	1.21E^−03^
Osteoclast differentiation	4	1.54E^−03^
HTLV-I infection	5	3.22E^−03^
Tuberculosis	4	4.24E^−03^
Natural killer cell–mediated cytotoxicity	3	1.56E^−02^

* *Number of genes enriched in the pathway. DEGs, differentially expressed genes; FDR, false discovery rate. CT means rats were kept at 22 ± 1°C and relative humidity of 50%; H120 means rats were housed at 42°C for 120 min and relative humidity of 50%. Red words represent the pathway was commonly enriched by genes identified in blood, liver, and adrenal gland tissues; pink words represent the pathway was commonly enriched by genes identified in blood and liver; black words represent the pathway was specifically enriched in blood*.

**Table 3:** Significantly enriched metabolic pathway (FDR = 0.05) using the list of DEGs identified in liver tissue under different heat-stressed conditions.

**Metabolic pathway**	Number^*^	**FDR**
**CT vs. H30**		
Metabolic pathways	72	3.21E^−10^
Oxidative phosphorylation	16	1.74E^−05^
Thermogenesis	21	1.74E^−05^
Fatty acid metabolism	10	2.14E^−05^
PPAR signaling pathway	12	2.14E^−05^
Parkinson disease	15	1.13E^−04^
Ribosome	14	1.33E^−03^
Biosynthesis of unsaturated fatty acids	6	1.53E^−03^
Fatty acid degradation	7	2.41E^−03^
Glycerolipid metabolism	8	2.80E^−03^
Terpenoid backbone biosynthesis	5	2.86E^−03^
Carbon metabolism	11	3.28E^−03^
Tryptophan metabolism	6	1.62E^−02^
Valine, leucine, and isoleucine degradation	6	3.20E^−02^
Glycine, serine, and threonine metabolism	5	4.64E^−02^
**Pathway**	Number^*^	**FDR**
**CT vs. H60**		
Metabolic pathways	100	1.32E^−11^
Non-alcoholic fatty liver disease	24	2.86E^−07^
Oxidative phosphorylation	21	2.35E^−06^
Thermogenesis	28	2.35E^−06^
Parkinson disease	21	5.72E^−06^
PPAR signaling pathway	14	5.31E^−05^
Huntington disease	21	3.31E^−04^
Retinol metabolism	11	7.75E^−04^
Ribosome	18	9.05E^−04^
Pyruvate metabolism	8	1.52E^−03^
Alzheimer disease	18	2.62E^−03^
Chemical carcinogenesis	10	8.62E^−03^
Retrograde endocannabinoid signaling	15	1.41E^−02^
Tryptophan metabolism	7	3.01E^−02^
Insulin resistance	11	3.27E^−02^
Steroid hormone biosynthesis	8	3.32E^−02^
Glycolysis/gluconeogenesis	8	3.85E^−02^
Fatty acid metabolism	7	4.70E^−02^
Fluid shear stress and atherosclerosis	13	4.70E^−02^
Tight junction	14	4.70E^−02^
Glycine, serine, and threonine metabolism	6	4.70E^−02^
Adipocytokine signaling pathway	8	5.00E^−02^
Apoptosis	12	5.00E^−02^
Drug metabolism—cytochrome P450	7	5.00E^−02^
**CT vs. H120**		
Metabolic pathways	59	9.86E^−08^
Terpenoid backbone biosynthesis	7	1.60E^−05^
PPAR signaling pathway	11	4.64E^−05^
Fatty acid metabolism	9	5.86E^−05^
Steroid hormone biosynthesis	9	1.88E^−04^
Pyruvate metabolism	7	3.56E^−04^
Steroid biosynthesis	5	1.15E^−03^
T_H_17 cell differentiation	10	1.64E^−03^
Antigen processing and presentation	8	4.83E^−03^
Biosynthesis of unsaturated fatty acids	5	5.43E^−03^
Glycerolipid metabolism	7	5.43E^−03^
Glycolysis/gluconeogenesis	7	6.10E^−03^
Protein processing in endoplasmic reticulum	11	7.81E^−03^
Bile secretion	7	1.00E^−02^
Carbon metabolism	9	1.26E^−02^
AMPK signaling pathway	9	1.41E^−02^
FoxO signaling pathway	9	1.49E^−02^
Fatty acid degradation	5	2.76E^−02^
Circadian rhythm	4	3.60E^−02^
Adipocytokine signaling pathway	6	3.60E^−02^

* *Number of genes enriched in the pathway. DEGs, differentially expressed genes; FDR, false discovery rate. CT means rats were kept at 22 ± 1°C and relative humidity of 50%; H30, H60, and H120 mean rats were housed at 42°C for 30, 60, and 120 min and relative humidity of 50%, respectively. The pathway colored with blue means these pathways were commonly shared among CT vs. H30, CT vs. H60, and CT vs. H120 comparisons. Pathway colored with light orange means these pathways were shared by two comparisons. Noncolored pathway means these pathways were treatment-specific enriched. Red words indicate that the pathway was commonly enriched by genes identified in blood, liver, and adrenal gland tissues; pink words indicate the pathway was commonly enriched by genes identified in blood and liver; green words indicate the pathway was commonly enriched by genes identified in liver and adrenal gland tissues; black words indicate the pathway was specifically enriched in liver*.

Compared to the CT group, 8 and 11 pathways were significantly (FDR = 0.05) enriched for DEGs identified in adrenal glands after H30 and H60 exposure, respectively, and no significant pathway was enriched by DEGs identified at H120 (Table 4). Among these, three significantly enriched pathways were shared in CT vs. H30 and CT vs. H60, namely, circadian rhythm, amphetamine addiction, and protein digestion and absorption. Interaction between circadian rhythm and the stress system is essential in a myriad of physiological processes to maintain homeostasis, which plays important roles in health by regulating blood pressure, sleep/wake cycles, and related cellular signaling pathways (Wilking et al., 2013). However, the disruption of intact communication by exogenous factors (i.e., heat stress) may increase the risk of developing metabolic, immune, or neuroendocrine disorders (Rakesh et al., 2014). In mammals, the circadian system is composed of a main pacemaker located in the hypothalamic suprachiasmatic nucleus (SCN) and subordinated clocks throughout the brain and periphery (Ralph et al., 1990). Among the most popular studied mediators of circadian entrainment, glucocorticoids play a critical role in stress response, and their secretion is controlled by the SCN and tissue clocks along the hypothalamus–pituitary–adrenal axis (Oster et al., 2006). As it is well-known, many physiological and metabolic rhythms are synchronized by the adrenal glands (Oster et al., 2006), including the glucocorticoids rhythms (corticosterone in rodent and cortisol in other mammals such as human and cattle) and clock gene rhythms, such as *Per1* (period circadian regulator 1), which is involved in the regulation of mitogen-activated protein kinase (MAPK) and cyclic adenosine monophosphate (cAMP) during the heat stress processes (Jerônimo et al., 2017). Therefore, the study of how the molecular circadian rhythms regulate the transcription of genes in adrenal glands will help clarify the influence of heat stress on metabolism and immunity (Pantazopoulos et al., 2018).

**Table 4:** Significantly enriched pathway (FDR = 0.05) of DEGs identified in adrenal glands.

**Pathway**	Number^*^	**FDR**
**CT vs. H30**		
Circadian rhythm	5	7.69E^−03^
Amphetamine addiction	6	2.26E^−02^
Dopaminergic synapse	8	2.26E^−02^
Cocaine addiction	5	2.26E^−02^
Ribosome	8	4.56E^−02^
Tyrosine metabolism	4	4.56E^−02^
Protein digestion and absorption	6	4.56E^−02^
Steroid biosynthesis	3	4.98E^−02^
**CT vs. H60**		
Circadian rhythm	7	7.25E^−03^
Transcriptional misregulation in cancers	17	7.54E^−03^
TNF signaling pathway	12	1.41E^−02^
Prostate cancer	11	1.41E^−02^
FoxO signaling pathway	12	2.83E^−02^
Antigen processing and presentation	9	2.83E^−02^
Protein digestion and absorption	10	2.83E^−02^
Relax in signaling pathway	12	3.02E^−02^
Amphetamine addiction	8	3.06E^−02^
Ferroptosis	6	3.96E^−02^
Protein processing in endoplasmic reticulum	13	3.96E^−02^

* *Number of genes enriched in the pathway. DEGs, differentially expressed genes; FDR, false discovery rate. CT means rats were kept at 22 ± 1°C and relative humidity of 50%; H30, H60, and H120 mean rats were housed at 42°C for 30, 60, and 120 min and relative humidity of 50%, respectively. Pathway colored with light orange means these pathways were shared by two comparisons. Noncolored pathway means these pathways were treatment-specific enriched. Red words indicate the pathway was commonly enriched by genes identified in blood, liver, and adrenal gland tissues; green words indicate the pathway was commonly enriched by genes identified in liver and adrenal gland tissues; black words indicate the pathway was specifically enriched in adrenal glands*.

### Shared and Specific Genes Among Various Treatments and Tissues

**Figure 3:**
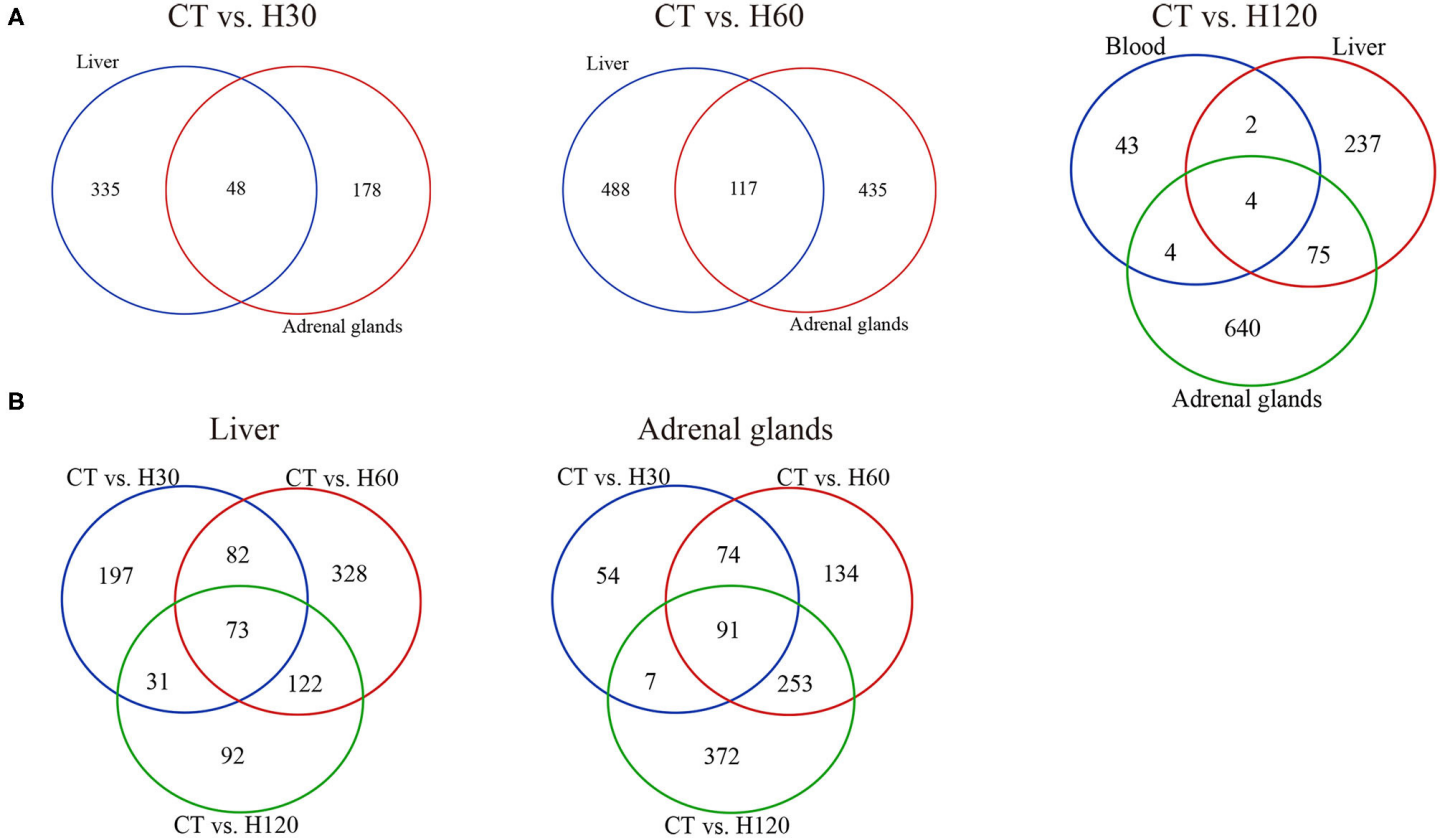
Analysis of shared and specific genes across treatments and tissues. **(A)** Venn diagram of DEGs in blood, liver, and adrenal gland tissues under the same heat stress conditions. CT means rats were kept at 22 ± 1°C and relative humidity 50%; H30, H60, and H120 mean rats were kept at 42°C for 30, 60, and 120 min, and the relative humidity 50% conditions. **(B)** Venn diagram of DEGs in comparisons of CT vs. H30, CT vs. H60, and CT vs. H120 in the liver and adrenal gland tissues, respectively.

Venn diagrams of DEGs identified under the same heat treatment time or in the same tissue were created (Figure 3). A total of 48 DEGs were shared by liver and adrenal gland tissues when H30 was compared to CT, and 21 and 23 of them were up-regulated in the liver and adrenal gland tissues, respectively (Figure 3A, left panel). For CT vs. H60, 117 genes were differentially expressed in both liver and adrenal glands, with ~78 (67.52%) and 86 (73.50%) DEGs, respectively, up-regulated (Figure 3A, central panel). Compared to CT vs. H30 and CT vs. H60, only four genes were commonly identified in the blood, liver, and adrenal gland tissues in CT vs. H120, including *Junb* (JunB proto-oncogene, AP-1 transcription factor subunit), *P4ha1*(prolyl 4-hydroxylase subunit α1), *Chordc1* (cysteine and histidine-rich domain containing 1) and *RT1-Bb* (RT1 class II, locus Bb) with FC values ranging from -3.67 to 12.19 (blood), 2.14 to 9.66 (liver), and 2.64 to 9.88 (adrenal glands). Furthermore, the *P4ha1* gene had the highest FC value. A heat map of the four shared genes shows that these genes in the liver and adrenal glands were clustered more closely in H120 (Supplementary Figure 2A). The endoplasmic reticulum (ER) can be triggered by heat stress via accumulation of unfolded or misfolded proteins (Alemu et al., 2018). It has been shown that *P4ha1* is up-regulated during ER stress (Vonk et al., 2010). In the current study, several genes involved in the pathways of antigen processing and presentation (blood, liver, and adrenal glands), protein processing in the ER (liver and adrenal glands), and protein digestion and absorption (adrenal glands) were identified, indicating the ER stress occurs under heat stress. The up-regulation of *P4ha1* was associated with the heat-induced ER stress and could serve as an indicator for heat stress. Figure 3B reveals that 73 and 91 genes were shared under different heat treatments in the liver and adrenal gland tissues, respectively, and had more similar expression mode after heat exposure than the control group (Supplementary Figures 2B,C).

### Temperature-Dependent Gene Expression Patterns Identified by RNA-Seq in the Liver and Adrenal Glands Under Different Heat Stress Times

**Figure 4:**
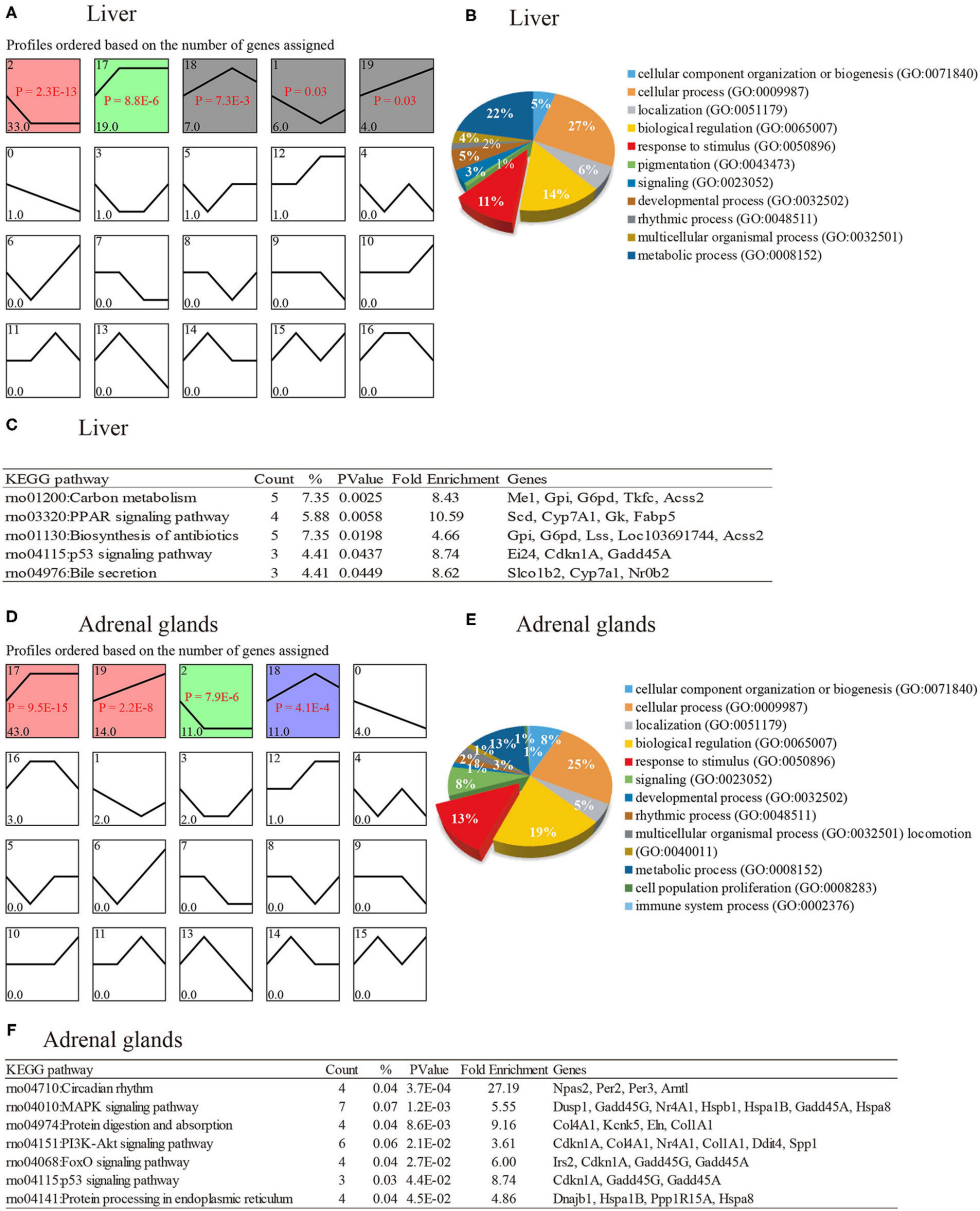
Temperature-dependent gene expression patterns and their functions. CT means rats were kept at 22 ± 1°C and relative humidity 50%; H30, H60, and H120 mean rats were kept at 42°C for 30, 60, and 120 min, and the relative humidity 50% conditions. **(A)** The expression pattern of 91 genes of liver identified in comparisons of CT vs. H30, CT vs. H60, and CT vs. H120. **(B)** Pie plot of biological processes (BPs) enriched by genes of the liver, which were significantly (*P* < 0.05) enriched in patterns. **(C)** Significantly enriched pathways for genes in liver that were significantly (*P* < 0.05) enriched in patterns. **(D)** The expression pattern of 73 genes of the adrenal glands identified in comparisons of CT vs. H30, CT vs. H60, and CT vs. H120. **(E)** Pie plot of biological processes (BPs) enriched by genes of adrenal glands, which were significantly (*P* < 0.05) enriched in patterns. **(F)** Significantly enriched pathways for genes in the adrenal glands that were significantly (*P* < 0.05) enriched in patterns.

Based on *P* < 0.05, five patterns were significantly enriched by 69 DEGs of the liver (Figure 4A). The most significantly (*P* = 2.3E^−13^) enriched pattern in the liver was profile 2, indicating that compared to the CT group, 33 DEGs were down-regulated after heat stress. Furthermore, the second significant pattern showed 19 DEGs were up-regulated after heat exposure. Profile 19 reveals that the expression levels of four genes, namely, *Tmem140* (transmembrane protein 140), *Slc28a2* (solute carrier family 28), *Igfbp1* (insulin-like growth factor binding protein 1), and *Irf2bp2* (interferon regulatory factor 2 binding protein 2), increased with longer heat stress challenge. Subsequently, GO analysis was performed on these 69 DEGs using PANTHER (Figure 4B), and ~11% of the genes were involved in the BPs of response to stimulus (GO:0050896), of which 4, 7, and 10 genes were engaged in three subgroups of response to stimulus, namely, response to stress (GO:0006950), response to chemical (GO:0042221), and cellular response to stimulus (GO:0051716), respectively. Pathway analysis indicated that these 69 genes were significantly (*P* < 0.05) enriched in five pathways (Figure 4C).

A total of 19 possible expression patterns were detected among the 91 DEGs in the adrenal glands (Figure 4D). Of these, four patterns (profiles 17, 19, 2, and 18), containing 79 DEGs, were significantly enriched (*P* < 0.05). Among the 79 genes, eight genes, namely, *Gpd1* (glycerol-3-phosphate dehydrogenase 1), *Dusp1* (dual specificity phosphatase 1), *Arntl* (aryl hydrocarbon receptor nuclear translocator-like), *Hspa1b, Cdkn1a* (cyclin-dependent kinase inhibitor 1A), *Pim3* (Pim-3 proto-oncogene, serine/threonine kinase), *Zfp36l1* (zinc finger protein 36, C3H type-like 1), and *Gadd45a* (growth arrest and DNA damage–inducible α), were also identified in the liver and were enriched in significant patterns (Figure 4A). Forty-three DEGs in profile 17 were up-regulated after heat stress (*P* < 0.05). In contrast, 11 DEGs in profile 2 were down-regulated in heat treatment groups compared with the CT group (*P* < 0.05). The second significant pattern (profile 19), represented by 14 DEGs, shows that the expression of these genes increased with prolonged duration of heat stress. The fourth significant pattern, which included 11 DEGs, indicated that these genes were highly expressed in H60 compared to H30 and H120 conditions. The functional annotation of the 79 genes revealed that 25% of genes were involved in cellular processes (GO: 0009987), followed by 13% of genes involved in the process of response to stimulus (GO:0050896). Furthermore, a total of seven sublevels of response to stimulus were enriched by 19 genes (such as *Hspa8* [HSP family A (Hsp70) member 8], *Igtp* (interferon gamma–induced GTPase), *Gadd45g* (growth arrest and DNA damage–inducible, gamma), *Ddit4* (DNA damage–inducible transcript 4), *Emd* (emerin), *Nr4a1* (nuclear receptor subfamily 4, group A, member 1), *Ppp1r15a* (protein phosphatase 1, regulatory subunit 15A), *Sik1* (salt-inducible kinase 1), *Irs2* (insulin receptor substrate 2), *Tnfaip6* (TNFα-induced protein 6), *Ndrg1* (N-myc downstream regulated 1), *Per2* (period circadian regulator 2), *Bok* (BCL2 family apoptosis regulator BOK), *Hspa1b, Amotl2* (angiomotin-like 2), *Gadd45a, Ackr3* (atypical chemokine receptor 3), *Dusp1* and *Per3* (period circadian regulator 2), and the seven sublevel processes included response to external stimulus (GO: 0009605), response to stress (GO:0006950), response to abiotic stimulus (GO:0009628), response to chemical (GO:0042221), response to endogenous stimulus (GO:0009719), immune response (GO:0006955) and cellular response to stimulus (GO:0051716) (Figure 4E). Seven pathways were significantly (*P* < 0.05) enriched by these 79 genes (Figure 4F), including the most significant pathway circadian rhythm, followed by MAPK signaling pathway and protein digestion and absorption. In addition, validation experiments are needed to further verify whether or not these pathways play a vital role in heat stress response.

### Cross-Species Analysis of Candidate Genes

Previous studies have shown that many heat stress responsive genes are orthologous across different mammalian species and have similar functions. For instance, *HSP70* termed as *Hspa4* in rat, *HSPA1A* in cattle, and *HSPA1* in human, is involved in modulating a variety of physiological processes, including proliferation, apoptosis, and stress responses (Hao et al., 2018). Therefore, in order to further study the importance of potential candidate genes identified in the rat model, a cross-species analysis was performed, including cattle and human, based on gene homology. After combing the 69 and 73 genes in the liver and adrenal glands, respectively, a total of 140 unique genes were retained and converted to 108 cattle genes. Overlapping analysis between cattle genes and SNPs associated with RT, RR, and DS traits, and then retaining the SNP with the smallest *P-*values for each gene generated 53 unique genes related to the traits. Finally, four [*SLCO1B3* (solute carrier organic anion transporter family member 1B3), *FADS1* (fatty acid desaturase 1), *KCNK5* (potassium two pore domain channel subfamily K member 5), *COL1A1* (collagen type Iα 1 chain)], five [*CLU* (clusterin), *FADS1, SPP1* (secreted phosphoprotein 1), *PDLIM1* (PDZ and LIM domain 1), *NPAS2* (neuronal PAS domain protein 2)], and five [*ARNTL* (aryl hydrocarbon receptor nuclear translocator-like), *CSRNP3* (cysteine and serine-rich nuclear protein 3), *NPAS2, NR4A1* (nuclear receptor subfamily 4 group A member 1), *MREG* (melanoregulin)] genes with significant SNPs for RT, RR, and DS were obtained, respectively. The detailed information related to these gene expression levels in rat tissues and their corresponding cattle genes and SNPs are shown in Supplementary Table 11. In dairy cattle, RT, RR, and DS are usually regarded as valuable physiological indicators for heat stress assessment (Mader et al., 2006; Kovacs et al., 2018).

**Figure 5:**
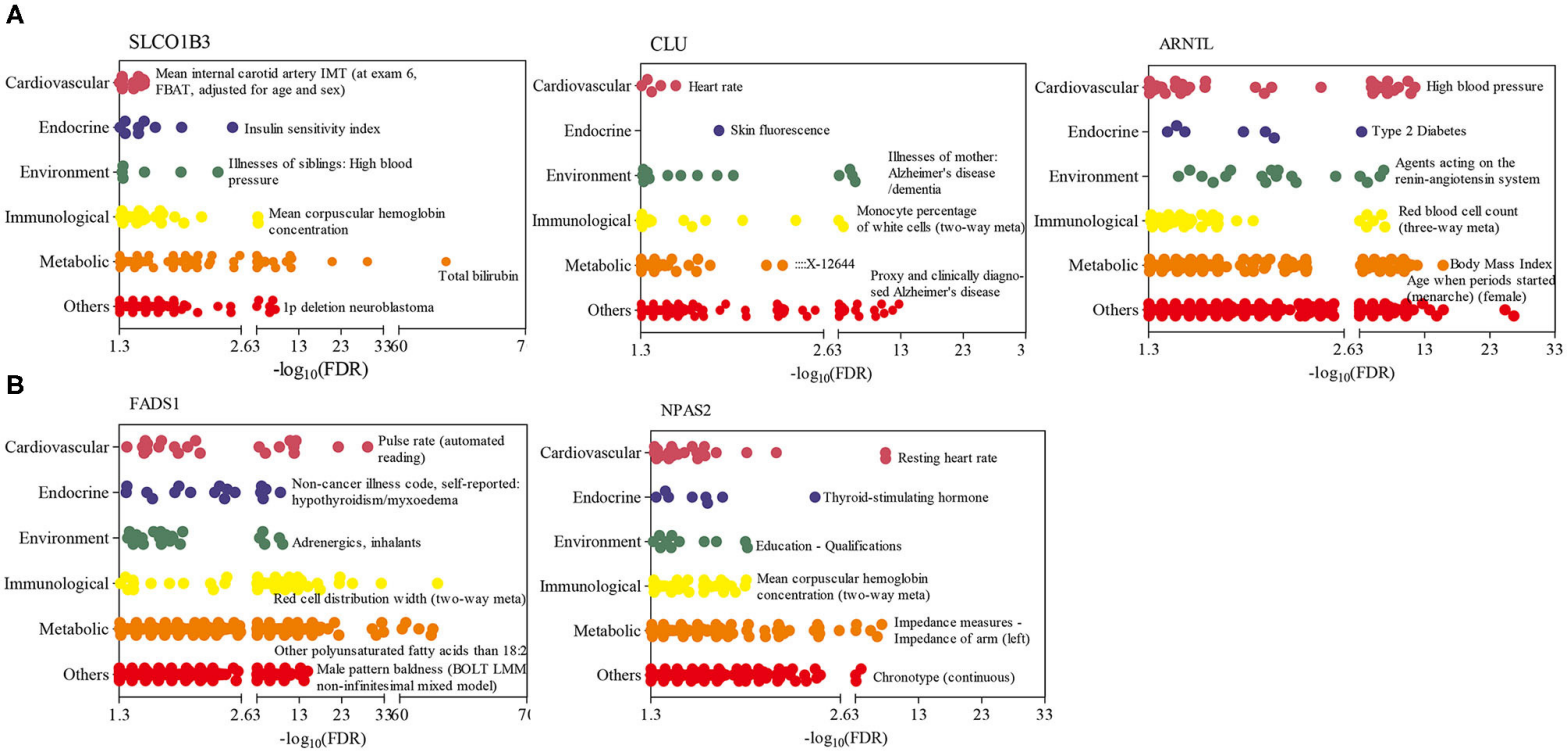
Phenome-wide association studies (PheWAS) results for five genes in humans. **(A)** PheWAS results of three genes, which had the most significant SNPs for rectal temperature (RT), respiration rate score (RR), and drooling score (DS) traits. **(B)** PheWAS results of two genes with SNPs identified for at least two SNP traits. The human complex traits were summarized mainly from six aspects, including cardiovascular, endocrinal, environmental, immunological, metabolic, and others (e.g., neurological, psychiatric, activities, respiratory). Only the name of the most significant trait in each aspect has been reported.

To further explore the function of candidate genes in humans, a PheWAS analysis was performed for five genes, namely, *SLCO1B3, CLU*, and *ARNTL*, which had the most significant SNPs for the RT, RR, and DS traits, and *FADS1* and *NPAS2*, which had significant SNPs identified for at least two traits. Figure 5 indicates that these genes were correlated with many human complex traits. From the endocrine category, we found that *SLCO1B3, ARNTL, FADS1*, and *NPAS2* were the most significant genes related to hormone metabolism (e.g., insulin and thyroid-stimulating hormone; Figure 5). From the perspective of the environment, *SLCO1B3* and *CLU* genes are strongly related to diseases, such as high blood pressure (Alencastro de Azevedo et al., 2012) and Alzheimer disease (Wilson and Zoubeidi, 2017), and *ARNT1* and *FADS1* are closely related to response to estradiol (Mitsushima et al., 2003) and hepatic metabolites (Zheng et al., 2020).

The *SLCO1B3* gene encodes a liver-specific member of the organic anion transporter family, which encodes a protein that transports a variety of endogenous and exogenous compounds, including hormones and their conjugates, in addition to anticancer drugs (Sun et al., 2020). Accumulated data indicate that the abnormal expression and function of *SLCO1B3* are related to resistance to anticancer drugs, and defective *SLCO1B3* causes hyperbilirubinemia, rotor type (HBLRR), SLC transporter disorders, and metabolism of lipids and steroids disorders (Alencastro de Azevedo et al., 2012; Woo et al., 2017). Therefore, more investigations have been carried out to identify the potential SLCO1B3-related mechanisms of cancer drug resistance. However, the function of *SLCO1B3* (*Slco1b2* in rats) has not been reported in heat stress studies. Its regulatory mechanism in cancer, critical role in bile acid, bilirubin, and hormone transport may be helpful when exploring its potential mechanism related to heat stress response.

The *CLU* encoding protein is a secreted chaperone that can be found in the cell cytosol under stress conditions. *CLU* is involved in basic BPs, such as cell death, neurodegenerative disorder, and tumor progression (Bertacchini et al., 2019; Seo et al., 2019). Previous studies also demonstrated that the CLU protein can interact with HSP90AA1 and enhance the effect of HSP90AA1 on the activation of the Eph receptor B1 (ERK), AKT serine/threonine kinase 1 (AKT), and nuclear factor kappa B (NF-κB) families, in addition to epithelial-to-mesenchymal transition and migration in breast cancer cells (Tian et al., 2019). The CLU protein is mainly produced by rat Sertoli cells and protects surviving cells during heat stress by binding to toxic compounds or dissolving cellular debris released by the degenerating cells (Clark and Griswold, 1997). Furthermore, another heat stress experiment (43°C) performed on the scrotums of rats found that the apoptotic index in the treatment with siRNA targeting the CLU (>70% reduction in the expression of CLU mRNA) was markedly higher than that of the control group, suggesting that an increase in the secretion of CLU by Sertoli cells protects the testis from heat stress–induced injury (Matsushita et al., 2016). In the current study, *CLU* was up-regulated with FC ranging from 2.27 to 2.93 in adrenal glands after H30, H60, and H120 exposure, indicating that *CLU* may protect adrenal glands from heat stress–induced injury by inhibiting cell apoptosis. However, the detailed regulatory mechanism of *CLU* in animals remains largely unknown.

The *ARNTL* gene, also named as *BMAL1*, in which the encoding protein can form a heterodimer with circadian locomotor output cycles kaput (CLOCK) and, then, the BMAL1: CLOCK heterodimer, activates the transcription of genes, such as *Period* (*PER1, PER2*, and *PER3*) and *Cryptochrome* (*CRY1, CRY2*) by binding E-box enhancer elements (Tamaru and Takamatsu, 2018). The CLOCK system has the function of invoking daily time-dependent adaptive responses via interaction with various stress protection systems (Tamaru and Ikeda, 2016). The circadian CLOCK interacts with BMAL1, and heat shock factor-1 (HSF1) during heat stress and oxidative stress was previously reported (Tamaru et al., 2011). In addition, the CLOCK system also controls the expression of stress resistance genes (i.e., *PER2* and *BMAL1*) (Zhang et al., 2014) and activates various adaptive protection pathways that control antioxidant and cell survival responses to protect cells from stressors (Kawamura et al., 2018). In summary, the *ARNTL* gene was identified in heat-stressed rats and cattle and also functions as an important biomarker for several diseases in humans (Engin, 2017; Maiese, 2017). Therefore, *ARNTL* is a good candidate gene for future heat stress studies.

The *FADS1* gene–coding protein belongs to the FADS gene family. Variants in *FADS1* have been associated with cardiometabolic risk factor (O'Neill and Minihane, 2017), plasma high sensitivity C-reactive protein (Roke et al., 2013), and oxylipin synthesis (Hester et al., 2014). *NPAS2* is a circadian clock gene and is involved in the circadian clock pathway and metabolism of lipids (Yuan et al., 2020). A study has shown that the BMAL1/NPAS2 complex significantly induces the transcription of *Fabp3* under cold stress (Chappuis et al., 2013). Up to now, no heat stress study has been conducted focusing on the *FADS1* and *NPAS2* genes; thus, this study provided new insights into the functions of *FADS1* and *NPAS2* in the heat stress response and the circadian adaptive system.

Five genes, including *Slco1b2, Clu, Arntl, Fads1*, and *Npas2*, were proposed as potential novel candidates for heat stress response in mammals. However, it is necessary to validate them by studying the expression changes in rats and other species at alternate temperatures and more time points. In addition, in a previous study in our research group, RNA-Seq analysis was performed on bovine granulosa cells at 38°C (control), 39, 40, and 41°C conditions, which revealed many DEGs associated with heat stress (Khan et al., 2020). Therefore, the RNA-Seq results from the rat study and bovine granulosa cells will be further integrated and analyzed to identify more important bovine heat stress response biomarkers.

## Conclusions

Transcriptome profiles of blood, liver, and adrenal tissues of female Sprague–Dawley rats were generated, and valuable transcriptional genetic markers under heat stress conditions were identified. Four genes (*Junb, P4hal, Chordcl*, and *RT1-Bb*) differentially expressed in the blood, liver, and adrenal gland tissues under the H120 treatment, and 69 (liver) and 73 (adrenal glands) genes identified under various heat treatment conditions are suggested as key candidate genes for future studies. In particular, five genes (*Slco1b2, Clu, Arntl, Fads1*, and *Npas2*) were considered as novel candidates for heat stress response. This study provides valuable datasets and important findings from a rat model as background for future studies in both livestock and humans and for the mitigation of adverse consequences of rising global temperatures.

## Data Availability Statement

The RNA-Sequencing datasets generated during the current study were available in the Sequence Read Archive (SRA) at the National Center for Biotechnology Information (NCBI) with BioProject accession number PRJNA690189.

## Ethics Statement

The animal study was reviewed and approved by Institutional Animal Care and Use Committee from the China Agricultural University (Beijing, China). The *in vivo* rat experiments were performed at the College of Animal Science and Technology (China Agricultural University, Beijing, China). The Institutional Animal Care and Use Committee approved all the experimental procedures, which complied with the China Physiological Society's guiding principles for research involving animals and adhered to the high standard (best practice) of veterinary care as stipulated in the Guide for Care and Use of Laboratory Animals.

## Author Contributions

YW and JD participated in the design of the study. JD collected samples, performed all the experiments, analyzed the data, and drafted the manuscript. YW and YY guided the experimental implementation. AC provided training on the bioinformatics and functional analysis to the first author, contributed to the analyses, and results interpretation. LB and FS gave editorial assistance. LB, FS, YW, YY, and AC edited the final version of the manuscript and provided valuable suggestions. All authors reviewed and approved the final draft.

## Conflict of Interest

The authors declare that the research was conducted in the absence of any commercial or financial relationships that could be construed as a potential conflict of interest.

## Supplementary Material

Supplementary Figure 1 The principal component analysis (PCA) of samples in each comparison of tissue. CT means rats were kept at 22 ± 1°C and relative humidity 50%; H30, H60, and H120 mean rats were kept at 42°C for 30, 60, and 120 min, and the relative humidity 50% conditions.

Supplementary Figure 2 Expression patterns of shared genes identified in varies conditions. **(A)** Heat map of four shared DEGs of blood, liver, and adrenal gland tissues in CT vs. H120. **(B)** Heat map of 73 shared DEGs of liver identified in comparisons of CT vs. H30, CT vs. H60, and CT vs. H120. **(C)** Heat map of 91 shared DEGs of adrenal glands identified in comparisons of CT vs. H30, CT vs. H60, and CT vs. H120.

Supplementary Table 1 Quality of sequencing reads of liver and adrenal glands in the H30 and H60 groups.

Supplementary Table 2 Descriptive statistics of reads generated by 48 RNA-Seq libraries alignment to reference genome.

Supplementary Table 3 The statistics of differentially expressed genes (DEGs) with *P* < 0.05 and |fold change| >2 in blood in comparison of CT vs. H120.

Supplementary Table 4 Statistics of differentially expressed genes (DEGs) with *P* < 0.01, FDR-adjusted *P* = 0.05 and |fold change| >2 in liver in comparison of CT vs. H30.

Supplementary Table 5 Statistics of differentially expressed genes (DEGs) with *P* < 0.01, FDR-adjusted *P* = 0.05 and |fold change| >2 in liver in comparison of CT vs. H60.

Supplementary Table 6 Statistics of differentially expressed genes (DEGs) with *P* < 0.01, FDR-adjusted *P* = 0.05 and |fold change| >2 in liver in comparison of CT vs. H120.

Supplementary Table 7 Statistics of differentially expressed genes (DEGs) with *P* < 0.01, FDR-adjusted *P* = 0.05 and |fold change| >2 in adrenal glands in comparison of CT vs. H30.

Supplementary Table 8 Statistics of differentially expressed genes (DEGs) with *P* < 0.01, FDR-adjusted *P* = 0.05 and |fold change| >2 in adrenal glands in comparison of CT vs. H60.

Supplementary Table 9 Statistics of differentially expressed genes (DEGs) with *P* < 0.01, FDR-adjusted *P* = 0.05 and |fold change| >2 in adrenal glands in comparison of CT vs. H120.

Supplementary Table 10 Significantly enriched biological processes (BPs) from differentially expressed genes (DEGs) identified in blood, liver, and adrenal glands.

Supplementary Table 11 Detailed information of genes in rat tissues and their overlapped cow single-nucleotide polymorphisms (SNPs) (*P* < 0.05).
