# Supplementary figures and images for: Comprehensive RNA-Seq Profiling Reveals Temporal and Tissue-Specific Changes in Gene Expression in Sprague–Dawley Rats as Response to Heat Stress Challenges
Source: Front Genet. 2021 Apr 9;12:651979. doi: 10.3389/fgene.2021.651979 (PMC8063118; doi:10.3389/fgene.2021.651979)

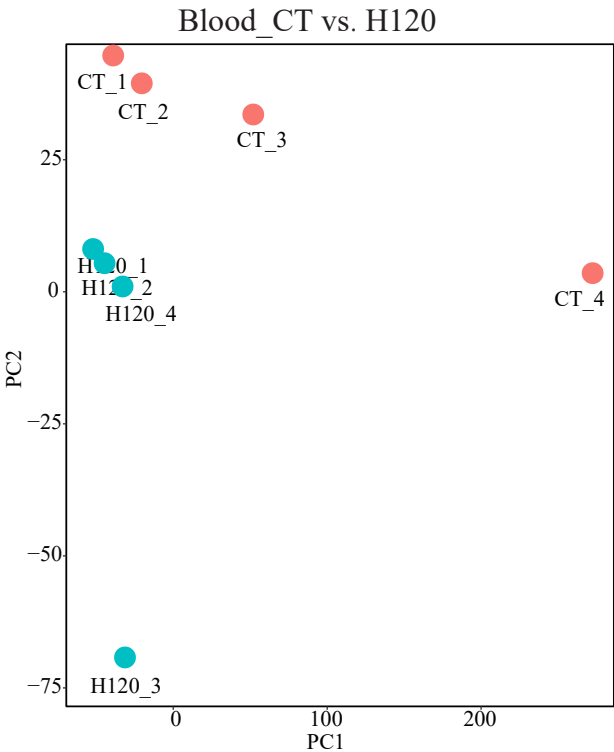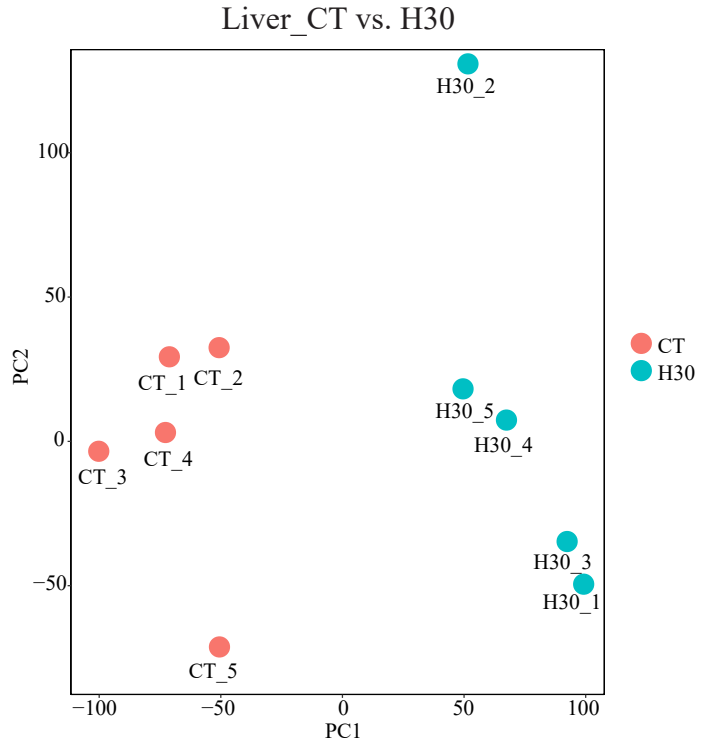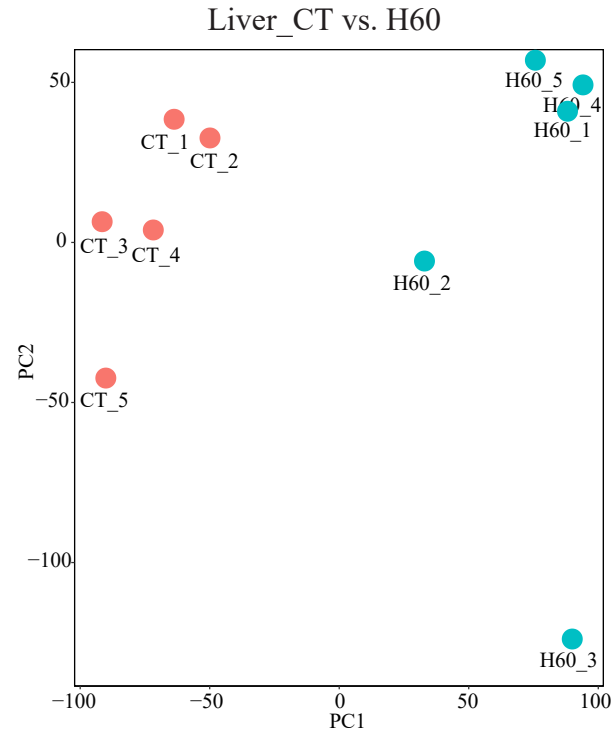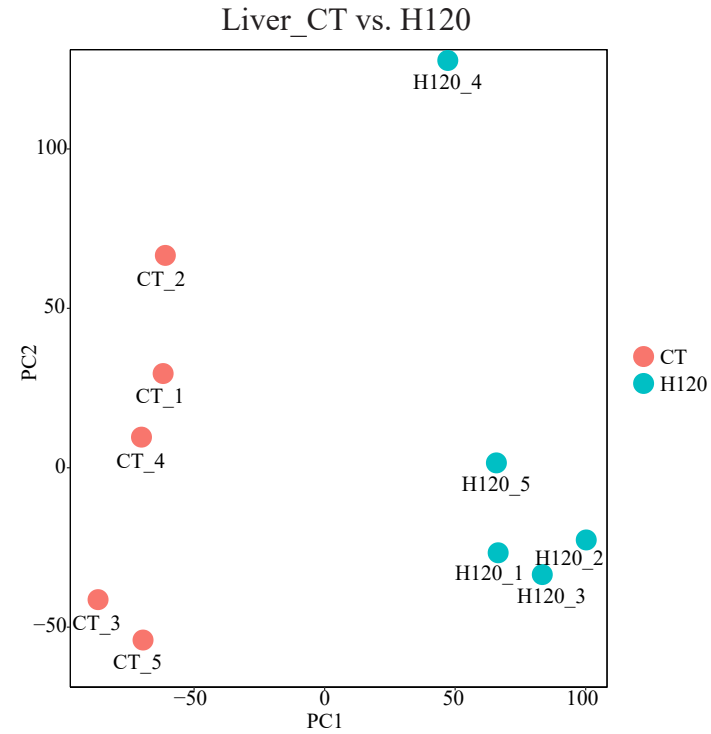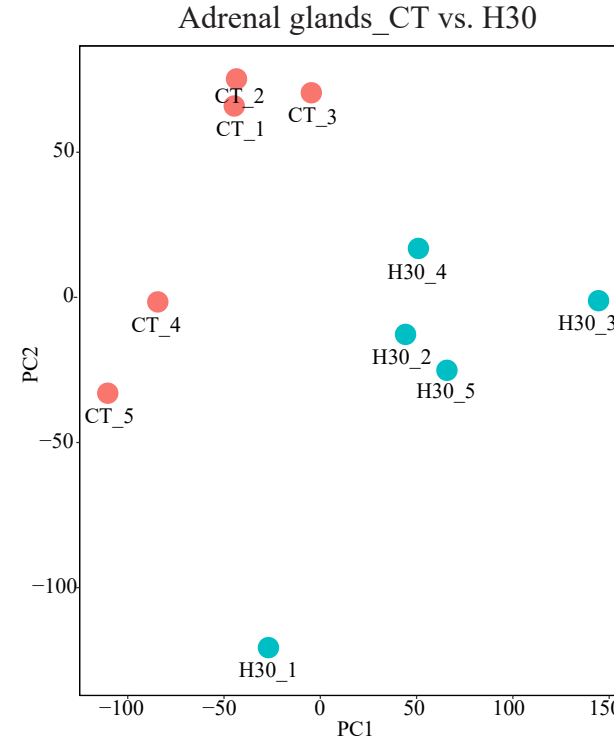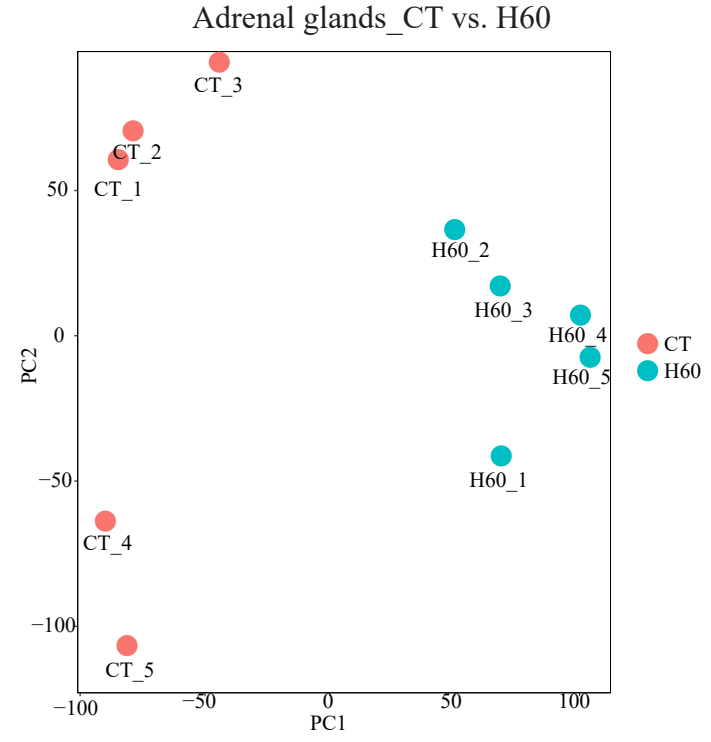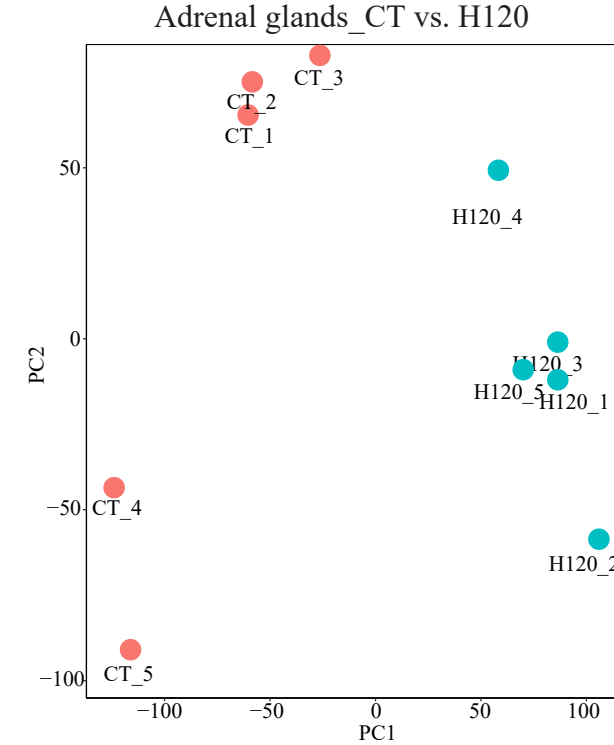

Supplement: Supplementary Figure 1 — The principal component analysis (PCA) of samples in each comparison of tissue. CT means rats were kept at 22 ± 1°C and relative humidity 50%; H30, H60, and H120 mean rats were kept at 42°C for 30, 60, and 120 min, and the relative humidity 50% conditions. [file Data_Sheet_1.PDF]

A CT vs. H120

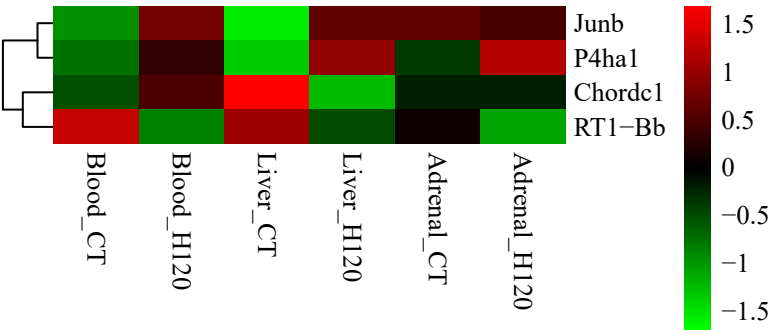

B Liver

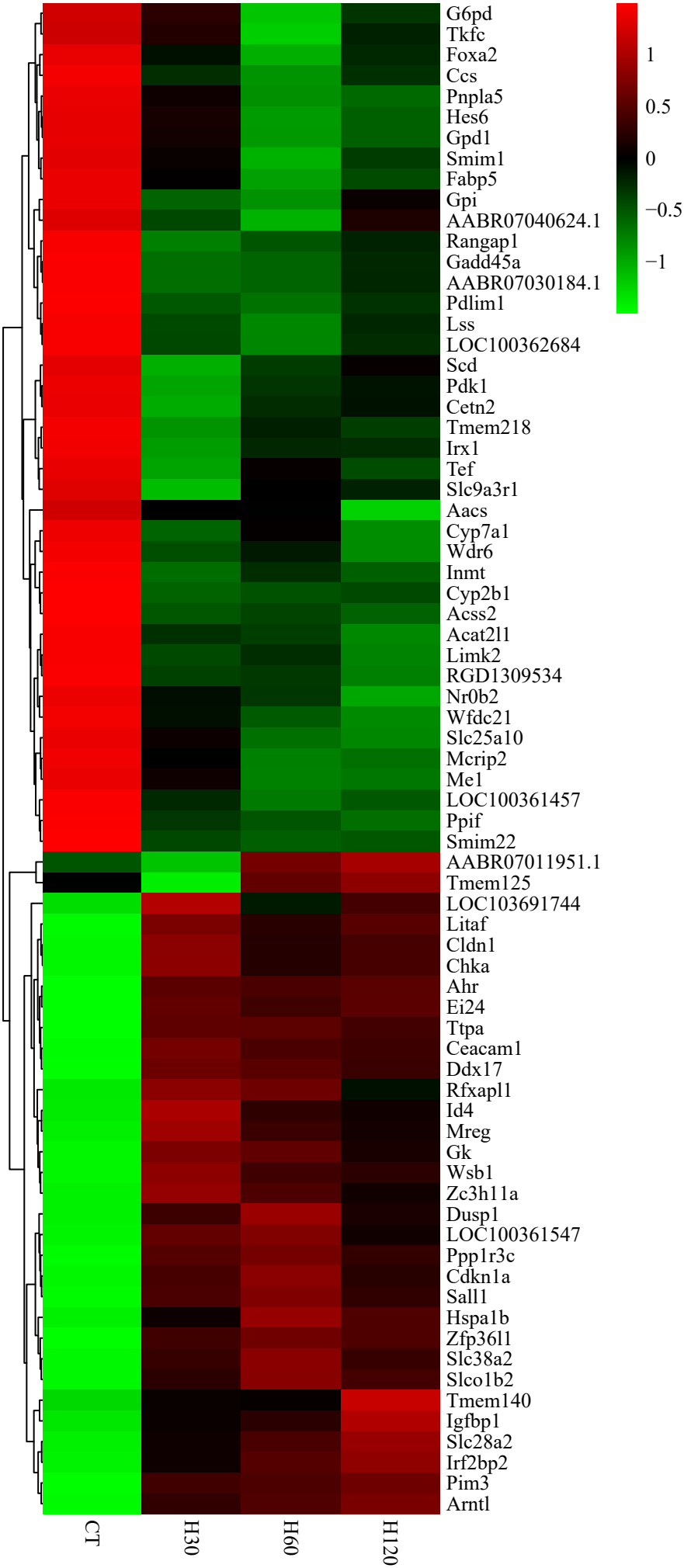

C Adrenal glands

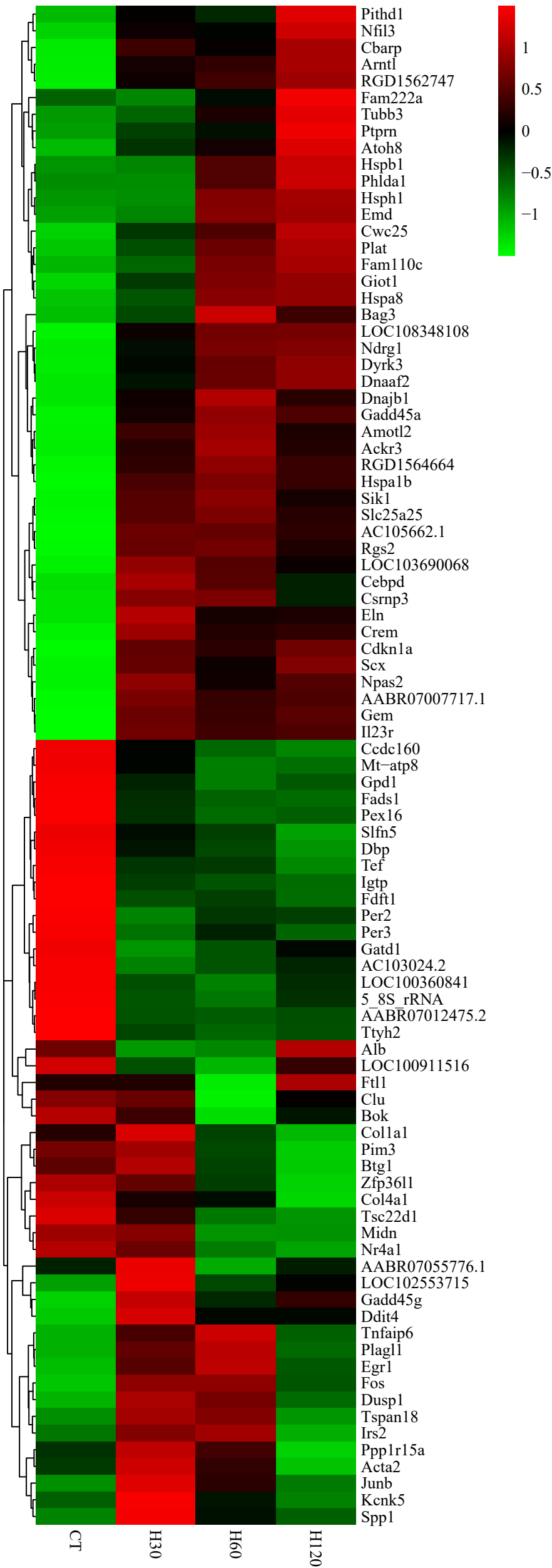

Supplement: Supplementary Figure 2 — Expression patterns of shared genes identified in varies conditions. (A) Heat map of four shared DEGs of blood, liver, and adrenal gland tissues in CT vs. H120. (B) Heat map of 73 shared DEGs of liver identified in comparisons of CT vs. H30, CT vs. H60, and CT vs. H120. (C) Heat map of 91 shared DEGs of adrenal glands identified in comparisons of CT vs. H30, CT vs. H60, and CT vs. H120. [file Data_Sheet_2.PDF]
