# Supplementary material for: Comprehensive RNA-Seq Profiling Reveals Temporal and Tissue-Specific Changes in Gene Expression in Sprague–Dawley Rats as Response to Heat Stress Challenges
Source: Front Genet. 2021 Apr 9;12:651979. doi: 10.3389/fgene.2021.651979 (PMC8063118; doi:10.3389/fgene.2021.651979)
Supplement: Supplementary Table 1 — Quality of sequencing reads of liver and adrenal glands in the H30 and H60 groups. [file Data_Sheet_3.docx]

Table S1. Quality of sequencing reads of liver and adrenal glands in the H30 and H60 groups.

| Sample | Raw Reads (bp) | Clean Reads (bp) | Raw Base(G) | Clean Base(G) | Effective Rate (%) | Error Rate (%) | Q20  (%) | Q30  (%) | GC Content (%) |
| --- | --- | --- | --- | --- | --- | --- | --- | --- | --- |
| Liver_H30_1 | 37450595 | 35670927 | 11.24 | 11.00 | 97.93 | 0.02 | 97.19 | 92.31 | 46.84 |
| Liver_H30_2 | 38679886 | 36887886 | 11.60 | 11.38 | 98.06 | 0.02 | 97.38 | 92.91 | 48.39 |
| Liver_H30_3 | 37587998 | 35242770 | 11.28 | 10.89 | 96.58 | 0.02 | 97.11 | 92.36 | 46.94 |
| Liver_H30_4 | 36550129 | 34547760 | 10.97 | 10.73 | 97.85 | 0.02 | 96.92 | 92.02 | 47.52 |
| Liver_H30_5 | 36383382 | 34557764 | 10.92 | 10.67 | 97.73 | 0.02 | 97.31 | 92.75 | 48.17 |
| Means | 37330398 | 35381421.4 | 11.20 | 10.93 | 97.63 | 0.02 | 97.18 | 92.47 | 47.57 |
| Liver_H60_1 | 42163010 | 40300061 | 12.65 | 12.40 | 98.05 | 0.02 | 97.57 | 93.29 | 49.51 |
| Liver_H60_2 | 41449293 | 39450039 | 12.43 | 12.17 | 97.90 | 0.02 | 97.39 | 92.91 | 48.98 |
| Liver_H60_3 | 43349392 | 41332243 | 13.00 | 12.73 | 97.87 | 0.02 | 97.48 | 93.17 | 48.66 |
| Liver_H60_4 | 47936311 | 45307362 | 14.38 | 13.98 | 97.20 | 0.02 | 97.34 | 92.80 | 49.10 |
| Liver_H60_5 | 40639187 | 38626582 | 12.19 | 11.86 | 97.31 | 0.02 | 97.64 | 93.47 | 49.14 |
| Means | 43107439 | 41003257.4 | 12.93 | 12.63 | 97.67 | 0.02 | 97.48 | 93.13 | 49.08 |
| Adrenal glands_H30_1 | 40536991 | 34172364 | 12.16 | 11.86 | 97.48 | 0.02 | 97.50 | 93.13 | 48.81 |
| Adrenal glands_H30_2 | 34297370 | 32431332 | 10.29 | 10.01 | 97.29 | 0.02 | 97.34 | 92.75 | 47.16 |
| Adrenal glands_H30_3 | 34950049 | 32462295 | 10.49 | 9.95 | 94.92 | 0.02 | 97.47 | 93.00 | 45.57 |
| Adrenal glands_H30_4 | 34422059 | 31141651 | 10.33 | 10.07 | 97.55 | 0.02 | 97.49 | 93.08 | 47.75 |
| Adrenal glands_H30_5 | 32036345 | 31408214 | 9.61 | 9.35 | 97.24 | 0.02 | 97.44 | 92.94 | 47.34 |
| Means | 35248563 | 32323171.2 | 10.58 | 10.25 | 96.90 | 0.02 | 97.45 | 92.98 | 47.33 |
| Adrenal glands_H60_1 | 39137112 | 37088350 | 11.74 | 11.46 | 97.57 | 0.02 | 97.36 | 92.78 | 48.33 |
| Adrenal glands_H60_2 | 31786811 | 30047147 | 9.54 | 9.29 | 97.37 | 0.02 | 97.29 | 92.73 | 48.52 |
| Adrenal glands_H60_3 | 34200342 | 32459906 | 10.26 | 9.95 | 96.97 | 0.02 | 97.71 | 93.69 | 48.27 |
| Adrenal glands_H60_4 | 36328045 | 34506210 | 10.90 | 10.60 | 97.23 | 0.02 | 97.59 | 93.34 | 48.81 |
| Adrenal glands_H60_5 | 29072607 | 27725160 | 8.72 | 8.49 | 97.35 | 0.02 | 97.83 | 93.96 | 49.23 |
| Means | 34104983 | 32365354.6 | 10.23 | 9.96 | 97.30 | 0.02 | 97.56 | 93.30 | 48.63 |

Note: H30 and H60 mean 42 ℃ heat stress for 30 min and 60 min, respectively.

Table S2. Descriptive statistics of reads generated by 48 RNA-seq libraries alignment to reference genome.

| Sample | Read number | Reads mapped in pairs % | | Broken pairs % | Non-mapped reads % | uniquely mapped reads | Map to genes % | Mapped to intergenic % |
| --- | --- | --- | --- | --- | --- | --- | --- | --- |
| Blood_CT1 | 70809870 | | 74.57 | 22.58 | 2.84 | 57422304 | 98.71 | 1.29 |
| Blood_CT2 | 62033200 | | 72.30 | 24.96 | 2.75 | 50014282 | 98.77 | 1.23 |
| Blood_CT3 | 70235814 | | 76.28 | 20.08 | 3.64 | 59765007 | 99.03 | 0.97 |
| Blood_CT4 | 59413984 | | 72.32 | 23.17 | 4.51 | 48894935 | 97.07 | 2.93 |
| Means | 65623217 | | 73.87 | 22.70 | 3.44 | 54024132 | 98.40 | 1.61 |
| Blood_H120_1 | 72505438 | | 70.41 | 26.27 | 3.33 | 58919950 | 99.01 | 0.99 |
| Blood_H120_2 | 63610226 | | 75.11 | 20.95 | 3.94 | 53259456 | 99.18 | 0.82 |
| Blood_H120_3 | 97059004 | | 79.37 | 16.25 | 4.38 | 83344412 | 99.09 | 0.91 |
| Blood_H120_4 | 66567392 | | 77.30 | 18.52 | 4.18 | 56139147 | 99.14 | 0.86 |
| Means | 74935515 | | 75.55 | 20.50 | 3.96 | 62915741.25 | 99.11 | 0.90 |
| Liver_CT1 | 74105438 | | 87.91 | 7.13 | 4.96 | 66337044 | 93.95 | 6.05 |
| Liver_CT2 | 64347374 | | 88.07 | 7.86 | 4.06 | 57966111 | 92.88 | 7.12 |
| Liver_CT3 | 53513200 | | 93.34 | 3.45 | 2.21 | 48756324 | 92.98 | 7.02 |
| Liver_CT4 | 52510642 | | 86.58 | 7.33 | 6.09 | 46191147 | 93.17 | 6.83 |
| Liver_CT5 | 59339084 | | 93.37 | 3.16 | 3.48 | 53467763 | 92.86 | 7.14 |
| Means | 60763148 | | 89.85 | 5.79 | 4.16 | 54543677.8 | 93.17 | 6.83 |
| Liver_H30_1 | 74901190 | | 92.04 | 5.08 | 2.88 | 67222317 | 92.86 | 7.14 |
| Liver_H30_2 | 77359772 | | 90.76 | 6.21 | 3.03 | 69261739 | 92.50 | 7.50 |
| Liver_H30_3 | 75175996 | | 90.94 | 5.72 | 3.35 | 66770207 | 91.73 | 8.27 |
| Liver_H30_4 | 73100258 | | 91.06 | 5.53 | 3.41 | 64855260 | 92.57 | 7.43 |
| Liver_H30_5 | 72766764 | | 91.89 | 5.19 | 2.93 | 65145917 | 92.84 | 7.16 |
| Means | 74660796 | | 91.34 | 5.55 | 3.12 | 66651088 | 92.50 | 7.50 |
| Liver_H60_1 | 84326020 | | 91.16 | 6.09 | 2.76 | 76633407 | 94.01 | 5.99 |
| Liver_H60_2 | 82898586 | | 92.87 | 4.41 | 2.72 | 75380917 | 93.25 | 6.75 |
| Liver_H60_3 | 86698784 | | 92.24 | 4.71 | 3.04 | 76924469 | 92.97 | 7.03 |
| Liver_H60_4 | 95872622 | | 92.42 | 4.55 | 3.03 | 86377530 | 93.67 | 6.33 |
| Liver_H60_5 | 81278374 | | 93.03 | 4.24 | 2.73 | 73570289 | 93.69 | 6.31 |
| Means | 86214877 | | 92.34 | 4.80 | 2.86 | 77777322.4 | 93.52 | 6.48 |
| Liver_H120_1 | 53978328 | | 85.52 | 8.50 | 5.98 | 47109425 | 94.02 | 5.98 |
| Liver_H120_2 | 55764470 | | 85.92 | 8.55 | 5.53 | 49220222 | 94.57 | 5.43 |
| Liver_H120_3 | 61440236 | | 85.80 | 8.74 | 5.46 | 54447339 | 94.22 | 5.78 |
| Liver_H120_4 | 77632214 | | 88.25 | 8.26 | 3.49 | 70608362 | 93.70 | 6.30 |
| Liver_H120_5 | 69491974 | | 83.69 | 9.30 | 6.38 | 61105939 | 94.02 | 5.98 |
| Means | 63661444 | | 85.84 | 8.67 | 5.37 | 56498257.4 | 94.11 | 5.89 |
| Adrenal glands_CT1 | 53991038 | | 92.30 | 4.77 | 2.92 | 46925378 | 88.12 | 11.88 |
| Adrenal glands_CT2 | 67994712 | | 90.94 | 4.73 | 4.33 | 57768437 | 86.82 | 13.18 |
| Adrenal glands_CT3 | 61623484 | | 91.70 | 4.41 | 3.89 | 52798248 | 88.07 | 11.93 |
| Adrenal glands_CT4 | 51706978 | | 93.44 | 4.02 | 2.54 | 45624841 | 87.28 | 12.72 |
| Adrenal glands_CT5 | 57712120 | | 90.37 | 6.23 | 3.40 | 50605768 | 88.05 | 11.95 |
| Means | 58605666 | | 91.75 | 4.83 | 3.42 | 50744534.4 | 87.67 | 12.33 |
| Adrenal glands_H30_1 | 81073982 | | 91.55 | 5.20 | 3.25 | 70451691 | 88.55 | 11.45 |
| Adrenal glands_H30_2 | 68594740 | | 91.99 | 4.97 | 3.04 | 59843452 | 89.38 | 10.62 |
| Adrenal glands_H30_3 | 69900098 | | 91.95 | 4.49 | 3.56 | 59824190 | 89.51 | 10.49 |
| Adrenal glands_H30_4 | 68844118 | | 92.27 | 4.72 | 3.00 | 60261588 | 89.10 | 10.90 |
| Adrenal glands_H30_5 | 64072690 | | 92.56 | 4.47 | 2.97 | 55668409 | 89.03 | 10.97 |
| Means | 70497126 | | 92.06 | 4.77 | 3.16 | 61209866 | 89.11 | 10.89 |
| Adrenal glands_H60_1 | 78274224 | | 91.85 | 4.92 | 3.23 | 67012178 | 89.29 | 10.71 |
| Adrenal glands_H60_2 | 63573622 | | 91.58 | 5.12 | 3.30 | 54524028 | 88.51 | 11.49 |
| Adrenal glands_H60_3 | 68400684 | | 91.81 | 4.93 | 3.27 | 58230932 | 89.14 | 10.86 |
| Adrenal glands_H60_4 | 72656090 | | 91.52 | 5.39 | 3.10 | 61446290 | 89.54 | 10.46 |
| Adrenal glands_H60_5 | 58145214 | | 91.45 | 5.40 | 3.16 | 49640316 | 87.66 | 12.34 |
| Means | 68209967 | | 91.64 | 5.15 | 3.21 | 58170748.8 | 88.83 | 11.17 |
| Adrenal glands_H120_1 | 67209702 | | 85.96 | 8.27 | 5.77 | 55816883 | 88.79 | 11.21 |
| Adrenal glands_H120_2 | 61742408 | | 86.21 | 8.22 | 5.58 | 51818950 | 88.34 | 11.66 |
| Adrenal glands_H120_3 | 68202116 | | 85.11 | 9.48 | 5.42 | 57932896 | 88.39 | 11.61 |
| Adrenal glands_H120_4 | 66671020 | | 88.21 | 7.58 | 4.21 | 57750365 | 88.30 | 11.70 |
| Adrenal glands_H120_5 | 66885812 | | 85.53 | 8.55 | 5.92 | 56564614 | 87.18 | 12.82 |
| Means | 66142212 | | 86.20 | 8.42 | 5.38 | 55976741.6 | 88.20 | 11.80 |

Note: The CT mean rats were housed at 22 ± 1 ℃ and relative humidity 50%. H30 and H60 mean 42 ℃ heat stress for 30 min and 60 min with relative humidity 50%, respectively.

Table S3. The statistics of differentially expressed genes (DEG) with *P* < 0.05 and |Fold change| > 2 in blood in comparison of CT vs. H120.

| Feature ID | Gene name | Position | P-value | Fold change | FDR-adjusted P value * |
| --- | --- | --- | --- | --- | --- |
| ENSRNOG00000000451.5 | RT1-Ba | 20:4066131-4070721 | 0.0011 | -4.16 | 1 |
| ENSRNOG00000000562.6 | Prf1 | 20:30915212-30921382 | 0.0385 | 2.86 | 1 |
| ENSRNOG00000000851.8 | Bag6 | 20:5125348-5138084 | 0.0128 | 2.96 | 1 |
| ENSRNOG00000002946.3 | Socs3 | 10:106975177-106976040 | 0.0138 | 4.88 | 1 |
| ENSRNOG00000003895.7 | Rgs1 | 13:61066152-61070599 | 0.0184 | 3.28 | 1 |
| ENSRNOG00000004662.6 | AC115277.1 | 7:15385867-15386419 | 0.0397 | 4.56 | 1 |
| ENSRNOG00000005825.8 | Lyz2 | 7:60335968-60341264 | 0.0001 | -3.94 | 1 |
| ENSRNOG00000009342.6 | Fcnb | 3:6617750-6626284 | 0.0011 | -7.49 | 1 |
| ENSRNOG00000010029.6 | Ubald2 | 10:105393071-105396863 | 0.0122 | 2.28 | 1 |
| ENSRNOG00000011557.3 | S100a8 | 2:190073814-190074354 | 0.0195 | -2.68 | 1 |
| ENSRNOG00000011647.5 | S100a6 | 2:190007215-190008511 | 0.0007 | -6.14 | 1 |
| ENSRNOG00000011821.4 | S100a4 | 2:189997128-189999604 | 0.0140 | -2.63 | 1 |
| ENSRNOG00000015753.6 | Epn1 | 1:72294152-72311856 | 0.0011 | 2.82 | 1 |
| ENSRNOG00000015903.6 | Add2 | 4:117743709-117882464 | 0.0436 | 2.44 | 1 |
| ENSRNOG00000017117.7 | Ybx2 | 10:56546709-56551863 | 0.0008 | 4.58 | 1 |
| ENSRNOG00000017645.6 | Mylpf | 1:198655741-198658583 | 0.0412 | 6.73 | 1 |
| ENSRNOG00000018087.5 | Vim | 17:80882665-80891212 | 0.0134 | -2.36 | 1 |
| ENSRNOG00000019090.6 | Cct3 | 2:187668795-187693610 | 0.0415 | 2.22 | 1 |
| ENSRNOG00000019568.6 | Jund | 16:20485028-20486707 | 0.0160 | 2.67 | 1 |
| ENSRNOG00000020349.3 | Rab3il1 | 1:226077119-226084830 | 0.0359 | 4.16 | 1 |
| ENSRNOG00000020845.6 | Tyrobp | 1:88875374-88879303 | 0.0109 | -2.20 | 1 |
| ENSRNOG00000024689.6 | Hopx | 14:33354806-33362551 | 0.0097 | -4.75 | 1 |
| ENSRNOG00000025040.6 | Gng10 | 5:76182120-76189013 | 0.0355 | -2.68 | 1 |
| ENSRNOG00000026643.5 | Chordc1 | 8:17421556-17446165 | 0.0470 | 4.02 | 1 |
| ENSRNOG00000026653.1 | Hcar2 | 12:38160463-38161546 | 0.0496 | 5.20 | 1 |
| ENSRNOG00000027767.5 | Slc38a5 | X:14963920-14972675 | 0.0054 | 3.68 | 1 |
| ENSRNOG00000027990.4 | Crip1 | 6:137959170-137967042 | 0.0002 | -3.67 | 1 |
| ENSRNOG00000029512.1 | LOC100361854 | X:115495697-115496045 | 0.0111 | 2.12 | 1 |
| ENSRNOG00000031090.6 | RT1-CE7 | 20:4694471-4896970 | 0.0140 | 2.31 | 1 |
| ENSRNOG00000031315.2 | Rpl36al | 10:57251252-57251573 | 0.0002 | 106.37 | 1 |
| ENSRNOG00000031540.5 | LOC100362384 | 13:89253616-89254171 | 0.0325 | 5.10 | 1 |
| ENSRNOG00000032401.3 | H3f3c | 7:94777701-94778696 | 0.0495 | -2.31 | 1 |
| ENSRNOG00000032708.5 | RT1-Bb | 20:4039412-4049711 | 0.0216 | -3.67 | 1 |
| ENSRNOG00000032844.4 | RT1-Da | 20:4127643-4132616 | 0.0025 | -3.74 | 1 |
| ENSRNOG00000033215.6 | RT1-Db1 | 20:4087617-4097190 | 0.0244 | -2.73 | 1 |
| ENSRNOG00000036701.4 | Actg1 | 10:109519133-109520846 | 0.0001 | 39.70 | 1 |
| ENSRNOG00000042838.3 | Junb | 19:26092973-26094756 | 0.0313 | 2.32 | 1 |
| ENSRNOG00000046600.1 | AABR07015066.1 | 14:46588405-46588816 | 0.0077 | 3.31 | 1 |
| ENSRNOG00000047854.2 | RT1-DMa | 20:3935528-3938915 | 0.0056 | -6.75 | 1 |
| ENSRNOG00000050108.1 | LOC100911319 | 7:2351932-2353875 | 0.0006 | 13.86 | 1 |
| ENSRNOG00000050655.1 | P4ha1 | 20:28920615-28971966 | 0.0069 | 12.19 | 1 |
| ENSRNOG00000052219.1 | Gm2a | 10:40438355-40450930 | 0.0171 | -3.48 | 1 |
| ENSRNOG00000055053.1 | AC120246.2 | 19:25839420-25839848 | 0.0482 | 2.82 | 1 |
| ENSRNOG00000055241.1 | AABR07053500.1 | 3:110540716-110541170 | 0.0085 | 6.16 | 1 |
| ENSRNOG00000055956.1 | AABR07015078.1 | 14:46649970-46650381 | 0.0242 | 3.00 | 1 |
| ENSRNOG00000056747.1 | AABR07015055.1 | 14:46523588-46523999 | 0.0363 | 2.85 | 1 |
| ENSRNOG00000058083.1 | Metazoa_SRP | 6:91680228-91680528 | 0.0065 | 9.45 | 1 |
| ENSRNOG00000058555.1 | 7SK | 8:85480291-85480622 | 0.0018 | 31.37 | 1 |
| ENSRNOG00000059538.1 | Clec2g | 4:162934194-162943981 | 0.0433 | -3.23 | 1 |
| ENSRNOG00000059586.1 | AABR07015080.2 | 14:46653034-46653445 | 0.0236 | 3.04 | 1 |
| ENSRNOG00000059900.1 | Bst2 | 16:19938783-19942353 | 0.0190 | 3.35 | 1 |
| ENSRNOG00000060289.1 | 7SK | X:23467546-23467864 | 0.0191 | 9.99 | 1 |
| ENSRNOG00000060896.1 | AABR07063424.1 | 6:30638732-30639143 | 0.0292 | 2.85 | 1 |
| ENSRNOG00000061096.1 | Rn7sl1 | 6:91456907-91457207 | 0.0095 | 13.83 | 1 |

*FDR: false discovery rate. The FDR-adjusted P value was sorted from lowest to highest. The CT mean rats were housed at 22 ± 1 ℃ and relative humidity 50%. H120 means 42 ℃ heat stress for 120 min with relative humidity 50%.

Table S4. Statistics of differentially expressed genes (DEG) with *P* < 0.01, FDR-adjusted P value = 0.05 and |Fold change| > 2 in liver in comparison of CT vs. H30.

| Feature ID | Gene name | Position | P-value | Fold change | FDR-adjusted P value* |
| --- | --- | --- | --- | --- | --- |
| ENSRNOG00000028505.6 | Rps18 | 20:5441875-5445553 | 0.0E+00 | -2.12 | 0.0000 |
| ENSRNOG00000031053.3 | Mt-nd4l | MT:9869-10166 | 1.1E-37 | 9.74 | 0.0000 |
| ENSRNOG00000050647.2 | Hspa1b | 20:4877323-4879779 | 1.5E-34 | 101.69 | 0.0000 |
| ENSRNOG00000001049.7 | Tpt1 | 15:57891679-57894504 | 4.8E-28 | 2.33 | 0.0000 |
| ENSRNOG00000034116.6 | Gk | X:54227396-54303864 | 7.1E-28 | 7.49 | 0.0000 |
| ENSRNOG00000033024.2 | AC099453.1 | 5:78392714-78393143 | 6.3E-25 | -6.40 | 0.0000 |
| ENSRNOG00000001442.3 | Por | 12:23998410-24046814 | 9.5E-23 | -4.87 | 0.0000 |
| ENSRNOG00000029971.3 | Mt-nd5 | MT:11735-13565 | 3.0E-22 | 5.02 | 0.0000 |
| ENSRNOG00000021027.6 | Dbp | 1:101687854-101692846 | 4.4E-21 | -20.55 | 0.0000 |
| ENSRNOG00000009057.4 | Sec62 | 2:116189861-116219750 | 3.2E-19 | -3.77 | 0.0000 |
| ENSRNOG00000006779.6 | Crot | 4:22081603-22116265 | 4.1E-19 | 5.31 | 0.0000 |
| ENSRNOG00000010658.6 | LOC103691744 | 2:264266983-264293046 | 4.2E-19 | 4.35 | 0.0000 |
| ENSRNOG00000002947.8 | Dpt | 13:83073543-83102401 | 5.6E-19 | -2.91 | 0.0000 |
| ENSRNOG00000003232.4 | Slc9a3r1 | 10:103713044-103730145 | 1.2E-18 | -4.38 | 0.0000 |
| ENSRNOG00000016220.7 | Rpl12 | 3:12009577-12011666 | 1.8E-18 | 2.80 | 0.0000 |
| ENSRNOG00000019578.7 | Rps16 | 1:85405511-85408444 | 5.3E-18 | -2.82 | 0.0000 |
| ENSRNOG00000003977.5 | Dusp1 | 10:16970625-16973418 | 1.9E-17 | 7.91 | 0.0000 |
| ENSRNOG00000015774.5 | Mreg | 9:79490049-79545024 | 9.5E-17 | 4.85 | 0.0000 |
| ENSRNOG00000062289.1 | AABR07055846.2 | 7:12664046-12665625 | 1.1E-16 | 5.40 | 0.0000 |
| ENSRNOG00000016791.8 | Chka | 1:219077770-219126220 | 2.6E-16 | 6.05 | 0.0000 |
| ENSRNOG00000031033.4 | Mt-nd2 | MT:3903-4942 | 4.3E-16 | 4.92 | 0.0000 |
| ENSRNOG00000018755.6 | Acss2 | 3:151032951-151075856 | 7.0E-16 | -2.85 | 0.0000 |
| ENSRNOG00000000571.6 | Psap | 20:29831313-29856875 | 1.7E-15 | -2.38 | 0.0000 |
| ENSRNOG00000053210.1 | Zc3h11a | 13:50196041-50234862 | 3.1E-15 | 5.12 | 0.0000 |
| ENSRNOG00000037673.3 | AABR07071891.1 | 1:84573495-84573959 | 7.1E-15 | -3.26 | 0.0000 |
| ENSRNOG00000002520.5 | Litaf | 10:4719712-4763510 | 1.7E-14 | 5.10 | 0.0000 |
| ENSRNOG00000009982.6 | Pnp | 15:27875910-27883350 | 3.0E-14 | -2.40 | 0.0000 |
| ENSRNOG00000002896.7 | Prdx6 | 13:79077032-79088127 | 4.0E-14 | -2.08 | 0.0000 |
| ENSRNOG00000019189.7 | Acat2 | 1:47972398-47992653 | 5.9E-14 | -4.46 | 0.0000 |
| ENSRNOG00000049075.2 | Fabp5 | 2:93981655-93985378 | 6.0E-14 | -4.57 | 0.0000 |
| ENSRNOG00000028690.3 | LOC100362366 | 3:24600602-24601063 | 7.0E-14 | -2.96 | 0.0000 |
| ENSRNOG00000008921.4 | Dynll2 | 10:75262536-75270561 | 1.0E-13 | -2.20 | 0.0000 |
| ENSRNOG00000000443.7 | LOC103689965 | 20:4302346-4508214 | 1.4E-13 | -3.01 | 0.0000 |
| ENSRNOG00000047708.2 | Gstz1 | 6:111176797-111187244 | 2.7E-13 | -2.12 | 0.0000 |
| ENSRNOG00000060229.1 | Arf1 | 10:45563546-45579029 | 3.3E-13 | -4.85 | 0.0000 |
| ENSRNOG00000019895.6 | Nedd8 | 15:34340606-34352673 | 4.0E-13 | -2.57 | 0.0000 |
| ENSRNOG00000047746.1 | AABR07000398.1 | 1:11963835-11968939 | 7.0E-13 | 3.42 | 0.0000 |
| ENSRNOG00000009875.2 | Akr1b7 | 4:61850347-61862526 | 7.7E-13 | 3.02 | 0.0000 |
| ENSRNOG00000020775.8 | Cyp2b2 | 1:83103924-83119193 | 7.8E-13 | -6.74 | 0.0000 |
| ENSRNOG00000050547.2 | Syngr2 | 10:106812738-106816675 | 9.5E-13 | -4.07 | 0.0000 |
| ENSRNOG00000005266.7 | Amdhd1 | 7:34361632-34406318 | 1.3E-12 | 2.92 | 0.0000 |
| ENSRNOG00000012123.3 | Fdx1 | 8:56373722-56393233 | 1.6E-12 | 2.51 | 0.0000 |
| ENSRNOG00000030712.6 | RT1-A2 | 20:5374984-5378539 | 3.1E-12 | -2.71 | 0.0000 |
| ENSRNOG00000021808.4 | Tecr | 19:24705480-24732024 | 4.1E-12 | -2.05 | 0.0000 |
| ENSRNOG00000033473.5 | Rpl36 | 9:10441258-10441834 | 4.9E-12 | 2.17 | 0.0000 |
| ENSRNOG00000050761.1 | LOC100909524 | 6:127500015-127508452 | 4.9E-12 | -6.35 | 0.0000 |
| ENSRNOG00000059593.1 | Cetn2 | X:152927717-152932953 | 5.0E-12 | -3.29 | 0.0000 |
| ENSRNOG00000028382.3 | Rfxapl1 | 2:144213402-144217600 | 6.0E-12 | 64.60 | 0.0000 |
| ENSRNOG00000049394.3 | LOC679739 | 1:32573227-32581781 | 6.1E-12 | -2.50 | 0.0000 |
| ENSRNOG00000016099.2 | Id4 | 17:16692556-16695126 | 6.2E-12 | 3.60 | 0.0000 |
| ENSRNOG00000005615.6 | Gadd45a | 4:97782511-97784842 | 7.6E-12 | -3.71 | 0.0000 |
| ENSRNOG00000023150.5 | Gpi | 1:90063410-90091287 | 7.6E-12 | -2.94 | 0.0000 |
| ENSRNOG00000011330.7 | Ces2a | 19:59196-64303 | 8.5E-12 | -3.74 | 0.0000 |
| ENSRNOG00000053207.1 | AABR07026125.1 | 16:64982381-64984767 | 9.9E-12 | 5.95 | 0.0000 |
| ENSRNOG00000009488.4 | Cyp7a1 | 5:19358733-19368431 | 1.3E-11 | -5.09 | 0.0000 |
| ENSRNOG00000023387.5 | LOC100912599 | 1:31264754-31273159 | 1.5E-11 | -2.47 | 0.0000 |
| ENSRNOG00000025768.6 | Clk1 | 9:65296777-65307995 | 1.6E-11 | 2.95 | 0.0000 |
| ENSRNOG00000016097.7 | Kyat1 | 3:8752624-8766433 | 1.8E-11 | -3.02 | 0.0000 |
| ENSRNOG00000005542.8 | Apob | 6:33176777-33224997 | 2.0E-11 | 2.34 | 0.0000 |
| ENSRNOG00000000184.6 | Tmprss6 | 7:119659323-119689938 | 2.6E-11 | -2.39 | 0.0000 |
| ENSRNOG00000004196.7 | LOC108352650 | 6:91455332-91456696 | 3.7E-11 | -2.59 | 0.0000 |
| ENSRNOG00000047158.2 | Selenbp1 | 2:195416468-195423787 | 4.4E-11 | -2.22 | 0.0000 |
| ENSRNOG00000042344.1 | Smim22 | 10:10766207-10767389 | 7.2E-11 | -3.17 | 0.0000 |
| ENSRNOG00000058646.1 | Zfp36l1 | 6:103308044-103313074 | 7.8E-11 | 3.30 | 0.0000 |
| ENSRNOG00000016122.4 | Hmgcr | 2:27480225-27500654 | 1.0E-10 | -4.99 | 0.0000 |
| ENSRNOG00000012929.6 | Wsb1 | 10:66586923-66602987 | 1.3E-10 | 4.67 | 0.0000 |
| ENSRNOG00000016166.4 | Pdlim1 | 1:259308295-259357056 | 1.5E-10 | -2.82 | 0.0000 |
| ENSRNOG00000008414.8 | Bsg | 7:12875536-12882753 | 1.6E-10 | -2.11 | 0.0000 |
| ENSRNOG00000031579.2 | LOC100363469 | 10:78992579-78993045 | 1.8E-10 | 2.98 | 0.0000 |
| ENSRNOG00000006539.7 | Pex16 | 3:81283136-81292573 | 2.0E-10 | -2.44 | 0.0000 |
| ENSRNOG00000030680.4 | Ddx5 | 10:94980927-94988461 | 2.1E-10 | 2.15 | 0.0000 |
| ENSRNOG00000050445.2 | Gde1 | 1:188394813-188407132 | 2.7E-10 | -3.88 | 0.0000 |
| ENSRNOG00000047565.2 | Echs1 | 1:141821915-141830764 | 3.3E-10 | -6.23 | 0.0000 |
| ENSRNOG00000009481.6 | Ddhd1 | 15:19965186-20032191 | 3.5E-10 | 3.38 | 0.0000 |
| ENSRNOG00000006787.6 | Dhcr24 | 5:126164673-126191206 | 3.7E-10 | -2.79 | 0.0000 |
| ENSRNOG00000011250.6 | Inmt | 4:85381888-85386231 | 6.2E-10 | -57.86 | 0.0000 |
| ENSRNOG00000002218.5 | Stbd1 | 14:16976355-16979760 | 6.8E-10 | -2.13 | 0.0000 |
| ENSRNOG00000029707.3 | Mt-nd4 | MT:10159-11537 | 7.7E-10 | 3.43 | 0.0000 |
| ENSRNOG00000051257.2 | AABR07039356.2 | X:74333328-74337975 | 8.5E-10 | 22.00 | 0.0000 |
| ENSRNOG00000032297.2 | Msmo1 | 16:26859396-26875973 | 9.9E-10 | -2.37 | 0.0000 |
| ENSRNOG00000051286.2 | LOC100911516 | 9:101106891-101107575 | 1.1E-09 | 4.47 | 0.0000 |
| ENSRNOG00000030644.4 | Mt-nd1 | MT:2739-3694 | 1.2E-09 | 2.94 | 0.0000 |
| ENSRNOG00000033152.3 | Rps18l1 | 10:104521699-104522241 | 1.3E-09 | 2.42 | 0.0000 |
| ENSRNOG00000030538.5 | Slco1b2 | 4:175814117-175881768 | 1.4E-09 | 2.06 | 0.0000 |
| ENSRNOG00000038370.4 | AABR07034632.1 | 11:81707803-81735592 | 1.7E-09 | -2.24 | 0.0000 |
| ENSRNOG00000062155.1 | AC134224.2 | 1:221151826-221153278 | 1.8E-09 | 5.28 | 0.0000 |
| ENSRNOG00000005437.6 | Rida | 7:73256507-73270308 | 1.8E-09 | 2.99 | 0.0000 |
| ENSRNOG00000015335.6 | Rpl13 | 19:55917735-55919996 | 2.1E-09 | 2.28 | 0.0000 |
| ENSRNOG00000056596.1 | Alas1 | 8:114927721-114940177 | 2.6E-09 | -2.34 | 0.0000 |
| ENSRNOG00000046307.1 | Glyctk | 8:114848083-114853103 | 2.6E-09 | -2.11 | 0.0000 |
| ENSRNOG00000019708.6 | Ctsf | 1:220114227-220119976 | 2.8E-09 | -3.00 | 0.0000 |
| ENSRNOG00000059770.1 | AABR07044383.1 | 20:3791628-3796275 | 3.6E-09 | -4.89 | 0.0000 |
| ENSRNOG00000057333.1 | AABR07030184.1 | 10:74765384-74765600 | 3.8E-09 | -87.59 | 0.0000 |
| ENSRNOG00000059883.1 | LOC100912578 | 3:74226406-74229066 | 4.1E-09 | 2.40 | 0.0000 |
| ENSRNOG00000037853.2 | Rarres1 | 2:164650721-164684985 | 4.2E-09 | 2.73 | 0.0000 |
| ENSRNOG00000007793.6 | Pnrc1 | 5:48501471-48504511 | 4.6E-09 | 3.52 | 0.0000 |
| ENSRNOG00000029810.3 | LOC100911766 | 1:214454089-214473741 | 4.7E-09 | -2.53 | 0.0000 |
| ENSRNOG00000020480.5 | Fads1 | 1:226234073-226249138 | 5.7E-09 | -2.15 | 0.0000 |
| ENSRNOG00000042905.3 | RT1-T24-4 | 20:3189472-3197457 | 6.6E-09 | -2.22 | 0.0000 |
| ENSRNOG00000027784.4 | Tsku | 1:163317281-163328591 | 7.7E-09 | -3.78 | 0.0000 |
| ENSRNOG00000049484.3 | Atp9a | 3:165412802-165477771 | 8.1E-09 | -6.46 | 0.0000 |
| ENSRNOG00000026605.5 | Ifi27l2b | 6:127336304-127337791 | 8.4E-09 | -2.41 | 0.0000 |
| ENSRNOG00000026293.2 | Jun | 5:114011188-114014277 | 1.1E-08 | 4.42 | 0.0000 |
| ENSRNOG00000033803.5 | LOC100360841 | 9:69790830-69791196 | 1.1E-08 | -3.57 | 0.0000 |
| ENSRNOG00000054181.1 | LOC100361547 | 1:148240503-148287217 | 1.1E-08 | 2.98 | 0.0000 |
| ENSRNOG00000055765.1 | Rasl10b | KL568414.1:23533-32066 | 1.1E-08 | -4.35 | 0.0000 |
| ENSRNOG00000004172.6 | Pdk2 | 10:82839152-82852660 | 1.3E-08 | -3.19 | 0.0000 |
| ENSRNOG00000017558.6 | Tubb2a | 17:31493106-31498651 | 1.4E-08 | 3.59 | 0.0000 |
| ENSRNOG00000020698.6 | Rnd2 | 10:89376529-89380101 | 1.5E-08 | -2.48 | 0.0000 |
| ENSRNOG00000049604.2 | Mvk | 12:47904718-47919400 | 1.5E-08 | -3.87 | 0.0000 |
| ENSRNOG00000031506.4 | Ftl1 | 5:152325870-152326778 | 1.6E-08 | 2.75 | 0.0000 |
| ENSRNOG00000018494.6 | Ppp1r3c | 1:255371809-255376833 | 1.6E-08 | 2.82 | 0.0000 |
| ENSRNOG00000001517.6 | Pdk1 | 3:58530869-58558027 | 1.7E-08 | -3.80 | 0.0000 |
| ENSRNOG00000013552.6 | Scd | 1:264160128-264172729 | 2.1E-08 | -7.12 | 0.0000 |
| ENSRNOG00000007235.5 | Atp5mc1 | 10:83895946-83898527 | 2.3E-08 | -2.19 | 0.0000 |
| ENSRNOG00000054017.1 | LOC100912564 | 17:44522139-44526583 | 2.9E-08 | 2.29 | 0.0000 |
| ENSRNOG00000012748.4 | Vamp8 | 4:100250300-100252755 | 2.9E-08 | -2.05 | 0.0000 |
| ENSRNOG00000007139.2 | Ttpa | 5:34007919-34028452 | 3.0E-08 | 2.37 | 0.0000 |
| ENSRNOG00000060518.1 | AABR07015057.1 | 14:46523593-46529375 | 3.6E-08 | 2.48 | 0.0000 |
| ENSRNOG00000046007.2 | Cldn3 | 12:21831341-21832813 | 3.9E-08 | -2.45 | 0.0000 |
| ENSRNOG00000006305.5 | Slc38a2 | 7:138088648-138100841 | 4.0E-08 | 3.77 | 0.0000 |
| ENSRNOG00000049876.1 | Prkar1a | 10:97940704-97957336 | 4.0E-08 | -2.11 | 0.0000 |
| ENSRNOG00000032745.5 | Slc17a3 | 17:43518654-43543185 | 4.6E-08 | 2.03 | 0.0000 |
| ENSRNOG00000002342.6 | Aldh3a2 | 10:47525492-47546345 | 4.9E-08 | -2.05 | 0.0000 |
| ENSRNOG00000021380.5 | Fads6 | 10:103832837-103848035 | 5.2E-08 | -2.97 | 0.0000 |
| ENSRNOG00000019383.7 | Tef | 7:123043502-123058604 | 5.5E-08 | -4.92 | 0.0000 |
| ENSRNOG00000031641.4 | Rpl35a | 5:164923301-164923634 | 6.0E-08 | 35.78 | 0.0000 |
| ENSRNOG00000010298.6 | Xbp1 | 14:85753759-85758145 | 7.0E-08 | -2.31 | 0.0000 |
| ENSRNOG00000010887.6 | RGD1309534 | 8:13838571-13861212 | 7.1E-08 | -3.33 | 0.0000 |
| ENSRNOG00000047706.3 | LOC103690108 | 20:3791406-3794027 | 7.4E-08 | -2.94 | 0.0000 |
| ENSRNOG00000013439.7 | Cpn1 | 1:263733518-263762785 | 8.1E-08 | -2.00 | 0.0000 |
| ENSRNOG00000012443.5 | Cpt2 | 5:127505613-127523089 | 8.3E-08 | -2.37 | 0.0000 |
| ENSRNOG00000007971.6 | Wbp2 | 10:104630572-104637823 | 8.9E-08 | -2.60 | 0.0000 |
| ENSRNOG00000011559.7 | Cnn3 | 2:225005018-225036188 | 9.4E-08 | -2.27 | 0.0000 |
| ENSRNOG00000009957.7 | Slc25a13 | 4:31608268-31730386 | 1.1E-07 | -4.27 | 0.0000 |
| ENSRNOG00000036693.2 | Slc25a10 | 10:109665681-109673143 | 1.1E-07 | -2.08 | 0.0000 |
| ENSRNOG00000031979.3 | Mt-atp6 | MT:7918-8599 | 1.3E-07 | 2.98 | 0.0000 |
| ENSRNOG00000021924.4 | Cyp2c22 | 1:259257656-259287684 | 1.4E-07 | -2.38 | 0.0000 |
| ENSRNOG00000047611.2 | Top1 | 3:156642463-156717686 | 1.4E-07 | -6.10 | 0.0000 |
| ENSRNOG00000053518.1 | Zscan26 | 17:45188247-45207065 | 1.5E-07 | 3.50 | 0.0000 |
| ENSRNOG00000056457.1 | Gpd1 | 7:141370490-141377928 | 1.5E-07 | -2.22 | 0.0000 |
| ENSRNOG00000020194.7 | Hes6 | 9:98549704-98551438 | 1.6E-07 | -2.72 | 0.0000 |
| ENSRNOG00000005987.5 | Suox | 7:3098244-3102142 | 1.6E-07 | -2.04 | 0.0000 |
| ENSRNOG00000031766.3 | Mt-cyb | MT:14135-15278 | 1.8E-07 | 2.93 | 0.0000 |
| ENSRNOG00000019598.8 | Vegfa | 9:17340340-17355681 | 1.8E-07 | 2.14 | 0.0000 |
| ENSRNOG00000058183.1 | AABR07040624.1 | X:106607046-106607352 | 1.8E-07 | -2.92 | 0.0000 |
| ENSRNOG00000019946.5 | Gmppa | 9:82632229-82639821 | 1.8E-07 | -2.21 | 0.0000 |
| ENSRNOG00000042237.3 | Tm6sf2 | 16:21092307-21099878 | 2.0E-07 | -3.00 | 0.0000 |
| ENSRNOG00000056189.1 | AABR07044364.2 | 20:3306817-3307262 | 2.2E-07 | 13.38 | 0.0000 |
| ENSRNOG00000050360.2 | Urad | 12:9446939-9455673 | 2.3E-07 | -2.38 | 0.0000 |
| ENSRNOG00000047551.2 | LOC100911615 | 1:213997708-214002815 | 2.7E-07 | 24.15 | 0.0000 |
| ENSRNOG00000042261.3 | Hdgf | 2:187275119-187284281 | 2.7E-07 | -2.63 | 0.0000 |
| ENSRNOG00000017899.2 | Akr7a3 | 5:157801162-157813756 | 2.9E-07 | -2.09 | 0.0001 |
| ENSRNOG00000047023.2 | Aldh1l1 | 4:123516787-123557501 | 3.0E-07 | -3.29 | 0.0001 |
| ENSRNOG00000007492.7 | Rpn2 | 3:153398129-153445632 | 3.6E-07 | -2.12 | 0.0001 |
| ENSRNOG00000047714.2 | Tmem37 | 13:36094519-36101411 | 3.9E-07 | -2.45 | 0.0001 |
| ENSRNOG00000001415.6 | Ap1s1 | 12:22665111-22676075 | 4.0E-07 | -2.13 | 0.0001 |
| ENSRNOG00000012772.5 | Nqo1 | 19:38422163-38437180 | 4.3E-07 | -2.78 | 0.0001 |
| ENSRNOG00000006870.6 | Mtdh | 7:72772439-72827908 | 4.7E-07 | -4.41 | 0.0001 |
| ENSRNOG00000031273.3 | AABR07034639.1 | 11:82366073-82366505 | 5.0E-07 | 6.81 | 0.0001 |
| ENSRNOG00000031889.4 | AABR07065438.1 | 6:128738387-128739319 | 5.2E-07 | -2.16 | 0.0001 |
| ENSRNOG00000048989.3 | Larp7 | 2:231867134-231881939 | 5.3E-07 | -2.80 | 0.0001 |
| ENSRNOG00000032908.5 | Acaa1a | 8:128027957-128036236 | 5.4E-07 | -2.01 | 0.0001 |
| ENSRNOG00000003815.4 | Slc25a11 | 10:57265703-57268081 | 5.5E-07 | -2.21 | 0.0001 |
| ENSRNOG00000033609.4 | Irx1 | 1:33910911-33916741 | 5.7E-07 | -3.35 | 0.0001 |
| ENSRNOG00000016000.6 | Atp5pb | 2:208408889-208420163 | 5.9E-07 | -2.21 | 0.0001 |
| ENSRNOG00000054978.1 | H1f2 | 17:43639748-43640387 | 6.2E-07 | -3.47 | 0.0001 |
| ENSRNOG00000062158.1 | AC134224.3 | 1:221158097-221158792 | 6.2E-07 | -7.68 | 0.0001 |
| ENSRNOG00000013794.4 | Rbp1 | 8:106449320-106470842 | 6.8E-07 | -2.06 | 0.0001 |
| ENSRNOG00000031789.5 | Rangap1 | 7:122940375-122967178 | 7.0E-07 | -2.56 | 0.0001 |
| ENSRNOG00000057626.1 | Kif1b | 5:165938575-166133491 | 7.5E-07 | -3.24 | 0.0001 |
| ENSRNOG00000007545.6 | Angptl4 | 7:18627807-18634079 | 7.8E-07 | 2.05 | 0.0001 |
| ENSRNOG00000016459.6 | Eif3j | 3:113976686-113998922 | 7.9E-07 | -2.25 | 0.0001 |
| ENSRNOG00000030445.3 | Ormdl3 | 10:86547338-86554478 | 8.2E-07 | -3.72 | 0.0001 |
| ENSRNOG00000020578.8 | Ceacam1 | 1:82327954-82344345 | 8.7E-07 | 2.27 | 0.0001 |
| ENSRNOG00000011351.7 | Mat1a | 16:18690245-18709133 | 8.7E-07 | -2.01 | 0.0001 |
| ENSRNOG00000008689.8 | Cbx1 | 10:84669913-84675741 | 9.0E-07 | -2.97 | 0.0001 |
| ENSRNOG00000049057.1 | Znrf2 | 4:85009349-85090914 | 1.0E-06 | 2.85 | 0.0001 |
| ENSRNOG00000038045.4 | AC128960.1 | 17:78876110-78876537 | 1.0E-06 | -2.18 | 0.0001 |
| ENSRNOG00000013409.4 | Gclm | 2:225827503-225847874 | 1.0E-06 | -3.03 | 0.0001 |
| ENSRNOG00000032232.3 | Snrpg | 1:91588227-91588609 | 1.1E-06 | -5.48 | 0.0002 |
| ENSRNOG00000029512.1 | LOC100361854 | X:115495697-115496045 | 1.1E-06 | -2.24 | 0.0002 |
| ENSRNOG00000014051.4 | Pgrmc2 | 2:127986108-128002005 | 1.2E-06 | 2.00 | 0.0002 |
| ENSRNOG00000010725.3 | Cpa1 | 4:57952981-57959109 | 1.3E-06 | 6.97 | 0.0002 |
| ENSRNOG00000025602.4 | Cdk4 | 7:70349862-70352418 | 1.3E-06 | -2.42 | 0.0002 |
| ENSRNOG00000048878.1 | Smim24 | 7:11183569-11186130 | 1.4E-06 | -2.44 | 0.0002 |
| ENSRNOG00000054549.1 | Lss | 20:12842883-12870497 | 1.5E-06 | -2.26 | 0.0002 |
| ENSRNOG00000013133.3 | Foxa2 | 3:142383277-142387481 | 1.7E-06 | -2.49 | 0.0002 |
| ENSRNOG00000016265.7 | Acsl5 | 1:276240702-276290008 | 1.9E-06 | -2.33 | 0.0002 |
| ENSRNOG00000000165.8 | Pfkfb1 | X:23092158-23144324 | 1.9E-06 | -2.66 | 0.0003 |
| ENSRNOG00000019772.5 | Dnpep | 9:82505529-82514399 | 2.0E-06 | -2.00 | 0.0003 |
| ENSRNOG00000019283.5 | P2ry2 | 1:166031271-166037424 | 2.0E-06 | -3.45 | 0.0003 |
| ENSRNOG00000046299.2 | LOC100911417 | 2:208566384-208577146 | 2.1E-06 | -2.17 | 0.0003 |
| ENSRNOG00000000521.4 | Cdkn1a | 20:6351457-6358864 | 2.1E-06 | 6.71 | 0.0003 |
| ENSRNOG00000026965.6 | Tmem140 | 4:62380913-62391912 | 2.1E-06 | 2.01 | 0.0003 |
| ENSRNOG00000000172.7 | Sqor | 3:114900342-114939370 | 2.2E-06 | -2.76 | 0.0003 |
| ENSRNOG00000050183.4 | RT1-CE1 | 20:4638662-4943564 | 2.2E-06 | -4.10 | 0.0003 |
| ENSRNOG00000020029.5 | Mcrip2 | 10:15230800-15235740 | 2.3E-06 | -2.44 | 0.0003 |
| ENSRNOG00000001926.6 | Cldn1 | 11:77815180-77830416 | 2.3E-06 | 3.67 | 0.0003 |
| ENSRNOG00000034234.3 | Mt-co1 | MT:5322-6867 | 2.4E-06 | 2.82 | 0.0003 |
| ENSRNOG00000011573.6 | Csad | 7:143781701-143793970 | 2.9E-06 | -2.43 | 0.0004 |
| ENSRNOG00000013408.5 | Npas2 | 9:45901740-46081880 | 3.0E-06 | 18.75 | 0.0004 |
| ENSRNOG00000013982.3 | Hsd17b2 | 19:50246401-50317891 | 3.2E-06 | -3.22 | 0.0004 |
| ENSRNOG00000031090.6 | RT1-CE7 | 20:4694471-4896970 | 3.5E-06 | -2.61 | 0.0004 |
| ENSRNOG00000005424.4 | Odc1 | 6:42852682-42859927 | 3.5E-06 | -2.69 | 0.0004 |
| ENSRNOG00000014604.6 | Sigmar1 | 5:58121785-58124681 | 3.6E-06 | 2.27 | 0.0004 |
| ENSRNOG00000048834.2 | Plin3 | 9:10773900-10781109 | 3.6E-06 | -2.26 | 0.0004 |
| ENSRNOG00000033426.4 | Cdc37 | 8:22154066-22173804 | 3.9E-06 | -2.13 | 0.0004 |
| ENSRNOG00000017506.7 | Cltb | 17:10537364-10554989 | 4.1E-06 | -2.02 | 0.0005 |
| ENSRNOG00000019506.7 | Dnajb2 | 9:82436457-82444668 | 4.3E-06 | -2.09 | 0.0005 |
| ENSRNOG00000020440.6 | Fads2 | 1:226114412-226152524 | 4.5E-06 | -3.51 | 0.0005 |
| ENSRNOG00000013907.7 | Sall1 | 19:23389374-23405039 | 4.5E-06 | 2.48 | 0.0005 |
| ENSRNOG00000037627.4 | Trappc1 | 10:55924937-55926783 | 6.1E-06 | -2.01 | 0.0007 |
| ENSRNOG00000005130.7 | LOC103693780 | 14:86414948-86613327 | 6.2E-06 | -3.27 | 0.0007 |
| ENSRNOG00000020704.5 | Tkfc | 1:226644199-226657408 | 6.3E-06 | -2.46 | 0.0007 |
| ENSRNOG00000015304.5 | Tmem160 | 1:78417718-78420427 | 6.5E-06 | -2.19 | 0.0007 |
| ENSRNOG00000059572.1 | Tsen34l1 | 1:64092225-64099277 | 6.7E-06 | -2.17 | 0.0007 |
| ENSRNOG00000023480.6 | Nabp2 | 7:2820006-2825608 | 6.8E-06 | -2.02 | 0.0007 |
| ENSRNOG00000010178.6 | Cgrrf1 | 15:23619122-23639628 | 7.2E-06 | -2.10 | 0.0008 |
| ENSRNOG00000019710.6 | Tm9sf1 | 15:34314655-34322115 | 7.5E-06 | -2.73 | 0.0008 |
| ENSRNOG00000046094.2 | Cd151 | 1:214446658-214450666 | 7.5E-06 | -2.93 | 0.0008 |
| ENSRNOG00000053452.1 | LOC100361457 | 3:75643053-75644954 | 7.7E-06 | -2.62 | 0.0008 |
| ENSRNOG00000000967.5 | Aacs | 12:36512392-36555694 | 8.0E-06 | -2.48 | 0.0008 |
| ENSRNOG00000049491.2 | RT1-DMb | 20:3945600-3952846 | 8.2E-06 | -6.84 | 0.0008 |
| ENSRNOG00000013975.7 | Acat2l1 | 1:47996520-48026268 | 8.7E-06 | -2.43 | 0.0009 |
| ENSRNOG00000003269.3 | Atp6v0e1 | 10:16769665-16792909 | 8.7E-06 | -2.29 | 0.0009 |
| ENSRNOG00000058412.1 | Morf4l1 | 8:97474091-97494834 | 8.8E-06 | -2.41 | 0.0009 |
| ENSRNOG00000020715.7 | Ddb1 | 1:226657560-226683347 | 8.8E-06 | -2.26 | 0.0009 |
| ENSRNOG00000011621.7 | Hnrnpc | 15:28486616-28517353 | 9.2E-06 | -2.12 | 0.0009 |
| ENSRNOG00000008218.6 | Atp6v0e2 | 4:78168116-78171265 | 9.7E-06 | -5.52 | 0.0010 |
| ENSRNOG00000023356.5 | Eif5b | 9:44681493-44713199 | 1.1E-05 | -3.40 | 0.0011 |
| ENSRNOG00000015577.3 | Lpar6 | 15:55126952-55128761 | 1.1E-05 | 2.67 | 0.0011 |
| ENSRNOG00000047816.2 | Ccs | 1:220075246-220096404 | 1.1E-05 | -2.27 | 0.0011 |
| ENSRNOG00000051854.1 | Enpep | 2:233667253-233743866 | 1.4E-05 | 2.27 | 0.0013 |
| ENSRNOG00000059016.1 | Tspan12 | 4:48852826-48928372 | 1.4E-05 | 2.00 | 0.0013 |
| ENSRNOG00000048199.2 | Rps19l2 | 9:16802318-16807966 | 1.4E-05 | 3.92 | 0.0014 |
| ENSRNOG00000009163.7 | Fam133b | 4:27730050-27755103 | 1.5E-05 | -4.97 | 0.0014 |
| ENSRNOG00000014448.8 | Arntl | 1:178039062-178137465 | 1.5E-05 | 6.50 | 0.0015 |
| ENSRNOG00000014779.5 | Pdcd4 | 1:274625223-274648204 | 1.6E-05 | 2.28 | 0.0015 |
| ENSRNOG00000054962.1 | Dao | 12:48354195-48365784 | 1.6E-05 | -2.19 | 0.0015 |
| ENSRNOG00000012034.6 | Ces2i | 1:282567673-282575276 | 1.7E-05 | -2.83 | 0.0016 |
| ENSRNOG00000012053.7 | S100a16 | 2:189922995-189928848 | 1.7E-05 | -2.13 | 0.0016 |
| ENSRNOG00000031706.2 | RGD1563601 | 17:27765007-27765754 | 1.7E-05 | -3.91 | 0.0016 |
| ENSRNOG00000010558.7 | Ppif | 16:1979190-1985908 | 1.9E-05 | -2.01 | 0.0017 |
| ENSRNOG00000010580.8 | Acot7 | 5:169357516-169450219 | 2.1E-05 | -2.29 | 0.0019 |
| ENSRNOG00000004860.6 | Myh9 | 7:118741109-118792625 | 2.1E-05 | -3.94 | 0.0019 |
| ENSRNOG00000029543.4 | Cish | 8:116054464-116060723 | 2.1E-05 | -4.46 | 0.0019 |
| ENSRNOG00000012911.5 | Erlin1 | 1:263810438-263845762 | 2.1E-05 | -2.09 | 0.0019 |
| ENSRNOG00000015441.7 | Il4r | 1:196942363-196967220 | 2.1E-05 | -2.10 | 0.0019 |
| ENSRNOG00000062127.1 | AC134224.1 | 1:221154560-221156910 | 2.2E-05 | 2.27 | 0.0020 |
| ENSRNOG00000029886.3 | Hba-a1 | 10:15602793-15603649 | 2.3E-05 | -3.35 | 0.0020 |
| ENSRNOG00000011586.7 | LOC100909712 | 2:157747541-157759838 | 2.3E-05 | 2.26 | 0.0021 |
| ENSRNOG00000059579.1 | Gpt2 | 19:22590880-22632071 | 2.8E-05 | -2.81 | 0.0024 |
| ENSRNOG00000048932.2 | Smagp | 7:142244294-142260896 | 2.9E-05 | 2.20 | 0.0025 |
| ENSRNOG00000002839.6 | Slc19a2 | 13:82552549-82566586 | 2.9E-05 | 2.01 | 0.0025 |
| ENSRNOG00000056808.1 | AABR07031184.2 | 18:3617255-3617607 | 2.9E-05 | -7.51 | 0.0025 |
| ENSRNOG00000013090.7 | Gadd45g | 17:13391466-13393243 | 3.0E-05 | -2.80 | 0.0026 |
| ENSRNOG00000006019.4 | G0s2 | 13:112004139-112005052 | 3.1E-05 | 2.67 | 0.0026 |
| ENSRNOG00000014766.5 | Galt | 5:58144704-58147929 | 3.2E-05 | 2.06 | 0.0027 |
| ENSRNOG00000011276.7 | Tmem254 | 16:3817401-3821270 | 3.2E-05 | -2.16 | 0.0027 |
| ENSRNOG00000008757.6 | Tmem218 | 8:39687268-39702902 | 3.3E-05 | -2.67 | 0.0028 |
| ENSRNOG00000043377.4 | Fdps | 2:188392857-188413219 | 3.4E-05 | -2.09 | 0.0028 |
| ENSRNOG00000030391.4 | Ei24 | 8:39254888-39266959 | 3.6E-05 | 2.10 | 0.0030 |
| ENSRNOG00000018736.7 | Pnpla2 | 1:214434625-214439720 | 3.9E-05 | -2.49 | 0.0032 |
| ENSRNOG00000029574.2 | Rps4x | 4:35729627-35730599 | 4.1E-05 | -6.41 | 0.0033 |
| ENSRNOG00000014350.4 | Ccn1 | 2:251529353-251532312 | 4.2E-05 | 4.61 | 0.0034 |
| ENSRNOG00000014480.7 | Sys1 | 3:160908768-160912507 | 4.5E-05 | -2.05 | 0.0036 |
| ENSRNOG00000051170.2 | Ddx17 | 7:120761825-120780641 | 5.2E-05 | 2.94 | 0.0041 |
| ENSRNOG00000054176.1 | LOC103692171 | 4:114814055-114817465 | 5.3E-05 | -3.97 | 0.0041 |
| ENSRNOG00000029223.6 | Prss32 | 10:13196945-13202392 | 5.4E-05 | -3.77 | 0.0041 |
| ENSRNOG00000016269.7 | Rpl7l1 | 9:16508798-16513120 | 5.5E-05 | -2.76 | 0.0043 |
| ENSRNOG00000059069.1 | AABR07061378.1 | 4:120040771-120041238 | 5.7E-05 | -3.34 | 0.0043 |
| ENSRNOG00000013443.8 | Tm9sf3 | 1:260469042-260517323 | 5.9E-05 | -3.15 | 0.0045 |
| ENSRNOG00000058589.1 | AABR07046778.1 | 5:6373582-6373849 | 6.7E-05 | 7.37 | 0.0050 |
| ENSRNOG00000012404.6 | Thrsp | 1:162381252-162385575 | 7.0E-05 | -2.13 | 0.0052 |
| ENSRNOG00000014254.3 | Cpt1a | 1:218569509-218629678 | 7.0E-05 | -3.71 | 0.0052 |
| ENSRNOG00000003714.6 | Clk4 | 10:36715564-36733133 | 7.5E-05 | 2.29 | 0.0055 |
| ENSRNOG00000033680.5 | Cyp2b1 | 7:99142449-99181783 | 7.7E-05 | -27.58 | 0.0057 |
| ENSRNOG00000004342.7 | Ahr | 6:54963989-55001464 | 8.3E-05 | 2.85 | 0.0060 |
| ENSRNOG00000008830.7 | Nfe2l1 | 10:84682459-84698886 | 8.3E-05 | -3.40 | 0.0060 |
| ENSRNOG00000021513.1 | Rtn4rl2 | 3:72273423-72289310 | 8.3E-05 | -4.71 | 0.0060 |
| ENSRNOG00000007091.8 | Ly6e | 7:116355697-116359862 | 8.3E-05 | -2.23 | 0.0060 |
| ENSRNOG00000050630.2 | AABR07070043.1 | 8:45561389-45562574 | 8.4E-05 | -4.23 | 0.0061 |
| ENSRNOG00000006663.8 | Usp2 | 8:48406259-48430885 | 8.9E-05 | -6.84 | 0.0063 |
| ENSRNOG00000014204.7 | Pacsin3 | 3:80072488-80081047 | 9.6E-05 | -2.19 | 0.0068 |
| ENSRNOG00000004662.6 | AC115277.1 | 7:15385867-15386419 | 9.7E-05 | -7.04 | 0.0068 |
| ENSRNOG00000057116.1 | LOC102548286 | 13:90514335-90530349 | 1.1E-04 | -2.00 | 0.0075 |
| ENSRNOG00000011404.2 | Chkb | 7:130404831-130408187 | 1.1E-04 | 2.22 | 0.0076 |
| ENSRNOG00000018168.7 | Klc4 | 9:16647597-16661464 | 1.1E-04 | -2.17 | 0.0076 |
| ENSRNOG00000034093.4 | AABR07011951.1 | 2:177651240-177653288 | 1.2E-04 | -2.19 | 0.0080 |
| ENSRNOG00000020853.7 | Scap | 8:118570502-118628651 | 1.2E-04 | -2.05 | 0.0083 |
| ENSRNOG00000051238.1 | Mphosph8 | 15:36918842-36946708 | 1.2E-04 | -2.75 | 0.0083 |
| ENSRNOG00000013039.8 | Add1 | 14:81367467-81426496 | 1.3E-04 | -2.36 | 0.0086 |
| ENSRNOG00000012508.6 | Slc39a8 | 2:241029692-241092582 | 1.3E-04 | 2.17 | 0.0089 |
| ENSRNOG00000003649.9 | Qsox1 | 13:73423396-73460935 | 1.3E-04 | -3.00 | 0.0089 |
| ENSRNOG00000019661.3 | Gdf15 | 16:20555394-20557978 | 1.4E-04 | 2.94 | 0.0090 |
| ENSRNOG00000029668.4 | Wfdc21 | 10:71047309-71053191 | 1.4E-04 | -2.52 | 0.0090 |
| ENSRNOG00000058393.1 | Taf15 | 10:70689862-70721892 | 1.5E-04 | -2.16 | 0.0095 |
| ENSRNOG00000012017.4 | Otulin | 2:80269586-80293181 | 1.5E-04 | -2.09 | 0.0098 |
| ENSRNOG00000051831.1 | 5_8S_rRNA | 1:11906440-11906593 | 1.5E-04 | -4.10 | 0.0098 |
| ENSRNOG00000060723.1 | AABR07071244.1 | 8:106586688-106602837 | 1.5E-04 | -2.67 | 0.0100 |
| ENSRNOG00000010838.5 | Araf | X:1379193-1390873 | 1.5E-04 | -2.03 | 0.0100 |
| ENSRNOG00000019711.5 | Isoc1 | 18:53727208-53745200 | 1.6E-04 | -4.77 | 0.0101 |
| ENSRNOG00000015772.5 | RGD1560341 | 18:51218276-51220217 | 1.6E-04 | -7.40 | 0.0102 |
| ENSRNOG00000000219.7 | Ano10 | 8:130813213-130930990 | 1.6E-04 | -2.32 | 0.0103 |
| ENSRNOG00000030700.3 | Mt-co3 | MT:8598-9382 | 1.6E-04 | 2.19 | 0.0104 |
| ENSRNOG00000012266.4 | Zcchc17 | 5:148535518-148577292 | 1.7E-04 | -2.56 | 0.0105 |
| ENSRNOG00000020204.7 | Srp19 | 18:27114821-27121059 | 1.7E-04 | -2.15 | 0.0111 |
| ENSRNOG00000051784.1 | Apoc1 | KL568199.1:10856-11406 | 1.8E-04 | -3.43 | 0.0112 |
| ENSRNOG00000056028.1 | Rfxap | 2:144003453-144007636 | 1.8E-04 | -3.81 | 0.0113 |
| ENSRNOG00000022082.6 | LOC103690006 | 4:117800627-117814658 | 1.8E-04 | -4.28 | 0.0113 |
| ENSRNOG00000048613.2 | LOC100911130 | X:15441650-15448403 | 1.8E-04 | -2.04 | 0.0114 |
| ENSRNOG00000017404.6 | Pcmtd2 | 3:177351517-177371468 | 2.0E-04 | 2.88 | 0.0121 |
| ENSRNOG00000034139.4 | Lyc2 | 7:60316044-60323609 | 2.0E-04 | -2.22 | 0.0121 |
| ENSRNOG00000045539.2 | Dusp11 | 4:117617532-117631716 | 2.0E-04 | 2.38 | 0.0122 |
| ENSRNOG00000005861.6 | Hsd11b1 | 13:111926441-111972603 | 2.0E-04 | -2.36 | 0.0122 |
| ENSRNOG00000012650.5 | Mrpl3 | 8:113603532-113626893 | 2.0E-04 | -2.06 | 0.0122 |
| ENSRNOG00000036678.4 | Aspscr1 | 10:109851853-109888623 | 2.1E-04 | -2.08 | 0.0126 |
| ENSRNOG00000022296.4 | Pnpla5 | 7:124988523-124999137 | 2.1E-04 | -3.51 | 0.0129 |
| ENSRNOG00000000799.7 | Abcf1 | 20:3309898-3322825 | 2.2E-04 | -2.21 | 0.0136 |
| ENSRNOG00000021881.7 | Metap2 | 7:34762230-34789479 | 2.3E-04 | -2.07 | 0.0137 |
| ENSRNOG00000013973.5 | Lcn2 | 3:11414185-11417546 | 2.4E-04 | -3.63 | 0.0142 |
| ENSRNOG00000014508.7 | Mgll | 4:120671488-120771088 | 2.4E-04 | -4.15 | 0.0145 |
| ENSRNOG00000049900.1 | Irf2bp2 | 19:59499875-59502161 | 2.5E-04 | 2.49 | 0.0149 |
| ENSRNOG00000025156.6 | Timm17b | X:15340838-15347591 | 2.5E-04 | -2.00 | 0.0150 |
| ENSRNOG00000045738.2 | Ak4 | 5:120250615-120305897 | 2.6E-04 | -3.91 | 0.0151 |
| ENSRNOG00000004268.7 | Zfp386 | 6:143901279-143917575 | 2.6E-04 | 2.77 | 0.0154 |
| ENSRNOG00000025504.5 | Ankrd46 | 7:75279445-75288365 | 2.7E-04 | -3.13 | 0.0157 |
| ENSRNOG00000002217.6 | Plac8 | 14:10692763-10714524 | 2.8E-04 | -2.52 | 0.0160 |
| ENSRNOG00000005464.5 | Lgalsl | 14:105047918-105055421 | 2.8E-04 | 3.49 | 0.0160 |
| ENSRNOG00000046621.2 | AABR07043748.1 | 19:38340737-38344845 | 2.8E-04 | -2.38 | 0.0162 |
| ENSRNOG00000011871.7 | AABR07066180.1 | 9:4440981-4447715 | 3.1E-04 | 2.34 | 0.0177 |
| ENSRNOG00000043193.2 | Smim1 | 5:171309334-171312026 | 3.1E-04 | -2.07 | 0.0177 |
| ENSRNOG00000019000.8 | Limk2 | 14:83573927-83641892 | 3.2E-04 | -2.14 | 0.0182 |
| ENSRNOG00000056728.1 | G6pd | X:156274799-156293926 | 3.2E-04 | -2.66 | 0.0182 |
| ENSRNOG00000005930.5 | Nnmt | 8:52925174-52937972 | 3.2E-04 | -2.02 | 0.0183 |
| ENSRNOG00000056194.1 | LOC100912405 | 8:38545714-38549268 | 3.2E-04 | -12.21 | 0.0184 |
| ENSRNOG00000009715.6 | Me1 | 8:94256838-94368834 | 3.4E-04 | -2.62 | 0.0190 |
| ENSRNOG00000012237.6 | Rdx | 8:56585395-56610612 | 3.5E-04 | -2.66 | 0.0194 |
| ENSRNOG00000008586.8 | Aldh1l2 | 7:26375865-26425108 | 3.6E-04 | -2.20 | 0.0200 |
| ENSRNOG00000009446.4 | Rxra | 3:6211788-6295908 | 3.7E-04 | -2.70 | 0.0208 |
| ENSRNOG00000008519.7 | Dipk2a | 8:102088504-102159828 | 3.9E-04 | 3.46 | 0.0214 |
| ENSRNOG00000002828.7 | Tob1 | 10:81913688-81915716 | 3.9E-04 | 2.18 | 0.0215 |
| ENSRNOG00000048576.2 | LOC100911107 | 17:32066212-32076181 | 3.9E-04 | -6.55 | 0.0215 |
| ENSRNOG00000002871.6 | Rbm25l1 | 10:10457319-10460756 | 3.9E-04 | -2.35 | 0.0217 |
| ENSRNOG00000024964.7 | Nufip2 | 10:65176647-65200109 | 4.0E-04 | 7.46 | 0.0222 |
| ENSRNOG00000030345.4 | LOC100362684 | 9:1012090-1012450 | 4.0E-04 | -6.56 | 0.0222 |
| ENSRNOG00000028366.7 | Gphn | 6:101532517-101859164 | 4.2E-04 | 2.29 | 0.0230 |
| ENSRNOG00000028668.6 | Slc28a2 | 3:114355797-114647382 | 4.3E-04 | 2.07 | 0.0235 |
| ENSRNOG00000019915.5 | Xrcc1 | 1:81413352-81441678 | 4.6E-04 | -2.24 | 0.0244 |
| ENSRNOG00000003348.6 | Rasd1 | 10:46330367-46332172 | 4.6E-04 | 2.61 | 0.0247 |
| ENSRNOG00000032348.2 | LOC103690996 | 6:135139291-135139621 | 4.7E-04 | -22.32 | 0.0249 |
| ENSRNOG00000029698.5 | Pim3 | 7:129860113-129863441 | 4.9E-04 | 2.45 | 0.0257 |
| ENSRNOG00000058780.1 | Igfbp1 | 14:87448691-87453785 | 5.0E-04 | 2.53 | 0.0262 |
| ENSRNOG00000028822.4 | Bex3 | X:106823490-106825014 | 5.0E-04 | -2.69 | 0.0262 |
| ENSRNOG00000050214.2 | Amt | 8:117068581-117077913 | 5.4E-04 | -2.33 | 0.0277 |
| ENSRNOG00000058561.1 | Srrm2 | 10:13145098-13162343 | 5.4E-04 | 2.17 | 0.0277 |
| ENSRNOG00000009432.4 | Eif2s1 | 6:102048371-102073041 | 5.4E-04 | -2.11 | 0.0278 |
| ENSRNOG00000016150.5 | Ifrd2 | 8:116343095-116348314 | 5.6E-04 | -2.57 | 0.0288 |
| ENSRNOG00000007390.6 | Nfkbia | 6:76267227-76270457 | 5.7E-04 | 2.10 | 0.0289 |
| ENSRNOG00000037690.2 | Sertad3 | 1:84280944-84284663 | 5.8E-04 | 3.71 | 0.0293 |
| ENSRNOG00000045872.1 | Tmem125 | 5:137371824-137373205 | 6.1E-04 | -3.85 | 0.0307 |
| ENSRNOG00000012862.5 | Spsb4 | 8:104840939-104912959 | 6.8E-04 | -2.83 | 0.0337 |
| ENSRNOG00000020185.7 | Wdr6 | 8:117358786-117366096 | 6.9E-04 | -3.21 | 0.0341 |
| ENSRNOG00000007229.2 | Nr0b2 | 5:151776003-151779319 | 7.3E-04 | -2.75 | 0.0357 |
| ENSRNOG00000014178.5 | Acad9 | 2:122782059-122805768 | 7.5E-04 | -2.09 | 0.0369 |
| ENSRNOG00000036837.3 | Nfe2 | 7:144872746-144880092 | 7.5E-04 | -3.13 | 0.0369 |
| ENSRNOG00000055446.1 | Amfr | 19:11473540-11508102 | 7.6E-04 | -2.18 | 0.0370 |
| ENSRNOG00000002385.7 | Prg4 | 13:67672877-67688477 | 7.7E-04 | 2.02 | 0.0374 |
| ENSRNOG00000033979.3 | Gm5471 | 5:21043449-21043791 | 7.7E-04 | -10.31 | 0.0374 |
| ENSRNOG00000039249.3 | AABR07035539.1 | 12:19159467-19159901 | 7.7E-04 | -3.62 | 0.0374 |
| ENSRNOG00000015953.6 | Oaz2 | 8:71216177-71228713 | 7.8E-04 | -2.28 | 0.0376 |
| ENSRNOG00000037911.2 | LOC680227 | X:74304291-74345507 | 7.9E-04 | 5.60 | 0.0379 |
| ENSRNOG00000017901.6 | Acy3 | 1:219233749-219237098 | 8.3E-04 | -2.66 | 0.0397 |
| ENSRNOG00000029115.5 | LOC102555453 | 7:59762501-59763219 | 8.5E-04 | 2.17 | 0.0404 |
| ENSRNOG00000007315.7 | Thoc2 | X:128161894-128268322 | 8.6E-04 | -6.18 | 0.0409 |
| ENSRNOG00000017463.4 | Bloc1s3 | 1:80415685-80416273 | 8.6E-04 | -2.24 | 0.0410 |
| ENSRNOG00000030776.4 | Sytl2 | 1:154579948-154599430 | 8.9E-04 | 3.17 | 0.0420 |
| ENSRNOG00000008245.7 | AABR07054614.1 | 3:163950776-163986059 | 9.2E-04 | -2.82 | 0.0435 |
| ENSRNOG00000008243.7 | Slc43a1 | 3:72238980-72264466 | 9.4E-04 | -2.38 | 0.0441 |
| ENSRNOG00000019468.6 | Rce1 | 1:219860826-219863926 | 9.4E-04 | -2.34 | 0.0441 |
| ENSRNOG00000014050.6 | Ptges2 | 3:11424098-11431402 | 9.4E-04 | -2.28 | 0.0441 |
| ENSRNOG00000048164.2 | Acad8 | 8:28024122-28044876 | 1.1E-03 | -2.78 | 0.0498 |

*FDR: false discovery rate. The FDR-adjusted P value was sorted from lowest to highest.

Table S5. Statistics of differentially expressed genes (DEG) with *P* < 0.01, FDR-adjusted P value = 0.05 and |Fold change| > 2 in liver in comparison of CT vs. H60.

| Feature ID | Gene name | Position | P-value | Fold change | FDR-adjusted P value * |
| --- | --- | --- | --- | --- | --- |
| ENSRNOG00000000064.4 | Atp5me | 14:2325307-2326436 | 0.0000 | -3.43 | 0.0000 |
| ENSRNOG00000004196.7 | LOC108352650 | 6:91455332-91456696 | 0.0000 | -2.43 | 0.0000 |
| ENSRNOG00000007235.5 | Atp5mc1 | 10:83895946-83898527 | 0.0000 | -2.88 | 0.0000 |
| ENSRNOG00000026605.5 | Ifi27l2b | 6:127336304-127337791 | 0.0000 | -4.68 | 0.0000 |
| ENSRNOG00000028505.6 | Rps18 | 20:5441875-5445553 | 0.0000 | -2.97 | 0.0000 |
| ENSRNOG00000031022.5 | LOC108348287 | 18:1504177-1505570 | 0.0000 | -2.55 | 0.0000 |
| ENSRNOG00000033916.4 | AC141489.1 | 5:159606646-159606989 | 0.0000 | -3.04 | 0.0000 |
| ENSRNOG00000042886.1 | Rps28 | 7:18682070-18683440 | 0.0000 | -2.88 | 0.0000 |
| ENSRNOG00000050647.2 | Hspa1b | 20:4877323-4879779 | 3.53E-63 | 1281.27 | 1.05E-59 |
| ENSRNOG00000028690.3 | LOC100362366 | 3:24600602-24601063 | 6.55E-42 | -5.58 | 1.79E-38 |
| ENSRNOG00000037673.3 | AABR07071891.1 | 1:84573495-84573959 | 1.36E-39 | -5.81 | 3.44E-36 |
| ENSRNOG00000048199.2 | Rps19l2 | 9:16802318-16807966 | 1.7E-38 | 9.91 | 3.99E-35 |
| ENSRNOG00000033024.2 | AC099453.1 | 5:78392714-78393143 | 1.44E-35 | -11.59 | 3.14E-32 |
| ENSRNOG00000030538.5 | Slco1b2 | 4:175814117-175881768 | 2.33E-28 | 3.35 | 4.79E-25 |
| ENSRNOG00000026616.4 | Ndufb2 | 4:67378187-67385266 | 8.46E-26 | -2.52 | 1.63E-22 |
| ENSRNOG00000020704.5 | Tkfc | 1:226644199-226657408 | 1.53E-22 | -4.94 | 2.78E-19 |
| ENSRNOG00000049075.2 | Fabp5 | 2:93981655-93985378 | 7.05E-22 | -9.39 | 1.22E-18 |
| ENSRNOG00000034116.6 | Gk | X:54227396-54303864 | 7.11E-21 | 7.15 | 1.16E-17 |
| ENSRNOG00000000902.7 | Hsph1 | 12:6322667-6341902 | 1.3E-20 | 11.45 | 2.03E-17 |
| ENSRNOG00000031053.3 | Mt-nd4l | MT:9869-10166 | 1.62E-20 | 4.84 | 2.42E-17 |
| ENSRNOG00000020298.6 | Bag3 | 1:199941160-199965191 | 7.93E-20 | 13.18 | 1.13E-16 |
| ENSRNOG00000058646.1 | Zfp36l1 | 6:103308044-103313074 | 1.01E-19 | 4.60 | 1.38E-16 |
| ENSRNOG00000057823.1 | Ubc | 12:36638456-36642734 | 3.77E-19 | 3.53 | 4.95E-16 |
| ENSRNOG00000002693.2 | Nme1 | 10:81657152-81666523 | 4.57E-19 | -3.05 | 5.77E-16 |
| ENSRNOG00000007139.2 | Ttpa | 5:34007919-34028452 | 1.22E-18 | 2.86 | 1.48E-15 |
| ENSRNOG00000031889.4 | AABR07065438.1 | 6:128738387-128739319 | 1.89E-18 | -4.55 | 2.22E-15 |
| ENSRNOG00000012290.4 | Gchfr | 3:110975922-110979957 | 3.08E-18 | -2.73 | 3.48E-15 |
| ENSRNOG00000024967.5 | Uqcrb | 7:71264513-71269869 | 3.4E-18 | -2.52 | 3.72E-15 |
| ENSRNOG00000003597.5 | Tuba4a | 9:82415604-82419288 | 8.06E-18 | -3.13 | 8.55E-15 |
| ENSRNOG00000028616.6 | PCT1 | 3:171213935-171219871 | 8.64E-18 | 10.43 | 8.88E-15 |
| ENSRNOG00000005542.8 | Apob | 6:33176777-33224997 | 2.78E-17 | 2.62 | 2.77E-14 |
| ENSRNOG00000009364.7 | Ndufb9 | 7:98813039-98819452 | 6.35E-17 | -2.05 | 6.12E-14 |
| ENSRNOG00000032917.3 | Zfand2a | 12:17252092-17263477 | 6.52E-17 | 86.38 | 6.12E-14 |
| ENSRNOG00000016660.5 | Cox5b | 9:43259708-43262040 | 2.22E-16 | -2.39 | 2.02E-13 |
| ENSRNOG00000032232.3 | Snrpg | 1:91588227-91588609 | 4.01E-16 | -73.70 | 3.56E-13 |
| ENSRNOG00000011696.6 | Lifr | 2:56426366-56489415 | 4.31E-16 | 4.67 | 3.73E-13 |
| ENSRNOG00000010732.7 | RGD1561590 | 15:38069319-38073622 | 4.43E-16 | -3.07 | 3.73E-13 |
| ENSRNOG00000042903.1 | Cox7a2l2 | 8:87209529-87213627 | 8.88E-16 | -2.20 | 7.30E-13 |
| ENSRNOG00000011494.4 | Rpl36a | X:105402866-105405580 | 1E-15 | -2.87 | 8.02E-13 |
| ENSRNOG00000023546.5 | Hspb1 | 12:23839398-23841049 | 1.67E-15 | 15.02 | 1.30E-12 |
| ENSRNOG00000005698.2 | Ndufa5 | 4:51590412-51598771 | 3.76E-15 | -2.23 | 2.87E-12 |
| ENSRNOG00000010420.3 | Sem1 | 4:32067443-32087600 | 3.87E-15 | -2.30 | 2.89E-12 |
| ENSRNOG00000001420.8 | Fis1 | 12:22750484-22765308 | 4.26E-15 | -2.50 | 3.11E-12 |
| ENSRNOG00000005668.7 | Ndufa8 | 3:15362823-15379381 | 6.59E-15 | -2.88 | 4.71E-12 |
| ENSRNOG00000042503.2 | Ndufv2 | 9:113875702-113900190 | 7.09E-15 | -2.02 | 4.96E-12 |
| ENSRNOG00000016723.7 | Phpt1 | 3:2796996-2798470 | 1.13E-14 | -2.48 | 7.55E-12 |
| ENSRNOG00000021405.6 | Cyp2c7 | 1:148053686-148119857 | 1.11E-14 | 2.54 | 7.55E-12 |
| ENSRNOG00000006305.5 | Slc38a2 | 7:138088648-138100841 | 1.17E-14 | 5.66 | 7.71E-12 |
| ENSRNOG00000029512.1 | LOC100361854 | X:115495697-115496045 | 1.9E-14 | -3.59 | 1.20E-11 |
| ENSRNOG00000045654.2 | LOC108348108 | 20:2699711-2701815 | 1.87E-14 | 657.11 | 1.20E-11 |
| ENSRNOG00000022392.3 | Hspb8 | 12:45905370-45920013 | 2.01E-14 | 3.44 | 1.24E-11 |
| ENSRNOG00000062158.1 | AC134224.3 | 1:221158097-221158792 | 2.27E-14 | -26.57 | 1.38E-11 |
| ENSRNOG00000007336.7 | Churc1 | 6:99817430-99831576 | 6.05E-14 | -2.45 | 3.42E-11 |
| ENSRNOG00000045555.2 | LOC100910944 | 3:152222725-152224326 | 8.97E-14 | -2.41 | 5.00E-11 |
| ENSRNOG00000033803.5 | LOC100360841 | 9:69790830-69791196 | 9.4E-14 | -6.05 | 5.15E-11 |
| ENSRNOG00000021824.5 | Dnajb1 | 19:24747177-24750919 | 1.24E-13 | 12.42 | 6.69E-11 |
| ENSRNOG00000024577.6 | Gamt | 7:12314847-12317998 | 1.3E-13 | -2.19 | 6.87E-11 |
| ENSRNOG00000025909.5 | Uqcc2 | 20:5712202-5723902 | 1.49E-13 | -2.88 | 7.66E-11 |
| ENSRNOG00000003975.6 | Pfn1 | 10:57273004-57275708 | 1.9E-13 | -2.13 | 9.61E-11 |
| ENSRNOG00000003430.7 | F9 | X:143097524-143141794 | 2.34E-13 | 2.22 | 1.16E-10 |
| ENSRNOG00000042696.2 | Micos10 | 5:157546855-157573183 | 2.69E-13 | -2.17 | 1.32E-10 |
| ENSRNOG00000003977.5 | Dusp1 | 10:16970625-16973418 | 3.65E-13 | 17.48 | 1.76E-10 |
| ENSRNOG00000030747.2 | LOC685963 | 2:199479006-199479309 | 3.86E-13 | -2.62 | 1.84E-10 |
| ENSRNOG00000013011.7 | Dnajb4 | 2:257394817-257425242 | 4.18E-13 | 5.93 | 1.96E-10 |
| ENSRNOG00000054181.1 | LOC100361547 | 1:148240503-148287217 | 4.3E-13 | 3.88 | 1.99E-10 |
| ENSRNOG00000056728.1 | G6pd | X:156274799-156293926 | 5.27E-13 | -7.74 | 2.34E-10 |
| ENSRNOG00000060449.1 | Rps21 | 3:175629373-175630048 | 5.21E-13 | -2.61 | 2.34E-10 |
| ENSRNOG00000034066.5 | Hspa8 | 8:44990013-44993179 | 7.13E-13 | 8.77 | 3.12E-10 |
| ENSRNOG00000026293.2 | Jun | 5:114011188-114014277 | 1.52E-12 | 20.05 | 6.39E-10 |
| ENSRNOG00000045636.2 | Fasn | 10:109987734-110005901 | 1.84E-12 | -5.16 | 7.66E-10 |
| ENSRNOG00000023150.5 | Gpi | 1:90063410-90091287 | 2.24E-12 | -2.91 | 9.07E-10 |
| ENSRNOG00000014568.5 | Ndufb10 | 10:14090127-14092289 | 2.78E-12 | -2.19 | 1.11E-09 |
| ENSRNOG00000050655.1 | P4ha1 | 20:28920615-28971966 | 4.61E-12 | 6.28 | 1.78E-09 |
| ENSRNOG00000019578.7 | Rps16 | 1:85405511-85408444 | 6.98E-12 | -2.35 | 2.66E-09 |
| ENSRNOG00000020178.7 | Cope | 16:20863140-20873521 | 7.07E-12 | -2.11 | 2.67E-09 |
| ENSRNOG00000056457.1 | Gpd1 | 7:141370490-141377928 | 7.86E-12 | -2.74 | 2.93E-09 |
| ENSRNOG00000021438.3 | Tuba1c | 7:140716112-140723714 | 9.59E-12 | -2.42 | 3.50E-09 |
| ENSRNOG00000013133.3 | Foxa2 | 3:142383277-142387481 | 1.05E-11 | -4.26 | 3.80E-09 |
| ENSRNOG00000038045.4 | AC128960.1 | 17:78876110-78876537 | 1.24E-11 | -3.18 | 4.43E-09 |
| ENSRNOG00000006539.7 | Pex16 | 3:81283136-81292573 | 1.29E-11 | -2.59 | 4.54E-09 |
| ENSRNOG00000015774.5 | Mreg | 9:79490049-79545024 | 1.34E-11 | 3.53 | 4.69E-09 |
| ENSRNOG00000003724.7 | Mrpl27 | 10:82375571-82381332 | 1.53E-11 | -2.16 | 5.29E-09 |
| ENSRNOG00000047988.2 | Bola2 | 1:198120098-198121158 | 1.56E-11 | -2.21 | 5.33E-09 |
| ENSRNOG00000005615.6 | Gadd45a | 4:97782511-97784842 | 2.15E-11 | -3.57 | 7.21E-09 |
| ENSRNOG00000016166.4 | Pdlim1 | 1:259308295-259357056 | 2.14E-11 | -2.93 | 7.21E-09 |
| ENSRNOG00000008459.4 | Anapc13 | 8:111107357-111115774 | 2.41E-11 | -2.25 | 7.86E-09 |
| ENSRNOG00000055314.1 | Msrb1 | 10:14105749-14111463 | 2.39E-11 | -2.01 | 7.86E-09 |
| ENSRNOG00000015703.6 | Hypk | 3:113423692-113424846 | 2.8E-11 | -2.16 | 9.01E-09 |
| ENSRNOG00000042905.3 | RT1-T24-4 | 20:3189472-3197457 | 2.96E-11 | -2.71 | 9.44E-09 |
| ENSRNOG00000059714.1 | Hsp90aa1 | 6:135107270-135112775 | 3.65E-11 | 3.73 | 1.13E-08 |
| ENSRNOG00000059069.1 | AABR07061378.1 | 4:120040771-120041238 | 3.9E-11 | -5.28 | 1.19E-08 |
| ENSRNOG00000053086.1 | Selenop | 2:53109683-53114858 | 4.01E-11 | 3.63 | 1.22E-08 |
| ENSRNOG00000007029.3 | Dnaja1 | 5:57028466-57039378 | 4.07E-11 | 2.96 | 1.22E-08 |
| ENSRNOG00000039249.3 | AABR07035539.1 | 12:19159467-19159901 | 5.49E-11 | -17.11 | 1.62E-08 |
| ENSRNOG00000050315.2 | Dcxr | 10:109906814-109909715 | 5.72E-11 | -2.51 | 1.66E-08 |
| ENSRNOG00000024309.4 | Cox6b1 | 1:89075974-89084859 | 5.83E-11 | -2.45 | 1.68E-08 |
| ENSRNOG00000024845.5 | Mrps21 | 2:197974760-197982385 | 5.95E-11 | -2.37 | 1.70E-08 |
| ENSRNOG00000020029.5 | Mcrip2 | 10:15230800-15235740 | 6.95E-11 | -3.70 | 1.97E-08 |
| ENSRNOG00000003228.5 | Mid1ip1 | X:13114568-13116743 | 8.51E-11 | -3.91 | 2.39E-08 |
| ENSRNOG00000011189.5 | Acy1 | 8:115134764-115140080 | 9.05E-11 | -2.59 | 2.52E-08 |
| ENSRNOG00000056808.1 | AABR07031184.2 | 18:3617255-3617607 | 1.1E-10 | -108.87 | 3.01E-08 |
| ENSRNOG00000021035.6 | Rpl18 | 1:101701974-101703550 | 1.18E-10 | -2.05 | 3.21E-08 |
| ENSRNOG00000042821.3 | Cd59 | 3:94010474-94028621 | 1.23E-10 | 3.02 | 3.31E-08 |
| ENSRNOG00000000814.5 | Fabp7 | 20:40769585-40773349 | 1.26E-10 | 3.78 | 3.35E-08 |
| ENSRNOG00000015866.5 | Hint2 | 5:59150344-59152599 | 1.33E-10 | -2.10 | 3.48E-08 |
| ENSRNOG00000058183.1 | AABR07040624.1 | X:106607046-106607352 | 1.36E-10 | -4.12 | 3.55E-08 |
| ENSRNOG00000028382.3 | Rfxapl1 | 2:144213402-144217600 | 1.44E-10 | 56.19 | 3.72E-08 |
| ENSRNOG00000000473.6 | Pfdn6 | 20:5455973-5457444 | 1.56E-10 | -2.17 | 4.01E-08 |
| ENSRNOG00000019293.7 | AABR07006025.1 | 1:214015218-214024153 | 1.89E-10 | -2.65 | 4.83E-08 |
| ENSRNOG00000050997.1 | Ifrd1 | 6:60132023-60151420 | 2.23E-10 | 3.43 | 5.63E-08 |
| ENSRNOG00000008047.7 | Khk | 6:26810578-26820959 | 3.18E-10 | -2.32 | 7.86E-08 |
| ENSRNOG00000024568.6 | Ndufs7 | 7:12318756-12326392 | 3.39E-10 | -2.37 | 8.31E-08 |
| ENSRNOG00000009057.4 | Sec62 | 2:116189861-116219750 | 3.88E-10 | -2.58 | 9.44E-08 |
| ENSRNOG00000015591.5 | Cndp2 | 18:81521967-81539065 | 4.73E-10 | -2.61 | 1.14E-07 |
| ENSRNOG00000031263.3 | Haao | 6:7045151-7058314 | 5.48E-10 | -2.02 | 1.31E-07 |
| ENSRNOG00000043114.1 | Tomm7 | 4:7835948-7842789 | 5.86E-10 | -2.01 | 1.38E-07 |
| ENSRNOG00000014117.7 | Hmox1 | 19:14508615-14515456 | 8.05E-10 | 3.28 | 1.85E-07 |
| ENSRNOG00000021575.6 | Cfhr2 | 13:56642364-56693968 | 8.38E-10 | 2.89 | 1.90E-07 |
| ENSRNOG00000037518.3 | Hyi | 5:137189472-137192240 | 9.3E-10 | -2.30 | 2.09E-07 |
| ENSRNOG00000038001.3 | Slc25a1 | 11:87204174-87207264 | 9.35E-10 | -2.22 | 2.09E-07 |
| ENSRNOG00000031641.4 | Rpl35a | 5:164923301-164923634 | 9.82E-10 | 43.88 | 2.18E-07 |
| ENSRNOG00000049394.3 | LOC679739 | 1:32573227-32581781 | 1.06E-09 | -2.16 | 2.30E-07 |
| ENSRNOG00000018289.6 | Park7 | 5:167982438-168004724 | 1.21E-09 | -2.32 | 2.60E-07 |
| ENSRNOG00000020460.5 | Banf1 | 1:220744194-220746224 | 1.26E-09 | -2.06 | 2.68E-07 |
| ENSRNOG00000006898.5 | Mrps16 | 15:4351291-4353694 | 1.46E-09 | -2.10 | 3.06E-07 |
| ENSRNOG00000033611.5 | Wdr83os | 19:26194464-26195837 | 1.49E-09 | -2.06 | 3.09E-07 |
| ENSRNOG00000015455.6 | Spr | 4:116912350-116916236 | 1.55E-09 | -2.16 | 3.21E-07 |
| ENSRNOG00000028717.5 | Ndufb7 | 19:24701048-24705405 | 1.68E-09 | -2.01 | 3.41E-07 |
| ENSRNOG00000048282.2 | Mpnd | 9:11055370-11061611 | 1.87E-09 | -2.17 | 3.71E-07 |
| ENSRNOG00000029971.3 | Mt-nd5 | MT:11735-13565 | 1.91E-09 | 2.28 | 3.78E-07 |
| ENSRNOG00000057333.1 | AABR07030184.1 | 10:74765384-74765600 | 2.12E-09 | -91.19 | 4.15E-07 |
| ENSRNOG00000012106.4 | Dnaja4 | 8:59278261-59294003 | 2.37E-09 | 7.24 | 4.60E-07 |
| ENSRNOG00000022296.4 | Pnpla5 | 7:124988523-124999137 | 2.82E-09 | -8.61 | 5.42E-07 |
| ENSRNOG00000004575.8 | Il1a | 3:121825411-121836086 | 2.84E-09 | 9.63 | 5.43E-07 |
| ENSRNOG00000053210.1 | Zc3h11a | 13:50196041-50234862 | 2.98E-09 | 4.04 | 5.65E-07 |
| ENSRNOG00000020628.7 | LOC100361913 | 1:87190380-87191588 | 3.43E-09 | -4.92 | 6.48E-07 |
| ENSRNOG00000023387.5 | LOC100912599 | 1:31264754-31273159 | 3.71E-09 | -2.14 | 6.93E-07 |
| ENSRNOG00000042344.1 | Smim22 | 10:10766207-10767389 | 3.76E-09 | -3.35 | 6.97E-07 |
| ENSRNOG00000046316.1 | Tomm6 | 9:15313522-15314435 | 3.87E-09 | -2.04 | 7.11E-07 |
| ENSRNOG00000019428.7 | Higd1a | 8:130482492-130491998 | 4.21E-09 | -2.55 | 7.64E-07 |
| ENSRNOG00000055634.1 | AABR07024908.1 | 16:21807190-21807526 | 4.59E-09 | -3.04 | 8.30E-07 |
| ENSRNOG00000032630.5 | Mrps28 | 2:95045033-95173753 | 5.23E-09 | -2.30 | 9.35E-07 |
| ENSRNOG00000038012.2 | Commd6 | 15:86136095-86142672 | 5.64E-09 | -2.75 | 1.00E-06 |
| ENSRNOG00000000909.8 | Uspl1 | 12:6929977-6956984 | 6.02E-09 | 5.64 | 1.05E-06 |
| ENSRNOG00000048470.2 | LOC100911238 | 12:8746854-8759599 | 6.33E-09 | -4.05 | 1.10E-06 |
| ENSRNOG00000026965.6 | Tmem140 | 4:62380913-62391912 | 6.44E-09 | 2.20 | 1.12E-06 |
| ENSRNOG00000049814.2 | LOC100910882 | 3:152226709-152259156 | 6.49E-09 | 61.82 | 1.12E-06 |
| ENSRNOG00000058780.1 | Igfbp1 | 14:87448691-87453785 | 7.29E-09 | 3.43 | 1.25E-06 |
| ENSRNOG00000001182.7 | Ndufv3 | 20:10265805-10275304 | 7.59E-09 | -2.12 | 1.30E-06 |
| ENSRNOG00000016272.5 | Edf1 | 3:2781168-2785474 | 7.66E-09 | -2.25 | 1.30E-06 |
| ENSRNOG00000042869.2 | Atp5md | 10:89187166-89187474 | 7.81E-09 | -2.10 | 1.32E-06 |
| ENSRNOG00000020083.5 | Scly | 9:98438438-98459080 | 7.97E-09 | -2.17 | 1.34E-06 |
| ENSRNOG00000009565.4 | Pdk4 | 4:30546663-30556814 | 8.25E-09 | 4.20 | 1.37E-06 |
| ENSRNOG00000011952.6 | Samm50 | 7:125058896-125082505 | 8.22E-09 | -2.19 | 1.37E-06 |
| ENSRNOG00000056596.1 | Alas1 | 8:114927721-114940177 | 8.53E-09 | 2.06 | 1.41E-06 |
| ENSRNOG00000017851.5 | Etfb | 1:98472744-98486976 | 8.77E-09 | -2.01 | 1.43E-06 |
| ENSRNOG00000032303.2 | LOC108349682 | 1:219395275-219395623 | 8.88E-09 | -2.10 | 1.43E-06 |
| ENSRNOG00000052142.1 | Ahsa2 | 14:108265206-108273938 | 8.89E-09 | 3.35 | 1.43E-06 |
| ENSRNOG00000024964.7 | Nufip2 | 10:65176647-65200109 | 9.37E-09 | 14.51 | 1.51E-06 |
| ENSRNOG00000029861.5 | Gsta2 | 8:85640054-85645718 | 1.03E-08 | -2.86 | 1.65E-06 |
| ENSRNOG00000001142.3 | Prkab1 | 12:46316235-46326790 | 1.05E-08 | -2.51 | 1.67E-06 |
| ENSRNOG00000010408.7 | Polr2k | 7:74989222-74992582 | 1.09E-08 | -2.20 | 1.73E-06 |
| ENSRNOG00000015974.5 | Tmem208 | 19:37282017-37284718 | 1.11E-08 | -2.01 | 1.75E-06 |
| ENSRNOG00000019682.3 | Timm13 | 7:11677339-11678442 | 1.21E-08 | -2.10 | 1.90E-06 |
| ENSRNOG00000020990.2 | Fgf21 | 1:101595578-101596822 | 1.21E-08 | 11.77 | 1.90E-06 |
| ENSRNOG00000014288.8 | Fn1 | 9:78900102-78969078 | 1.32E-08 | 2.04 | 2.05E-06 |
| ENSRNOG00000047503.2 | Apoc3 | 8:50529317-50531498 | 1.33E-08 | -2.10 | 2.06E-06 |
| ENSRNOG00000002478.5 | Insig2 | 13:37266311-37287458 | 1.47E-08 | 3.51 | 2.26E-06 |
| ENSRNOG00000056135.1 | Tsc22d3 | X:111884294-111887906 | 1.65E-08 | 2.96 | 2.51E-06 |
| ENSRNOG00000047089.1 | Alkbh7 | 9:10045374-10047507 | 1.97E-08 | -3.03 | 2.97E-06 |
| ENSRNOG00000037627.4 | Trappc1 | 10:55924937-55926783 | 2.12E-08 | -2.46 | 3.16E-06 |
| ENSRNOG00000011250.6 | Inmt | 4:85381888-85386231 | 2.17E-08 | -18.72 | 3.23E-06 |
| ENSRNOG00000010706.4 | Ccdc117 | 14:85763396-85772762 | 2.33E-08 | 4.93 | 3.44E-06 |
| ENSRNOG00000019201.3 | Naxe | 2:187424326-187426375 | 2.87E-08 | -2.16 | 4.21E-06 |
| ENSRNOG00000015654.8 | Ghr | 2:53150369-53413638 | 2.93E-08 | 2.34 | 4.28E-06 |
| ENSRNOG00000047158.2 | Selenbp1 | 2:195416468-195423787 | 3.07E-08 | -2.02 | 4.46E-06 |
| ENSRNOG00000002520.5 | Litaf | 10:4719712-4763510 | 3.21E-08 | 3.80 | 4.64E-06 |
| ENSRNOG00000010224.6 | Rab30 | 1:157573323-157660008 | 3.22E-08 | 4.63 | 4.64E-06 |
| ENSRNOG00000012929.6 | Wsb1 | 10:66586923-66602987 | 3.45E-08 | 3.62 | 4.95E-06 |
| ENSRNOG00000015736.6 | Dhrs3 | 5:162808645-162843383 | 3.54E-08 | -2.30 | 5.06E-06 |
| ENSRNOG00000009538.6 | Etfdh | 2:178367548-178389608 | 3.61E-08 | 2.32 | 5.14E-06 |
| ENSRNOG00000018405.7 | Apoc4 | 1:80595325-80599572 | 3.92E-08 | -2.02 | 5.55E-06 |
| ENSRNOG00000002572.5 | Cacybp | 13:77948770-77959110 | 4.34E-08 | 2.42 | 6.10E-06 |
| ENSRNOG00000043193.2 | Smim1 | 5:171309334-171312026 | 4.34E-08 | -3.50 | 6.10E-06 |
| ENSRNOG00000012681.9 | Lgals9 | 10:64737021-64760201 | 4.54E-08 | -2.16 | 6.35E-06 |
| ENSRNOG00000051257.2 | AABR07039356.2 | X:74333328-74337975 | 4.75E-08 | 16.08 | 6.62E-06 |
| ENSRNOG00000053468.1 | Tuba1b | 7:140614751-140617721 | 5.57E-08 | -2.09 | 7.66E-06 |
| ENSRNOG00000008639.7 | Pabpc1 | 7:75409580-75422268 | 6.28E-08 | 2.14 | 8.43E-06 |
| ENSRNOG00000020836.7 | Rorc | 2:195617020-195637630 | 6.64E-08 | -2.58 | 8.87E-06 |
| ENSRNOG00000010240.6 | Tent5a | 8:92937738-92942076 | 7.04E-08 | 9.72 | 9.37E-06 |
| ENSRNOG00000051784.1 | Apoc1 | KL568199.1:10856-11406 | 7.43E-08 | -6.52 | 9.84E-06 |
| ENSRNOG00000000053.7 | Crp | 13:91054973-91093713 | 7.6E-08 | 2.16 | 1.00E-05 |
| ENSRNOG00000007227.7 | Mien1 | 10:86391847-86393141 | 7.7E-08 | -2.03 | 1.01E-05 |
| ENSRNOG00000016791.8 | Chka | 1:219077770-219126220 | 9.64E-08 | 3.99 | 1.25E-05 |
| ENSRNOG00000020420.6 | Pklr | 2:188449209-188459592 | 9.8E-08 | -3.56 | 1.26E-05 |
| ENSRNOG00000023360.4 | Fus | 1:199412833-199426702 | 9.82E-08 | -2.74 | 1.26E-05 |
| ENSRNOG00000015206.6 | Alad | 5:78368866-78379346 | 1.01E-07 | -2.17 | 1.30E-05 |
| ENSRNOG00000014350.4 | Ccn1 | 2:251529353-251532312 | 1.32E-07 | 10.56 | 1.65E-05 |
| ENSRNOG00000001415.6 | Ap1s1 | 12:22665111-22676075 | 1.34E-07 | -2.18 | 1.66E-05 |
| ENSRNOG00000029360.5 | Serinc1 | 20:38967237-38985036 | 1.46E-07 | 3.37 | 1.81E-05 |
| ENSRNOG00000037690.2 | Sertad3 | 1:84280944-84284663 | 1.49E-07 | 6.11 | 1.83E-05 |
| ENSRNOG00000004448.7 | Acss3 | 7:49047012-49250957 | 1.54E-07 | 2.30 | 1.89E-05 |
| ENSRNOG00000017693.7 | Slc2a5 | 5:167141874-167174310 | 1.63E-07 | -4.02 | 1.99E-05 |
| ENSRNOG00000036693.2 | Slc25a10 | 10:109665681-109673143 | 1.67E-07 | -2.44 | 2.03E-05 |
| ENSRNOG00000016099.2 | Id4 | 17:16692556-16695126 | 1.9E-07 | 2.58 | 2.30E-05 |
| ENSRNOG00000033195.6 | A1cf | 1:250426157-250514866 | 1.93E-07 | 6.01 | 2.33E-05 |
| ENSRNOG00000018796.5 | Herpud1 | 19:11046908-11057254 | 2.02E-07 | 2.57 | 2.40E-05 |
| ENSRNOG00000013907.7 | Sall1 | 19:23389374-23405039 | 2.06E-07 | 3.13 | 2.44E-05 |
| ENSRNOG00000016265.7 | Acsl5 | 1:276240702-276290008 | 2.11E-07 | -2.52 | 2.49E-05 |
| ENSRNOG00000016097.7 | Kyat1 | 3:8752624-8766433 | 2.22E-07 | -2.28 | 2.61E-05 |
| ENSRNOG00000000073.6 | Tmed5 | 14:2613405-2633950 | 2.24E-07 | 5.73 | 2.62E-05 |
| ENSRNOG00000046112.2 | Dedd2 | 1:82072543-82088275 | 2.28E-07 | 3.90 | 2.66E-05 |
| ENSRNOG00000049900.1 | Irf2bp2 | 19:59499875-59502161 | 2.39E-07 | 3.30 | 2.78E-05 |
| ENSRNOG00000015844.4 | Snrpd2 | 1:80056754-80059996 | 2.41E-07 | -2.01 | 2.79E-05 |
| ENSRNOG00000002828.7 | Tob1 | 10:81913688-81915716 | 2.44E-07 | 2.66 | 2.81E-05 |
| ENSRNOG00000003232.4 | Slc9a3r1 | 10:103713044-103730145 | 2.46E-07 | -2.22 | 2.82E-05 |
| ENSRNOG00000020624.4 | Acadsb | 1:201981356-202021008 | 2.55E-07 | 4.15 | 2.92E-05 |
| ENSRNOG00000000186.7 | Tst | 7:119616323-119623072 | 2.64E-07 | -2.12 | 3.01E-05 |
| ENSRNOG00000017105.7 | Dpyd | 2:221823686-222694627 | 2.81E-07 | 2.50 | 3.19E-05 |
| ENSRNOG00000006789.6 | Ddit3 | 7:70580197-70585084 | 2.83E-07 | 2.96 | 3.21E-05 |
| ENSRNOG00000009715.6 | Me1 | 8:94256838-94368834 | 2.95E-07 | -4.15 | 3.33E-05 |
| ENSRNOG00000018076.5 | Fmo5 | 2:199796880-199823927 | 3.05E-07 | 2.15 | 3.42E-05 |
| ENSRNOG00000038951.2 | Cox17 | 11:64962665-64968437 | 3.05E-07 | -2.03 | 3.42E-05 |
| ENSRNOG00000027089.6 | Ell2 | 2:2456734-2524237 | 3.21E-07 | 3.36 | 3.58E-05 |
| ENSRNOG00000062247.1 | AABR07031521.1 | 18:17116551-17118501 | 3.33E-07 | 3.65 | 3.71E-05 |
| ENSRNOG00000051615.1 | Hmgn2 | 5:152195360-152198813 | 3.43E-07 | -2.04 | 3.81E-05 |
| ENSRNOG00000020723.3 | Pten | 1:251421595-251487832 | 3.48E-07 | 2.63 | 3.84E-05 |
| ENSRNOG00000047816.2 | Ccs | 1:220075246-220096404 | 3.51E-07 | -2.52 | 3.85E-05 |
| ENSRNOG00000058484.1 | Anp32a | AABR07024206.1:1945-8593 | 3.8E-07 | -2.17 | 4.13E-05 |
| ENSRNOG00000014700.3 | Ttc36 | 8:49106373-49109981 | 4.06E-07 | -2.26 | 4.39E-05 |
| ENSRNOG00000017780.6 | Akr7a2 | 5:157759415-157768471 | 4.13E-07 | -2.05 | 4.44E-05 |
| ENSRNOG00000020676.3 | Ppp1r14a | 1:87224676-87232848 | 4.17E-07 | -3.23 | 4.45E-05 |
| ENSRNOG00000055079.1 | Ndufaf2 | 2:39321740-39434560 | 4.2E-07 | -2.99 | 4.47E-05 |
| ENSRNOG00000001189.6 | Sik1 | 20:10668410-10680283 | 4.49E-07 | 5.06 | 4.73E-05 |
| ENSRNOG00000019552.5 | Lsm7 | 7:11724961-11727388 | 4.7E-07 | -2.17 | 4.94E-05 |
| ENSRNOG00000003745.3 | Atf3 | 13:109817727-109849632 | 4.9E-07 | 36.69 | 5.12E-05 |
| ENSRNOG00000034093.4 | AABR07011951.1 | 2:177651240-177653288 | 5.02E-07 | 3.04 | 5.19E-05 |
| ENSRNOG00000014338.6 | Slc25a25 | 3:11442396-11452529 | 5.23E-07 | 2.92 | 5.39E-05 |
| ENSRNOG00000022268.6 | Pnpla3 | 7:125034763-125055976 | 5.73E-07 | -14.18 | 5.89E-05 |
| ENSRNOG00000007625.4 | B3galt1 | 3:54253948-54267292 | 5.97E-07 | 7.31 | 6.09E-05 |
| ENSRNOG00000019734.6 | Mrpl14 | 9:17687621-17698569 | 6.03E-07 | -2.61 | 6.14E-05 |
| ENSRNOG00000057855.1 | F5 | 13:82479997-82535534 | 6.5E-07 | 3.84 | 6.57E-05 |
| ENSRNOG00000030391.4 | Ei24 | 8:39254888-39266959 | 6.81E-07 | 2.17 | 6.86E-05 |
| ENSRNOG00000017899.2 | Akr7a3 | 5:157801162-157813756 | 7.47E-07 | -2.08 | 7.51E-05 |
| ENSRNOG00000010887.6 | RGD1309534 | 8:13838571-13861212 | 7.75E-07 | -3.15 | 7.76E-05 |
| ENSRNOG00000021079.4 | Fxyd1 | 1:89484198-89488223 | 8.05E-07 | -2.25 | 8.02E-05 |
| ENSRNOG00000012053.7 | S100a16 | 2:189922995-189928848 | 8.13E-07 | -2.43 | 8.04E-05 |
| ENSRNOG00000013963.7 | Il6st | 2:44289392-44314944 | 8.42E-07 | 4.75 | 8.31E-05 |
| ENSRNOG00000016348.4 | Tat | 19:41675354-41686229 | 8.58E-07 | 2.59 | 8.45E-05 |
| ENSRNOG00000002947.8 | Dpt | 13:83073543-83102401 | 9.02E-07 | -2.28 | 8.80E-05 |
| ENSRNOG00000031579.2 | LOC100363469 | 10:78992579-78993045 | 9.02E-07 | 2.23 | 8.80E-05 |
| ENSRNOG00000006331.4 | Elovl5 | 8:85259981-85285983 | 9.06E-07 | 3.25 | 8.81E-05 |
| ENSRNOG00000015967.5 | Sh3bgrl3 | 5:152357269-152358643 | 9.63E-07 | -2.42 | 9.31E-05 |
| ENSRNOG00000052604.1 | LOC100361907 | 13:56730396-56763981 | 1.02E-06 | 2.28 | 9.86E-05 |
| ENSRNOG00000051286.2 | LOC100911516 | 9:101106891-101107575 | 1.15E-06 | 3.04 | 0.0001 |
| ENSRNOG00000009592.6 | Cyb5r3 | 7:124023994-124041594 | 1.2E-06 | -2.40 | 0.0001 |
| ENSRNOG00000004006.5 | Dnajb9 | 6:64165912-64170151 | 1.32E-06 | 3.01 | 0.0001 |
| ENSRNOG00000050210.2 | RT1-CE10 | 20:154073-157861 | 1.34E-06 | -2.17 | 0.0001 |
| ENSRNOG00000029993.5 | Kynu | 3:28416953-28566928 | 1.44E-06 | 2.60 | 0.0001 |
| ENSRNOG00000004247.8 | Nhp2 | 10:34975707-34979082 | 1.51E-06 | -3.46 | 0.0001 |
| ENSRNOG00000012724.6 | Czib | 5:127489417-127497293 | 1.56E-06 | -2.31 | 0.0001 |
| ENSRNOG00000031769.3 | Chchd7 | 5:16845630-16847776 | 1.59E-06 | -2.64 | 0.0001 |
| ENSRNOG00000014387.4 | Chac1 | 3:111160204-111163425 | 1.64E-06 | 12.61 | 0.0002 |
| ENSRNOG00000053452.1 | LOC100361457 | 3:75643053-75644954 | 1.71E-06 | -2.84 | 0.0002 |
| ENSRNOG00000013004.7 | Akr1d1 | 4:65110745-65143941 | 1.78E-06 | 2.13 | 0.0002 |
| ENSRNOG00000004744.5 | Lratd2 | 7:101139375-101140308 | 1.88E-06 | 4.96 | 0.0002 |
| ENSRNOG00000036984.4 | Slco1a1 | 4:176158639-176231344 | 1.89E-06 | 2.37 | 0.0002 |
| ENSRNOG00000059593.1 | Cetn2 | X:152927717-152932953 | 1.93E-06 | -2.23 | 0.0002 |
| ENSRNOG00000058393.1 | Taf15 | 10:70689862-70721892 | 1.94E-06 | -2.72 | 0.0002 |
| ENSRNOG00000020763.8 | Snrnp70 | 1:101367470-101388129 | 2.04E-06 | -2.25 | 0.0002 |
| ENSRNOG00000001797.6 | Umps | 11:70034138-70044628 | 2.1E-06 | -2.14 | 0.0002 |
| ENSRNOG00000006990.6 | Grb7 | 10:86399826-86409348 | 2.13E-06 | 2.99 | 0.0002 |
| ENSRNOG00000007040.4 | Timm17a | 13:52124634-52136127 | 2.32E-06 | -2.31 | 0.0002 |
| ENSRNOG00000000461.7 | LOC108348112 | 20:5262119-5269337 | 2.38E-06 | 2.46 | 0.0002 |
| ENSRNOG00000042499.1 | LOC100364435 | 4:100882215-100883275 | 2.44E-06 | -2.01 | 0.0002 |
| ENSRNOG00000017120.6 | Abhd2 | 1:140998239-141087405 | 2.6E-06 | 3.57 | 0.0002 |
| ENSRNOG00000019442.5 | Josd2 | 1:100473642-100477351 | 2.6E-06 | -2.41 | 0.0002 |
| ENSRNOG00000017506.7 | Cltb | 17:10537364-10554989 | 2.68E-06 | -2.05 | 0.0002 |
| ENSRNOG00000029862.5 | Spc24 | 8:22780724-22785671 | 2.68E-06 | -5.33 | 0.0002 |
| ENSRNOG00000010658.6 | LOC103691744 | 2:264266983-264293046 | 2.72E-06 | 2.27 | 0.0002 |
| ENSRNOG00000007964.4 | Tp53inp1 | 5:24410862-24416888 | 3.07E-06 | 11.16 | 0.0003 |
| ENSRNOG00000056194.1 | LOC100912405 | 8:38545714-38549268 | 3.11E-06 | -29.15 | 0.0003 |
| ENSRNOG00000057601.1 | AABR07044080.2 | 19:56443631-56443869 | 3.44E-06 | -5.06 | 0.0003 |
| ENSRNOG00000031789.5 | Rangap1 | 7:122940375-122967178 | 3.5E-06 | -2.23 | 0.0003 |
| ENSRNOG00000047714.2 | Tmem37 | 13:36094519-36101411 | 3.6E-06 | -2.31 | 0.0003 |
| ENSRNOG00000050630.2 | AABR07070043.1 | 8:45561389-45562574 | 3.69E-06 | -5.79 | 0.0003 |
| ENSRNOG00000032825.2 | LOC100362027 | 10:38024476-38024937 | 3.71E-06 | -2.09 | 0.0003 |
| ENSRNOG00000004860.6 | Myh9 | 7:118741109-118792625 | 3.85E-06 | -4.56 | 0.0003 |
| ENSRNOG00000018107.5 | Zfand5 | 1:238843337-238852566 | 3.82E-06 | 2.46 | 0.0003 |
| ENSRNOG00000022082.6 | LOC103690006 | 4:117800627-117814658 | 3.84E-06 | -6.61 | 0.0003 |
| ENSRNOG00000058920.1 | Coa8 | 6:136279495-136304095 | 3.85E-06 | 32.14 | 0.0003 |
| ENSRNOG00000007390.6 | Nfkbia | 6:76267227-76270457 | 4.02E-06 | 2.58 | 0.0003 |
| ENSRNOG00000030715.6 | Cfh | 13:56978606-57080622 | 4.24E-06 | 2.05 | 0.0003 |
| ENSRNOG00000019684.5 | Antkmt | 10:15159802-15161938 | 4.28E-06 | -2.26 | 0.0003 |
| ENSRNOG00000018397.6 | Dnph1 | 9:16845524-16848503 | 4.53E-06 | -2.53 | 0.0004 |
| ENSRNOG00000008706.6 | Tbx3 | 12:42480559-42492526 | 4.57E-06 | 5.50 | 0.0004 |
| ENSRNOG00000006924.7 | Tmem125 | 5:137370576-137372993 | 4.79E-06 | -2.53 | 0.0004 |
| ENSRNOG00000007895.5 | Pdhb | 15:18539209-18546854 | 4.87E-06 | -2.05 | 0.0004 |
| ENSRNOG00000017744.7 | Dram2 | 2:209161046-209188622 | 4.98E-06 | 2.17 | 0.0004 |
| ENSRNOG00000020578.8 | Ceacam1 | 1:82327954-82344345 | 5.03E-06 | 2.20 | 0.0004 |
| ENSRNOG00000015218.7 | Nsmce1 | 1:196857460-196884302 | 5.4E-06 | -2.60 | 0.0004 |
| ENSRNOG00000018237.4 | Gstp1 | 1:219291678-219294147 | 5.86E-06 | -2.21 | 0.0004 |
| ENSRNOG00000017775.6 | Slc5a1 | 14:82909980-82975263 | 6.07E-06 | 18.64 | 0.0005 |
| ENSRNOG00000004206.5 | Glrx5 | 6:128750794-128760880 | 6.19E-06 | -2.00 | 0.0005 |
| ENSRNOG00000018176.7 | Rab6a | 1:165625057-165664192 | 6.37E-06 | 2.63 | 0.0005 |
| ENSRNOG00000018698.7 | Wac | 17:60888016-60946426 | 6.56E-06 | 4.60 | 0.0005 |
| ENSRNOG00000028668.6 | Slc28a2 | 3:114355797-114647382 | 6.67E-06 | 2.63 | 0.0005 |
| ENSRNOG00000007229.2 | Nr0b2 | 5:151776003-151779319 | 6.77E-06 | -3.21 | 0.0005 |
| ENSRNOG00000046621.2 | AABR07043748.1 | 19:38340737-38344845 | 7.26E-06 | -2.94 | 0.0005 |
| ENSRNOG00000005116.5 | LOC108353446 | 3:148283208-148284141 | 7.49E-06 | -2.05 | 0.0005 |
| ENSRNOG00000007817.5 | Kctd6 | 15:18484509-18493366 | 7.69E-06 | 2.82 | 0.0006 |
| ENSRNOG00000042237.3 | Tm6sf2 | 16:21092307-21099878 | 7.83E-06 | -2.39 | 0.0006 |
| ENSRNOG00000001391.6 | Sdsl | 12:41636993-41647205 | 7.99E-06 | -2.95 | 0.0006 |
| ENSRNOG00000005513.7 | Srsf5 | 6:104611025-104615302 | 8.09E-06 | -2.33 | 0.0006 |
| ENSRNOG00000047821.1 | Smlr1 | 1:20856186-20877716 | 8.08E-06 | -2.01 | 0.0006 |
| ENSRNOG00000009873.7 | Ints6 | 15:45858607-45947030 | 8.12E-06 | 5.07 | 0.0006 |
| ENSRNOG00000012980.5 | Hyls1 | 8:36763480-36764422 | 8.56E-06 | 7.35 | 0.0006 |
| ENSRNOG00000006019.4 | G0s2 | 13:112004139-112005052 | 9E-06 | 2.83 | 0.0006 |
| ENSRNOG00000021240.4 | Atrn | 3:123434408-123567918 | 8.98E-06 | 3.00 | 0.0006 |
| ENSRNOG00000004377.6 | Lpin1 | 6:41799748-41870046 | 9.34E-06 | 3.90 | 0.0007 |
| ENSRNOG00000006328.5 | Wwp1 | 5:33609135-33664435 | 9.38E-06 | 2.93 | 0.0007 |
| ENSRNOG00000012379.8 | Wdr18 | 7:12561323-12569143 | 9.35E-06 | -2.24 | 0.0007 |
| ENSRNOG00000016831.6 | Serpinh1 | 1:164301009-164308317 | 9.38E-06 | 2.14 | 0.0007 |
| ENSRNOG00000031033.4 | Mt-nd2 | MT:3903-4942 | 9.37E-06 | 2.15 | 0.0007 |
| ENSRNOG00000003472.8 | Atp11c | X:143346251-143453612 | 1.03E-05 | 4.27 | 0.0007 |
| ENSRNOG00000002280.6 | Sh3bgrl | X:80213331-80309936 | 1.05E-05 | 3.68 | 0.0007 |
| ENSRNOG00000008474.7 | Acox3 | 14:80314430-80355420 | 1.08E-05 | 2.39 | 0.0007 |
| ENSRNOG00000017866.5 | Sirt5 | 17:23986757-24013881 | 1.14E-05 | -2.25 | 0.0008 |
| ENSRNOG00000050305.2 | LOC100911854 | 1:248723396-248729962 | 1.17E-05 | 2.21 | 0.0008 |
| ENSRNOG00000007091.8 | Ly6e | 7:116355697-116359862 | 1.17E-05 | -2.39 | 0.0008 |
| ENSRNOG00000047746.1 | AABR07000398.1 | 1:11963835-11968939 | 1.24E-05 | 2.16 | 0.0008 |
| ENSRNOG00000017306.5 | AABR07071876.1 | 1:72624704-72625239 | 1.27E-05 | -2.03 | 0.0009 |
| ENSRNOG00000019982.6 | Ethe1 | 1:81456983-81472097 | 1.33E-05 | -2.24 | 0.0009 |
| ENSRNOG00000013975.7 | Acat2l1 | 1:47996520-48026268 | 1.34E-05 | -2.49 | 0.0009 |
| ENSRNOG00000046763.3 | Adssl1 | 6:137184819-137206693 | 1.46E-05 | -2.70 | 0.0010 |
| ENSRNOG00000020068.5 | Ndufaf3 | 8:117351628-117353672 | 1.49E-05 | -2.59 | 0.0010 |
| ENSRNOG00000045967.2 | AABR07064061.1 | 6:62796950-62798384 | 1.52E-05 | 4.84 | 0.0010 |
| ENSRNOG00000026643.5 | Chordc1 | 8:17421556-17446165 | 1.55E-05 | 2.53 | 0.0010 |
| ENSRNOG00000042714.2 | RGD1559459 | 14:22937420-22952822 | 1.57E-05 | 3.73 | 0.0010 |
| ENSRNOG00000020938.7 | Ppp1r15a | 1:101511900-101514974 | 1.58E-05 | 3.91 | 0.0010 |
| ENSRNOG00000050205.1 | Afmid | 10:106828980-106844314 | 1.62E-05 | -2.11 | 0.0011 |
| ENSRNOG00000000658.7 | Acacb | 12:48127148-48238887 | 1.63E-05 | -2.56 | 0.0011 |
| ENSRNOG00000014448.8 | Arntl | 1:178039062-178137465 | 1.63E-05 | 7.56 | 0.0011 |
| ENSRNOG00000008015.5 | Fos | 6:109300432-109303299 | 1.66E-05 | 29.99 | 0.0011 |
| ENSRNOG00000004662.6 | AC115277.1 | 7:15385867-15386419 | 1.69E-05 | -8.45 | 0.0011 |
| ENSRNOG00000001827.8 | Masp1 | 11:80736575-80803382 | 1.88E-05 | 3.43 | 0.0012 |
| ENSRNOG00000014597.5 | Irs1 | 9:88033667-88086488 | 1.96E-05 | 8.42 | 0.0013 |
| ENSRNOG00000018494.6 | Ppp1r3c | 1:255371809-255376833 | 2.01E-05 | 3.50 | 0.0013 |
| ENSRNOG00000042838.3 | Junb | 19:26092973-26094756 | 2.06E-05 | 4.16 | 0.0013 |
| ENSRNOG00000011857.7 | Mtpn | 4:63012010-63039422 | 2.07E-05 | 2.73 | 0.0013 |
| ENSRNOG00000015859.5 | Chdh | 16:6078121-6109382 | 2.13E-05 | -2.01 | 0.0013 |
| ENSRNOG00000032871.4 | Mlc1 | 7:129949966-129970550 | 2.31E-05 | -5.64 | 0.0014 |
| ENSRNOG00000000521.4 | Cdkn1a | 20:6351457-6358864 | 2.35E-05 | 9.40 | 0.0015 |
| ENSRNOG00000046950.2 | LOC100912481 | 4:24612204-24640686 | 2.44E-05 | -3.74 | 0.0015 |
| ENSRNOG00000009920.5 | LOC680121 | 11:13499163-13501263 | 2.49E-05 | 2.22 | 0.0015 |
| ENSRNOG00000009884.6 | Lgals1 | 7:120153183-120156289 | 2.5E-05 | -3.29 | 0.0015 |
| ENSRNOG00000011016.7 | Slc7a2 | 16:54460066-54513349 | 2.5E-05 | 3.18 | 0.0015 |
| ENSRNOG00000028100.4 | Pex11g | 12:2000426-2007516 | 2.62E-05 | -2.99 | 0.0016 |
| ENSRNOG00000010235.6 | Pkig | 3:160047295-160115177 | 2.8E-05 | -2.01 | 0.0017 |
| ENSRNOG00000046449.2 | Npy | 4:79573997-79581208 | 2.99E-05 | -5.06 | 0.0018 |
| ENSRNOG00000030345.4 | LOC100362684 | 9:1012090-1012450 | 3.17E-05 | -13.75 | 0.0019 |
| ENSRNOG00000020696.5 | Pmvk | 2:188784221-188793895 | 3.23E-05 | -2.06 | 0.0019 |
| ENSRNOG00000049132.2 | Map2k3 | 10:45089463-45110787 | 3.23E-05 | -2.48 | 0.0019 |
| ENSRNOG00000027990.4 | Crip1 | 6:137959170-137967042 | 3.25E-05 | -2.46 | 0.0019 |
| ENSRNOG00000011542.6 | Coa8 | 6:136185486-136210940 | 3.31E-05 | -2.06 | 0.0020 |
| ENSRNOG00000016629.7 | Tut7 | 17:5225834-5271794 | 3.35E-05 | 3.66 | 0.0020 |
| ENSRNOG00000055647.1 | Rbfa | 18:76704219-76714486 | 3.36E-05 | -2.13 | 0.0020 |
| ENSRNOG00000053932.1 | Ddx6 | 8:48925603-48958026 | 3.44E-05 | 6.23 | 0.0020 |
| ENSRNOG00000019481.5 | Cyp8b1 | 8:130548417-130550388 | 3.45E-05 | 2.06 | 0.0020 |
| ENSRNOG00000021924.4 | Cyp2c22 | 1:259257656-259287684 | 3.5E-05 | -2.57 | 0.0020 |
| ENSRNOG00000022054.2 | Paqr7 | 5:152708774-152720229 | 3.9E-05 | -2.34 | 0.0023 |
| ENSRNOG00000056670.1 | AABR07000902.1 | 1:27924824-27927782 | 3.98E-05 | -5.05 | 0.0023 |
| ENSRNOG00000021200.6 | Hjv | 2:198655436-198659317 | 4.08E-05 | -2.03 | 0.0023 |
| ENSRNOG00000046975.2 | Sult4a1 | 7:124958545-124982566 | 4.08E-05 | -10.57 | 0.0023 |
| ENSRNOG00000004342.7 | Ahr | 6:54963989-55001464 | 4.14E-05 | 2.78 | 0.0024 |
| ENSRNOG00000000967.5 | Aacs | 12:36512392-36555694 | 4.16E-05 | -2.49 | 0.0024 |
| ENSRNOG00000018755.6 | Acss2 | 3:151032951-151075856 | 4.17E-05 | -2.44 | 0.0024 |
| ENSRNOG00000046890.2 | Abca6 | 10:98478066-98544447 | 4.31E-05 | 2.61 | 0.0024 |
| ENSRNOG00000019215.6 | LOC100911730 | 1:214009783-214013765 | 4.34E-05 | -3.40 | 0.0024 |
| ENSRNOG00000036971.2 | Smim26 | 3:138765026-138766515 | 4.41E-05 | -2.02 | 0.0025 |
| ENSRNOG00000003463.8 | Srebf1 | 10:46570995-46593009 | 4.42E-05 | -2.46 | 0.0025 |
| ENSRNOG00000000201.7 | Gsta5 | 8:85553733-85565179 | 4.46E-05 | -2.17 | 0.0025 |
| ENSRNOG00000032690.3 | Ube2e2 | 15:7871496-7927939 | 4.46E-05 | -2.38 | 0.0025 |
| ENSRNOG00000001369.8 | Oas1a | 12:41200717-41211909 | 4.57E-05 | -4.17 | 0.0025 |
| ENSRNOG00000019996.5 | Slc16a1 | 2:207108551-207128554 | 4.76E-05 | 3.24 | 0.0026 |
| ENSRNOG00000057470.1 | Pla2g12a | 2:235311718-235327972 | 4.89E-05 | 3.07 | 0.0027 |
| ENSRNOG00000009117.5 | Otub2 | 6:127282465-127301083 | 4.9E-05 | -2.87 | 0.0027 |
| ENSRNOG00000017473.6 | Ttc25 | 10:88459489-88488237 | 4.98E-05 | -6.14 | 0.0027 |
| ENSRNOG00000054978.1 | H1f2 | 17:43639748-43640387 | 5.24E-05 | -2.53 | 0.0028 |
| ENSRNOG00000012659.5 | Vamp5 | 4:100231560-100232559 | 5.27E-05 | -2.51 | 0.0029 |
| ENSRNOG00000033680.5 | Cyp2b1 | 7:99142449-99181783 | 5.3E-05 | -23.93 | 0.0029 |
| ENSRNOG00000009284.6 | Foxa1 | 6:78545803-78549669 | 5.54E-05 | 4.97 | 0.0030 |
| ENSRNOG00000056076.1 | LOC103694877 | AABR07024106.1:11533-12195 | 5.6E-05 | -2.32 | 0.0030 |
| ENSRNOG00000046379.2 | Srm | 3:14588869-14591974 | 5.75E-05 | -3.96 | 0.0031 |
| ENSRNOG00000006420.6 | Rbm38 | 3:171037956-171050475 | 5.81E-05 | 3.27 | 0.0031 |
| ENSRNOG00000029698.5 | Pim3 | 7:129860113-129863441 | 5.86E-05 | 2.66 | 0.0031 |
| ENSRNOG00000006589.4 | Mif | 20:13732197-13732859 | 5.9E-05 | -2.34 | 0.0031 |
| ENSRNOG00000005918.5 | RGD1563941 | 8:52829084-52830609 | 6.02E-05 | -2.32 | 0.0032 |
| ENSRNOG00000049128.2 | Nktr | 8:130366774-130393178 | 6.02E-05 | 6.36 | 0.0032 |
| ENSRNOG00000050500.2 | Tob2 | 7:123079536-123088279 | 6.11E-05 | 5.68 | 0.0032 |
| ENSRNOG00000019485.6 | BCTdk | 1:199351627-199356881 | 6.47E-05 | -2.59 | 0.0034 |
| ENSRNOG00000049593.2 | Wbp11l1 | 4:170772162-170810080 | 6.66E-05 | -7.65 | 0.0035 |
| ENSRNOG00000049661.2 | Inafm1 | 1:78210866-78212350 | 6.86E-05 | -2.57 | 0.0035 |
| ENSRNOG00000010558.7 | Ppif | 16:1979190-1985908 | 7.26E-05 | -2.03 | 0.0037 |
| ENSRNOG00000054549.1 | Lss | 20:12842883-12870497 | 7.45E-05 | -2.42 | 0.0038 |
| ENSRNOG00000016924.8 | Acly | 10:88392247-88442845 | 7.48E-05 | -2.98 | 0.0038 |
| ENSRNOG00000001418.7 | Znhit1 | 12:22726642-22732943 | 7.59E-05 | -2.07 | 0.0039 |
| ENSRNOG00000060728.1 | Tuba1a | 7:140637286-140640953 | 7.65E-05 | -2.43 | 0.0039 |
| ENSRNOG00000038375.3 | AABR07026311.1 | 16:71438595-71439402 | 7.76E-05 | -4.15 | 0.0039 |
| ENSRNOG00000001647.6 | Ets2 | 11:36075708-36092495 | 7.91E-05 | 2.74 | 0.0040 |
| ENSRNOG00000051170.2 | Ddx17 | 7:120761825-120780641 | 7.99E-05 | 2.85 | 0.0040 |
| ENSRNOG00000033609.4 | Irx1 | 1:33910911-33916741 | 8.22E-05 | -2.39 | 0.0041 |
| ENSRNOG00000048989.3 | Larp7 | 2:231867134-231881939 | 8.51E-05 | -2.18 | 0.0043 |
| ENSRNOG00000004624.6 | Rnd3 | 3:36642586-36660758 | 8.54E-05 | 2.40 | 0.0043 |
| ENSRNOG00000009741.8 | Cyp4a3 | 5:134469269-134484839 | 8.56E-05 | 2.55 | 0.0043 |
| ENSRNOG00000048949.1 | LOC102549542 | 2:234375314-234483149 | 8.82E-05 | -3.31 | 0.0044 |
| ENSRNOG00000004821.6 | Sntb1 | 7:95397112-95669809 | 8.88E-05 | 7.16 | 0.0044 |
| ENSRNOG00000033169.5 | Cpeb4 | 10:15987920-16046033 | 8.88E-05 | 7.26 | 0.0044 |
| ENSRNOG00000003620.5 | Fmo3 | 13:80837419-80862963 | 8.94E-05 | 2.02 | 0.0044 |
| ENSRNOG00000043098.1 | Mt2A | 19:11307966-11308740 | 9.44E-05 | 4.88 | 0.0046 |
| ENSRNOG00000021084.7 | AABR07006310.1 | 1:228724899-228727160 | 9.55E-05 | 3.69 | 0.0047 |
| ENSRNOG00000011827.3 | LOC100360846 | 2:96031950-96032722 | 9.59E-05 | -3.08 | 0.0047 |
| ENSRNOG00000014090.3 | Retsat | 4:100465169-100474301 | 9.89E-05 | 2.75 | 0.0048 |
| ENSRNOG00000031421.5 | Eif1a | 18:40586314-40597671 | 1E-04 | 2.32 | 0.0049 |
| ENSRNOG00000001517.6 | Pdk1 | 3:58530869-58558027 | 0.0001 | -2.58 | 0.0049 |
| ENSRNOG00000059653.1 | Rpp25l | 5:58096212-58097577 | 0.0001 | -2.57 | 0.0049 |
| ENSRNOG00000020373.7 | Dap3 | 2:188225612-188252725 | 0.0001 | -2.21 | 0.0049 |
| ENSRNOG00000013498.4 | Pex14 | 5:165782894-165918445 | 0.0001 | -2.00 | 0.0049 |
| ENSRNOG00000048088.2 | Mest | 4:58053040-58063138 | 0.0001 | 7.01 | 0.0051 |
| ENSRNOG00000006779.6 | Crot | 4:22081603-22116265 | 0.0001 | 2.56 | 0.0053 |
| ENSRNOG00000012826.5 | Creb3l2 | 4:64869025-64981384 | 0.0001 | 4.84 | 0.0055 |
| ENSRNOG00000001285.8 | Atp2a2 | 12:39553902-39603326 | 0.0001 | 2.27 | 0.0056 |
| ENSRNOG00000005715.5 | Lgr4 | 3:101051954-101152119 | 0.0001 | 2.87 | 0.0057 |
| ENSRNOG00000013911.6 | Nagk | 4:115473810-115481438 | 0.0001 | -2.54 | 0.0057 |
| ENSRNOG00000048152.2 | Myo1b | 9:54558201-54766054 | 0.0001 | 2.63 | 0.0058 |
| ENSRNOG00000012772.5 | Nqo1 | 19:38422163-38437180 | 0.0001 | -2.40 | 0.0059 |
| ENSRNOG00000015222.5 | Tent5c | 2:202798197-202816562 | 0.0001 | 4.23 | 0.0059 |
| ENSRNOG00000027728.7 | Ibtk | 8:93324475-93390305 | 0.0001 | 2.80 | 0.0065 |
| ENSRNOG00000062125.1 | Aox3 | 9:65013861-65099433 | 0.0001 | 2.87 | 0.0066 |
| ENSRNOG00000060087.1 | Adra1b | 10:29392761-29450644 | 0.0001 | -2.06 | 0.0066 |
| ENSRNOG00000002832.5 | Slc16a2 | X:74577130-74706214 | 0.0001 | 2.49 | 0.0066 |
| ENSRNOG00000018829.6 | RGD1308134 | 10:56845382-56849255 | 0.0001 | -2.28 | 0.0066 |
| ENSRNOG00000008012.7 | Abcb4 | 4:22133520-22425515 | 0.0001 | 3.38 | 0.0066 |
| ENSRNOG00000001582.6 | Bach1 | 11:27364915-27398842 | 0.0001 | 8.88 | 0.0067 |
| ENSRNOG00000020775.8 | Cyp2b2 | 1:83103924-83119193 | 0.0001 | -2.41 | 0.0068 |
| ENSRNOG00000013552.6 | Scd | 1:264160128-264172729 | 0.0002 | -3.59 | 0.0068 |
| ENSRNOG00000032585.5 | AABR07048992.1 | 5:99031012-99033107 | 0.0002 | -2.45 | 0.0072 |
| ENSRNOG00000009994.5 | Dlat | 8:55062550-55087832 | 0.0002 | -2.54 | 0.0073 |
| ENSRNOG00000059255.1 | Rnu3a | 20:45357178-45357393 | 0.0002 | -33.12 | 0.0073 |
| ENSRNOG00000001738.8 | Eif4g1 | 11:83908108-83926524 | 0.0002 | 2.84 | 0.0073 |
| ENSRNOG00000020194.7 | Hes6 | 9:98549704-98551438 | 0.0002 | -4.03 | 0.0074 |
| ENSRNOG00000000420.7 | Nelfe | 20:4530341-4536209 | 0.0002 | -2.06 | 0.0076 |
| ENSRNOG00000045924.3 | RT1-T24-3 | 20:3226312-3233694 | 0.0002 | -3.35 | 0.0077 |
| ENSRNOG00000004287.6 | Wdr83 | 19:26188637-26194198 | 0.0002 | -2.44 | 0.0079 |
| ENSRNOG00000020506.6 | Khnyn | 15:34552409-34561727 | 0.0002 | 3.97 | 0.0079 |
| ENSRNOG00000055307.1 | AABR07058886.1 | 7:141666110-141676425 | 0.0002 | 4.98 | 0.0080 |
| ENSRNOG00000003291.6 | Creg1 | 13:83972211-83984675 | 0.0002 | 2.01 | 0.0084 |
| ENSRNOG00000009152.7 | Caprin1 | 3:93702486-93789617 | 0.0002 | 2.16 | 0.0084 |
| ENSRNOG00000004306.6 | Zbtb39 | 7:71036564-71044045 | 0.0002 | 4.48 | 0.0085 |
| ENSRNOG00000012736.5 | Znrd2 | 1:221087802-221089586 | 0.0002 | -2.32 | 0.0086 |
| ENSRNOG00000001516.7 | Rapgef4 | 3:58632475-58924038 | 0.0002 | 3.45 | 0.0090 |
| ENSRNOG00000010944.6 | Hyou1 | 8:48699768-48711910 | 0.0002 | 2.79 | 0.0094 |
| ENSRNOG00000055765.1 | Rasl10b | KL568414.1:23533-32066 | 0.0002 | -2.32 | 0.0094 |
| ENSRNOG00000033100.4 | AABR07054189.1 | 3:143828610-143829156 | 0.0002 | -4.53 | 0.0095 |
| ENSRNOG00000029956.3 | AABR07033023.1 | 11:1835184-1836897 | 0.0002 | 7.21 | 0.0095 |
| ENSRNOG00000051831.1 | 5_8S_rRNA | 1:11906440-11906593 | 0.0002 | -7.13 | 0.0096 |
| ENSRNOG00000011497.4 | Aldh1b1 | 5:61382350-61387358 | 0.0002 | -2.74 | 0.0097 |
| ENSRNOG00000009488.4 | Cyp7a1 | 5:19358733-19368431 | 0.0002 | -2.87 | 0.0102 |
| ENSRNOG00000011276.7 | Tmem254 | 16:3817401-3821270 | 0.0002 | -2.00 | 0.0102 |
| ENSRNOG00000007927.4 | Mettl7b | 7:3383878-3386522 | 0.0002 | -2.06 | 0.0104 |
| ENSRNOG00000020060.4 | Atf5 | 1:100808240-100810522 | 0.0003 | 2.21 | 0.0106 |
| ENSRNOG00000059017.1 | AC106292.2 | 11:65743891-65745038 | 0.0003 | 3.75 | 0.0107 |
| ENSRNOG00000000531.5 | Ccdc167 | 20:8369330-8384088 | 0.0003 | -2.44 | 0.0109 |
| ENSRNOG00000010643.7 | Kank2 | 8:22791994-22821397 | 0.0003 | -2.55 | 0.0110 |
| ENSRNOG00000000414.7 | Cep85l | 20:34575949-34684418 | 0.0003 | 11.81 | 0.0114 |
| ENSRNOG00000016119.6 | Fzd7 | 9:66305730-66308550 | 0.0003 | 8.91 | 0.0114 |
| ENSRNOG00000022934.6 | AABR07066818.1 | 9:17211758-17212628 | 0.0003 | -4.77 | 0.0115 |
| ENSRNOG00000005447.5 | Ypel2 | 10:74243811-74298599 | 0.0003 | 5.48 | 0.0115 |
| ENSRNOG00000008553.7 | Mthfr | 5:164845924-164860910 | 0.0003 | 5.56 | 0.0115 |
| ENSRNOG00000013090.7 | Gadd45g | 17:13391466-13393243 | 0.0003 | -2.81 | 0.0115 |
| ENSRNOG00000025406.6 | Iqgap2 | 2:26167198-26438790 | 0.0003 | 2.78 | 0.0116 |
| ENSRNOG00000029668.4 | Wfdc21 | 10:71047309-71053191 | 0.0003 | -2.56 | 0.0118 |
| ENSRNOG00000008209.6 | St3gal1 | 7:107895044-107963142 | 0.0003 | 2.52 | 0.0118 |
| ENSRNOG00000050181.2 | LOC100911881 | 1:214476386-214511529 | 0.0003 | -2.07 | 0.0119 |
| ENSRNOG00000008873.6 | Ino80b | 4:113954612-113957774 | 0.0003 | -2.41 | 0.0123 |
| ENSRNOG00000048291.2 | Tbcc | 9:16405184-16406338 | 0.0003 | -2.12 | 0.0125 |
| ENSRNOG00000008924.7 | Arhgef12 | 8:47259403-47393503 | 0.0003 | 4.15 | 0.0131 |
| ENSRNOG00000016246.5 | Tshz1 | 18:80862371-80865584 | 0.0003 | 4.39 | 0.0131 |
| ENSRNOG00000002609.7 | Ero1b | 17:90710554-90756048 | 0.0003 | 2.69 | 0.0132 |
| ENSRNOG00000003300.3 | Btg2 | 13:50913179-50916982 | 0.0003 | 3.40 | 0.0132 |
| ENSRNOG00000046560.1 | AC109096.1 | 1:220836091-220836504 | 0.0003 | -3.32 | 0.0132 |
| ENSRNOG00000001441.7 | Tmem120a | 12:23989595-23998254 | 0.0003 | -2.07 | 0.0136 |
| ENSRNOG00000006108.5 | Gngt2 | 10:83655459-83658589 | 0.0003 | -2.23 | 0.0137 |
| ENSRNOG00000020246.7 | Myl9 | 3:152857591-152863960 | 0.0004 | -2.04 | 0.0138 |
| ENSRNOG00000015195.6 | Hdhd3 | 5:78358202-78361647 | 0.0004 | -2.72 | 0.0141 |
| ENSRNOG00000048932.2 | Smagp | 7:142244294-142260896 | 0.0004 | 2.04 | 0.0143 |
| ENSRNOG00000023271.7 | Parl | 11:84517367-84544462 | 0.0004 | -2.12 | 0.0144 |
| ENSRNOG00000046643.2 | Cyp3a9 | 12:19074582-19114399 | 0.0004 | 2.47 | 0.0144 |
| ENSRNOG00000009084.6 | Rpusd3 | 4:145366764-145370992 | 0.0004 | -2.27 | 0.0144 |
| ENSRNOG00000001989.6 | Alcam | 11:50781126-50985083 | 0.0004 | 2.16 | 0.0145 |
| ENSRNOG00000001607.7 | Adamts1 | 11:25342125-25350974 | 0.0004 | 4.16 | 0.0147 |
| ENSRNOG00000010775.7 | Arrdc4 | 1:129766099-129780356 | 0.0004 | 3.51 | 0.0150 |
| ENSRNOG00000012314.4 | Slirp | 6:111476767-111486039 | 0.0004 | -2.49 | 0.0151 |
| ENSRNOG00000016298.7 | Lysmd3 | 2:9526208-9534362 | 0.0004 | 3.53 | 0.0152 |
| ENSRNOG00000018886.5 | Prxl2c | 17:1798387-1830001 | 0.0004 | 3.38 | 0.0153 |
| ENSRNOG00000018294.6 | Hspa5 | 3:13838303-13842762 | 0.0004 | 2.08 | 0.0159 |
| ENSRNOG00000008487.7 | Amotl2 | 8:111209907-111226226 | 0.0004 | 3.65 | 0.0161 |
| ENSRNOG00000024213.6 | Golim4 | 2:174322672-174413236 | 0.0004 | 3.41 | 0.0165 |
| ENSRNOG00000017463.4 | Bloc1s3 | 1:80415685-80416273 | 0.0004 | -2.31 | 0.0167 |
| ENSRNOG00000030418.3 | Tmem47 | X:48779109-48805644 | 0.0005 | 2.41 | 0.0172 |
| ENSRNOG00000057626.1 | Kif1b | 5:165938575-166133491 | 0.0005 | -2.25 | 0.0172 |
| ENSRNOG00000013954.5 | Alpl | 5:156086496-156141537 | 0.0005 | -5.03 | 0.0183 |
| ENSRNOG00000033522.4 | Prdm2 | 5:161770652-161879133 | 0.0005 | 5.02 | 0.0183 |
| ENSRNOG00000003714.6 | Clk4 | 10:36715564-36733133 | 0.0005 | 2.08 | 0.0183 |
| ENSRNOG00000058083.1 | Metazoa_SRP | 6:91680228-91680528 | 0.0005 | -7.02 | 0.0184 |
| ENSRNOG00000002116.6 | Rplp2 | 1:213991619-213993609 | 0.0005 | -2.10 | 0.0184 |
| ENSRNOG00000012274.8 | Ddi2 | 5:160230954-160282810 | 0.0005 | 2.02 | 0.0185 |
| ENSRNOG00000058752.1 | Popdc2 | 11:64936691-64952687 | 0.0005 | 5.70 | 0.0185 |
| ENSRNOG00000019407.5 | Dmap1 | 5:136533791-136541795 | 0.0005 | -2.35 | 0.0185 |
| ENSRNOG00000057056.1 | Rab5a | 14:72889037-72949307 | 0.0005 | 2.20 | 0.0189 |
| ENSRNOG00000013376.6 | Mvd | 19:55258904-55268951 | 0.0005 | -2.13 | 0.0190 |
| ENSRNOG00000051171.2 | G6pc | 10:89285854-89296213 | 0.0005 | 2.53 | 0.0191 |
| ENSRNOG00000003924.6 | Pi4k2b | 14:60567966-60594844 | 0.0005 | 2.46 | 0.0191 |
| ENSRNOG00000029223.6 | Prss32 | 10:13196945-13202392 | 0.0005 | -3.05 | 0.0193 |
| ENSRNOG00000014478.7 | Fndc3a | 15:54381043-54528480 | 0.0005 | 3.96 | 0.0193 |
| ENSRNOG00000009639.5 | Zrsr1 | 14:107750161-107751892 | 0.0006 | 4.53 | 0.0200 |
| ENSRNOG00000009068.5 | Phlda3 | 13:52588916-52592001 | 0.0006 | -2.26 | 0.0201 |
| ENSRNOG00000018484.5 | Plk3 | 5:135997724-136002900 | 0.0006 | 4.62 | 0.0202 |
| ENSRNOG00000008331.7 | Washc4 | 7:26311093-26361221 | 0.0006 | 3.57 | 0.0203 |
| ENSRNOG00000002871.6 | Rbm25l1 | 10:10457319-10460756 | 0.0006 | -2.27 | 0.0213 |
| ENSRNOG00000005389.5 | Ppp2ca | 10:37535870-37554664 | 0.0006 | 2.25 | 0.0214 |
| ENSRNOG00000049236.2 | Myh9l1 | 7:118992355-119071712 | 0.0006 | 3.01 | 0.0215 |
| ENSRNOG00000051850.1 | Scarna2 | 2:211344016-211344432 | 0.0006 | -3.62 | 0.0218 |
| ENSRNOG00000011081.7 | Serpina7 | X:110226571-110232179 | 0.0006 | 2.25 | 0.0223 |
| ENSRNOG00000022615.5 | Tp53i13 | 10:62650718-62655362 | 0.0007 | -2.32 | 0.0245 |
| ENSRNOG00000020799.6 | LOC103690054 | 1:88686730-88688258 | 0.0007 | -2.01 | 0.0247 |
| ENSRNOG00000037087.1 | LOC108351584 | 7:127081425-127081704 | 0.0007 | -4.01 | 0.0253 |
| ENSRNOG00000002936.7 | Golt1a | 13:50537132-50550015 | 0.0007 | -2.17 | 0.0254 |
| ENSRNOG00000003679.6 | Med13 | 10:73702326-73787083 | 0.0007 | 7.71 | 0.0255 |
| ENSRNOG00000060337.1 | Exosc7 | 8:132204603-132229969 | 0.0008 | -2.07 | 0.0267 |
| ENSRNOG00000047247.3 | Ptprs | 9:10603812-10645939 | 0.0008 | 2.83 | 0.0274 |
| ENSRNOG00000056462.1 | Tmf1 | 4:129521990-129544506 | 0.0008 | 2.92 | 0.0274 |
| ENSRNOG00000017850.4 | Dctpp1 | 1:198703460-198706852 | 0.0008 | -2.15 | 0.0278 |
| ENSRNOG00000045539.2 | Dusp11 | 4:117617532-117631716 | 0.0008 | 2.23 | 0.0280 |
| ENSRNOG00000023465.4 | Depp1 | 4:148782478-148784562 | 0.0008 | -2.60 | 0.0283 |
| ENSRNOG00000007587.6 | Tcp11l2 | 7:24859049-24885775 | 0.0008 | 2.53 | 0.0286 |
| ENSRNOG00000024849.5 | Tor1aip2 | 13:73708814-73735339 | 0.0008 | 2.03 | 0.0288 |
| ENSRNOG00000033372.5 | Klhl24 | 11:84615759-84633504 | 0.0009 | 3.40 | 0.0290 |
| ENSRNOG00000004626.5 | Slc34a2 | 14:60256711-60276794 | 0.0009 | 3.63 | 0.0292 |
| ENSRNOG00000006718.6 | Rbm33 | 4:594881-695383 | 0.0009 | 5.46 | 0.0292 |
| ENSRNOG00000031802.4 | LOC691427 | 1:141218094-141218277 | 0.0009 | -2.95 | 0.0299 |
| ENSRNOG00000007607.4 | Nr4a1 | 7:142905757-142920216 | 0.0009 | 11.73 | 0.0302 |
| ENSRNOG00000057806.1 | Trpm7 | 3:119259466-119330345 | 0.0009 | 4.77 | 0.0308 |
| ENSRNOG00000049324.2 | Tspan4 | 1:214016896-214038723 | 0.0009 | -3.95 | 0.0308 |
| ENSRNOG00000019383.7 | Tef | 7:123043502-123058604 | 0.0009 | -2.49 | 0.0313 |
| ENSRNOG00000061813.1 | Ly6c | KL567988.1:20092-23908 | 0.0009 | -3.80 | 0.0313 |
| ENSRNOG00000050183.4 | RT1-CE1 | 20:4638662-4943564 | 0.0010 | -2.35 | 0.0316 |
| ENSRNOG00000013265.6 | Tgfbr2 | 8:124312753-124399494 | 0.0010 | 2.86 | 0.0319 |
| ENSRNOG00000045872.1 | Tmem125 | 5:137371824-137373205 | 0.0010 | -3.44 | 0.0319 |
| ENSRNOG00000029341.4 | AABR07036375.1 | 12:40685889-40686249 | 0.0010 | -10.54 | 0.0323 |
| ENSRNOG00000030374.5 | LOC100360750 | 13:47380405-47382955 | 0.0010 | -2.04 | 0.0327 |
| ENSRNOG00000021010.5 | Arl2 | 1:221504169-221516110 | 0.0010 | -2.14 | 0.0330 |
| ENSRNOG00000016704.6 | Pcyox1 | 4:118187133-118198029 | 0.0010 | 3.46 | 0.0335 |
| ENSRNOG00000030154.5 | Cyp4a2 | 5:134196911-134207863 | 0.0010 | 2.95 | 0.0338 |
| ENSRNOG00000019426.6 | Ramac | 1:143583674-143589556 | 0.0011 | -2.05 | 0.0348 |
| ENSRNOG00000004649.4 | Il1b | 3:121876262-121882726 | 0.0011 | 3.30 | 0.0358 |
| ENSRNOG00000017645.6 | Mylpf | 1:198655741-198658583 | 0.0011 | -24.46 | 0.0365 |
| ENSRNOG00000007849.6 | Zmat5 | 14:84953785-84969116 | 0.0011 | -2.08 | 0.0367 |
| ENSRNOG00000060063.1 | Naa10 | X:156863753-156868950 | 0.0012 | -2.06 | 0.0371 |
| ENSRNOG00000034161.4 | Cox6b1 | 2:211337270-211337531 | 0.0012 | -2.17 | 0.0374 |
| ENSRNOG00000004563.7 | Sec24a | 10:37139805-37215899 | 0.0012 | 3.14 | 0.0386 |
| ENSRNOG00000017786.6 | Acta1 | 19:56674073-56677084 | 0.0012 | -36.38 | 0.0386 |
| ENSRNOG00000058439.1 | LOC100910130 | X:156392629-156399760 | 0.0012 | -2.44 | 0.0387 |
| ENSRNOG00000005917.5 | Pawr | 7:51273770-51353068 | 0.0012 | 2.39 | 0.0393 |
| ENSRNOG00000002128.3 | Ppat | 14:33580540-33614919 | 0.0012 | 2.43 | 0.0395 |
| ENSRNOG00000023356.5 | Eif5b | 9:44681493-44713199 | 0.0013 | -2.25 | 0.0397 |
| ENSRNOG00000002991.7 | Srr | 10:61756137-61772293 | 0.0013 | 2.14 | 0.0399 |
| ENSRNOG00000013328.8 | Rbpms | 16:61954589-62096497 | 0.0013 | 2.07 | 0.0400 |
| ENSRNOG00000008757.6 | Tmem218 | 8:39687268-39702902 | 0.0013 | -2.03 | 0.0400 |
| ENSRNOG00000053891.1 | AABR07018078.1 | 15:38647778-38709984 | 0.0013 | -2.05 | 0.0400 |
| ENSRNOG00000020185.7 | Wdr6 | 8:117358786-117366096 | 0.0013 | -2.75 | 0.0409 |
| ENSRNOG00000009145.7 | Klf12 | 15:84324469-84748525 | 0.0013 | 6.55 | 0.0413 |
| ENSRNOG00000018903.8 | Pik3r1 | 2:31745087-31826867 | 0.0013 | 2.34 | 0.0413 |
| ENSRNOG00000024089.6 | Fndc3b | 2:113112901-113345577 | 0.0013 | 6.04 | 0.0413 |
| ENSRNOG00000062197.1 | AABR07030514.1 | 10:90932053-90932937 | 0.0013 | -6.35 | 0.0413 |
| ENSRNOG00000002408.6 | Rbm47 | 14:43810118-43837662 | 0.0014 | 3.30 | 0.0429 |
| ENSRNOG00000013195.6 | Ruvbl1 | 4:120411990-120447455 | 0.0014 | -2.01 | 0.0431 |
| ENSRNOG00000037148.4 | Adap2 | 10:67494983-67526426 | 0.0014 | 3.12 | 0.0431 |
| ENSRNOG00000019000.8 | Limk2 | 14:83573927-83641892 | 0.0014 | -2.00 | 0.0436 |
| ENSRNOG00000061096.1 | Rn7sl1 | 6:91456907-91457207 | 0.0014 | -21.29 | 0.0441 |
| ENSRNOG00000004019.3 | Phlda1 | 7:54247459-54249664 | 0.0014 | 3.72 | 0.0442 |
| ENSRNOG00000023433.6 | Gata6 | 18:2416551-2446338 | 0.0014 | 3.64 | 0.0442 |
| ENSRNOG00000001926.6 | Cldn1 | 11:77815180-77830416 | 0.0015 | 2.66 | 0.0448 |
| ENSRNOG00000001329.4 | Gjc3 | 12:19166238-19167015 | 0.0015 | 4.92 | 0.0453 |
| ENSRNOG00000007725.2 | Mis12 | 10:57660066-57660687 | 0.0015 | 5.58 | 0.0453 |
| ENSRNOG00000015692.6 | Taok1 | 10:62566731-62630155 | 0.0015 | 5.38 | 0.0453 |
| ENSRNOG00000031335.4 | Ankrd37 | 16:49462888-49465888 | 0.0015 | 4.49 | 0.0453 |
| ENSRNOG00000037911.2 | LOC680227 | X:74304291-74345507 | 0.0015 | 5.41 | 0.0453 |
| ENSRNOG00000050465.1 | AABR07041109.1 | X:119532512-119532965 | 0.0015 | 5.49 | 0.0453 |
| ENSRNOG00000060289.1 | 7SK | X:23467546-23467864 | 0.0015 | -17.81 | 0.0454 |
| ENSRNOG00000003546.5 | Tnfrsf12a | 10:12995903-12997930 | 0.0015 | 3.41 | 0.0461 |
| ENSRNOG00000006338.7 | Lrp6 | 4:168194926-168323751 | 0.0016 | 4.58 | 0.0465 |
| ENSRNOG00000027988.5 | Zhx3 | 3:156764750-156777999 | 0.0016 | 4.24 | 0.0465 |
| ENSRNOG00000043866.1 | AY172581.24 | MT:1093-2664 | 0.0016 | -2.15 | 0.0467 |
| ENSRNOG00000001060.7 | Snrnp35 | 12:37531094-37538403 | 0.0016 | -2.26 | 0.0474 |
| ENSRNOG00000029052.3 | LOC108352861 | 14:104987708-104987918 | 0.0016 | -4.11 | 0.0480 |
| ENSRNOG00000019915.5 | Xrcc1 | 1:81413352-81441678 | 0.0016 | -2.02 | 0.0488 |
| ENSRNOG00000053518.1 | Zscan26 | 17:45188247-45207065 | 0.0017 | 2.25 | 0.0490 |

* FDR: false discovery rate. The FDR-adjusted P value was sorted from lowest to highest.

Table S6. Statistics of differentially expressed genes (DEG) with *P* < 0.01, FDR-adjusted P value = 0.05 and |Fold change| > 2 in liver in comparison of CT vs. H120.

| Feature ID | Gene name | Position | P-value | Fold change | FDR-adjusted P value* |
| --- | --- | --- | --- | --- | --- |
| ENSRNOG00000045636.2 | Fasn | 10:109987734-110005901 | 5.31751E-31 | -6.61 | 1.74E-26 |
| ENSRNOG00000050647.2 | Hspa1b | 20:4877323-4879779 | 8.5167E-25 | 315.32 | 1.40E-20 |
| ENSRNOG00000027784.4 | Tsku | 1:163317281-163328591 | 4.0439E-23 | -4.49 | 4.43E-19 |
| ENSRNOG00000026965.6 | Tmem140 | 4:62380913-62391912 | 1.06771E-20 | 4.20 | 8.77E-17 |
| ENSRNOG00000013376.6 | Mvd | 19:55258904-55268951 | 4.10275E-20 | -4.37 | 2.24E-16 |
| ENSRNOG00000016924.8 | Acly | 10:88392247-88442845 | 4.08504E-20 | -4.14 | 2.24E-16 |
| ENSRNOG00000003597.5 | Tuba4a | 9:82415604-82419288 | 3.36128E-19 | -3.42 | 1.57E-15 |
| ENSRNOG00000050655.1 | P4ha1 | 20:28920615-28971966 | 4.53861E-19 | 9.66 | 1.86E-15 |
| ENSRNOG00000020480.5 | Fads1 | 1:226234073-226249138 | 8.71625E-19 | -2.14 | 3.18E-15 |
| ENSRNOG00000010478.7 | LOC299282 | 6:127941525-128080889 | 1.13012E-18 | 2.27 | 3.71E-15 |
| ENSRNOG00000028616.6 | PCT1 | 3:171213935-171219871 | 1.97435E-17 | 9.76 | 5.41E-14 |
| ENSRNOG00000058780.1 | Igfbp1 | 14:87448691-87453785 | 1.9639E-17 | 5.72 | 5.41E-14 |
| ENSRNOG00000049075.2 | Fabp5 | 2:93981655-93985378 | 2.70186E-17 | -6.24 | 6.83E-14 |
| ENSRNOG00000000902.7 | Hsph1 | 12:6322667-6341902 | 3.68958E-17 | 9.43 | 8.66E-14 |
| ENSRNOG00000021027.6 | Dbp | 1:101687854-101692846 | 6.70204E-17 | -14.47 | 1.46E-13 |
| ENSRNOG00000003228.5 | Mid1ip1 | X:13114568-13116743 | 4.54863E-16 | -3.98 | 8.79E-13 |
| ENSRNOG00000022268.6 | Pnpla3 | 7:125034763-125055976 | 1.01129E-15 | -22.26 | 1.84E-12 |
| ENSRNOG00000034116.6 | Gk | X:54227396-54303864 | 1.31561E-15 | 4.57 | 2.27E-12 |
| ENSRNOG00000056457.1 | Gpd1 | 7:141370490-141377928 | 1.47288E-15 | -2.61 | 2.42E-12 |
| ENSRNOG00000006331.4 | Elovl5 | 8:85259981-85285983 | 3.39888E-15 | 3.64 | 5.32E-12 |
| ENSRNOG00000052142.1 | Ahsa2 | 14:108265206-108273938 | 5.35572E-15 | 4.40 | 8.00E-12 |
| ENSRNOG00000018755.6 | Acss2 | 3:151032951-151075856 | 9.35593E-15 | -2.78 | 1.33E-11 |
| ENSRNOG00000016122.4 | Hmgcr | 2:27480225-27500654 | 7.39632E-14 | -6.82 | 1.01E-10 |
| ENSRNOG00000002520.5 | Litaf | 10:4719712-4763510 | 1.09574E-13 | 4.55 | 1.44E-10 |
| ENSRNOG00000053086.1 | Selenop | 2:53109683-53114858 | 1.23775E-13 | 2.83 | 1.56E-10 |
| ENSRNOG00000048088.2 | Mest | 4:58053040-58063138 | 1.99812E-13 | 34.24 | 2.43E-10 |
| ENSRNOG00000045654.2 | LOC108348108 | 20:2699711-2701815 | 2.59111E-13 | 461.79 | 2.98E-10 |
| ENSRNOG00000059714.1 | Hsp90aa1 | 6:135107270-135112775 | 2.63648E-13 | 3.84 | 2.98E-10 |
| ENSRNOG00000020704.5 | Tkfc | 1:226644199-226657408 | 3.86516E-13 | -2.82 | 4.23E-10 |
| ENSRNOG00000053362.1 | Gabarapl1 | 4:163293723-163302858 | 5.98395E-13 | 2.59 | 6.34E-10 |
| ENSRNOG00000000967.5 | Aacs | 12:36512392-36555694 | 7.08969E-13 | -5.95 | 7.28E-10 |
| ENSRNOG00000017120.6 | Abhd2 | 1:140998239-141087405 | 8.85879E-13 | 4.54 | 8.82E-10 |
| ENSRNOG00000006789.6 | Ddit3 | 7:70580197-70585084 | 1.18797E-12 | 3.35 | 1.14E-09 |
| ENSRNOG00000000053.7 | Crp | 13:91054973-91093713 | 1.23273E-12 | 2.99 | 1.15E-09 |
| ENSRNOG00000001442.3 | Por | 12:23998410-24046814 | 1.29471E-12 | -3.05 | 1.18E-09 |
| ENSRNOG00000012404.6 | Thrsp | 1:162381252-162385575 | 1.54765E-12 | -3.65 | 1.37E-09 |
| ENSRNOG00000002478.5 | Insig2 | 13:37266311-37287458 | 2.47756E-12 | 3.07 | 2.14E-09 |
| ENSRNOG00000009488.4 | Cyp7a1 | 5:19358733-19368431 | 2.86255E-12 | -5.53 | 2.41E-09 |
| ENSRNOG00000022392.3 | Hspb8 | 12:45905370-45920013 | 3.31444E-12 | 3.27 | 2.72E-09 |
| ENSRNOG00000058646.1 | Zfp36l1 | 6:103308044-103313074 | 4.75766E-12 | 3.66 | 3.81E-09 |
| ENSRNOG00000020420.6 | Pklr | 2:188449209-188459592 | 4.91202E-12 | -3.06 | 3.84E-09 |
| ENSRNOG00000049814.2 | LOC100910882 | 3:152226709-152259156 | 5.07325E-12 | 69.14 | 3.87E-09 |
| ENSRNOG00000032297.2 | Msmo1 | 16:26859396-26875973 | 7.15069E-12 | -2.68 | 5.26E-09 |
| ENSRNOG00000042344.1 | Smim22 | 10:10766207-10767389 | 7.20837E-12 | -3.25 | 5.26E-09 |
| ENSRNOG00000015195.6 | Hdhd3 | 5:78358202-78361647 | 1.06783E-11 | -4.15 | 7.45E-09 |
| ENSRNOG00000023546.5 | Hspb1 | 12:23839398-23841049 | 1.19931E-11 | 16.34 | 8.04E-09 |
| ENSRNOG00000001142.3 | Prkab1 | 12:46316235-46326790 | 1.41342E-11 | -3.17 | 9.29E-09 |
| ENSRNOG00000034093.4 | AABR07011951.1 | 2:177651240-177653288 | 2.59162E-11 | 3.74 | 1.60E-08 |
| ENSRNOG00000036693.2 | Slc25a10 | 10:109665681-109673143 | 2.5788E-11 | -2.72 | 1.60E-08 |
| ENSRNOG00000019189.7 | Acat2 | 1:47972398-47992653 | 3.30279E-11 | -3.46 | 2.01E-08 |
| ENSRNOG00000003977.5 | Dusp1 | 10:16970625-16973418 | 4.04603E-11 | 6.43 | 2.41E-08 |
| ENSRNOG00000046727.2 | Abcc2 | 1:263554452-263613252 | 8.13161E-11 | 2.28 | 4.77E-08 |
| ENSRNOG00000017693.7 | Slc2a5 | 5:167141874-167174310 | 8.35571E-11 | -7.36 | 4.82E-08 |
| ENSRNOG00000042838.3 | Junb | 19:26092973-26094756 | 1.17126E-10 | 4.95 | 6.64E-08 |
| ENSRNOG00000016791.8 | Chka | 1:219077770-219126220 | 1.5635E-10 | 4.60 | 8.60E-08 |
| ENSRNOG00000022054.2 | Paqr7 | 5:152708774-152720229 | 1.88043E-10 | -4.73 | 1.01E-07 |
| ENSRNOG00000012929.6 | Wsb1 | 10:66586923-66602987 | 3.41006E-10 | 3.21 | 1.77E-07 |
| ENSRNOG00000010658.6 | LOC103691744 | 2:264266983-264293046 | 3.59268E-10 | 3.06 | 1.84E-07 |
| ENSRNOG00000011016.7 | Slc7a2 | 16:54460066-54513349 | 4.0455E-10 | 4.81 | 2.04E-07 |
| ENSRNOG00000016831.6 | Serpinh1 | 1:164301009-164308317 | 4.10089E-10 | 2.99 | 2.04E-07 |
| ENSRNOG00000011250.6 | Inmt | 4:85381888-85386231 | 4.9422E-10 | -43.75 | 2.38E-07 |
| ENSRNOG00000007229.2 | Nr0b2 | 5:151776003-151779319 | 5.67341E-10 | -4.95 | 2.70E-07 |
| ENSRNOG00000021824.5 | Dnajb1 | 19:24747177-24750919 | 6.10062E-10 | 5.66 | 2.86E-07 |
| ENSRNOG00000003620.5 | Fmo3 | 13:80837419-80862963 | 7.82817E-10 | 2.35 | 3.62E-07 |
| ENSRNOG00000029993.5 | Kynu | 3:28416953-28566928 | 8.4494E-10 | 2.42 | 3.85E-07 |
| ENSRNOG00000020029.5 | Mcrip2 | 10:15230800-15235740 | 9.47236E-10 | -3.45 | 4.26E-07 |
| ENSRNOG00000020194.7 | Hes6 | 9:98549704-98551438 | 9.91116E-10 | -3.59 | 4.40E-07 |
| ENSRNOG00000020696.5 | Pmvk | 2:188784221-188793895 | 1.35092E-09 | -2.06 | 5.92E-07 |
| ENSRNOG00000034066.5 | Hspa8 | 8:44990013-44993179 | 1.42139E-09 | 5.37 | 6.07E-07 |
| ENSRNOG00000015774.5 | Mreg | 9:79490049-79545024 | 1.46564E-09 | 2.87 | 6.17E-07 |
| ENSRNOG00000003232.4 | Slc9a3r1 | 10:103713044-103730145 | 1.58563E-09 | -2.57 | 6.60E-07 |
| ENSRNOG00000010887.6 | RGD1309534 | 8:13838571-13861212 | 1.65637E-09 | -4.04 | 6.80E-07 |
| ENSRNOG00000015859.5 | Chdh | 16:6078121-6109382 | 1.67992E-09 | -2.33 | 6.81E-07 |
| ENSRNOG00000007545.6 | Angptl4 | 7:18627807-18634079 | 2.34664E-09 | 2.50 | 9.41E-07 |
| ENSRNOG00000021314.4 | Fdft1 | 15:46339248-46367302 | 3.6959E-09 | -2.10 | 1.44E-06 |
| ENSRNOG00000002572.5 | Cacybp | 13:77948770-77959110 | 3.81973E-09 | 2.37 | 1.47E-06 |
| ENSRNOG00000007040.4 | Timm17a | 13:52124634-52136127 | 3.90253E-09 | -2.96 | 1.49E-06 |
| ENSRNOG00000028092.2 | Cpa2 | 4:57855415-57879239 | 3.96802E-09 | 2.94 | 1.49E-06 |
| ENSRNOG00000016552.6 | Hmgcs1 | 2:52431600-52445055 | 4.31329E-09 | -3.09 | 1.61E-06 |
| ENSRNOG00000009565.4 | Pdk4 | 4:30546663-30556814 | 5.06256E-09 | 4.22 | 1.84E-06 |
| ENSRNOG00000019481.5 | Cyp8b1 | 8:130548417-130550388 | 5.87027E-09 | 2.72 | 2.12E-06 |
| ENSRNOG00000006305.5 | Slc38a2 | 7:138088648-138100841 | 7.79692E-09 | 3.88 | 2.75E-06 |
| ENSRNOG00000016166.4 | Pdlim1 | 1:259308295-259357056 | 7.79405E-09 | -2.46 | 2.75E-06 |
| ENSRNOG00000009715.6 | Me1 | 8:94256838-94368834 | 8.92131E-09 | -4.05 | 3.12E-06 |
| ENSRNOG00000022296.4 | Pnpla5 | 7:124988523-124999137 | 9.17718E-09 | -6.54 | 3.17E-06 |
| ENSRNOG00000002839.6 | Slc19a2 | 13:82552549-82566586 | 9.74806E-09 | 2.34 | 3.33E-06 |
| ENSRNOG00000021405.6 | Cyp2c7 | 1:148053686-148119857 | 1.07558E-08 | 2.13 | 3.60E-06 |
| ENSRNOG00000028668.6 | Slc28a2 | 3:114355797-114647382 | 1.2247E-08 | 3.29 | 4.06E-06 |
| ENSRNOG00000051615.1 | Hmgn2 | 5:152195360-152198813 | 1.24551E-08 | -2.16 | 4.09E-06 |
| ENSRNOG00000021164.7 | Stip1 | 1:222274132-222293148 | 1.58783E-08 | 2.41 | 5.16E-06 |
| ENSRNOG00000012274.8 | Ddi2 | 5:160230954-160282810 | 1.79581E-08 | 2.98 | 5.73E-06 |
| ENSRNOG00000011668.6 | Nfil3 | 17:12261101-12276315 | 2.06122E-08 | 2.87 | 6.51E-06 |
| ENSRNOG00000014448.8 | Arntl | 1:178039062-178137465 | 2.12162E-08 | 9.44 | 6.64E-06 |
| ENSRNOG00000014117.7 | Hmox1 | 19:14508615-14515456 | 2.20877E-08 | 3.61 | 6.85E-06 |
| ENSRNOG00000009094.4 | Nudt4 | 7:36643915-36660141 | 2.82572E-08 | 2.16 | 8.68E-06 |
| ENSRNOG00000012106.4 | Dnaja4 | 8:59278261-59294003 | 2.99631E-08 | 8.29 | 9.12E-06 |
| ENSRNOG00000008474.7 | Acox3 | 14:80314430-80355420 | 3.03672E-08 | 2.52 | 9.16E-06 |
| ENSRNOG00000018494.6 | Ppp1r3c | 1:255371809-255376833 | 3.66785E-08 | 2.67 | 1.06E-05 |
| ENSRNOG00000031769.3 | Chchd7 | 5:16845630-16847776 | 4.21385E-08 | -3.25 | 1.21E-05 |
| ENSRNOG00000019742.5 | Stat3 | 10:88790407-88842233 | 4.48972E-08 | 2.62 | 1.28E-05 |
| ENSRNOG00000009592.6 | Cyb5r3 | 7:124023994-124041594 | 4.99413E-08 | -2.14 | 1.41E-05 |
| ENSRNOG00000018294.6 | Hspa5 | 3:13838303-13842762 | 5.67577E-08 | 2.02 | 1.58E-05 |
| ENSRNOG00000021084.7 | AABR07006310.1 | 1:228724899-228727160 | 6.31991E-08 | 4.00 | 1.73E-05 |
| ENSRNOG00000030538.5 | Slco1b2 | 4:175814117-175881768 | 6.28607E-08 | 2.45 | 1.73E-05 |
| ENSRNOG00000038012.2 | Commd6 | 15:86136095-86142672 | 7.34505E-08 | -2.57 | 1.94E-05 |
| ENSRNOG00000030391.4 | Ei24 | 8:39254888-39266959 | 7.67129E-08 | 2.18 | 2.01E-05 |
| ENSRNOG00000007139.2 | Ttpa | 5:34007919-34028452 | 8.52823E-08 | 2.37 | 2.22E-05 |
| ENSRNOG00000019834.6 | Hsp90ab1 | 9:17817720-17823243 | 9.3508E-08 | 2.22 | 2.42E-05 |
| ENSRNOG00000048949.1 | LOC102549542 | 2:234375314-234483149 | 9.84849E-08 | -3.76 | 2.51E-05 |
| ENSRNOG00000013975.7 | Acat2l1 | 1:47996520-48026268 | 1.1701E-07 | -3.02 | 2.95E-05 |
| ENSRNOG00000010224.6 | Rab30 | 1:157573323-157660008 | 1.18889E-07 | 4.45 | 2.98E-05 |
| ENSRNOG00000049900.1 | Irf2bp2 | 19:59499875-59502161 | 1.56399E-07 | 4.00 | 3.83E-05 |
| ENSRNOG00000020506.6 | Khnyn | 15:34552409-34561727 | 1.73171E-07 | 5.85 | 4.21E-05 |
| ENSRNOG00000005615.6 | Gadd45a | 4:97782511-97784842 | 1.79059E-07 | -2.83 | 4.32E-05 |
| ENSRNOG00000012443.5 | Cpt2 | 5:127505613-127523089 | 3.30515E-07 | -2.30 | 7.87E-05 |
| ENSRNOG00000056728.1 | G6pd | X:156274799-156293926 | 3.40038E-07 | -3.85 | 8.04E-05 |
| ENSRNOG00000000827.6 | Ier3 | 20:3438797-3440769 | 3.5879E-07 | 2.16 | 8.33E-05 |
| ENSRNOG00000001797.6 | Umps | 11:70034138-70044628 | 4.06945E-07 | -2.26 | 9.18E-05 |
| ENSRNOG00000047706.3 | LOC103690108 | 20:3791406-3794027 | 4.0777E-07 | 3.39 | 9.18E-05 |
| ENSRNOG00000012123.3 | Fdx1 | 8:56373722-56393233 | 4.37205E-07 | 2.29 | 9.78E-05 |
| ENSRNOG00000023150.5 | Gpi | 1:90063410-90091287 | 5.30533E-07 | -2.08 | 0.0001 |
| ENSRNOG00000009920.5 | LOC680121 | 11:13499163-13501263 | 5.34375E-07 | 2.41 | 0.0001 |
| ENSRNOG00000055391.1 | Eif4ebp2 | 20:31055788-31072469 | 5.67494E-07 | 2.76 | 0.0001 |
| ENSRNOG00000014806.7 | Pnkd | 9:81566073-81634534 | 6.22836E-07 | 2.29 | 0.0001 |
| ENSRNOG00000022657.3 | Tmem97 | 10:65811455-65820538 | 6.64515E-07 | -2.01 | 0.0001 |
| ENSRNOG00000046379.2 | Srm | 3:14588869-14591974 | 7.34035E-07 | -7.28 | 0.0002 |
| ENSRNOG00000005447.5 | Ypel2 | 10:74243811-74298599 | 7.97315E-07 | 8.54 | 0.0002 |
| ENSRNOG00000014532.2 | Lbp | 3:154786214-154813464 | 8.18971E-07 | 2.56 | 0.0002 |
| ENSRNOG00000011497.4 | Aldh1b1 | 5:61382350-61387358 | 8.32709E-07 | -2.64 | 0.0002 |
| ENSRNOG00000019428.7 | Higd1a | 8:130482492-130491998 | 8.6202E-07 | -2.13 | 0.0002 |
| ENSRNOG00000032917.3 | Zfand2a | 12:17252092-17263477 | 8.86039E-07 | 10.59 | 0.0002 |
| ENSRNOG00000008245.7 | AABR07054614.1 | 3:163950776-163986059 | 9.55892E-07 | -5.22 | 0.0002 |
| ENSRNOG00000029360.5 | Serinc1 | 20:38967237-38985036 | 9.53044E-07 | 2.44 | 0.0002 |
| ENSRNOG00000053210.1 | Zc3h11a | 13:50196041-50234862 | 9.53321E-07 | 3.08 | 0.0002 |
| ENSRNOG00000057333.1 | AABR07030184.1 | 10:74765384-74765600 | 1.14802E-06 | -15.23 | 0.0002 |
| ENSRNOG00000009446.4 | Rxra | 3:6211788-6295908 | 1.25732E-06 | 2.46 | 0.0002 |
| ENSRNOG00000054945.1 | AABR07015081.2 | 14:46653039-46657975 | 1.27809E-06 | 3.38 | 0.0002 |
| ENSRNOG00000011796.7 | C1r | 4:157125997-157136829 | 1.31842E-06 | 2.08 | 0.0002 |
| ENSRNOG00000020298.6 | Bag3 | 1:199941160-199965191 | 1.63513E-06 | 5.56 | 0.0003 |
| ENSRNOG00000007029.3 | Dnaja1 | 5:57028466-57039378 | 1.86066E-06 | 2.35 | 0.0003 |
| ENSRNOG00000029698.5 | Pim3 | 7:129860113-129863441 | 1.85102E-06 | 2.84 | 0.0003 |
| ENSRNOG00000017523.5 | H6pd | 5:166998880-167030441 | 1.92805E-06 | 2.29 | 0.0003 |
| ENSRNOG00000057832.1 | Rnf125 | 18:15192961-15225427 | 2.12669E-06 | 3.72 | 0.0004 |
| ENSRNOG00000042821.3 | Cd59 | 3:94010474-94028621 | 2.17762E-06 | 2.29 | 0.0004 |
| ENSRNOG00000036837.3 | Nfe2 | 7:144872746-144880092 | 2.25316E-06 | -6.93 | 0.0004 |
| ENSRNOG00000002802.3 | Cxcl1 | 14:18743684-18745457 | 2.32651E-06 | 5.78 | 0.0004 |
| ENSRNOG00000011696.6 | Lifr | 2:56426366-56489415 | 2.4174E-06 | 2.87 | 0.0004 |
| ENSRNOG00000004377.6 | Lpin1 | 6:41799748-41870046 | 2.4914E-06 | 4.09 | 0.0004 |
| ENSRNOG00000016456.7 | Il33 | 1:248132089-248147029 | 2.56109E-06 | -2.00 | 0.0004 |
| ENSRNOG00000004659.5 | Creld2 | 7:129812788-129819634 | 2.75613E-06 | 2.53 | 0.0005 |
| ENSRNOG00000029668.4 | Wfdc21 | 10:71047309-71053191 | 2.88186E-06 | -3.17 | 0.0005 |
| ENSRNOG00000009994.5 | Dlat | 8:55062550-55087832 | 3.77419E-06 | -3.18 | 0.0006 |
| ENSRNOG00000033195.6 | A1cf | 1:250426157-250514866 | 4.09381E-06 | 4.86 | 0.0007 |
| ENSRNOG00000013133.3 | Foxa2 | 3:142383277-142387481 | 4.21927E-06 | -2.63 | 0.0007 |
| ENSRNOG00000009875.2 | Akr1b7 | 4:61850347-61862526 | 4.31483E-06 | 2.28 | 0.0007 |
| ENSRNOG00000053452.1 | LOC100361457 | 3:75643053-75644954 | 4.53935E-06 | -2.73 | 0.0007 |
| ENSRNOG00000020060.4 | Atf5 | 1:100808240-100810522 | 4.70468E-06 | 2.35 | 0.0007 |
| ENSRNOG00000016690.6 | Idi1 | 17:57976267-57984036 | 4.76292E-06 | -2.24 | 0.0007 |
| ENSRNOG00000033740.3 | Lurap1l | 5:98469046-98515125 | 4.81277E-06 | 2.20 | 0.0007 |
| ENSRNOG00000010558.7 | Ppif | 16:1979190-1985908 | 5.07902E-06 | -2.16 | 0.0008 |
| ENSRNOG00000047816.2 | Ccs | 1:220075246-220096404 | 5.84399E-06 | -2.16 | 0.0009 |
| ENSRNOG00000005861.6 | Hsd11b1 | 13:111926441-111972603 | 6.02172E-06 | -3.04 | 0.0009 |
| ENSRNOG00000010643.7 | Kank2 | 8:22791994-22821397 | 6.54673E-06 | -3.44 | 0.0010 |
| ENSRNOG00000021240.4 | Atrn | 3:123434408-123567918 | 6.7444E-06 | 2.89 | 0.0010 |
| ENSRNOG00000010944.6 | Hyou1 | 8:48699768-48711910 | 6.99904E-06 | 2.26 | 0.0010 |
| ENSRNOG00000010951.7 | Cmtm6 | 8:122789452-122807560 | 7.64039E-06 | 2.41 | 0.0011 |
| ENSRNOG00000019383.7 | Tef | 7:123043502-123058604 | 8.02885E-06 | -3.40 | 0.0012 |
| ENSRNOG00000049236.2 | Myh9l1 | 7:118992355-119071712 | 8.29636E-06 | 3.24 | 0.0012 |
| ENSRNOG00000001738.8 | Eif4g1 | 11:83908108-83926524 | 8.64265E-06 | 2.66 | 0.0013 |
| ENSRNOG00000047089.1 | Alkbh7 | 9:10045374-10047507 | 9.13127E-06 | -2.31 | 0.0013 |
| ENSRNOG00000023360.4 | Fus | 1:199412833-199426702 | 9.41312E-06 | -2.27 | 0.0013 |
| ENSRNOG00000049132.2 | Map2k3 | 10:45089463-45110787 | 9.38659E-06 | -2.49 | 0.0013 |
| ENSRNOG00000054181.1 | LOC100361547 | 1:148240503-148287217 | 9.55884E-06 | 2.37 | 0.0013 |
| ENSRNOG00000005424.4 | Odc1 | 6:42852682-42859927 | 9.76398E-06 | -2.54 | 0.0014 |
| ENSRNOG00000003302.5 | Flcn | 10:46153187-46172309 | 1.00198E-05 | 2.91 | 0.0014 |
| ENSRNOG00000009550.7 | Sqle | 7:99609190-99624732 | 1.02621E-05 | -2.25 | 0.0014 |
| ENSRNOG00000013963.7 | Il6st | 2:44289392-44314944 | 1.0544E-05 | 3.76 | 0.0014 |
| ENSRNOG00000006663.8 | Usp2 | 8:48406259-48430885 | 1.06031E-05 | -9.40 | 0.0014 |
| ENSRNOG00000000521.4 | Cdkn1a | 20:6351457-6358864 | 1.12832E-05 | 5.82 | 0.0015 |
| ENSRNOG00000008074.6 | Cyp11a1 | 8:62779874-62809893 | 1.13017E-05 | 14.66 | 0.0015 |
| ENSRNOG00000020836.7 | Rorc | 2:195617020-195637630 | 1.19994E-05 | -2.09 | 0.0016 |
| ENSRNOG00000003260.6 | Nr1i3 | 13:89586282-89591277 | 1.2292E-05 | -4.42 | 0.0016 |
| ENSRNOG00000043193.2 | Smim1 | 5:171309334-171312026 | 1.25021E-05 | -2.45 | 0.0016 |
| ENSRNOG00000004342.7 | Ahr | 6:54963989-55001464 | 1.27788E-05 | 2.88 | 0.0017 |
| ENSRNOG00000046643.2 | Cyp3a9 | 12:19074582-19114399 | 1.33528E-05 | 2.54 | 0.0017 |
| ENSRNOG00000020624.4 | Acadsb | 1:201981356-202021008 | 1.51212E-05 | 3.23 | 0.0019 |
| ENSRNOG00000026643.5 | Chordc1 | 8:17421556-17446165 | 1.57676E-05 | 2.45 | 0.0020 |
| ENSRNOG00000011269.6 | Sult1c3 | 9:4930817-4978892 | 1.6572E-05 | -2.62 | 0.0021 |
| ENSRNOG00000013907.7 | Sall1 | 19:23389374-23405039 | 1.68689E-05 | 2.39 | 0.0021 |
| ENSRNOG00000013552.6 | Scd | 1:264160128-264172729 | 1.69905E-05 | -3.07 | 0.0021 |
| ENSRNOG00000059330.1 | AABR07004549.1 | 1:147422778-147435897 | 1.73699E-05 | 2.01 | 0.0021 |
| ENSRNOG00000054549.1 | Lss | 20:12842883-12870497 | 1.77184E-05 | -2.01 | 0.0022 |
| ENSRNOG00000008510.6 | Abtb2 | 3:93495105-93647721 | 1.88223E-05 | 3.34 | 0.0023 |
| ENSRNOG00000038999.4 | RT1-A1 | 20:5351604-5421098 | 1.8787E-05 | 2.03 | 0.0023 |
| ENSRNOG00000059593.1 | Cetn2 | X:152927717-152932953 | 2.03275E-05 | -2.11 | 0.0024 |
| ENSRNOG00000007793.6 | Pnrc1 | 5:48501471-48504511 | 2.18171E-05 | 2.08 | 0.0026 |
| ENSRNOG00000047598.1 | Ctdsp2 | 7:70283709-70288568 | 2.32256E-05 | 3.41 | 0.0027 |
| ENSRNOG00000002292.8 | Hnrnpd | 14:11256267-11274578 | 2.39682E-05 | -2.31 | 0.0028 |
| ENSRNOG00000013265.6 | Tgfbr2 | 8:124312753-124399494 | 2.40366E-05 | 3.40 | 0.0028 |
| ENSRNOG00000014617.7 | Tgoln2 | 4:100475415-100483168 | 2.66456E-05 | 2.05 | 0.0031 |
| ENSRNOG00000016099.2 | Id4 | 17:16692556-16695126 | 2.7243E-05 | 2.26 | 0.0031 |
| ENSRNOG00000013328.8 | Rbpms | 16:61954589-62096497 | 2.79532E-05 | 2.46 | 0.0032 |
| ENSRNOG00000020578.8 | Ceacam1 | 1:82327954-82344345 | 2.97633E-05 | 2.07 | 0.0034 |
| ENSRNOG00000015052.4 | Star | 16:71036203-71040847 | 3.06967E-05 | 11.52 | 0.0035 |
| ENSRNOG00000060518.1 | AABR07015057.1 | 14:46523593-46529375 | 3.0827E-05 | 2.32 | 0.0035 |
| ENSRNOG00000050869.2 | Cebpd | 11:89008007-89009146 | 3.12485E-05 | 3.14 | 0.0035 |
| ENSRNOG00000005266.7 | Amdhd1 | 7:34361632-34406318 | 3.2589E-05 | 2.37 | 0.0036 |
| ENSRNOG00000016957.5 | Igfbp2 | 9:80118028-80144789 | 3.3737E-05 | -3.08 | 0.0037 |
| ENSRNOG00000009088.7 | Txnrd1 | 7:26946124-26984400 | 3.70316E-05 | 2.11 | 0.0041 |
| ENSRNOG00000017558.6 | Tubb2a | 17:31493106-31498651 | 3.92488E-05 | 2.22 | 0.0042 |
| ENSRNOG00000003334.5 | Klhl21 | 5:169181417-169190073 | 4.27785E-05 | 3.54 | 0.0046 |
| ENSRNOG00000007124.6 | Krcc1 | 4:99133841-99146827 | 4.71578E-05 | -2.02 | 0.0050 |
| ENSRNOG00000017473.6 | Ttc25 | 10:88459489-88488237 | 4.97161E-05 | -7.07 | 0.0052 |
| ENSRNOG00000019638.7 | Lmna | 2:187842884-187863516 | 5.27029E-05 | -2.28 | 0.0055 |
| ENSRNOG00000004448.7 | Acss3 | 7:49047012-49250957 | 5.40443E-05 | 2.05 | 0.0056 |
| ENSRNOG00000032708.5 | RT1-Bb | 20:4039412-4049711 | 5.46988E-05 | 2.14 | 0.0057 |
| ENSRNOG00000033573.5 | Sgk2 | 3:159368272-159384775 | 5.52479E-05 | -4.11 | 0.0057 |
| ENSRNOG00000031789.5 | Rangap1 | 7:122940375-122967178 | 5.62586E-05 | -2.01 | 0.0058 |
| ENSRNOG00000019000.8 | Limk2 | 14:83573927-83641892 | 5.8952E-05 | -2.38 | 0.0060 |
| ENSRNOG00000007625.4 | B3galt1 | 3:54253948-54267292 | 6.01193E-05 | 5.14 | 0.0061 |
| ENSRNOG00000048874.2 | GCTr | 6:26355295-26385761 | 6.06088E-05 | 2.03 | 0.0061 |
| ENSRNOG00000009663.5 | Apex1 | 15:27849978-27852082 | 6.21354E-05 | -2.37 | 0.0062 |
| ENSRNOG00000004640.6 | Mtfp1 | 14:84330217-84334066 | 6.52268E-05 | -2.10 | 0.0065 |
| ENSRNOG00000007763.7 | Plod1 | 5:164720586-164747083 | 6.57348E-05 | 2.22 | 0.0065 |
| ENSRNOG00000033680.5 | Cyp2b1 | 7:99142449-99181783 | 7.10392E-05 | -16.94 | 0.0070 |
| ENSRNOG00000001827.8 | Masp1 | 11:80736575-80803382 | 7.43835E-05 | 3.10 | 0.0072 |
| ENSRNOG00000013408.5 | Npas2 | 9:45901740-46081880 | 7.67748E-05 | 14.30 | 0.0074 |
| ENSRNOG00000046975.2 | Sult4a1 | 7:124958545-124982566 | 7.67748E-05 | -14.26 | 0.0074 |
| ENSRNOG00000001106.7 | Denr | 12:38096033-38116919 | 8.88314E-05 | -2.13 | 0.0085 |
| ENSRNOG00000018397.6 | Dnph1 | 9:16845524-16848503 | 8.9495E-05 | -2.14 | 0.0085 |
| ENSRNOG00000001517.6 | Pdk1 | 3:58530869-58558027 | 9.11992E-05 | -2.35 | 0.0086 |
| ENSRNOG00000057957.1 | Crybg2 | 5:152290083-152321541 | 9.0969E-05 | 3.40 | 0.0086 |
| ENSRNOG00000014828.5 | Avpi1 | 1:261365589-261371508 | 9.14669E-05 | -2.30 | 0.0086 |
| ENSRNOG00000000520.8 | Srsf3 | 20:6288266-6298213 | 9.38112E-05 | -2.31 | 0.0088 |
| ENSRNOG00000004692.7 | A1bg | 7:102294796-102298522 | 9.53771E-05 | 2.35 | 0.0090 |
| ENSRNOG00000057601.1 | AABR07044080.2 | 19:56443631-56443869 | 0.0001 | -3.62 | 0.0094 |
| ENSRNOG00000019422.4 | Egr1 | 18:27657627-27661429 | 0.0001 | -5.04 | 0.0098 |
| ENSRNOG00000010107.8 | AABR07025295.1 | 16:31734943-31946242 | 0.0001 | 5.23 | 0.0099 |
| ENSRNOG00000045924.3 | RT1-T24-3 | 20:3226312-3233694 | 0.0001 | -3.57 | 0.0102 |
| ENSRNOG00000055634.1 | AABR07024908.1 | 16:21807190-21807526 | 0.0001 | -2.00 | 0.0102 |
| ENSRNOG00000046112.2 | Dedd2 | 1:82072543-82088275 | 0.0001 | 3.06 | 0.0103 |
| ENSRNOG00000020185.7 | Wdr6 | 8:117358786-117366096 | 0.0001 | -4.14 | 0.0121 |
| ENSRNOG00000051854.1 | Enpep | 2:233667253-233743866 | 0.0001 | 2.00 | 0.0128 |
| ENSRNOG00000008648.4 | Mogs | 4:113948513-113951847 | 0.0001 | -5.56 | 0.0129 |
| ENSRNOG00000000428.6 | Cyp21a1 | 20:4486218-4489550 | 0.0002 | 6.08 | 0.0144 |
| ENSRNOG00000012100.6 | Ssbp1 | 4:68634928-68645172 | 0.0002 | -2.09 | 0.0148 |
| ENSRNOG00000008519.7 | Dipk2a | 8:102088504-102159828 | 0.0002 | 3.64 | 0.0148 |
| ENSRNOG00000001030.8 | Tsc22d1 | 15:58554373-58658153 | 0.0002 | -2.13 | 0.0150 |
| ENSRNOG00000031802.4 | LOC691427 | 1:141218094-141218277 | 0.0002 | -3.60 | 0.0150 |
| ENSRNOG00000033609.4 | Irx1 | 1:33910911-33916741 | 0.0002 | -2.38 | 0.0152 |
| ENSRNOG00000009639.5 | Zrsr1 | 14:107750161-107751892 | 0.0002 | 4.77 | 0.0155 |
| ENSRNOG00000009734.4 | Akr1b8 | 4:61771969-61828657 | 0.0002 | 5.70 | 0.0156 |
| ENSRNOG00000011857.7 | Mtpn | 4:63012010-63039422 | 0.0002 | 2.34 | 0.0156 |
| ENSRNOG00000012826.5 | Creb3l2 | 4:64869025-64981384 | 0.0002 | 4.87 | 0.0163 |
| ENSRNOG00000008927.4 | Hbp1 | 6:51231479-51257625 | 0.0002 | 2.09 | 0.0164 |
| ENSRNOG00000009284.6 | Foxa1 | 6:78545803-78549669 | 0.0002 | 4.49 | 0.0164 |
| ENSRNOG00000001926.6 | Cldn1 | 11:77815180-77830416 | 0.0002 | 3.03 | 0.0166 |
| ENSRNOG00000029042.3 | Mt-nd6 | MT:13542-14061 | 0.0002 | -2.04 | 0.0167 |
| ENSRNOG00000059255.1 | Rnu3a | 20:45357178-45357393 | 0.0002 | -31.87 | 0.0176 |
| ENSRNOG00000037198.4 | Usp18 | 4:153805992-153834430 | 0.0002 | -4.12 | 0.0177 |
| ENSRNOG00000002280.6 | Sh3bgrl | X:80213331-80309936 | 0.0002 | 2.28 | 0.0178 |
| ENSRNOG00000013954.5 | Alpl | 5:156086496-156141537 | 0.0002 | -9.68 | 0.0180 |
| ENSRNOG00000023271.7 | Parl | 11:84517367-84544462 | 0.0002 | -2.24 | 0.0182 |
| ENSRNOG00000058183.1 | AABR07040624.1 | X:106607046-106607352 | 0.0002 | -2.06 | 0.0184 |
| ENSRNOG00000028382.3 | Rfxapl1 | 2:144213402-144217600 | 0.0002 | 22.05 | 0.0194 |
| ENSRNOG00000019639.6 | Pgpep1 | 16:20521955-20531396 | 0.0003 | 2.07 | 0.0198 |
| ENSRNOG00000021678.5 | Alkbh3 | 3:82874247-82906581 | 0.0003 | -2.07 | 0.0198 |
| ENSRNOG00000000909.8 | Uspl1 | 12:6929977-6956984 | 0.0003 | 3.23 | 0.0222 |
| ENSRNOG00000058555.1 | 7SK | 8:85480291-85480622 | 0.0003 | -29.27 | 0.0222 |
| ENSRNOG00000006990.6 | Grb7 | 10:86399826-86409348 | 0.0003 | 2.48 | 0.0223 |
| ENSRNOG00000007817.5 | Kctd6 | 15:18484509-18493366 | 0.0003 | 2.36 | 0.0228 |
| ENSRNOG00000019412.5 | Rhbg | 2:187610086-187622373 | 0.0003 | -2.57 | 0.0230 |
| ENSRNOG00000021575.6 | Cfhr2 | 13:56642364-56693968 | 0.0003 | 2.08 | 0.0237 |
| ENSRNOG00000051171.2 | G6pc | 10:89285854-89296213 | 0.0003 | 3.14 | 0.0237 |
| ENSRNOG00000005387.6 | Rbm3 | X:15098903-15102340 | 0.0003 | -2.42 | 0.0240 |
| ENSRNOG00000012428.5 | Maf | 19:48194803-48196748 | 0.0003 | 2.85 | 0.0240 |
| ENSRNOG00000002609.7 | Ero1b | 17:90710554-90756048 | 0.0003 | 2.71 | 0.0241 |
| ENSRNOG00000021680.7 | Setdb2 | 15:39712860-39742103 | 0.0003 | 4.59 | 0.0242 |
| ENSRNOG00000004294.5 | Ascl1 | 7:28038661-28040510 | 0.0003 | -3.10 | 0.0243 |
| ENSRNOG00000000165.8 | Pfkfb1 | X:23092158-23144324 | 0.0003 | -2.05 | 0.0243 |
| ENSRNOG00000060289.1 | 7SK | X:23467546-23467864 | 0.0003 | -38.80 | 0.0245 |
| ENSRNOG00000060337.1 | Exosc7 | 8:132204603-132229969 | 0.0003 | -2.19 | 0.0246 |
| ENSRNOG00000012862.5 | Spsb4 | 8:104840939-104912959 | 0.0003 | -3.06 | 0.0247 |
| ENSRNOG00000002946.3 | Socs3 | 10:106975177-106976040 | 0.0003 | 4.04 | 0.0249 |
| ENSRNOG00000004726.7 | Mapkapk2 | 13:47785160-47831110 | 0.0003 | 2.05 | 0.0252 |
| ENSRNOG00000009117.5 | Otub2 | 6:127282465-127301083 | 0.0004 | -2.47 | 0.0254 |
| ENSRNOG00000033372.5 | Klhl24 | 11:84615759-84633504 | 0.0004 | 3.53 | 0.0257 |
| ENSRNOG00000000781.3 | Rnf39 | 20:2098374-2103864 | 0.0004 | 2.50 | 0.0266 |
| ENSRNOG00000027434.5 | Fitm2 | 3:159856993-159863506 | 0.0004 | -2.69 | 0.0267 |
| ENSRNOG00000055079.1 | Ndufaf2 | 2:39321740-39434560 | 0.0004 | -2.04 | 0.0273 |
| ENSRNOG00000004744.5 | Lratd2 | 7:101139375-101140308 | 0.0004 | 3.64 | 0.0281 |
| ENSRNOG00000002278.7 | Tec | 14:37918651-38027925 | 0.0004 | 2.35 | 0.0288 |
| ENSRNOG00000019485.6 | BCTdk | 1:199351627-199356881 | 0.0004 | -2.24 | 0.0290 |
| ENSRNOG00000005811.2 | LOC688655 | 6:34553366-34555306 | 0.0004 | 3.39 | 0.0294 |
| ENSRNOG00000015498.8 | Il17rb | 16:6064287-6077978 | 0.0004 | -3.21 | 0.0298 |
| ENSRNOG00000016731.8 | Tpm2 | 5:59016621-59025631 | 0.0004 | -3.90 | 0.0309 |
| ENSRNOG00000010728.6 | Stradb | 9:65763436-65782513 | 0.0005 | 2.08 | 0.0312 |
| ENSRNOG00000017775.6 | Slc5a1 | 14:82909980-82975263 | 0.0005 | 12.75 | 0.0312 |
| ENSRNOG00000006924.7 | Tmem125 | 5:137370576-137372993 | 0.0005 | -2.06 | 0.0313 |
| ENSRNOG00000002832.5 | Slc16a2 | X:74577130-74706214 | 0.0005 | 2.08 | 0.0314 |
| ENSRNOG00000020640.7 | Zbtb7b | 2:188704686-188718704 | 0.0005 | 2.23 | 0.0316 |
| ENSRNOG00000002826.5 | Hsd17b7 | 13:88288045-88307988 | 0.0005 | -2.73 | 0.0317 |
| ENSRNOG00000057470.1 | Pla2g12a | 2:235311718-235327972 | 0.0005 | 2.65 | 0.0328 |
| ENSRNOG00000000787.7 | AABR07044364.1 | 20:3242469-3249189 | 0.0005 | -5.17 | 0.0329 |
| ENSRNOG00000045649.2 | Arrdc3 | 2:8732931-8743069 | 0.0005 | 2.42 | 0.0362 |
| ENSRNOG00000018109.4 | Clic4 | 5:153568744-153625869 | 0.0006 | 3.64 | 0.0365 |
| ENSRNOG00000051170.2 | Ddx17 | 7:120761825-120780641 | 0.0006 | 2.60 | 0.0370 |
| ENSRNOG00000014504.6 | Il1r1 | 9:46962287-47036670 | 0.0006 | 3.41 | 0.0380 |
| ENSRNOG00000042714.2 | RGD1559459 | 14:22937420-22952822 | 0.0006 | 2.49 | 0.0391 |
| ENSRNOG00000048152.2 | Myo1b | 9:54558201-54766054 | 0.0006 | 2.42 | 0.0396 |
| ENSRNOG00000003127.4 | Spryd4 | 7:2621960-2623781 | 0.0006 | -2.26 | 0.0409 |
| ENSRNOG00000001782.6 | Osbpl11 | 11:70770506-70833577 | 0.0007 | -2.29 | 0.0441 |
| ENSRNOG00000058561.1 | Srrm2 | 10:13145098-13162343 | 0.0007 | 2.09 | 0.0448 |
| ENSRNOG00000049604.2 | Mvk | 12:47904718-47919400 | 0.0007 | -2.05 | 0.0451 |
| ENSRNOG00000012067.6 | Fam111a | 1:229003960-229019527 | 0.0007 | -25.36 | 0.0453 |
| ENSRNOG00000030345.4 | LOC100362684 | 9:1012090-1012450 | 0.0007 | -5.51 | 0.0454 |
| ENSRNOG00000008757.6 | Tmem218 | 8:39687268-39702902 | 0.0008 | -2.15 | 0.0473 |
| ENSRNOG00000010421.6 | Wdr91 | 4:62398796-62438958 | 0.0008 | 2.49 | 0.0484 |
| ENSRNOG00000002191.5 | LOC498368 | 14:46050749-46054022 | 0.0008 | -2.52 | 0.0484 |

* FDR: false discovery rate. The FDR-adjusted P value was sorted from lowest to highest.

Table S7. Statistics of differentially expressed genes (DEG) with *P* < 0.01, FDR-adjusted P value = 0.05 and |Fold change| > 2 in adrenal glands in comparison of CT vs. H30.

| Feature ID | Gene name | Position | P-value | Fold change | FDR-adjusted P value * |
| --- | --- | --- | --- | --- | --- |
| ENSRNOG00000048199.2 | Rps19l2 | 9:16802318-16807966 | 9.40E-23 | 24.92 | 3.09E-18 |
| ENSRNOG00000033024.2 | AC099453.1 | 5:78392714-78393143 | 2.58E-20 | -6.80 | 4.24E-16 |
| ENSRNOG00000042838.3 | Junb | 19:26092973-26094756 | 7.67E-20 | 11.02 | 8.41E-16 |
| ENSRNOG00000020480.5 | Fads1 | 1:226234073-226249138 | 7.34E-19 | -3.48 | 6.04E-15 |
| ENSRNOG00000028690.3 | LOC100362366 | 3:24600602-24601063 | 4.22E-18 | -5.31 | 2.77E-14 |
| ENSRNOG00000015160.5 | Gem | 5:25349927-25353661 | 6.38E-18 | 7.25 | 3.50E-14 |
| ENSRNOG00000008015.5 | Fos | 6:109300432-109303299 | 2.43E-17 | 11.99 | 1.14E-13 |
| ENSRNOG00000050647.2 | Hspa1b | 20:4877323-4879779 | 4.47E-17 | 35.00 | 1.84E-13 |
| ENSRNOG00000001189.6 | Sik1 | 20:10668410-10680283 | 2.16E-15 | 6.57 | 7.88E-12 |
| ENSRNOG00000056651.1 | LOC103690068 | 1:88192369-88193346 | 1.18E-14 | 14.19 | 3.90E-11 |
| ENSRNOG00000019422.4 | Egr1 | 18:27657627-27661429 | 2.43E-14 | 7.85 | 7.26E-11 |
| ENSRNOG00000003687.6 | Rgs2 | 13:60846307-60849094 | 3.16E-13 | 6.73 | 8.67E-10 |
| ENSRNOG00000032232.3 | Snrpg | 1:91588227-91588609 | 3.46E-13 | -37.22 | 8.74E-10 |
| ENSRNOG00000009768.6 | LOC100912228 | 4:79557853-79565097 | 3.10E-12 | 33.25 | 7.29E-09 |
| ENSRNOG00000033473.5 | Rpl36 | 9:10441258-10441834 | 1.62E-11 | 3.27 | 3.32E-08 |
| ENSRNOG00000059069.1 | AABR07061378.1 | 4:120040771-120041238 | 1.56E-11 | -3.39 | 3.32E-08 |
| ENSRNOG00000021027.6 | Dbp | 1:101687854-101692846 | 6.72E-11 | -4.21 | 1.25E-07 |
| ENSRNOG00000055564.1 | RGD1564664 | 3:82854776-82856171 | 6.87E-11 | 4.89 | 1.25E-07 |
| ENSRNOG00000021824.5 | Dnajb1 | 19:24747177-24750919 | 7.52E-11 | 8.20 | 1.30E-07 |
| ENSRNOG00000003476.7 | Slc6a4 | 10:63153650-63176463 | 1.41E-10 | 5.71 | 2.21E-07 |
| ENSRNOG00000033803.5 | LOC100360841 | 9:69790830-69791196 | 1.35E-10 | -3.88 | 2.21E-07 |
| ENSRNOG00000000321.5 | Cd24 | 20:48335539-48340846 | 2.27E-10 | 6.70 | 3.39E-07 |
| ENSRNOG00000020938.7 | Ppp1r15a | 1:101511900-101514974 | 3.13E-10 | 2.98 | 4.48E-07 |
| ENSRNOG00000005615.6 | Gadd45a | 4:97782511-97784842 | 3.60E-10 | 5.11 | 4.94E-07 |
| ENSRNOG00000013408.5 | Npas2 | 9:45901740-46081880 | 4.67E-10 | 9.42 | 5.69E-07 |
| ENSRNOG00000019584.6 | Dlk1 | 6:133552820-133583751 | 4.45E-10 | 2.49 | 5.69E-07 |
| ENSRNOG00000058039.1 | Acta2 | 1:252537614-252550394 | 4.56E-10 | 3.61 | 5.69E-07 |
| ENSRNOG00000005600.7 | Nr4a2 | 3:43111239-43119159 | 5.25E-10 | 5.69 | 6.16E-07 |
| ENSRNOG00000037352.3 | RGD1562747 | 8:69484173-69489891 | 5.70E-10 | 5.52 | 6.46E-07 |
| ENSRNOG00000013090.7 | Gadd45g | 17:13391466-13393243 | 6.59E-10 | 5.60 | 6.99E-07 |
| ENSRNOG00000003300.3 | Btg2 | 13:50913179-50916982 | 1.30E-09 | 3.21 | 1.34E-06 |
| ENSRNOG00000047005.2 | Kcnk5 | 15:4554602-4597000 | 1.35E-09 | 3.84 | 1.35E-06 |
| ENSRNOG00000031889.4 | AABR07065438.1 | 6:128738387-128739319 | 1.51E-09 | -3.09 | 1.45E-06 |
| ENSRNOG00000045683.2 | LOC102553715 | 10:49000972-49003931 | 1.54E-09 | 4.89 | 1.45E-06 |
| ENSRNOG00000057078.1 | Ddit4 | 20:29509288-29511382 | 1.97E-09 | 5.56 | 1.80E-06 |
| ENSRNOG00000014448.8 | Arntl | 1:178039062-178137465 | 2.50E-09 | 7.45 | 2.22E-06 |
| ENSRNOG00000014350.4 | Ccn1 | 2:251529353-251532312 | 2.67E-09 | 5.65 | 2.25E-06 |
| ENSRNOG00000023546.5 | Hspb1 | 12:23839398-23841049 | 2.62E-09 | 2.85 | 2.25E-06 |
| ENSRNOG00000003977.5 | Dusp1 | 10:16970625-16973418 | 3.09E-09 | 5.46 | 2.48E-06 |
| ENSRNOG00000031506.4 | Ftl1 | 5:152325870-152326778 | 3.09E-09 | 2.74 | 2.48E-06 |
| ENSRNOG00000017783.6 | Sfrp1 | 16:73372006-73410777 | 3.25E-09 | 3.52 | 2.55E-06 |
| ENSRNOG00000051238.1 | Mphosph8 | 15:36918842-36946708 | 3.89E-09 | -2.87 | 2.98E-06 |
| ENSRNOG00000021269.5 | Chgb | 3:125428259-125441594 | 5.20E-09 | 4.86 | 3.89E-06 |
| ENSRNOG00000033152.3 | Rps18l1 | 10:104521699-104522241 | 5.81E-09 | 3.47 | 4.25E-06 |
| ENSRNOG00000025889.5 | Gnas | 3:172374956-172428483 | 6.67E-09 | 4.59 | 4.77E-06 |
| ENSRNOG00000020254.7 | Per2 | 9:98555168-98597359 | 7.77E-09 | -5.01 | 5.44E-06 |
| ENSRNOG00000052549.1 | Chga | 6:126434225-126445568 | 8.12E-09 | 4.12 | 5.56E-06 |
| ENSRNOG00000031641.4 | Rpl35a | 5:164923301-164923634 | 8.49E-09 | 42.78 | 5.70E-06 |
| ENSRNOG00000020644.4 | Nsg2 | 10:15867886-15928169 | 9.59E-09 | 4.01 | 6.31E-06 |
| ENSRNOG00000020298.6 | Bag3 | 1:199941160-199965191 | 1.16E-08 | 4.20 | 7.50E-06 |
| ENSRNOG00000033299.3 | Mt-atp8 | MT:7757-7961 | 1.21E-08 | -4.08 | 7.65E-06 |
| ENSRNOG00000025587.4 | Plagl1 | 1:7252348-7259035 | 1.29E-08 | 5.04 | 8.03E-06 |
| ENSRNOG00000014338.6 | Slc25a25 | 3:11442396-11452529 | 1.68E-08 | 3.89 | 1.02E-05 |
| ENSRNOG00000011668.6 | Nfil3 | 17:12261101-12276315 | 2.39E-08 | 4.94 | 1.43E-05 |
| ENSRNOG00000051286.2 | LOC100911516 | 9:101106891-101107575 | 2.47E-08 | 7.28 | 1.45E-05 |
| ENSRNOG00000003515.6 | Ephx1 | 13:99271365-99300579 | 2.57E-08 | -2.78 | 1.49E-05 |
| ENSRNOG00000020410.5 | Th | 1:216073030-216080287 | 2.91E-08 | 3.60 | 1.65E-05 |
| ENSRNOG00000062289.1 | AABR07055846.2 | 7:12664046-12665625 | 3.09E-08 | 10.69 | 1.72E-05 |
| ENSRNOG00000030447.5 | AC105662.1 | 5:173148883-173149276 | 3.59E-08 | 11.24 | 1.97E-05 |
| ENSRNOG00000006604.3 | Thy1 | 8:48382120-48386935 | 3.67E-08 | 3.40 | 1.98E-05 |
| ENSRNOG00000008487.7 | Amotl2 | 8:111209907-111226226 | 4.02E-08 | 3.13 | 2.13E-05 |
| ENSRNOG00000054344.1 | Ier2 | 19:25774142-25775659 | 4.76E-08 | 2.53 | 2.45E-05 |
| ENSRNOG00000005359.7 | Csrnp3 | 3:51883558-52120290 | 4.93E-08 | 16.91 | 2.49E-05 |
| ENSRNOG00000020763.8 | Snrnp70 | 1:101367470-101388129 | 5.19E-08 | -2.01 | 2.58E-05 |
| ENSRNOG00000059720.1 | Syp | X:15695565-15707436 | 6.89E-08 | 4.35 | 3.38E-05 |
| ENSRNOG00000046996.1 | Pea15 | 13:90579845-90589466 | 7.73E-08 | 2.62 | 3.74E-05 |
| ENSRNOG00000012847.5 | Scgb1c1 | 1:213595239-213596459 | 8.16E-08 | 5.89 | 3.89E-05 |
| ENSRNOG00000001889.7 | Comt | 11:86715980-86735622 | 1.14E-07 | 2.42 | 5.37E-05 |
| ENSRNOG00000024349.6 | Cbarp | 7:12433932-12441048 | 1.21E-07 | 5.12 | 5.61E-05 |
| ENSRNOG00000014900.8 | Crem | 17:57031765-57090888 | 1.31E-07 | 3.64 | 6.00E-05 |
| ENSRNOG00000025235.6 | Tmem130 | 12:11814070-11840100 | 1.65E-07 | 4.42 | 7.44E-05 |
| ENSRNOG00000016299.3 | Klf4 | 5:72283312-72287669 | 1.70E-07 | 5.39 | 7.54E-05 |
| ENSRNOG00000021314.4 | Fdft1 | 15:46339248-46367302 | 1.72E-07 | -2.72 | 7.54E-05 |
| ENSRNOG00000046667.2 | Fosb | 1:80214493-80221710 | 2.06E-07 | 14.73 | 8.90E-05 |
| ENSRNOG00000051831.1 | 5_8S_rRNA | 1:11906440-11906593 | 2.16E-07 | -20.38 | 9.21E-05 |
| ENSRNOG00000014205.2 | Klf2 | 16:19223086-19225037 | 2.39E-07 | 2.77 | 9.99E-05 |
| ENSRNOG00000046382.2 | Gatd1 | 1:214389254-214394411 | 2.42E-07 | -37.67 | 9.99E-05 |
| ENSRNOG00000057347.1 | Cebpb | 3:164424514-164425910 | 2.43E-07 | 2.48 | 9.99E-05 |
| ENSRNOG00000050869.2 | Cebpd | 11:89008007-89009146 | 2.58E-07 | 3.44 | 1.05E-04 |
| ENSRNOG00000005700.7 | Nsg1 | 14:77380261-77401996 | 2.73E-07 | 4.02 | 1.09E-04 |
| ENSRNOG00000037673.3 | AABR07071891.1 | 1:84573495-84573959 | 2.94E-07 | -2.67 | 1.16E-04 |
| ENSRNOG00000031851.5 | Ndufa4l2 | 7:70821956-70824085 | 3.12E-07 | 3.78 | 1.22E-04 |
| ENSRNOG00000002343.7 | Uchl1 | 14:43133217-43143973 | 3.17E-07 | 3.58 | 1.23E-04 |
| ENSRNOG00000008001.7 | Rab3b | 5:128501846-128568188 | 3.32E-07 | 3.68 | 1.25E-04 |
| ENSRNOG00000016460.7 | Clu | 15:42640145-42665857 | 3.28E-07 | 2.30 | 1.25E-04 |
| ENSRNOG00000050006.1 | Agtr2 | X:119390012-119393842 | 3.95E-07 | 2.79 | 1.48E-04 |
| ENSRNOG00000000521.4 | Cdkn1a | 20:6351457-6358864 | 4.18E-07 | 5.03 | 1.53E-04 |
| ENSRNOG00000019704.3 | Resp18 | 9:82470758-82477181 | 4.15E-07 | 3.78 | 1.53E-04 |
| ENSRNOG00000019556.7 | Cd9 | 4:157977161-158010166 | 4.33E-07 | 2.33 | 1.57E-04 |
| ENSRNOG00000015514.7 | Bcat1 | 4:179259307-179339795 | 4.40E-07 | 3.62 | 1.57E-04 |
| ENSRNOG00000007544.5 | Il23r | 4:98203957-98305173 | 4.55E-07 | 6.56 | 1.61E-04 |
| ENSRNOG00000004284.4 | Btg1 | 7:37812830-37815088 | 5.51E-07 | 2.93 | 1.93E-04 |
| ENSRNOG00000003897.6 | Col1a1 | 10:82745800-82762789 | 6.75E-07 | 2.70 | 2.33E-04 |
| ENSRNOG00000007808.3 | Nap1l5 | 4:89149316-89151184 | 6.81E-07 | 4.28 | 2.33E-04 |
| ENSRNOG00000019383.7 | Tef | 7:123043502-123058604 | 7.24E-07 | -2.85 | 2.46E-04 |
| ENSRNOG00000058470.1 | Col12a1 | 8:87040798-87158368 | 7.53E-07 | 3.18 | 2.53E-04 |
| ENSRNOG00000001128.6 | Tesc | 12:44141421-44174583 | 7.70E-07 | 2.68 | 2.56E-04 |
| ENSRNOG00000015055.6 | Scg2 | 9:85237457-85243001 | 8.44E-07 | 3.89 | 2.77E-04 |
| ENSRNOG00000007393.7 | Ndrg1 | 7:107734322-107775714 | 9.79E-07 | 3.11 | 3.19E-04 |
| ENSRNOG00000012477.4 | Eef1a2 | 3:176657106-176666282 | 1.05E-06 | 4.12 | 3.40E-04 |
| ENSRNOG00000030291.4 | AC095947.1 | 10:70100660-70101169 | 1.29E-06 | -2.14 | 4.09E-04 |
| ENSRNOG00000043451.4 | Spp1 | 14:6673685-6679901 | 1.48E-06 | 7.43 | 4.64E-04 |
| ENSRNOG00000021812.4 | Scx | 7:117519074-117521093 | 1.58E-06 | 6.09 | 4.90E-04 |
| ENSRNOG00000011992.4 | Slc18a1 | 16:22361997-22395183 | 1.68E-06 | 3.58 | 5.17E-04 |
| ENSRNOG00000010872.5 | CTb | 6:136142955-136145837 | 1.74E-06 | 2.02 | 5.26E-04 |
| ENSRNOG00000058183.1 | AABR07040624.1 | X:106607046-106607352 | 1.78E-06 | -2.82 | 5.31E-04 |
| ENSRNOG00000015434.7 | Midn | 7:12414732-12424367 | 1.81E-06 | 2.32 | 5.36E-04 |
| ENSRNOG00000031244.3 | LOC108349606 | 18:4371050-4371899 | 1.88E-06 | -2.04 | 5.53E-04 |
| ENSRNOG00000028155.6 | Dnaaf2 | 6:91481953-91490366 | 1.92E-06 | 6.58 | 5.58E-04 |
| ENSRNOG00000000957.5 | Rpl21 | 12:9996950-9998779 | 2.67E-06 | 2.16 | 7.51E-04 |
| ENSRNOG00000024923.6 | Nnat | 3:154043872-154046330 | 2.66E-06 | 3.96 | 7.51E-04 |
| ENSRNOG00000007542.6 | Scg5 | 3:105235049-105279462 | 3.69E-06 | 3.31 | 1.03E-03 |
| ENSRNOG00000018214.6 | Bok | 9:100829551-100840504 | 3.83E-06 | 2.08 | 1.06E-03 |
| ENSRNOG00000000827.6 | Ier3 | 20:3438797-3440769 | 4.06E-06 | -2.63 | 1.11E-03 |
| ENSRNOG00000001030.8 | Tsc22d1 | 15:58554373-58658153 | 4.25E-06 | 2.15 | 1.15E-03 |
| ENSRNOG00000004091.6 | Cwc25 | 10:85684897-85709568 | 5.54E-06 | 4.36 | 1.47E-03 |
| ENSRNOG00000050786.2 | AC103024.2 | 10:34316836-34317161 | 5.79E-06 | -17.63 | 1.51E-03 |
| ENSRNOG00000027030.1 | Adm | 1:175445087-175447259 | 6.39E-06 | 3.65 | 1.62E-03 |
| ENSRNOG00000016924.8 | Acly | 10:88392247-88442845 | 6.79E-06 | -2.10 | 1.70E-03 |
| ENSRNOG00000033581.5 | AABR07044388.2 | 20:4436418-4486680 | 7.61E-06 | -3.26 | 1.89E-03 |
| ENSRNOG00000006539.7 | Pex16 | 3:81283136-81292573 | 8.02E-06 | -2.16 | 1.97E-03 |
| ENSRNOG00000032297.2 | Msmo1 | 16:26859396-26875973 | 8.41E-06 | -2.72 | 2.05E-03 |
| ENSRNOG00000016220.7 | Rpl12 | 3:12009577-12011666 | 8.60E-06 | 2.15 | 2.08E-03 |
| ENSRNOG00000004870.5 | Dyrk3 | 13:47906230-47916877 | 8.92E-06 | 3.23 | 2.13E-03 |
| ENSRNOG00000039249.3 | AABR07035539.1 | 12:19159467-19159901 | 9.14E-06 | -4.90 | 2.13E-03 |
| ENSRNOG00000045654.2 | LOC108348108 | 20:2699711-2701815 | 8.99E-06 | 16.44 | 2.13E-03 |
| ENSRNOG00000046484.1 | Ccdc160 | X:140175860-140176829 | 9.11E-06 | -2.35 | 2.13E-03 |
| ENSRNOG00000056457.1 | Gpd1 | 7:141370490-141377928 | 9.14E-06 | -2.43 | 2.13E-03 |
| ENSRNOG00000007607.4 | Nr4a1 | 7:142905757-142920216 | 9.42E-06 | 2.34 | 2.18E-03 |
| ENSRNOG00000001469.8 | Eln | 12:24978482-25021863 | 9.50E-06 | 4.12 | 2.18E-03 |
| ENSRNOG00000011292.6 | NEWGENE_621351 | 4:31534224-31569151 | 9.96E-06 | 2.63 | 2.27E-03 |
| ENSRNOG00000018184.8 | Tpm1 | 8:72814739-72842228 | 1.08E-05 | 2.13 | 2.46E-03 |
| ENSRNOG00000006787.6 | Dhcr24 | 5:126164673-126191206 | 1.10E-05 | -2.40 | 2.47E-03 |
| ENSRNOG00000053207.1 | AABR07026125.1 | 16:64982381-64984767 | 1.13E-05 | 4.09 | 2.54E-03 |
| ENSRNOG00000019622.3 | ACTr3 | 9:97355923-97367445 | 1.25E-05 | 4.54 | 2.76E-03 |
| ENSRNOG00000051563.1 | Giot1 | 7:13104480-13108723 | 1.27E-05 | 4.41 | 2.79E-03 |
| ENSRNOG00000017209.6 | Tubb3 | 19:56220754-56229813 | 1.35E-05 | 2.56 | 2.93E-03 |
| ENSRNOG00000008376.6 | LOC100909595 | 4:155408232-155631856 | 1.58E-05 | 3.42 | 3.39E-03 |
| ENSRNOG00000031706.2 | RGD1563601 | 17:27765007-27765754 | 1.66E-05 | -2.38 | 3.54E-03 |
| ENSRNOG00000009311.6 | Fstl3 | 7:12804918-12810570 | 1.67E-05 | 2.79 | 3.55E-03 |
| ENSRNOG00000031216.3 | AABR07055776.1 | 7:9974624-9976367 | 1.71E-05 | 3.49 | 3.58E-03 |
| ENSRNOG00000011946.7 | Ptn | 4:64239157-64330996 | 1.74E-05 | 2.31 | 3.63E-03 |
| ENSRNOG00000007433.8 | Cyb561 | 10:94136992-94147621 | 1.82E-05 | 3.12 | 3.74E-03 |
| ENSRNOG00000058388.2 | Zfp36 | 1:85380087-85382569 | 1.83E-05 | 2.45 | 3.74E-03 |
| ENSRNOG00000019018.6 | Plat | 16:74098259-74122889 | 1.97E-05 | 3.23 | 3.98E-03 |
| ENSRNOG00000004554.6 | Dcn | 7:38742050-38782323 | 2.76E-05 | 2.08 | 5.46E-03 |
| ENSRNOG00000024578.7 | Ttyh2 | 10:103206013-103248862 | 2.86E-05 | -2.39 | 5.64E-03 |
| ENSRNOG00000007152.2 | Bhlhe40 | 4:140703618-140709319 | 3.08E-05 | -2.57 | 5.92E-03 |
| ENSRNOG00000002911.5 | Alb | 14:19176276-19191863 | 3.22E-05 | -7.70 | 6.15E-03 |
| ENSRNOG00000007793.6 | Pnrc1 | 5:48501471-48504511 | 3.26E-05 | 2.11 | 6.20E-03 |
| ENSRNOG00000052880.1 | Prph | 7:140742417-140746197 | 3.48E-05 | 3.78 | 6.57E-03 |
| ENSRNOG00000011705.7 | Stmn2 | 2:95424250-95472115 | 3.50E-05 | 3.45 | 6.58E-03 |
| ENSRNOG00000038045.4 | AC128960.1 | 17:78876110-78876537 | 3.63E-05 | -2.13 | 6.79E-03 |
| ENSRNOG00000005660.4 | Fam110c | 6:49968472-49974728 | 3.76E-05 | 2.68 | 6.98E-03 |
| ENSRNOG00000013011.7 | Dnajb4 | 2:257394817-257425242 | 3.81E-05 | 2.14 | 6.99E-03 |
| ENSRNOG00000012410.4 | S100a1 | 2:189900666-189903219 | 3.93E-05 | 3.18 | 7.16E-03 |
| ENSRNOG00000010146.6 | Ndn | 1:122981754-122983351 | 4.22E-05 | 2.20 | 7.62E-03 |
| ENSRNOG00000004794.8 | Rtn1 | 6:94951019-95061174 | 4.25E-05 | 3.79 | 7.64E-03 |
| ENSRNOG00000018413.7 | Per3 | 5:168086997-168123395 | 4.30E-05 | -4.60 | 7.69E-03 |
| ENSRNOG00000021719.7 | Slfn5 | 10:70298518-70312070 | 4.38E-05 | -2.05 | 7.78E-03 |
| ENSRNOG00000000902.7 | Hsph1 | 12:6322667-6341902 | 4.82E-05 | 2.52 | 8.52E-03 |
| ENSRNOG00000000490.7 | Rps10 | 20:7215773-7219548 | 4.94E-05 | 2.00 | 8.57E-03 |
| ENSRNOG00000010873.7 | Pithd1 | 5:154322550-154332940 | 4.91E-05 | 2.60 | 8.57E-03 |
| ENSRNOG00000016311.7 | Slc6a2 | 19:15391580-15431274 | 4.98E-05 | 6.47 | 8.57E-03 |
| ENSRNOG00000019587.6 | Ptprn | 9:82446632-82461903 | 4.95E-05 | 2.87 | 8.57E-03 |
| ENSRNOG00000055188.1 | Tlx2 | 4:113900555-113902399 | 4.98E-05 | 5.76 | 8.57E-03 |
| ENSRNOG00000049693.2 | LOC108348130 | 11:33845462-33847793 | 5.11E-05 | 331.39 | 8.75E-03 |
| ENSRNOG00000032729.3 | Bex2 | X:106556837-106558366 | 5.20E-05 | 2.94 | 8.86E-03 |
| ENSRNOG00000009715.6 | Me1 | 8:94256838-94368834 | 5.51E-05 | -2.19 | 9.33E-03 |
| ENSRNOG00000062158.1 | AC134224.3 | 1:221158097-221158792 | 5.74E-05 | -4.25 | 9.67E-03 |
| ENSRNOG00000016343.7 | Dkk3 | 1:176940692-176983045 | 5.86E-05 | 3.36 | 9.83E-03 |
| ENSRNOG00000060448.1 | Hand2 | 16:36371488-36373546 | 6.13E-05 | 3.30 | 1.01E-02 |
| ENSRNOG00000010408.7 | Polr2k | 7:74989222-74992582 | 6.58E-05 | -2.09 | 1.08E-02 |
| ENSRNOG00000000875.8 | Fhl1 | X:159112879-159172524 | 6.79E-05 | 2.41 | 1.11E-02 |
| ENSRNOG00000017333.5 | Syt4 | 18:24172586-24182012 | 7.07E-05 | 4.41 | 1.15E-02 |
| ENSRNOG00000013165.4 | Bet1l | 1:213602849-213605643 | 7.36E-05 | -2.28 | 1.19E-02 |
| ENSRNOG00000001518.7 | Itga6 | 3:58443100-58515124 | 7.75E-05 | 2.78 | 1.24E-02 |
| ENSRNOG00000059883.1 | LOC100912578 | 3:74226406-74229066 | 8.26E-05 | 2.21 | 1.31E-02 |
| ENSRNOG00000047790.2 | LOC108348055 | 10:47785032-47792590 | 8.89E-05 | 7.15 | 1.41E-02 |
| ENSRNOG00000059764.1 | Snrpn | 1:195074329-195096460 | 9.01E-05 | 2.62 | 1.42E-02 |
| ENSRNOG00000056427.1 | AABR07007717.1 | 2:25564883-25565757 | 1.16E-04 | 3.16 | 1.79E-02 |
| ENSRNOG00000058646.1 | Zfp36l1 | 6:103308044-103313074 | 1.20E-04 | 2.26 | 1.84E-02 |
| ENSRNOG00000010716.3 | Atoh8 | 4:100067340-100099517 | 1.21E-04 | 3.10 | 1.84E-02 |
| ENSRNOG00000056028.1 | Rfxap | 2:144003453-144007636 | 1.27E-04 | -2.98 | 1.93E-02 |
| ENSRNOG00000057823.1 | Ubc | 12:36638456-36642734 | 1.30E-04 | 2.28 | 1.96E-02 |
| ENSRNOG00000006641.8 | Dbh | 3:5709235-5731898 | 1.37E-04 | 3.32 | 2.04E-02 |
| ENSRNOG00000026787.5 | Disp2 | 3:110574416-110589921 | 1.45E-04 | 3.06 | 2.12E-02 |
| ENSRNOG00000004019.3 | Phlda1 | 7:54247459-54249664 | 1.50E-04 | 2.42 | 2.15E-02 |
| ENSRNOG00000008365.5 | Hoxb2 | 10:84200879-84202688 | 1.50E-04 | 2.24 | 2.15E-02 |
| ENSRNOG00000033979.3 | Gm5471 | 5:21043449-21043791 | 1.50E-04 | -5.28 | 2.15E-02 |
| ENSRNOG00000058882.1 | Emd | X:156452817-156456035 | 1.50E-04 | 2.33 | 2.15E-02 |
| ENSRNOG00000050792.2 | Tnfaip6 | 3:37545237-37564699 | 1.52E-04 | 3.30 | 2.16E-02 |
| ENSRNOG00000026124.5 | Id3 | 5:154489589-154491223 | 1.54E-04 | 2.17 | 2.17E-02 |
| ENSRNOG00000061056.1 | AABR07042514.3 | X:158581630-158582269 | 1.55E-04 | 2.59 | 2.18E-02 |
| ENSRNOG00000027008.6 | Igtp | 10:43613930-43653390 | 1.59E-04 | -2.76 | 2.22E-02 |
| ENSRNOG00000018337.6 | Caly | 1:212537847-212549477 | 1.62E-04 | 3.35 | 2.25E-02 |
| ENSRNOG00000016281.5 | Col4a1 | 16:83522161-83632153 | 1.66E-04 | 2.00 | 2.31E-02 |
| ENSRNOG00000017628.6 | Tagln | 8:50222895-50231357 | 1.81E-04 | 2.14 | 2.48E-02 |
| ENSRNOG00000008758.5 | Tspan18 | 3:82097067-82236664 | 1.83E-04 | 2.74 | 2.49E-02 |
| ENSRNOG00000030345.4 | LOC100362684 | 9:1012090-1012450 | 1.85E-04 | -5.98 | 2.49E-02 |
| ENSRNOG00000002793.5 | Sstr2 | 10:102136282-102143434 | 1.90E-04 | 2.49 | 2.54E-02 |
| ENSRNOG00000031273.3 | AABR07034639.1 | 11:82366073-82366505 | 1.94E-04 | 4.05 | 2.58E-02 |
| ENSRNOG00000043387.3 | Cpe | 16:26906715-27014813 | 1.96E-04 | 2.70 | 2.59E-02 |
| ENSRNOG00000000529.5 | Pim1 | 20:8165306-8169555 | 2.02E-04 | -3.24 | 2.66E-02 |
| ENSRNOG00000031579.2 | LOC100363469 | 10:78992579-78993045 | 2.02E-04 | 2.97 | 2.66E-02 |
| ENSRNOG00000018358.4 | Nt5dc2 | 16:7212487-7220540 | 2.39E-04 | 2.54 | 3.11E-02 |
| ENSRNOG00000023509.6 | Irs2 | 16:83824429-83848684 | 2.39E-04 | 2.67 | 3.11E-02 |
| ENSRNOG00000004327.8 | Ddc | 14:91905918-91996774 | 2.46E-04 | 2.77 | 3.18E-02 |
| ENSRNOG00000059827.1 | LOC691995 | 10:57669529-57671080 | 2.51E-04 | 3.19 | 3.24E-02 |
| ENSRNOG00000019973.6 | Arl3 | 1:266287016-266333105 | 2.72E-04 | 2.04 | 3.45E-02 |
| ENSRNOG00000052421.1 | AABR07012475.2 | 2:196587205-196591324 | 2.76E-04 | -3.71 | 3.46E-02 |
| ENSRNOG00000015093.6 | Sparcl1 | 14:6994189-7025308 | 2.78E-04 | 2.61 | 3.48E-02 |
| ENSRNOG00000034066.5 | Hspa8 | 8:44990013-44993179 | 2.95E-04 | 3.33 | 3.68E-02 |
| ENSRNOG00000042152.3 | Gipc2 | 2:257293166-257376756 | 3.09E-04 | 2.52 | 3.82E-02 |
| ENSRNOG00000020871.7 | Ltbp4 | 1:84118045-84150084 | 3.14E-04 | 2.53 | 3.84E-02 |
| ENSRNOG00000006037.8 | Snap25 | 3:129599352-129788400 | 3.28E-04 | 3.68 | 3.98E-02 |
| ENSRNOG00000000195.7 | LOC108348048 | 8:42443441-42466907 | 3.51E-04 | 2.50 | 4.17E-02 |
| ENSRNOG00000003580.4 | Ucp1 | 19:24456975-24464807 | 3.49E-04 | -11.33 | 4.17E-02 |
| ENSRNOG00000001989.6 | Alcam | 11:50781126-50985083 | 3.55E-04 | 2.66 | 4.19E-02 |
| ENSRNOG00000029698.5 | Pim3 | 7:129860113-129863441 | 3.66E-04 | 2.30 | 4.30E-02 |
| ENSRNOG00000002773.2 | Rgs4 | 13:88054816-88061108 | 3.83E-04 | 2.12 | 4.47E-02 |
| ENSRNOG00000001198.4 | Fam222a | 12:47748164-47793534 | 3.96E-04 | 2.26 | 4.56E-02 |
| ENSRNOG00000003171.7 | Mpz | 13:89524328-89530068 | 4.05E-04 | 6.75 | 4.63E-02 |
| ENSRNOG00000004936.6 | Sdc2 | 7:71572940-71686044 | 4.08E-04 | 2.07 | 4.64E-02 |
| ENSRNOG00000019244.6 | Mxra8 | 5:173288446-173292929 | 4.44E-04 | 2.48 | 4.97E-02 |
| ENSRNOG00000036802.4 | Snhg11 | 3:154863071-154869460 | 4.40E-04 | -2.52 | 4.97E-02 |

* FDR: false discovery rate. The FDR-adjusted P value was sorted from lowest to highest.

Table S8. Statistics of differentially expressed genes (DEG) with *P* < 0.01, FDR-adjusted P value = 0.05 and |Fold change| > 2 in adrenal glands in comparison of CT vs. H60.

| Feature ID | Gene name | Position | P-value | Fold change | FDR-adjusted P value * |
| --- | --- | --- | --- | --- | --- |
| ENSRNOG00000033299.3 | Mt-atp8 | MT:7757-7961 | 0.00E+00 | -7.46 | 0.00E+00 |
| ENSRNOG00000021824.5 | Dnajb1 | 19:24747177-24750919 | 6.7E-64 | 50.57 | 1.1E-59 |
| ENSRNOG00000020298.6 | Bag3 | 1:199941160-199965191 | 1.87E-51 | 35.79 | 2.05E-47 |
| ENSRNOG00000027089.6 | Ell2 | 2:2456734-2524237 | 8.15E-44 | 7.68 | 6.7E-40 |
| ENSRNOG00000032917.3 | Zfand2a | 12:17252092-17263477 | 1.52E-41 | 42.20 | 8.5E-38 |
| ENSRNOG00000050647.2 | Hspa1b | 20:4877323-4879779 | 1.55E-41 | 182.12 | 8.5E-38 |
| ENSRNOG00000023546.5 | Hspb1 | 12:23839398-23841049 | 2.41E-40 | 20.37 | 1.13E-36 |
| ENSRNOG00000045654.2 | LOC108348108 | 20:2699711-2701815 | 2.4E-39 | 104.09 | 9.86E-36 |
| ENSRNOG00000013011.7 | Dnajb4 | 2:257394817-257425242 | 4.86E-39 | 6.85 | 1.78E-35 |
| ENSRNOG00000000902.7 | Hsph1 | 12:6322667-6341902 | 6.98E-38 | 22.35 | 2.29E-34 |
| ENSRNOG00000004091.6 | Cwc25 | 10:85684897-85709568 | 1.76E-37 | 12.20 | 5.27E-34 |
| ENSRNOG00000014900.8 | Crem | 17:57031765-57090888 | 2.41E-35 | 6.19 | 6.61E-32 |
| ENSRNOG00000007029.3 | Dnaja1 | 5:57028466-57039378 | 2.68E-35 | 9.43 | 6.77E-32 |
| ENSRNOG00000003687.6 | Rgs2 | 13:60846307-60849094 | 1.31E-34 | 15.08 | 3.08E-31 |
| ENSRNOG00000046112.2 | Dedd2 | 1:82072543-82088275 | 6.46E-34 | 7.61 | 1.42E-30 |
| ENSRNOG00000059714.1 | Hsp90aa1 | 6:135107270-135112775 | 1.44E-33 | 9.56 | 2.95E-30 |
| ENSRNOG00000014456.6 | Coq10b | 9:61655962-61675873 | 2.96E-32 | 2.96 | 5.73E-29 |
| ENSRNOG00000001189.6 | Sik1 | 20:10668410-10680283 | 1.36E-31 | 11.74 | 2.49E-28 |
| ENSRNOG00000028155.6 | Dnaaf2 | 6:91481953-91490366 | 1.33E-30 | 19.77 | 2.22E-27 |
| ENSRNOG00000051563.1 | Giot1 | 7:13104480-13108723 | 1.35E-30 | 15.75 | 2.22E-27 |
| ENSRNOG00000009920.5 | LOC680121 | 11:13499163-13501263 | 2.69E-30 | 5.44 | 4.21E-27 |
| ENSRNOG00000056651.1 | LOC103690068 | 1:88192369-88193346 | 4.48E-30 | 13.68 | 6.69E-27 |
| ENSRNOG00000031506.4 | Ftl1 | 5:152325870-152326778 | 1.21E-29 | 3.69 | 1.73E-26 |
| ENSRNOG00000057823.1 | Ubc | 12:36638456-36642734 | 3.11E-29 | 7.34 | 4.26E-26 |
| ENSRNOG00000002572.5 | Cacybp | 13:77948770-77959110 | 7.76E-29 | 4.52 | 1.02E-25 |
| ENSRNOG00000055564.1 | RGD1564664 | 3:82854776-82856171 | 9.84E-29 | 8.93 | 1.24E-25 |
| ENSRNOG00000033024.2 | AC099453.1 | 5:78392714-78393143 | 1.34E-28 | -8.12 | 1.63E-25 |
| ENSRNOG00000028690.3 | LOC100362366 | 3:24600602-24601063 | 6.82E-28 | -6.09 | 8.01E-25 |
| ENSRNOG00000015160.5 | Gem | 5:25349927-25353661 | 2.37E-26 | 9.56 | 2.69E-23 |
| ENSRNOG00000004019.3 | Phlda1 | 7:54247459-54249664 | 6.41E-26 | 7.48 | 7.03E-23 |
| ENSRNOG00000012106.4 | Dnaja4 | 8:59278261-59294003 | 8.16E-26 | 16.65 | 8.66E-23 |
| ENSRNOG00000037352.3 | RGD1562747 | 8:69484173-69489891 | 1.2E-25 | 10.42 | 1.23E-22 |
| ENSRNOG00000052142.1 | Ahsa2 | 14:108265206-108273938 | 6.89E-24 | 6.81 | 6.86E-21 |
| ENSRNOG00000022392.3 | Hspb8 | 12:45905370-45920013 | 1.28E-23 | 7.88 | 1.24E-20 |
| ENSRNOG00000034066.5 | Hspa8 | 8:44990013-44993179 | 2.75E-23 | 13.49 | 2.58E-20 |
| ENSRNOG00000003597.5 | Tuba4a | 9:82415604-82419288 | 3.65E-23 | 5.19 | 3.34E-20 |
| ENSRNOG00000048199.2 | Rps19l2 | 9:16802318-16807966 | 6.3E-23 | 17.74 | 5.6E-20 |
| ENSRNOG00000011771.3 | Ptp4a1 | 9:37223728-37231291 | 1.64E-22 | 4.57 | 1.42E-19 |
| ENSRNOG00000019018.6 | Plat | 16:74098259-74122889 | 3.96E-22 | 8.67 | 3.34E-19 |
| ENSRNOG00000005660.4 | Fam110c | 6:49968472-49974728 | 9.79E-22 | 6.23 | 8.05E-19 |
| ENSRNOG00000020843.6 | LOC100360087 | 1:101448345-101449829 | 5.55E-21 | 2.95 | 4.45E-18 |
| ENSRNOG00000010873.7 | Pithd1 | 5:154322550-154332940 | 6.17E-21 | 4.23 | 4.76E-18 |
| ENSRNOG00000019622.3 | ACTr3 | 9:97355923-97367445 | 6.22E-21 | 8.34 | 4.76E-18 |
| ENSRNOG00000024349.6 | Cbarp | 7:12433932-12441048 | 1.41E-20 | 5.73 | 1.06E-17 |
| ENSRNOG00000020938.7 | Ppp1r15a | 1:101511900-101514974 | 1.48E-20 | 3.98 | 1.08E-17 |
| ENSRNOG00000000461.7 | LOC108348112 | 20:5262119-5269337 | 2.53E-20 | 4.25 | 1.81E-17 |
| ENSRNOG00000001030.8 | Tsc22d1 | 15:58554373-58658153 | 5.36E-20 | 3.03 | 3.75E-17 |
| ENSRNOG00000042838.3 | Junb | 19:26092973-26094756 | 6.81E-20 | 11.04 | 4.67E-17 |
| ENSRNOG00000042271.3 | Ubb | 10:48881048-48881881 | 1.16E-19 | 4.50 | 7.76E-17 |
| ENSRNOG00000031216.3 | AABR07055776.1 | 7:9974624-9976367 | 1.96E-19 | 4.74 | 1.29E-16 |
| ENSRNOG00000011668.6 | Nfil3 | 17:12261101-12276315 | 3.29E-19 | 8.42 | 2.12E-16 |
| ENSRNOG00000004284.4 | Btg1 | 7:37812830-37815088 | 4.25E-19 | 4.49 | 2.69E-16 |
| ENSRNOG00000062247.1 | AABR07031521.1 | 18:17116551-17118501 | 1.09E-18 | 6.46 | 6.76E-16 |
| ENSRNOG00000014338.6 | Slc25a25 | 3:11442396-11452529 | 1.78E-18 | 6.85 | 1.08E-15 |
| ENSRNOG00000046667.2 | Fosb | 1:80214493-80221710 | 3.09E-18 | 26.14 | 1.84E-15 |
| ENSRNOG00000019834.6 | Hsp90ab1 | 9:17817720-17823243 | 5.95E-18 | 3.24 | 3.49E-15 |
| ENSRNOG00000033473.5 | Rpl36 | 9:10441258-10441834 | 8.61E-18 | 2.91 | 4.97E-15 |
| ENSRNOG00000058461.1 | Sfpq | 5:145079802-145088498 | 2.2E-17 | 2.89 | 1.25E-14 |
| ENSRNOG00000011039.7 | Gch1 | 15:23934584-23969011 | 8.98E-17 | 4.06 | 5.00E-14 |
| ENSRNOG00000033803.5 | LOC100360841 | 9:69790830-69791196 | 9.42E-17 | -5.62 | 5.16E-14 |
| ENSRNOG00000050655.1 | P4ha1 | 20:28920615-28971966 | 1.01E-16 | 6.19 | 5.45E-14 |
| ENSRNOG00000008487.7 | Amotl2 | 8:111209907-111226226 | 1.09E-16 | 5.10 | 5.78E-14 |
| ENSRNOG00000012886.4 | Maff | 7:120580742-120592095 | 1.42E-16 | 6.43 | 7.40E-14 |
| ENSRNOG00000026643.5 | Chordc1 | 8:17421556-17446165 | 1.48E-16 | 5.15 | 7.58E-14 |
| ENSRNOG00000023509.6 | Irs2 | 16:83824429-83848684 | 1.54E-16 | 4.22 | 7.79E-14 |
| ENSRNOG00000005964.5 | Nr4a3 | 5:63781800-63821637 | 2.07E-16 | 4.63 | 1.03E-13 |
| ENSRNOG00000031889.4 | AABR07065438.1 | 6:128738387-128739319 | 2.71E-16 | -3.55 | 1.33E-13 |
| ENSRNOG00000016831.6 | Serpinh1 | 1:164301009-164308317 | 3.64E-16 | 2.19 | 1.76E-13 |
| ENSRNOG00000026605.5 | Ifi27l2b | 6:127336304-127337791 | 8.88E-16 | -3.12 | 4.23E-13 |
| ENSRNOG00000020480.5 | Fads1 | 1:226234073-226249138 | 2.08E-15 | -3.34 | 9.75E-13 |
| ENSRNOG00000019466.7 | Agpat2 | 3:4044541-4055806 | 2.35E-15 | -3.35 | 1.09E-12 |
| ENSRNOG00000058882.1 | Emd | X:156452817-156456035 | 2.69E-15 | 3.77 | 1.23E-12 |
| ENSRNOG00000001198.4 | Fam222a | 12:47748164-47793534 | 2.82E-15 | 4.19 | 1.27E-12 |
| ENSRNOG00000017801.7 | Atf4 | 7:121480722-121482772 | 3.38E-15 | 3.16 | 1.50E-12 |
| ENSRNOG00000058646.1 | Zfp36l1 | 6:103308044-103313074 | 8.32E-15 | 3.24 | 3.65E-12 |
| ENSRNOG00000005615.6 | Gadd45a | 4:97782511-97784842 | 1.52E-14 | 9.96 | 6.57E-12 |
| ENSRNOG00000021475.6 | Ldah | 6:33407247-33491725 | 1.95E-14 | 6.64 | 8.32E-12 |
| ENSRNOG00000010706.4 | Ccdc117 | 14:85763396-85772762 | 2.12E-14 | 4.37 | 8.95E-12 |
| ENSRNOG00000009358.6 | Osgin2 | 5:29663684-29682005 | 2.5E-14 | 4.27 | 1.03E-11 |
| ENSRNOG00000016299.3 | Klf4 | 5:72283312-72287669 | 2.48E-14 | 6.44 | 1.03E-11 |
| ENSRNOG00000004084.7 | Lratd1 | 6:39358984-39363458 | 4.08E-14 | 24.71 | 1.66E-11 |
| ENSRNOG00000025587.4 | Plagl1 | 1:7252348-7259035 | 4.5E-14 | 7.76 | 1.81E-11 |
| ENSRNOG00000062158.1 | AC134224.3 | 1:221158097-221158792 | 5.33E-14 | -11.43 | 2.11E-11 |
| ENSRNOG00000015434.7 | Midn | 7:12414732-12424367 | 6.4E-14 | 2.85 | 2.51E-11 |
| ENSRNOG00000034093.4 | AABR07011951.1 | 2:177651240-177653288 | 7.01E-14 | 3.68 | 2.71E-11 |
| ENSRNOG00000009768.6 | LOC100912228 | 4:79557853-79565097 | 9.48E-14 | 21.01 | 3.63E-11 |
| ENSRNOG00000050792.2 | Tnfaip6 | 3:37545237-37564699 | 9.71E-14 | 5.71 | 3.67E-11 |
| ENSRNOG00000046484.1 | Ccdc160 | X:140175860-140176829 | 2.07E-13 | -5.41 | 7.74E-11 |
| ENSRNOG00000000661.5 | Hps4 | 12:50285277-50314406 | 2.44E-13 | 4.79 | 9.03E-11 |
| ENSRNOG00000011238.6 | Tiparp | 2:157316265-157341469 | 1.07E-12 | 8.34 | 3.89E-10 |
| ENSRNOG00000021729.6 | Iqub | 4:51482169-51586881 | 1.5E-12 | 47.97 | 5.43E-10 |
| ENSRNOG00000009754.6 | Nampt | 6:52122084-52156472 | 1.54E-12 | 3.22 | 5.47E-10 |
| ENSRNOG00000016930.5 | Ube2s | 1:72580423-72584482 | 1.55E-12 | 2.08 | 5.47E-10 |
| ENSRNOG00000000918.7 | Zbed5 | 12:30308468-30314519 | 2.23E-12 | 2.59 | 7.81E-10 |
| ENSRNOG00000000909.8 | Uspl1 | 12:6929977-6956984 | 2.73E-12 | 3.85 | 9.44E-10 |
| ENSRNOG00000012872.7 | Carnmt1 | 1:234677691-234704792 | 2.94E-12 | 4.00 | 1.01E-09 |
| ENSRNOG00000007393.7 | Ndrg1 | 7:107734322-107775714 | 3.32E-12 | 5.12 | 1.13E-09 |
| ENSRNOG00000012559.6 | Man1b1 | 3:2547985-2569049 | 3.37E-12 | 2.74 | 1.13E-09 |
| ENSRNOG00000014117.7 | Hmox1 | 19:14508615-14515456 | 4.74E-12 | 5.29 | 1.57E-09 |
| ENSRNOG00000006756.8 | Maged1 | X:63803188-63809861 | 4.94E-12 | 2.35 | 1.63E-09 |
| ENSRNOG00000020644.4 | Nsg2 | 10:15867886-15928169 | 5.12E-12 | 3.89 | 1.67E-09 |
| ENSRNOG00000002393.7 | Eprs | 13:103300931-103371577 | 5.59E-12 | 3.57 | 1.78E-09 |
| ENSRNOG00000013328.8 | Rbpms | 16:61954589-62096497 | 5.54E-12 | 2.37 | 1.78E-09 |
| ENSRNOG00000005413.5 | Creb3l1 | 3:80892434-80933283 | 6.76E-12 | 14.80 | 2.14E-09 |
| ENSRNOG00000003745.3 | Atf3 | 13:109817727-109849632 | 7.27E-12 | 15.28 | 2.28E-09 |
| ENSRNOG00000004726.7 | Mapkapk2 | 13:47785160-47831110 | 8.27E-12 | 3.38 | 2.54E-09 |
| ENSRNOG00000014743.5 | Hagh | 10:14216154-14230515 | 1.47E-11 | 3.34 | 4.47E-09 |
| ENSRNOG00000019584.6 | Dlk1 | 6:133552820-133583751 | 1.55E-11 | 2.21 | 4.68E-09 |
| ENSRNOG00000007607.4 | Nr4a1 | 7:142905757-142920216 | 1.84E-11 | 3.08 | 5.49E-09 |
| ENSRNOG00000057078.1 | Ddit4 | 20:29509288-29511382 | 3.06E-11 | 6.69 | 9.05E-09 |
| ENSRNOG00000008015.5 | Fos | 6:109300432-109303299 | 3.37E-11 | 16.80 | 9.81E-09 |
| ENSRNOG00000019996.5 | Slc16a1 | 2:207108551-207128554 | 3.36E-11 | 5.52 | 9.81E-09 |
| ENSRNOG00000000304.7 | Cd164 | 20:46250362-46261994 | 3.98E-11 | 2.33 | 1.14E-08 |
| ENSRNOG00000005513.7 | Srsf5 | 6:104611025-104615302 | 3.98E-11 | -2.31 | 1.14E-08 |
| ENSRNOG00000017209.6 | Tubb3 | 19:56220754-56229813 | 4.45E-11 | 4.72 | 1.26E-08 |
| ENSRNOG00000037673.3 | AABR07071891.1 | 1:84573495-84573959 | 5.94E-11 | -3.04 | 1.67E-08 |
| ENSRNOG00000014525.5 | Hspd1 | 9:61680529-61690956 | 6.16E-11 | 2.02 | 1.72E-08 |
| ENSRNOG00000019822.4 | Gadd45b | 7:11646268-11648322 | 6.28E-11 | 4.80 | 1.74E-08 |
| ENSRNOG00000009205.6 | Lmo4 | 2:250218640-250241673 | 6.63E-11 | 2.54 | 1.80E-08 |
| ENSRNOG00000059017.1 | AC106292.2 | 11:65743891-65745038 | 6.59E-11 | 6.43 | 1.80E-08 |
| ENSRNOG00000059589.1 | Lmbr1 | 4:2116093-2201749 | 6.73E-11 | 6.60 | 1.81E-08 |
| ENSRNOG00000000796.8 | Ranbp2 | 20:28027053-28076664 | 6.87E-11 | 2.96 | 1.84E-08 |
| ENSRNOG00000051286.2 | LOC100911516 | 9:101106891-101107575 | 7.28E-11 | 10.52 | 1.93E-08 |
| ENSRNOG00000014350.4 | Ccn1 | 2:251529353-251532312 | 8.17E-11 | 7.76 | 2.15E-08 |
| ENSRNOG00000017212.7 | Spsb1 | 5:166913734-166926636 | 1.27E-10 | 3.80 | 3.31E-08 |
| ENSRNOG00000009734.4 | Akr1b8 | 4:61771969-61828657 | 1.3E-10 | 3.12 | 3.38E-08 |
| ENSRNOG00000021164.7 | Stip1 | 1:222274132-222293148 | 1.5E-10 | 3.00 | 3.86E-08 |
| ENSRNOG00000000250.6 | Jmjd6 | 10:105781752-105787803 | 1.59E-10 | 3.58 | 4.04E-08 |
| ENSRNOG00000014448.8 | Arntl | 1:178039062-178137465 | 2.13E-10 | 9.73 | 5.39E-08 |
| ENSRNOG00000028711.3 | Dgat1 | 7:117566367-117576737 | 2.24E-10 | 5.47 | 5.62E-08 |
| ENSRNOG00000004870.5 | Dyrk3 | 13:47906230-47916877 | 2.4E-10 | 4.70 | 5.98E-08 |
| ENSRNOG00000025889.5 | Gnas | 3:172374956-172428483 | 2.44E-10 | 4.02 | 6.04E-08 |
| ENSRNOG00000059857.1 | Rnd1 | 7:140349137-140356209 | 2.55E-10 | 9.60 | 6.25E-08 |
| ENSRNOG00000014727.6 | Fahd1 | 10:14214518-14215957 | 2.75E-10 | 2.76 | 6.70E-08 |
| ENSRNOG00000005600.7 | Nr4a2 | 3:43111239-43119159 | 3.25E-10 | 5.85 | 7.85E-08 |
| ENSRNOG00000000321.5 | Cd24 | 20:48335539-48340846 | 3.39E-10 | 5.32 | 8.14E-08 |
| ENSRNOG00000046207.1 | Cbx4 | 10:108190994-108196217 | 3.66E-10 | 2.77 | 8.72E-08 |
| ENSRNOG00000023786.6 | Ybx1 | 5:138319761-138336475 | 3.73E-10 | 2.09 | 8.82E-08 |
| ENSRNOG00000013090.7 | Gadd45g | 17:13391466-13393243 | 3.96E-10 | 7.05 | 9.29E-08 |
| ENSRNOG00000021812.4 | Scx | 7:117519074-117521093 | 3.99E-10 | 9.86 | 9.29E-08 |
| ENSRNOG00000019422.4 | Egr1 | 18:27657627-27661429 | 4.09E-10 | 16.54 | 9.46E-08 |
| ENSRNOG00000021269.5 | Chgb | 3:125428259-125441594 | 4.38E-10 | 4.39 | 1.01E-07 |
| ENSRNOG00000033152.3 | Rps18l1 | 10:104521699-104522241 | 4.55E-10 | 3.14 | 1.04E-07 |
| ENSRNOG00000001728.7 | Fam43a | 11:73676600-73678984 | 5.72E-10 | 2.30 | 1.29E-07 |
| ENSRNOG00000003476.7 | Slc6a4 | 10:63153650-63176463 | 5.91E-10 | 4.98 | 1.31E-07 |
| ENSRNOG00000011222.5 | Dynll1 | 12:47074199-47076572 | 5.91E-10 | 2.76 | 1.31E-07 |
| ENSRNOG00000010178.6 | Cgrrf1 | 15:23619122-23639628 | 7.64E-10 | 3.13 | 1.68E-07 |
| ENSRNOG00000052549.1 | Chga | 6:126434225-126445568 | 7.68E-10 | 3.20 | 1.68E-07 |
| ENSRNOG00000051831.1 | 5_8S_rRNA | 1:11906440-11906593 | 8.6E-10 | -43.07 | 1.87E-07 |
| ENSRNOG00000011511.7 | Stk24 | 15:106629311-106678918 | 9.28E-10 | 3.36 | 2.01E-07 |
| ENSRNOG00000015222.5 | Tent5c | 2:202798197-202816562 | 9.93E-10 | 4.96 | 2.13E-07 |
| ENSRNOG00000015496.6 | Tpm4 | 16:19385735-19399903 | 1.08E-09 | 2.24 | 2.31E-07 |
| ENSRNOG00000056457.1 | Gpd1 | 7:141370490-141377928 | 1.15E-09 | -3.25 | 2.43E-07 |
| ENSRNOG00000020410.5 | Th | 1:216073030-216080287 | 1.17E-09 | 3.32 | 2.47E-07 |
| ENSRNOG00000010833.5 | Mthfd2 | 4:115004447-115015965 | 1.18E-09 | 2.51 | 2.48E-07 |
| ENSRNOG00000016573.6 | Dgat2 | 1:164113458-164143818 | 1.21E-09 | -5.52 | 2.53E-07 |
| ENSRNOG00000010524.6 | Cryab | 8:55178288-55182545 | 1.28E-09 | 3.71 | 2.65E-07 |
| ENSRNOG00000011704.5 | Fbxo34 | 15:24254041-24334890 | 1.3E-09 | 4.09 | 2.66E-07 |
| ENSRNOG00000009887.7 | Arih1 | 8:64166359-64268555 | 1.32E-09 | 2.56 | 2.69E-07 |
| ENSRNOG00000024428.4 | Kif20a | 18:27424327-27432814 | 1.39E-09 | 6.46 | 2.83E-07 |
| ENSRNOG00000021131.6 | Mindy1 | 2:196462553-196470130 | 1.73E-09 | -3.15 | 3.49E-07 |
| ENSRNOG00000014204.7 | Pacsin3 | 3:80072488-80081047 | 2.35E-09 | -2.74 | 4.69E-07 |
| ENSRNOG00000004132.7 | Lasp1 | 10:85744567-85785133 | 2.37E-09 | 2.88 | 4.70E-07 |
| ENSRNOG00000058186.1 | Errfi1 | 5:167952727-167966009 | 2.43E-09 | 3.08 | 4.78E-07 |
| ENSRNOG00000005359.7 | Csrnp3 | 3:51883558-52120290 | 2.48E-09 | 18.47 | 4.85E-07 |
| ENSRNOG00000006444.6 | Fkbp4 | 4:161748992-161757447 | 2.65E-09 | 2.20 | 5.15E-07 |
| ENSRNOG00000014205.2 | Klf2 | 16:19223086-19225037 | 3.58E-09 | 3.05 | 6.88E-07 |
| ENSRNOG00000050343.3 | Jmy | 2:23042188-23097610 | 3.56E-09 | 7.09 | 6.88E-07 |
| ENSRNOG00000020723.3 | Pten | 1:251421595-251487832 | 3.77E-09 | 2.44 | 7.21E-07 |
| ENSRNOG00000007573.4 | Hoxb9 | 10:84119883-84123390 | 3.91E-09 | 3.22 | 7.43E-07 |
| ENSRNOG00000009766.7 | Gpr180 | 15:103344475-103369630 | 4.1E-09 | 4.80 | 7.75E-07 |
| ENSRNOG00000032232.3 | Snrpg | 1:91588227-91588609 | 4.35E-09 | -9.68 | 8.18E-07 |
| ENSRNOG00000020325.6 | Calhm2 | 1:266908368-266914093 | 4.4E-09 | 5.98 | 8.21E-07 |
| ENSRNOG00000003228.5 | Mid1ip1 | X:13114568-13116743 | 6.28E-09 | -2.51 | 1.17E-06 |
| ENSRNOG00000016989.3 | Dolk | 3:8850153-8852192 | 7.07E-09 | -2.97 | 1.31E-06 |
| ENSRNOG00000049593.2 | Wbp11l1 | 4:170772162-170810080 | 7.57E-09 | -44.36 | 1.39E-06 |
| ENSRNOG00000001172.7 | Rnf10 | 12:47103313-47138128 | 7.82E-09 | 2.26 | 1.43E-06 |
| ENSRNOG00000014287.6 | Stk11 | 7:12440750-12457513 | 8.09E-09 | 2.13 | 1.47E-06 |
| ENSRNOG00000007390.6 | Nfkbia | 6:76267227-76270457 | 8.35E-09 | 2.44 | 1.51E-06 |
| ENSRNOG00000020679.6 | Icam1 | 8:22035255-22047059 | 9.61E-09 | 6.13 | 1.73E-06 |
| ENSRNOG00000058183.1 | AABR07040624.1 | X:106607046-106607352 | 9.68E-09 | -3.39 | 1.73E-06 |
| ENSRNOG00000005698.2 | Ndufa5 | 4:51590412-51598771 | 9.84E-09 | 3.33 | 1.75E-06 |
| ENSRNOG00000001414.2 | Serpine1 | 12:22641103-22651482 | 1.21E-08 | 5.08 | 2.13E-06 |
| ENSRNOG00000004255.4 | Usf1 | 13:89797799-89805558 | 1.26E-08 | -2.46 | 2.21E-06 |
| ENSRNOG00000002470.8 | Ifi47 | 10:34277992-34285623 | 1.3E-08 | -2.56 | 2.27E-06 |
| ENSRNOG00000027405.6 | Ccp110 | 1:188420460-188444991 | 1.39E-08 | 12.57 | 2.41E-06 |
| ENSRNOG00000010240.6 | Tent5a | 8:92937738-92942076 | 1.41E-08 | 7.13 | 2.42E-06 |
| ENSRNOG00000013654.4 | Cbln2 | 18:83777664-83782930 | 1.51E-08 | 6.01 | 2.59E-06 |
| ENSRNOG00000039249.3 | AABR07035539.1 | 12:19159467-19159901 | 1.89E-08 | -8.21 | 3.20E-06 |
| ENSRNOG00000045967.2 | AABR07064061.1 | 6:62796950-62798384 | 1.96E-08 | 7.86 | 3.31E-06 |
| ENSRNOG00000011927.7 | Sdc3 | 5:148923097-148956404 | 2.14E-08 | -2.61 | 3.58E-06 |
| ENSRNOG00000031244.3 | LOC108349606 | 18:4371050-4371899 | 2.14E-08 | -2.11 | 3.58E-06 |
| ENSRNOG00000013520.6 | Mat2a | 4:100296074-100303080 | 2.46E-08 | 2.07 | 4.08E-06 |
| ENSRNOG00000005248.6 | Slc1a4 | 14:104581189-104612597 | 2.53E-08 | 7.57 | 4.17E-06 |
| ENSRNOG00000015514.7 | Bcat1 | 4:179259307-179339795 | 2.72E-08 | 3.82 | 4.45E-06 |
| ENSRNOG00000000275.8 | Fam13c | 20:19360229-19479325 | 2.87E-08 | 4.20 | 4.64E-06 |
| ENSRNOG00000006305.5 | Slc38a2 | 7:138088648-138100841 | 2.88E-08 | 2.47 | 4.64E-06 |
| ENSRNOG00000010574.4 | Ptpn1 | 3:164665531-164711848 | 2.88E-08 | 2.67 | 4.64E-06 |
| ENSRNOG00000018214.6 | Bok | 9:100829551-100840504 | 3.22E-08 | 2.08 | 5.13E-06 |
| ENSRNOG00000019536.5 | Smim3 | 18:55744809-55771730 | 3.2E-08 | 2.11 | 5.13E-06 |
| ENSRNOG00000024923.6 | Nnat | 3:154043872-154046330 | 3.24E-08 | 2.58 | 5.15E-06 |
| ENSRNOG00000015036.6 | Ccn2 | 1:21851659-21854773 | 3.47E-08 | 2.89 | 5.47E-06 |
| ENSRNOG00000047005.2 | Kcnk5 | 15:4554602-4597000 | 3.48E-08 | 3.12 | 5.47E-06 |
| ENSRNOG00000050869.2 | Cebpd | 11:89008007-89009146 | 3.62E-08 | 3.81 | 5.67E-06 |
| ENSRNOG00000025235.6 | Tmem130 | 12:11814070-11840100 | 3.68E-08 | 4.58 | 5.74E-06 |
| ENSRNOG00000008116.5 | Plpp3 | 5:124690213-124765498 | 3.76E-08 | 2.09 | 5.80E-06 |
| ENSRNOG00000008709.6 | Arhgap32 | 8:33239138-33392305 | 3.78E-08 | 5.77 | 5.80E-06 |
| ENSRNOG00000059720.1 | Syp | X:15695565-15707436 | 3.97E-08 | 4.19 | 6.07E-06 |
| ENSRNOG00000001049.7 | Tpt1 | 15:57891679-57894504 | 4.16E-08 | 2.35 | 6.32E-06 |
| ENSRNOG00000013165.4 | Bet1l | 1:213602849-213605643 | 4.17E-08 | -3.33 | 6.32E-06 |
| ENSRNOG00000000957.5 | Rpl21 | 12:9996950-9998779 | 4.23E-08 | 2.10 | 6.38E-06 |
| ENSRNOG00000002232.6 | Aff1 | 14:7226585-7384876 | 4.26E-08 | 5.10 | 6.40E-06 |
| ENSRNOG00000022054.2 | Paqr7 | 5:152708774-152720229 | 4.3E-08 | -3.29 | 6.42E-06 |
| ENSRNOG00000016460.7 | Clu | 15:42640145-42665857 | 4.32E-08 | 2.27 | 6.43E-06 |
| ENSRNOG00000030447.5 | AC105662.1 | 5:173148883-173149276 | 4.58E-08 | 11.79 | 6.78E-06 |
| ENSRNOG00000018107.5 | Zfand5 | 1:238843337-238852566 | 4.84E-08 | 2.04 | 7.10E-06 |
| ENSRNOG00000026880.6 | Usp38 | 19:30668601-30699859 | 5.5E-08 | 3.74 | 8.04E-06 |
| ENSRNOG00000010819.6 | Hspa4l | 2:127625682-127677503 | 5.89E-08 | 4.70 | 8.57E-06 |
| ENSRNOG00000025443.4 | Map1lc3a | 3:150801288-150802935 | 6.5E-08 | 2.69 | 9.42E-06 |
| ENSRNOG00000019587.6 | Ptprn | 9:82446632-82461903 | 6.83E-08 | 3.84 | 9.85E-06 |
| ENSRNOG00000018393.4 | Uts2 | 5:168078747-168084145 | 7.06E-08 | 17.92 | 1.00E-05 |
| ENSRNOG00000021719.7 | Slfn5 | 10:70298518-70312070 | 7.03E-08 | -2.67 | 1.00E-05 |
| ENSRNOG00000016281.5 | Col4a1 | 16:83522161-83632153 | 7.7E-08 | 2.73 | 1.09E-05 |
| ENSRNOG00000047706.3 | LOC103690108 | 20:3791406-3794027 | 9E-08 | 3.56 | 1.27E-05 |
| ENSRNOG00000058920.1 | Coa8 | 6:136279495-136304095 | 9.35E-08 | 4.47 | 1.31E-05 |
| ENSRNOG00000020546.5 | Lipe | 1:82248045-82266727 | 1E-07 | -2.44 | 1.40E-05 |
| ENSRNOG00000016374.8 | Fgfr2 | 1:200590952-200696928 | 1.02E-07 | 2.06 | 1.41E-05 |
| ENSRNOG00000047396.2 | Rmnd5b | 10:34979922-34990943 | 1.04E-07 | -7.27 | 1.44E-05 |
| ENSRNOG00000019202.8 | Pvr | 1:80820049-80835701 | 1.07E-07 | 4.65 | 1.48E-05 |
| ENSRNOG00000027151.5 | Lrrc58 | 11:65747035-65759581 | 1.19E-07 | 9.57 | 1.63E-05 |
| ENSRNOG00000009639.5 | Zrsr1 | 14:107750161-107751892 | 1.31E-07 | 2.83 | 1.78E-05 |
| ENSRNOG00000010737.7 | Mbnl2 | 15:105640096-105797932 | 1.36E-07 | 2.39 | 1.83E-05 |
| ENSRNOG00000019141.3 | Ch25h | 1:252807064-252808380 | 1.36E-07 | 4.94 | 1.83E-05 |
| ENSRNOG00000060728.1 | Tuba1a | 7:140637286-140640953 | 1.39E-07 | 2.53 | 1.87E-05 |
| ENSRNOG00000018487.3 | Slc3a2 | 1:224906553-224921092 | 1.44E-07 | 2.06 | 1.93E-05 |
| ENSRNOG00000006539.7 | Pex16 | 3:81283136-81292573 | 1.48E-07 | -2.35 | 1.97E-05 |
| ENSRNOG00000056596.1 | Alas1 | 8:114927721-114940177 | 1.58E-07 | 2.13 | 2.09E-05 |
| ENSRNOG00000003977.5 | Dusp1 | 10:16970625-16973418 | 1.63E-07 | 8.61 | 2.14E-05 |
| ENSRNOG00000005292.7 | Trip11 | 6:125742795-125812517 | 1.75E-07 | 3.86 | 2.28E-05 |
| ENSRNOG00000007433.8 | Cyb561 | 10:94136992-94147621 | 1.81E-07 | 3.11 | 2.35E-05 |
| ENSRNOG00000009625.6 | Dpysl2 | 15:43477628-43542939 | 1.81E-07 | 2.75 | 2.35E-05 |
| ENSRNOG00000015055.6 | Scg2 | 9:85237457-85243001 | 1.94E-07 | 3.65 | 2.51E-05 |
| ENSRNOG00000025608.4 | Lrat | 2:181896304-181905300 | 2.32E-07 | 3.02 | 2.98E-05 |
| ENSRNOG00000001052.7 | Slc25a30 | 15:57813355-57834173 | 2.38E-07 | 4.02 | 3.04E-05 |
| ENSRNOG00000014429.5 | Arfgap2 | 3:80081646-80093658 | 2.63E-07 | -2.05 | 3.33E-05 |
| ENSRNOG00000058039.1 | Acta2 | 1:252537614-252550394 | 3.05E-07 | 3.65 | 3.82E-05 |
| ENSRNOG00000005700.7 | Nsg1 | 14:77380261-77401996 | 3.59E-07 | 3.09 | 4.49E-05 |
| ENSRNOG00000033581.5 | AABR07044388.2 | 20:4436418-4486680 | 3.62E-07 | -4.23 | 4.51E-05 |
| ENSRNOG00000001843.5 | Bcl6 | 11:80255789-80279075 | 3.72E-07 | 6.36 | 4.62E-05 |
| ENSRNOG00000021027.6 | Dbp | 1:101687854-101692846 | 4.18E-07 | -4.93 | 5.16E-05 |
| ENSRNOG00000001416.5 | Vgf | 12:22677606-22680630 | 4.22E-07 | 14.70 | 5.18E-05 |
| ENSRNOG00000010716.3 | Atoh8 | 4:100067340-100099517 | 4.22E-07 | 4.08 | 5.18E-05 |
| ENSRNOG00000019661.3 | Gdf15 | 16:20555394-20557978 | 4.32E-07 | 5.48 | 5.28E-05 |
| ENSRNOG00000017333.5 | Syt4 | 18:24172586-24182012 | 4.52E-07 | 5.85 | 5.51E-05 |
| ENSRNOG00000046382.2 | Gatd1 | 1:214389254-214394411 | 4.81E-07 | -36.36 | 5.82E-05 |
| ENSRNOG00000050786.2 | AC103024.2 | 10:34316836-34317161 | 4.81E-07 | -35.46 | 5.82E-05 |
| ENSRNOG00000043451.4 | Spp1 | 14:6673685-6679901 | 5E-07 | 7.52 | 6.00E-05 |
| ENSRNOG00000016595.5 | Hhex | 1:256101902-256107649 | 5.11E-07 | 2.66 | 6.11E-05 |
| ENSRNOG00000006604.3 | Thy1 | 8:48382120-48386935 | 5.18E-07 | 2.61 | 6.17E-05 |
| ENSRNOG00000023257.5 | Adamts9 | 4:124660261-124858250 | 5.41E-07 | 3.88 | 6.42E-05 |
| ENSRNOG00000018294.6 | Hspa5 | 3:13838303-13842762 | 5.9E-07 | 2.05 | 6.98E-05 |
| ENSRNOG00000002343.7 | Uchl1 | 14:43133217-43143973 | 5.95E-07 | 2.57 | 7.01E-05 |
| ENSRNOG00000002218.5 | Stbd1 | 14:16976355-16979760 | 6.12E-07 | 2.64 | 7.19E-05 |
| ENSRNOG00000020551.7 | Bles03 | 1:220806472-220808804 | 6.54E-07 | -2.72 | 7.63E-05 |
| ENSRNOG00000045636.2 | Fasn | 10:109987734-110005901 | 6.68E-07 | -4.40 | 7.76E-05 |
| ENSRNOG00000006420.6 | Rbm38 | 3:171037956-171050475 | 7.48E-07 | 4.32 | 8.66E-05 |
| ENSRNOG00000000906.7 | Medag | 12:6711746-6740714 | 7.66E-07 | -3.28 | 8.78E-05 |
| ENSRNOG00000001607.7 | Adamts1 | 11:25342125-25350974 | 7.64E-07 | 3.96 | 8.78E-05 |
| ENSRNOG00000019704.3 | Resp18 | 9:82470758-82477181 | 7.65E-07 | 2.38 | 8.78E-05 |
| ENSRNOG00000024578.7 | Ttyh2 | 10:103206013-103248862 | 8.17E-07 | -2.77 | 9.33E-05 |
| ENSRNOG00000011310.8 | Pde10a | 1:52360295-52544450 | 8.91E-07 | 6.33 | 0.0001 |
| ENSRNOG00000021198.4 | Hist1h2bq | 17:43679924-43689311 | 9.1E-07 | 2.02 | 0.0001 |
| ENSRNOG00000018109.4 | Clic4 | 5:153568744-153625869 | 9.73E-07 | 4.34 | 0.0001 |
| ENSRNOG00000022268.6 | Pnpla3 | 7:125034763-125055976 | 1.03E-06 | -13.31 | 0.0001 |
| ENSRNOG00000002946.3 | Socs3 | 10:106975177-106976040 | 1.06E-06 | 5.43 | 0.0001 |
| ENSRNOG00000040287.4 | Cyp1b1 | 6:2307807-2316722 | 1.08E-06 | 2.29 | 0.0001 |
| ENSRNOG00000056028.1 | Rfxap | 2:144003453-144007636 | 1.12E-06 | -4.34 | 0.0001 |
| ENSRNOG00000010170.5 | Tubb4b | 3:2441732-2444281 | 1.13E-06 | 2.23 | 0.0001 |
| ENSRNOG00000004139.8 | Ndel1 | 10:55411900-55444861 | 1.19E-06 | 2.11 | 0.0001 |
| ENSRNOG00000008572.6 | Ppcs | 5:138466292-138470096 | 1.19E-06 | -5.20 | 0.0001 |
| ENSRNOG00000014648.6 | Efnb2 | 16:86631063-86675049 | 1.18E-06 | 2.86 | 0.0001 |
| ENSRNOG00000025484.3 | Tmem177 | 13:35929818-35933243 | 1.19E-06 | -5.14 | 0.0001 |
| ENSRNOG00000019383.7 | Tef | 7:123043502-123058604 | 1.21E-06 | -2.89 | 0.0001 |
| ENSRNOG00000013250.5 | Pdcd5 | 1:91173472-91178801 | 1.25E-06 | -3.48 | 0.0001 |
| ENSRNOG00000010630.6 | Prcp | 1:157701088-157750169 | 1.28E-06 | 4.43 | 0.0001 |
| ENSRNOG00000020771.4 | Sap18 | 10:89635674-89636193 | 1.36E-06 | 2.02 | 0.0001 |
| ENSRNOG00000017748.6 | Nkx6-2 | 1:211922388-211923929 | 1.46E-06 | 6.53 | 0.0002 |
| ENSRNOG00000013948.5 | Zc3hav1 | 4:66011914-66062089 | 1.68E-06 | 2.03 | 0.0002 |
| ENSRNOG00000000521.4 | Cdkn1a | 20:6351457-6358864 | 1.87E-06 | 4.73 | 0.0002 |
| ENSRNOG00000001086.5 | Vps37b | 12:37984789-38023368 | 1.93E-06 | 2.64 | 0.0002 |
| ENSRNOG00000001548.6 | Nfe2l2 | 3:62497570-62524996 | 2.05E-06 | -2.02 | 0.0002 |
| ENSRNOG00000057696.1 | Sbk1 | 1:197659186-197681006 | 2.31E-06 | -3.50 | 0.0002 |
| ENSRNOG00000002461.7 | Nid1 | 17:90553393-90627101 | 2.35E-06 | 3.07 | 0.0002 |
| ENSRNOG00000006931.5 | Eepd1 | 8:26652120-26758325 | 2.46E-06 | -2.49 | 0.0003 |
| ENSRNOG00000050006.1 | Agtr2 | X:119390012-119393842 | 2.55E-06 | 2.51 | 0.0003 |
| ENSRNOG00000000158.5 | Cdo1 | 18:40701983-40716686 | 2.58E-06 | -3.13 | 0.0003 |
| ENSRNOG00000028652.4 | Map10 | 19:58505810-58508603 | 2.59E-06 | 7.98 | 0.0003 |
| ENSRNOG00000058393.1 | Taf15 | 10:70689862-70721892 | 2.61E-06 | -2.36 | 0.0003 |
| ENSRNOG00000011992.4 | Slc18a1 | 16:22361997-22395183 | 2.66E-06 | 3.19 | 0.0003 |
| ENSRNOG00000018809.7 | Psmd5 | 3:13916202-13933658 | 2.79E-06 | -2.39 | 0.0003 |
| ENSRNOG00000015408.5 | Vil1 | 9:81689801-81717621 | 3.01E-06 | 12.15 | 0.0003 |
| ENSRNOG00000031641.4 | Rpl35a | 5:164923301-164923634 | 3.28E-06 | 45.89 | 0.0003 |
| ENSRNOG00000016596.3 | Hspa4 | 10:38601623-38642397 | 3.33E-06 | 2.06 | 0.0003 |
| ENSRNOG00000028368.7 | Etnk2 | 13:50481162-50499140 | 3.49E-06 | -2.25 | 0.0003 |
| ENSRNOG00000029115.5 | LOC102555453 | 7:59762501-59763219 | 3.73E-06 | 3.06 | 0.0004 |
| ENSRNOG00000047250.2 | Gmfb | 15:23601367-23606634 | 3.89E-06 | 2.36 | 0.0004 |
| ENSRNOG00000008510.6 | Abtb2 | 3:93495105-93647721 | 4.32E-06 | 5.32 | 0.0004 |
| ENSRNOG00000008712.6 | Tmem268 | 5:79382095-79407433 | 4.54E-06 | -2.89 | 0.0004 |
| ENSRNOG00000015999.6 | Cirbp | 7:12401206-12405022 | 4.79E-06 | -2.38 | 0.0005 |
| ENSRNOG00000029512.1 | LOC100361854 | X:115495697-115496045 | 5.29E-06 | -2.32 | 0.0005 |
| ENSRNOG00000050997.1 | Ifrd1 | 6:60132023-60151420 | 5.37E-06 | 2.32 | 0.0005 |
| ENSRNOG00000003463.8 | Srebf1 | 10:46570995-46593009 | 5.84E-06 | -2.28 | 0.0005 |
| ENSRNOG00000001851.7 | Far2 | 4:182483193-182565436 | 6.11E-06 | 3.29 | 0.0006 |
| ENSRNOG00000051592.1 | AABR07044925.1 | 20:26833356-26852199 | 6.13E-06 | 3.15 | 0.0006 |
| ENSRNOG00000027008.6 | Igtp | 10:43613930-43653390 | 6.37E-06 | -3.77 | 0.0006 |
| ENSRNOG00000038045.4 | AC128960.1 | 17:78876110-78876537 | 6.65E-06 | -2.17 | 0.0006 |
| ENSRNOG00000027145.6 | Rora | 8:75516903-75607212 | 6.96E-06 | 3.56 | 0.0006 |
| ENSRNOG00000061056.1 | AABR07042514.3 | X:158581630-158582269 | 6.96E-06 | 3.08 | 0.0006 |
| ENSRNOG00000002254.6 | Tmem33 | 14:42535700-42560174 | 7.16E-06 | 2.71 | 0.0007 |
| ENSRNOG00000020232.5 | Rit1 | 2:188087485-188099444 | 7.25E-06 | -2.12 | 0.0007 |
| ENSRNOG00000014475.4 | Slc31a1 | 5:78222503-78249358 | 7.37E-06 | 2.17 | 0.0007 |
| ENSRNOG00000007544.5 | Il23r | 4:98203957-98305173 | 7.54E-06 | 5.86 | 0.0007 |
| ENSRNOG00000016099.2 | Id4 | 17:16692556-16695126 | 7.5E-06 | 3.38 | 0.0007 |
| ENSRNOG00000052421.1 | AABR07012475.2 | 2:196587205-196591324 | 7.54E-06 | -5.84 | 0.0007 |
| ENSRNOG00000031851.5 | Ndufa4l2 | 7:70821956-70824085 | 7.9E-06 | 2.75 | 0.0007 |
| ENSRNOG00000049203.3 | Pex5 | 4:156983914-157009674 | 7.91E-06 | 2.28 | 0.0007 |
| ENSRNOG00000022273.6 | Rfk | 1:236494220-236501733 | 8.12E-06 | 2.01 | 0.0007 |
| ENSRNOG00000005802.7 | Usp24 | 5:125896724-126030401 | 8.77E-06 | 2.38 | 0.0008 |
| ENSRNOG00000058470.1 | Col12a1 | 8:87040798-87158368 | 8.88E-06 | 3.21 | 0.0008 |
| ENSRNOG00000017260.6 | Cdr2 | 1:190889700-190914610 | 9.11E-06 | 4.59 | 0.0008 |
| ENSRNOG00000008837.6 | Ass1 | 3:10327413-10375826 | 9.43E-06 | 4.24 | 0.0008 |
| ENSRNOG00000012477.4 | Eef1a2 | 3:176657106-176666282 | 9.51E-06 | 3.26 | 0.0008 |
| ENSRNOG00000010408.7 | Polr2k | 7:74989222-74992582 | 1E-05 | -2.13 | 0.0009 |
| ENSRNOG00000006331.4 | Elovl5 | 8:85259981-85285983 | 1.02E-05 | 2.56 | 0.0009 |
| ENSRNOG00000013408.5 | Npas2 | 9:45901740-46081880 | 1.11E-05 | 6.23 | 0.0009 |
| ENSRNOG00000005275.6 | Shmt1 | 10:47031049-47059216 | 1.24E-05 | -2.22 | 0.0011 |
| ENSRNOG00000020703.7 | Sipa1l3 | 1:87260834-87468288 | 1.27E-05 | 3.02 | 0.0011 |
| ENSRNOG00000004234.5 | Mgat2 | 6:91476697-91479183 | 1.29E-05 | 2.14 | 0.0011 |
| ENSRNOG00000007542.6 | Scg5 | 3:105235049-105279462 | 1.31E-05 | 2.70 | 0.0011 |
| ENSRNOG00000054344.1 | Ier2 | 19:25774142-25775659 | 1.48E-05 | 2.31 | 0.0012 |
| ENSRNOG00000007808.3 | Nap1l5 | 4:89149316-89151184 | 1.5E-05 | 3.74 | 0.0013 |
| ENSRNOG00000051158.3 | Cfb | 20:4536211-4542073 | 1.54E-05 | 9.12 | 0.0013 |
| ENSRNOG00000008782.6 | Pnisr | 5:35991067-36017944 | 1.56E-05 | -2.16 | 0.0013 |
| ENSRNOG00000004693.8 | Pbx1 | 13:86390740-86451002 | 1.64E-05 | 2.29 | 0.0014 |
| ENSRNOG00000030345.4 | LOC100362684 | 9:1012090-1012450 | 1.68E-05 | -9.09 | 0.0014 |
| ENSRNOG00000020254.7 | Per2 | 9:98555168-98597359 | 1.7E-05 | -3.09 | 0.0014 |
| ENSRNOG00000033169.5 | Cpeb4 | 10:15987920-16046033 | 1.71E-05 | 2.84 | 0.0014 |
| ENSRNOG00000000064.4 | Atp5me | 14:2325307-2326436 | 1.78E-05 | -2.05 | 0.0015 |
| ENSRNOG00000008001.7 | Rab3b | 5:128501846-128568188 | 1.78E-05 | 2.79 | 0.0015 |
| ENSRNOG00000014310.7 | Tmtc4 | 15:109339563-109394927 | 1.81E-05 | -2.33 | 0.0015 |
| ENSRNOG00000059268.2 | Ppp1r10 | 20:3329676-3344286 | 2.04E-05 | 2.44 | 0.0017 |
| ENSRNOG00000003895.7 | Rgs1 | 13:61066152-61070599 | 2.12E-05 | 7.89 | 0.0017 |
| ENSRNOG00000002911.5 | Alb | 14:19176276-19191863 | 2.23E-05 | -9.70 | 0.0018 |
| ENSRNOG00000008376.6 | LOC100909595 | 4:155408232-155631856 | 2.29E-05 | 3.40 | 0.0018 |
| ENSRNOG00000015415.6 | Rhoq | 6:10533150-10568581 | 2.5E-05 | 2.42 | 0.0020 |
| ENSRNOG00000009946.7 | Ldlr | 8:22750335-22774903 | 2.62E-05 | 2.20 | 0.0021 |
| ENSRNOG00000016298.7 | Lysmd3 | 2:9526208-9534362 | 2.63E-05 | 2.89 | 0.0021 |
| ENSRNOG00000028899.3 | LOC100910807 | X:124393331-124398001 | 2.72E-05 | 4.00 | 0.0021 |
| ENSRNOG00000031090.6 | RT1-CE7 | 20:4694471-4896970 | 2.76E-05 | 2.36 | 0.0022 |
| ENSRNOG00000004660.5 | Fzd6 | 7:77899321-77931029 | 2.78E-05 | -3.00 | 0.0022 |
| ENSRNOG00000008224.6 | Jdp2 | 6:109466059-109505161 | 2.78E-05 | 3.28 | 0.0022 |
| ENSRNOG00000009474.5 | Ddx42 | 10:94407558-94439052 | 2.84E-05 | -2.08 | 0.0022 |
| ENSRNOG00000003538.7 | Adamts4 | 13:89622631-89634419 | 3.07E-05 | 19.33 | 0.0024 |
| ENSRNOG00000005059.5 | LOC100911917 | 3:4861752-4866833 | 3.07E-05 | -25.40 | 0.0024 |
| ENSRNOG00000018524.6 | Ezr | 1:47287873-47331412 | 3.12E-05 | 2.15 | 0.0024 |
| ENSRNOG00000019050.4 | Ifit1 | 1:252944102-252946170 | 3.16E-05 | -4.76 | 0.0024 |
| ENSRNOG00000042592.2 | Rgs10 | 1:199782077-199823386 | 3.16E-05 | 2.16 | 0.0024 |
| ENSRNOG00000033433.4 | Csrnp1 | 8:128659867-128672284 | 3.35E-05 | 2.62 | 0.0025 |
| ENSRNOG00000049215.1 | Cbx2 | 10:108132104-108140935 | 3.43E-05 | 5.84 | 0.0026 |
| ENSRNOG00000008758.5 | Tspan18 | 3:82097067-82236664 | 3.44E-05 | 2.97 | 0.0026 |
| ENSRNOG00000059492.1 | LOC100365363 | 1:60884760-60894948 | 3.65E-05 | -3.53 | 0.0027 |
| ENSRNOG00000011815.8 | Sgk1 | 1:24185434-24302298 | 3.83E-05 | 2.84 | 0.0029 |
| ENSRNOG00000011292.6 | NEWGENE_621351 | 4:31534224-31569151 | 3.85E-05 | 3.00 | 0.0029 |
| ENSRNOG00000048195.2 | Nudt13 | 15:4434312-4454958 | 3.91E-05 | -3.67 | 0.0029 |
| ENSRNOG00000046536.1 | AABR07016141.1 | 14:94590764-94602088 | 3.94E-05 | -2.60 | 0.0029 |
| ENSRNOG00000021984.6 | Rgs7 | 13:93095199-93307199 | 3.96E-05 | 5.02 | 0.0029 |
| ENSRNOG00000008415.6 | Nab2 | 7:70961574-70969905 | 4.28E-05 | 4.09 | 0.0031 |
| ENSRNOG00000049814.2 | LOC100910882 | 3:152226709-152259156 | 4.5E-05 | 7.52 | 0.0033 |
| ENSRNOG00000018567.7 | Slc20a1 | 3:121725858-121739173 | 4.66E-05 | 2.25 | 0.0034 |
| ENSRNOG00000022582.2 | Ccnq | 10:66019519-66020682 | 4.73E-05 | -2.85 | 0.0034 |
| ENSRNOG00000024839.2 | Tysnd1 | 20:31313017-31318736 | 4.86E-05 | -2.18 | 0.0035 |
| ENSRNOG00000016758.7 | Loxl2 | 15:51303908-51365234 | 4.92E-05 | 2.92 | 0.0035 |
| ENSRNOG00000020379.7 | Cog8 | 19:39246624-39257451 | 5.04E-05 | -2.63 | 0.0036 |
| ENSRNOG00000001128.6 | Tesc | 12:44141421-44174583 | 5.06E-05 | 2.06 | 0.0036 |
| ENSRNOG00000009329.7 | Nr1d1 | 10:86683874-86690815 | 5.14E-05 | -2.01 | 0.0037 |
| ENSRNOG00000027087.2 | Tmem186 | 10:7077487-7080798 | 5.25E-05 | -2.71 | 0.0037 |
| ENSRNOG00000012565.4 | Cln8 | 16:79828323-79838212 | 5.29E-05 | 2.95 | 0.0037 |
| ENSRNOG00000001251.6 | Grifin | 12:16170161-16172129 | 5.3E-05 | -6.24 | 0.0038 |
| ENSRNOG00000014142.6 | Ogfrl1 | 9:29634178-29647903 | 5.37E-05 | 2.72 | 0.0038 |
| ENSRNOG00000015125.5 | LOC100911548 | 4:157326726-157328379 | 5.53E-05 | -2.36 | 0.0039 |
| ENSRNOG00000017382.4 | Snx33 | 8:61584655-61595032 | 5.53E-05 | -2.57 | 0.0039 |
| ENSRNOG00000002793.5 | Sstr2 | 10:102136282-102143434 | 5.63E-05 | 2.43 | 0.0039 |
| ENSRNOG00000018778.7 | Cadm1 | 8:52127398-52189722 | 6.27E-05 | 3.46 | 0.0043 |
| ENSRNOG00000009565.4 | Pdk4 | 4:30546663-30556814 | 6.6E-05 | 2.88 | 0.0045 |
| ENSRNOG00000002582.5 | Hand1 | 10:43250744-43253296 | 6.65E-05 | 8.04 | 0.0046 |
| ENSRNOG00000029394.5 | Dusp8 | 1:215030428-215033460 | 7.11E-05 | 3.85 | 0.0049 |
| ENSRNOG00000010539.5 | Mcat | 7:124412761-124423832 | 7.23E-05 | -2.29 | 0.0049 |
| ENSRNOG00000031939.5 | AABR07066416.1 | 9:8629215-8632017 | 7.34E-05 | -2.93 | 0.0050 |
| ENSRNOG00000010775.7 | Arrdc4 | 1:129766099-129780356 | 7.47E-05 | 3.12 | 0.0051 |
| ENSRNOG00000013111.7 | Mettl3 | 15:28710299-28721127 | 7.66E-05 | -2.10 | 0.0052 |
| ENSRNOG00000005849.7 | Aco1 | 5:56425023-56481218 | 7.91E-05 | -2.13 | 0.0054 |
| ENSRNOG00000001465.7 | Ids | 8:69449800-69466618 | 7.97E-05 | 2.94 | 0.0054 |
| ENSRNOG00000000503.7 | Ppard | 20:7818288-7885333 | 8.01E-05 | 2.23 | 0.0054 |
| ENSRNOG00000027860.6 | Ggnbp2 | 10:72156727-72188308 | 8.17E-05 | -2.16 | 0.0055 |
| ENSRNOG00000053468.1 | Tuba1b | 7:140614751-140617721 | 8.23E-05 | 2.13 | 0.0055 |
| ENSRNOG00000056427.1 | AABR07007717.1 | 2:25564883-25565757 | 8.31E-05 | 3.15 | 0.0056 |
| ENSRNOG00000023972.6 | Col4a2 | 16:83387363-83438561 | 8.48E-05 | 2.18 | 0.0056 |
| ENSRNOG00000026787.5 | Disp2 | 3:110574416-110589921 | 9.16E-05 | 3.33 | 0.0060 |
| ENSRNOG00000007398.4 | Zfp691 | 5:138217844-138222534 | 9.37E-05 | -2.72 | 0.0062 |
| ENSRNOG00000048152.2 | Myo1b | 9:54558201-54766054 | 9.42E-05 | 2.31 | 0.0062 |
| ENSRNOG00000006320.7 | Ptges | 3:9727407-9738752 | 9.99E-05 | 3.61 | 0.0065 |
| ENSRNOG00000018003.6 | F2rl1 | 2:25222327-25235275 | 9.96E-05 | 11.95 | 0.0065 |
| ENSRNOG00000033915.3 | Gpt | 7:117759082-117761931 | 9.99E-05 | -3.58 | 0.0065 |
| ENSRNOG00000007730.3 | Vom2r53 | 7:13151591-13184257 | 0.0001 | 42.32 | 0.0071 |
| ENSRNOG00000009549.4 | Fbxo3 | 3:93968854-94000863 | 0.0001 | -2.22 | 0.0074 |
| ENSRNOG00000010633.3 | Acsl1 | 16:48937455-49003246 | 0.0001 | -2.09 | 0.0075 |
| ENSRNOG00000004535.6 | Kcng3 | 6:6794807-6842758 | 0.0001 | 7.26 | 0.0077 |
| ENSRNOG00000018646.6 | Hbegf | 18:29329763-29340403 | 0.0001 | 8.38 | 0.0077 |
| ENSRNOG00000012404.6 | Thrsp | 1:162381252-162385575 | 0.0001 | -7.83 | 0.0079 |
| ENSRNOG00000006048.6 | Ezh2 | 4:77284403-77347011 | 0.0001 | 4.40 | 0.0080 |
| ENSRNOG00000010658.6 | LOC103691744 | 2:264266983-264293046 | 0.0001 | 2.92 | 0.0080 |
| ENSRNOG00000011015.6 | Hivep2 | 1:8310576-8333885 | 0.0001 | 3.16 | 0.0081 |
| ENSRNOG00000038375.3 | AABR07026311.1 | 16:71438595-71439402 | 0.0001 | -3.25 | 0.0081 |
| ENSRNOG00000002097.5 | Rasl11b | 14:36550339-36554580 | 0.0001 | -2.05 | 0.0089 |
| ENSRNOG00000060544.1 | Kdm5b | 13:51384388-51455357 | 0.0001 | -2.13 | 0.0091 |
| ENSRNOG00000048686.3 | AABR07066379.1 | 9:8349032-8442298 | 0.0002 | -3.01 | 0.0097 |
| ENSRNOG00000007830.4 | Apold1 | 4:168752132-168755023 | 0.0002 | 3.40 | 0.0098 |
| ENSRNOG00000047072.2 | LOC100910656 | 7:116275816-116284587 | 0.0002 | 3.93 | 0.0098 |
| ENSRNOG00000014030.7 | Synm | 1:128692111-128722048 | 0.0002 | 2.39 | 0.0099 |
| ENSRNOG00000031328.3 | Zfp110 | 1:65742088-65756528 | 0.0002 | -2.34 | 0.0101 |
| ENSRNOG00000013712.7 | Tex261 | 4:115448707-115453659 | 0.0002 | 2.24 | 0.0102 |
| ENSRNOG00000007817.5 | Kctd6 | 15:18484509-18493366 | 0.0002 | 2.98 | 0.0108 |
| ENSRNOG00000010896.5 | Tprn | 3:2480231-2487710 | 0.0002 | 2.59 | 0.0108 |
| ENSRNOG00000003927.7 | Cd55 | 13:47126740-47154292 | 0.0002 | 4.59 | 0.0109 |
| ENSRNOG00000019361.5 | Inpp5j | 14:83730861-83741969 | 0.0002 | -5.86 | 0.0109 |
| ENSRNOG00000030091.6 | Dusp14 | 10:71363687-71383602 | 0.0002 | 2.27 | 0.0109 |
| ENSRNOG00000052347.1 | AABR07045684.1 | KL568128.1:56-3244 | 0.0002 | 5.03 | 0.0109 |
| ENSRNOG00000031174.6 | Arnt | 2:196594302-196651179 | 0.0002 | -2.03 | 0.0110 |
| ENSRNOG00000033844.4 | LOC100912195 | 1:116565789-116567189 | 0.0002 | 3.81 | 0.0110 |
| ENSRNOG00000012386.6 | Zbtb38 | 8:104589620-104593625 | 0.0002 | 2.57 | 0.0112 |
| ENSRNOG00000027694.3 | AABR07050545.1 | 5:169021559-169022145 | 0.0002 | -5.70 | 0.0112 |
| ENSRNOG00000016343.7 | Dkk3 | 1:176940692-176983045 | 0.0002 | 3.07 | 0.0118 |
| ENSRNOG00000000891.5 | AABR07035107.1 | 12:4737816-4741997 | 0.0002 | -2.43 | 0.0120 |
| ENSRNOG00000058457.1 | Chtopl1 | 4:163118451-163119391 | 0.0002 | 2.42 | 0.0125 |
| ENSRNOG00000001374.6 | Rasal1 | 12:41416217-41448668 | 0.0002 | -2.63 | 0.0129 |
| ENSRNOG00000013541.5 | Sh2d4a | 16:23083953-23156962 | 0.0002 | -2.15 | 0.0129 |
| ENSRNOG00000038184.4 | Camk2n2 | 11:83975366-83976588 | 0.0002 | 7.14 | 0.0129 |
| ENSRNOG00000011984.5 | Cxcl14 | 17:9109730-9117750 | 0.0002 | 2.11 | 0.0136 |
| ENSRNOG00000017657.5 | Phf23 | 10:56605139-56609233 | 0.0002 | -2.05 | 0.0136 |
| ENSRNOG00000012410.4 | S100a1 | 2:189900666-189903219 | 0.0002 | 2.84 | 0.0137 |
| ENSRNOG00000062039.1 | AABR07044420.2 | 20:5347665-5347939 | 0.0002 | 2.87 | 0.0137 |
| ENSRNOG00000038999.4 | RT1-A1 | 20:5351604-5421098 | 0.0002 | 2.65 | 0.0140 |
| ENSRNOG00000049866.2 | Plcxd1 | 12:52641221-52676608 | 0.0002 | 13.48 | 0.0141 |
| ENSRNOG00000016326.5 | Cx3cl1 | 19:10644243-10653800 | 0.0002 | -2.15 | 0.0143 |
| ENSRNOG00000002722.5 | Sec14l1 | 10:106065711-106112857 | 0.0003 | -2.02 | 0.0150 |
| ENSRNOG00000015518.7 | Rbp4 | 1:256806471-256813711 | 0.0003 | -4.54 | 0.0152 |
| ENSRNOG00000028895.4 | Rtp4 | 11:80638944-80650802 | 0.0003 | -3.48 | 0.0155 |
| ENSRNOG00000002894.7 | Mnt | 10:61685240-61700491 | 0.0003 | -3.82 | 0.0155 |
| ENSRNOG00000001989.6 | Alcam | 11:50781126-50985083 | 0.0003 | 2.73 | 0.0156 |
| ENSRNOG00000013413.7 | Rorb | 1:234252756-234435839 | 0.0003 | 11.89 | 0.0159 |
| ENSRNOG00000028382.3 | Rfxapl1 | 2:144213402-144217600 | 0.0003 | 2.83 | 0.0159 |
| ENSRNOG00000000720.5 | MGC95208 | 11:1814293-1819094 | 0.0003 | 2.10 | 0.0165 |
| ENSRNOG00000009570.7 | Pdxp | 7:120140459-120145908 | 0.0003 | 2.06 | 0.0166 |
| ENSRNOG00000016678.3 | Angptl2 | 3:12262821-12292663 | 0.0003 | -2.26 | 0.0166 |
| ENSRNOG00000011111.6 | Cipc | 6:110968060-110990912 | 0.0003 | -2.63 | 0.0169 |
| ENSRNOG00000021314.4 | Fdft1 | 15:46339248-46367302 | 0.0003 | -2.09 | 0.0171 |
| ENSRNOG00000061544.1 | SpoCT2 | 20:29655225-29677926 | 0.0003 | 2.76 | 0.0172 |
| ENSRNOG00000032922.7 | Dclk1 | 2:144646307-144936927 | 0.0003 | 3.74 | 0.0174 |
| ENSRNOG00000048109.2 | LOC100365839 | 11:79547698-79548560 | 0.0003 | -3.78 | 0.0174 |
| ENSRNOG00000019330.7 | Procr | 3:151285248-151289595 | 0.0003 | 4.34 | 0.0179 |
| ENSRNOG00000049099.1 | AABR07017250.1 | 15:19429507-19430011 | 0.0003 | -4.63 | 0.0179 |
| ENSRNOG00000018812.3 | Rpp25 | 8:62283335-62284716 | 0.0003 | -2.73 | 0.0180 |
| ENSRNOG00000020225.5 | Dlgap4 | 3:152752090-152846118 | 0.0003 | 2.16 | 0.0180 |
| ENSRNOG00000029698.5 | Pim3 | 7:129860113-129863441 | 0.0003 | 2.29 | 0.0185 |
| ENSRNOG00000019542.5 | MGC108823 | 18:55576238-55584957 | 0.0003 | -2.64 | 0.0188 |
| ENSRNOG00000006641.8 | Dbh | 3:5709235-5731898 | 0.0004 | 2.86 | 0.0190 |
| ENSRNOG00000017783.6 | Sfrp1 | 16:73372006-73410777 | 0.0004 | 2.31 | 0.0191 |
| ENSRNOG00000023465.4 | Depp1 | 4:148782478-148784562 | 0.0004 | 2.36 | 0.0191 |
| ENSRNOG00000011156.3 | Clstn3 | 4:157043924-157078130 | 0.0004 | 2.39 | 0.0195 |
| ENSRNOG00000014187.6 | Igf1r | 1:128924965-129206516 | 0.0004 | 2.76 | 0.0200 |
| ENSRNOG00000018775.7 | Cystm1 | 18:29191730-29232978 | 0.0004 | 2.14 | 0.0223 |
| ENSRNOG00000018295.7 | Ccar2 | 15:51818896-51834030 | 0.0004 | -2.14 | 0.0223 |
| ENSRNOG00000050465.1 | AABR07041109.1 | X:119532512-119532965 | 0.0004 | 2.72 | 0.0226 |
| ENSRNOG00000006965.6 | Aff4 | 10:38692210-38773021 | 0.0004 | 2.05 | 0.0229 |
| ENSRNOG00000003127.4 | Spryd4 | 7:2621960-2623781 | 0.0004 | -2.00 | 0.0231 |
| ENSRNOG00000001773.7 | Senp2 | 11:82630435-82664630 | 0.0005 | 2.32 | 0.0236 |
| ENSRNOG00000037658.3 | Gprasp2 | X:106360392-106363300 | 0.0005 | 2.59 | 0.0244 |
| ENSRNOG00000003300.3 | Btg2 | 13:50913179-50916982 | 0.0005 | 2.58 | 0.0247 |
| ENSRNOG00000007412.4 | Dok1 | 4:113864214-113866674 | 0.0005 | -3.52 | 0.0248 |
| ENSRNOG00000025310.4 | Pop7 | 12:22259712-22260754 | 0.0005 | -2.13 | 0.0248 |
| ENSRNOG00000031579.2 | LOC100363469 | 10:78992579-78993045 | 0.0005 | 2.97 | 0.0248 |
| ENSRNOG00000009686.2 | Aqp7 | 5:57358326-57372239 | 0.0005 | -5.30 | 0.0249 |
| ENSRNOG00000043387.3 | Cpe | 16:26906715-27014813 | 0.0005 | 2.39 | 0.0249 |
| ENSRNOG00000045621.2 | Gatc | 12:47031594-47036956 | 0.0005 | 20.87 | 0.0253 |
| ENSRNOG00000019900.6 | Slc35b2 | 9:17823399-17827032 | 0.0005 | -2.11 | 0.0253 |
| ENSRNOG00000047300.2 | Bdkrb2 | 6:129399467-129429676 | 0.0005 | 3.83 | 0.0261 |
| ENSRNOG00000049270.1 | Ppp2r3b | 14:1469747-1476355 | 0.0005 | 2.44 | 0.0261 |
| ENSRNOG00000018358.4 | Nt5dc2 | 16:7212487-7220540 | 0.0005 | 2.30 | 0.0267 |
| ENSRNOG00000010099.7 | Asb8 | 7:139724345-139734568 | 0.0005 | -2.20 | 0.0271 |
| ENSRNOG00000000303.7 | Cep57l1 | 20:46610145-46666830 | 0.0005 | -3.25 | 0.0273 |
| ENSRNOG00000032398.4 | RGD1562136 | 9:111327401-111347430 | 0.0006 | -2.05 | 0.0281 |
| ENSRNOG00000000648.6 | Jmjd1c | 20:22751742-22882672 | 0.0006 | 2.32 | 0.0286 |
| ENSRNOG00000054757.1 | Adcy6 | 7:140270691-140291620 | 0.0006 | 2.26 | 0.0300 |
| ENSRNOG00000020622.5 | Cilp2 | 16:21288875-21295869 | 0.0006 | -3.47 | 0.0304 |
| ENSRNOG00000008274.3 | Xpc | 4:123134456-123161985 | 0.0006 | -2.23 | 0.0319 |
| ENSRNOG00000002480.7 | Gpr137b | 17:90670871-90746804 | 0.0007 | 2.16 | 0.0326 |
| ENSRNOG00000006859.6 | Insig1 | 4:342301-350515 | 0.0007 | 2.17 | 0.0328 |
| ENSRNOG00000037478.2 | Noc4l | 12:52072187-52076739 | 0.0007 | -2.45 | 0.0332 |
| ENSRNOG00000050625.1 | LOC100911217 | 3:153949214-153952108 | 0.0007 | -4.93 | 0.0339 |
| ENSRNOG00000011114.7 | Tbpl1 | 1:23977687-24002506 | 0.0007 | 2.15 | 0.0347 |
| ENSRNOG00000020047.6 | Tut1 | 1:225151403-225162595 | 0.0007 | -2.16 | 0.0349 |
| ENSRNOG00000022082.6 | LOC103690006 | 4:117800627-117814658 | 0.0007 | -8.07 | 0.0350 |
| ENSRNOG00000052354.1 | Arhgef40 | 15:28377995-28403090 | 0.0007 | -2.11 | 0.0355 |
| ENSRNOG00000027938.5 | Jcad | 17:55670330-55709740 | 0.0007 | 3.32 | 0.0356 |
| ENSRNOG00000018242.7 | Camkk1 | 10:59585071-59608165 | 0.0008 | -3.62 | 0.0361 |
| ENSRNOG00000050539.2 | Fbln5 | 6:125644803-125723944 | 0.0008 | 2.00 | 0.0364 |
| ENSRNOG00000018755.6 | Acss2 | 3:151032951-151075856 | 0.0008 | -2.51 | 0.0385 |
| ENSRNOG00000001130.8 | Nos1 | 12:44213942-44520341 | 0.0008 | 3.42 | 0.0386 |
| ENSRNOG00000015133.6 | Kmt2a | 8:49114989-49158971 | 0.0008 | 3.33 | 0.0388 |
| ENSRNOG00000005984.7 | Etv6 | 4:167754524-167992168 | 0.0008 | 2.98 | 0.0389 |
| ENSRNOG00000001469.8 | Eln | 12:24978482-25021863 | 0.0008 | 3.33 | 0.0392 |
| ENSRNOG00000010587.7 | Crnkl1 | 3:140126796-140141679 | 0.0008 | -2.31 | 0.0392 |
| ENSRNOG00000016311.7 | Slc6a2 | 19:15391580-15431274 | 0.0009 | 5.18 | 0.0399 |
| ENSRNOG00000060972.1 | Heca | 1:13036908-13050644 | 0.0009 | 2.49 | 0.0404 |
| ENSRNOG00000045683.2 | LOC102553715 | 10:49000972-49003931 | 0.0009 | 2.88 | 0.0406 |
| ENSRNOG00000007327.5 | Pars2 | 5:126254141-126259200 | 0.0009 | -3.88 | 0.0406 |
| ENSRNOG00000055236.1 | Gemin4 | 10:64368808-64375816 | 0.0009 | -4.18 | 0.0406 |
| ENSRNOG00000010121.5 | Lef1 | 2:236233238-236345056 | 0.0009 | -2.02 | 0.0408 |
| ENSRNOG00000000875.8 | Fhl1 | X:159112879-159172524 | 0.0009 | 2.11 | 0.0408 |
| ENSRNOG00000004135.6 | RGD1311745 | 8:132029620-132036472 | 0.0009 | -2.37 | 0.0408 |
| ENSRNOG00000059764.1 | Snrpn | 1:195074329-195096460 | 0.0009 | 2.33 | 0.0415 |
| ENSRNOG00000018992.7 | Dpysl3 | 18:37716142-37819097 | 0.0009 | 3.89 | 0.0416 |
| ENSRNOG00000046566.2 | Tub | 1:173607100-173625550 | 0.0009 | 4.02 | 0.0416 |
| ENSRNOG00000059770.1 | AABR07044383.1 | 20:3791628-3796275 | 0.0009 | 4.63 | 0.0416 |
| ENSRNOG00000013017.7 | Arnt2 | 1:146399216-146556171 | 0.0009 | 3.00 | 0.0428 |
| ENSRNOG00000016167.5 | Spata2L | 19:56032609-56037077 | 0.0009 | 2.91 | 0.0428 |
| ENSRNOG00000018413.7 | Per3 | 5:168086997-168123395 | 0.0009 | -3.07 | 0.0428 |
| ENSRNOG00000056855.1 | LOC100911991 | X:154916378-154918095 | 0.0010 | -14.23 | 0.0438 |
| ENSRNOG00000002802.3 | Cxcl1 | 14:18743684-18745457 | 0.0010 | 10.05 | 0.0441 |
| ENSRNOG00000029330.4 | Ca5b | X:32232141-32292072 | 0.0010 | -3.20 | 0.0448 |
| ENSRNOG00000005670.5 | Art4 | 4:170830850-170841187 | 0.0010 | -2.35 | 0.0453 |
| ENSRNOG00000008965.4 | Socs2 | 7:36495479-36499784 | 0.0010 | -2.69 | 0.0457 |
| ENSRNOG00000022807.5 | Pced1b | 7:138707425-138846123 | 0.0010 | -2.40 | 0.0458 |
| ENSRNOG00000033100.4 | AABR07054189.1 | 3:143828610-143829156 | 0.0010 | -3.11 | 0.0459 |
| ENSRNOG00000004649.4 | Il1b | 3:121876262-121882726 | 0.0011 | 6.24 | 0.0466 |
| ENSRNOG00000032394.5 | Tymp | 7:130342482-130347587 | 0.0011 | -2.23 | 0.0466 |
| ENSRNOG00000003897.6 | Col1a1 | 10:82745800-82762789 | 0.0011 | 2.54 | 0.0467 |
| ENSRNOG00000016013.7 | Gprc5b | 1:188688742-188713280 | 0.0011 | 3.20 | 0.0469 |
| ENSRNOG00000018659.7 | Csf1 | 2:210522374-210550560 | 0.0011 | 2.37 | 0.0471 |
| ENSRNOG00000002886.6 | Myh10 | 10:55275410-55406732 | 0.0011 | 2.79 | 0.0479 |
| ENSRNOG00000014426.7 | Lox | 18:47500329-47577819 | 0.0011 | 2.15 | 0.0483 |
| ENSRNOG00000020340.7 | Brd8 | 18:27398687-27424090 | 0.0011 | 2.06 | 0.0486 |

* FDR: false discovery rate. The FDR-adjusted P value was sorted from lowest to highest.

Table S9. Statistics of differentially expressed genes (DEG) with *P* < 0.01, FDR-adjusted P value = 0.05 and |Fold change| > 2 in adrenal glands in comparison of CT vs. H120.

| Feature ID | Gene name | Position | P-value | Fold change | FDR-adjusted P value* |
| --- | --- | --- | --- | --- | --- |
| ENSRNOG00000033299.3 | Mt-atp8 | MT:7757-7961 | 0.00E+00 | -7.54 | 0.00E+00 |
| ENSRNOG00000031506.4 | Ftl1 | 5:152325870-152326778 | 6.4E-128 | 6.99 | 1.1E-123 |
| ENSRNOG00000020843.6 | LOC100360087 | 1:101448345-101449829 | 1.7E-113 | 5.78 | 1.9E-109 |
| ENSRNOG00000022392.3 | Hspb8 | 12:45905370-45920013 | 1.52E-58 | 15.10 | 1.25E-54 |
| ENSRNOG00000009920.5 | LOC680121 | 11:13499163-13501263 | 2.29E-57 | 7.37 | 1.50E-53 |
| ENSRNOG00000037352.3 | RGD1562747 | 8:69484173-69489891 | 1.02E-56 | 13.84 | 5.60E-53 |
| ENSRNOG00000021164.7 | Stip1 | 1:222274132-222293148 | 5.22E-48 | 5.37 | 2.45E-44 |
| ENSRNOG00000003147.8 | Sqstm1 | 10:35704729-35716294 | 9.13E-48 | 5.33 | 3.75E-44 |
| ENSRNOG00000011668.6 | Nfil3 | 17:12261101-12276315 | 2.09E-46 | 15.18 | 7.65E-43 |
| ENSRNOG00000015160.5 | Gem | 5:25349927-25353661 | 3.05E-44 | 9.95 | 1.00E-40 |
| ENSRNOG00000052142.1 | Ahsa2 | 14:108265206-108273938 | 2.18E-42 | 11.54 | 6.52E-39 |
| ENSRNOG00000059714.1 | Hsp90aa1 | 6:135107270-135112775 | 8.97E-41 | 11.35 | 2.46E-37 |
| ENSRNOG00000053577.1 | Gramd1b | 8:44165366-44327551 | 1.03E-40 | 4.05 | 2.61E-37 |
| ENSRNOG00000003597.5 | Tuba4a | 9:82415604-82419288 | 2.58E-40 | 11.76 | 6.06E-37 |
| ENSRNOG00000004019.3 | Phlda1 | 7:54247459-54249664 | 4.69E-38 | 8.29 | 1.03E-34 |
| ENSRNOG00000023546.5 | Hspb1 | 12:23839398-23841049 | 1.3E-37 | 28.13 | 2.62E-34 |
| ENSRNOG00000028155.6 | Dnaaf2 | 6:91481953-91490366 | 1.36E-37 | 22.92 | 2.62E-34 |
| ENSRNOG00000008709.6 | Arhgap32 | 8:33239138-33392305 | 2.1E-37 | 20.23 | 3.84E-34 |
| ENSRNOG00000050655.1 | P4ha1 | 20:28920615-28971966 | 2.64E-37 | 9.89 | 4.57E-34 |
| ENSRNOG00000021027.6 | Dbp | 1:101687854-101692846 | 8.97E-37 | -12.11 | 1.48E-33 |
| ENSRNOG00000004091.6 | Cwc25 | 10:85684897-85709568 | 1.16E-36 | 18.63 | 1.82E-33 |
| ENSRNOG00000032917.3 | Zfand2a | 12:17252092-17263477 | 7.04E-36 | 35.14 | 1.05E-32 |
| ENSRNOG00000017209.6 | Tubb3 | 19:56220754-56229813 | 7.8E-35 | 8.11 | 1.11E-31 |
| ENSRNOG00000000902.7 | Hsph1 | 12:6322667-6341902 | 8.44E-34 | 22.56 | 1.11E-30 |
| ENSRNOG00000002572.5 | Cacybp | 13:77948770-77959110 | 8.25E-34 | 4.61 | 1.11E-30 |
| ENSRNOG00000051563.1 | Giot1 | 7:13104480-13108723 | 2.73E-33 | 15.02 | 3.46E-30 |
| ENSRNOG00000018487.3 | Slc3a2 | 1:224906553-224921092 | 8.79E-33 | 3.33 | 1.07E-29 |
| ENSRNOG00000019834.6 | Hsp90ab1 | 9:17817720-17823243 | 1.12E-32 | 4.23 | 1.32E-29 |
| ENSRNOG00000006931.5 | Eepd1 | 8:26652120-26758325 | 1.8E-32 | -8.34 | 2.04E-29 |
| ENSRNOG00000048981.1 | Ahsa1 | 6:111296416-111304224 | 9.14E-32 | 3.07 | 1.00E-28 |
| ENSRNOG00000051180.1 | LOC100911516 | 9:101388150-101388833 | 1.77E-31 | 3.29 | 1.88E-28 |
| ENSRNOG00000027405.6 | Ccp110 | 1:188420460-188444991 | 2.73E-30 | 34.90 | 2.80E-27 |
| ENSRNOG00000059017.1 | AC106292.2 | 11:65743891-65745038 | 5.38E-30 | 5.96 | 5.36E-27 |
| ENSRNOG00000021475.6 | Ldah | 6:33407247-33491725 | 1.9E-29 | 9.78 | 1.83E-26 |
| ENSRNOG00000005698.2 | Ndufa5 | 4:51590412-51598771 | 4.42E-29 | 9.81 | 4.15E-26 |
| ENSRNOG00000045654.2 | LOC108348108 | 20:2699711-2701815 | 4.58E-29 | 81.31 | 4.18E-26 |
| ENSRNOG00000017212.7 | Spsb1 | 5:166913734-166926636 | 9.32E-29 | 4.98 | 8.29E-26 |
| ENSRNOG00000014338.6 | Slc25a25 | 3:11442396-11452529 | 1.22E-28 | 5.31 | 1.06E-25 |
| ENSRNOG00000007029.3 | Dnaja1 | 5:57028466-57039378 | 1.84E-28 | 9.24 | 1.55E-25 |
| ENSRNOG00000028711.3 | Dgat1 | 7:117566367-117576737 | 2.63E-28 | 11.07 | 2.16E-25 |
| ENSRNOG00000012559.6 | Man1b1 | 3:2547985-2569049 | 3.92E-28 | 3.75 | 3.15E-25 |
| ENSRNOG00000001198.4 | Fam222a | 12:47748164-47793534 | 5.62E-28 | 6.04 | 4.40E-25 |
| ENSRNOG00000013469.7 | Txndc5 | 17:26925827-26953970 | 6.26E-28 | 3.54 | 4.79E-25 |
| ENSRNOG00000000918.7 | Zbed5 | 12:30308468-30314519 | 1.81E-27 | 3.40 | 1.35E-24 |
| ENSRNOG00000005413.5 | Creb3l1 | 3:80892434-80933283 | 2.42E-27 | 20.41 | 1.77E-24 |
| ENSRNOG00000005660.4 | Fam110c | 6:49968472-49974728 | 1.56E-26 | 6.63 | 1.11E-23 |
| ENSRNOG00000012106.4 | Dnaja4 | 8:59278261-59294003 | 4.41E-26 | 22.41 | 3.09E-23 |
| ENSRNOG00000049270.1 | Ppp2r3b | 14:1469747-1476355 | 8.32E-26 | 8.33 | 5.70E-23 |
| ENSRNOG00000011222.5 | Dynll1 | 12:47074199-47076572 | 9.95E-26 | 3.67 | 6.68E-23 |
| ENSRNOG00000006444.6 | Fkbp4 | 4:161748992-161757447 | 3.26E-25 | 3.64 | 2.14E-22 |
| ENSRNOG00000014448.8 | Arntl | 1:178039062-178137465 | 2.67E-24 | 17.47 | 1.72E-21 |
| ENSRNOG00000021729.6 | Iqub | 4:51482169-51586881 | 4.39E-24 | 98.95 | 2.77E-21 |
| ENSRNOG00000016930.5 | Ube2s | 1:72580423-72584482 | 4.6E-24 | 2.79 | 2.85E-21 |
| ENSRNOG00000004132.7 | Lasp1 | 10:85744567-85785133 | 1.17E-23 | 4.63 | 7.15E-21 |
| ENSRNOG00000010178.6 | Cgrrf1 | 15:23619122-23639628 | 6.94E-23 | 5.36 | 4.15E-20 |
| ENSRNOG00000019018.6 | Plat | 16:74098259-74122889 | 8.85E-23 | 9.90 | 5.20E-20 |
| ENSRNOG00000019587.6 | Ptprn | 9:82446632-82461903 | 1.16E-22 | 8.43 | 6.70E-20 |
| ENSRNOG00000014900.8 | Crem | 17:57031765-57090888 | 6.97E-22 | 5.47 | 3.95E-19 |
| ENSRNOG00000009734.4 | Akr1b8 | 4:61771969-61828657 | 8.24E-22 | 4.77 | 4.59E-19 |
| ENSRNOG00000004726.7 | Mapkapk2 | 13:47785160-47831110 | 1.34E-21 | 4.50 | 7.34E-19 |
| ENSRNOG00000032585.5 | AABR07048992.1 | 5:99031012-99033107 | 1.76E-21 | 4.34 | 9.47E-19 |
| ENSRNOG00000028652.4 | Map10 | 19:58505810-58508603 | 4.8E-21 | 27.12 | 2.54E-18 |
| ENSRNOG00000002642.7 | Ptges3 | 7:2479310-2497013 | 6.24E-21 | 2.21 | 3.26E-18 |
| ENSRNOG00000003687.6 | Rgs2 | 13:60846307-60849094 | 8.66E-21 | 9.68 | 4.45E-18 |
| ENSRNOG00000027089.6 | Ell2 | 2:2456734-2524237 | 8.92E-21 | 7.95 | 4.51E-18 |
| ENSRNOG00000024349.6 | Cbarp | 7:12433932-12441048 | 1.63E-20 | 9.09 | 8.11E-18 |
| ENSRNOG00000034093.4 | AABR07011951.1 | 2:177651240-177653288 | 1.81E-20 | 4.01 | 8.87E-18 |
| ENSRNOG00000020480.5 | Fads1 | 1:226234073-226249138 | 3.8E-20 | -3.94 | 1.84E-17 |
| ENSRNOG00000000461.7 | LOC108348112 | 20:5262119-5269337 | 8.88E-20 | 4.66 | 4.23E-17 |
| ENSRNOG00000002480.7 | Gpr137b | 17:90670871-90746804 | 1.47E-19 | 4.55 | 6.92E-17 |
| ENSRNOG00000010170.5 | Tubb4b | 3:2441732-2444281 | 1.73E-19 | 3.38 | 8.03E-17 |
| ENSRNOG00000034066.5 | Hspa8 | 8:44990013-44993179 | 2.14E-19 | 11.76 | 9.75E-17 |
| ENSRNOG00000031216.3 | AABR07055776.1 | 7:9974624-9976367 | 4.75E-19 | 4.63 | 2.14E-16 |
| ENSRNOG00000001189.6 | Sik1 | 20:10668410-10680283 | 5.42E-19 | 7.29 | 2.41E-16 |
| ENSRNOG00000047072.2 | LOC100910656 | 7:116275816-116284587 | 7.28E-19 | 9.29 | 3.19E-16 |
| ENSRNOG00000014743.5 | Hagh | 10:14216154-14230515 | 1.17E-18 | 5.57 | 5.07E-16 |
| ENSRNOG00000033765.4 | Eif1 | 10:88227359-88229765 | 1.51E-18 | 2.25 | 6.45E-16 |
| ENSRNOG00000019466.7 | Agpat2 | 3:4044541-4055806 | 1.58E-18 | -3.88 | 6.65E-16 |
| ENSRNOG00000001030.8 | Tsc22d1 | 15:58554373-58658153 | 1.69E-18 | 2.68 | 7.02E-16 |
| ENSRNOG00000026643.5 | Chordc1 | 8:17421556-17446165 | 2.76E-18 | 5.06 | 1.14E-15 |
| ENSRNOG00000016831.6 | Serpinh1 | 1:164301009-164308317 | 2.85E-18 | 2.52 | 1.14E-15 |
| ENSRNOG00000004084.7 | Lratd1 | 6:39358984-39363458 | 3.1E-18 | 29.66 | 1.23E-15 |
| ENSRNOG00000010873.7 | Pithd1 | 5:154322550-154332940 | 3.39E-18 | 4.31 | 1.33E-15 |
| ENSRNOG00000013023.5 | Nop53 | 1:77836195-77844189 | 3.77E-18 | 2.53 | 1.46E-15 |
| ENSRNOG00000061128.1 | Pelo | 2:47266737-47268625 | 3.87E-18 | 3.44 | 1.48E-15 |
| ENSRNOG00000008487.7 | Amotl2 | 8:111209907-111226226 | 4.01E-18 | 4.16 | 1.51E-15 |
| ENSRNOG00000015408.5 | Vil1 | 9:81689801-81717621 | 6.84E-18 | 81.50 | 2.56E-15 |
| ENSRNOG00000027151.5 | Lrrc58 | 11:65747035-65759581 | 9.09E-18 | 8.55 | 3.36E-15 |
| ENSRNOG00000020063.5 | Nfkbib | 1:86941073-86948845 | 1.49E-17 | 3.69 | 5.43E-15 |
| ENSRNOG00000019661.3 | Gdf15 | 16:20555394-20557978 | 1.53E-17 | 9.19 | 5.54E-15 |
| ENSRNOG00000062247.1 | AABR07031521.1 | 18:17116551-17118501 | 1.75E-17 | 6.04 | 6.26E-15 |
| ENSRNOG00000000661.5 | Hps4 | 12:50285277-50314406 | 2.07E-17 | 7.40 | 7.31E-15 |
| ENSRNOG00000014287.6 | Stk11 | 7:12440750-12457513 | 2.63E-17 | 3.06 | 9.21E-15 |
| ENSRNOG00000010574.4 | Ptpn1 | 3:164665531-164711848 | 3.57E-17 | 4.02 | 1.24E-14 |
| ENSRNOG00000050647.2 | Hspa1b | 20:4877323-4879779 | 3.64E-17 | 70.52 | 1.25E-14 |
| ENSRNOG00000013654.4 | Cbln2 | 18:83777664-83782930 | 4.53E-17 | 11.87 | 1.54E-14 |
| ENSRNOG00000009642.3 | Cct4 | 14:107767391-107780270 | 5.21E-17 | 2.25 | 1.75E-14 |
| ENSRNOG00000053362.1 | Gabarapl1 | 4:163293723-163302858 | 6.45E-17 | 2.07 | 2.14E-14 |
| ENSRNOG00000011310.8 | Pde10a | 1:52360295-52544450 | 9.72E-17 | 11.93 | 3.20E-14 |
| ENSRNOG00000012886.4 | Maff | 7:120580742-120592095 | 1.17E-16 | 7.04 | 3.82E-14 |
| ENSRNOG00000009766.7 | Gpr180 | 15:103344475-103369630 | 1.29E-16 | 9.78 | 4.16E-14 |
| ENSRNOG00000011039.7 | Gch1 | 15:23934584-23969011 | 1.68E-16 | 3.65 | 5.36E-14 |
| ENSRNOG00000016596.3 | Hspa4 | 10:38601623-38642397 | 2.44E-16 | 2.63 | 7.72E-14 |
| ENSRNOG00000017801.7 | Atf4 | 7:121480722-121482772 | 3.86E-16 | 3.56 | 1.21E-13 |
| ENSRNOG00000007730.3 | Vom2r53 | 7:13151591-13184257 | 3.91E-16 | 128.91 | 1.21E-13 |
| ENSRNOG00000012942.6 | Satb1 | 9:2190914-2274629 | 6.68E-16 | 3.99 | 2.05E-13 |
| ENSRNOG00000000989.8 | Bud31 | 12:11232963-11240400 | 7.16E-16 | 2.43 | 2.18E-13 |
| ENSRNOG00000019598.8 | Vegfa | 9:17340340-17355681 | 7.85E-16 | 2.19 | 2.37E-13 |
| ENSRNOG00000049203.3 | Pex5 | 4:156983914-157009674 | 9.69E-16 | 4.18 | 2.90E-13 |
| ENSRNOG00000013328.8 | Rbpms | 16:61954589-62096497 | 1E-15 | 2.58 | 2.97E-13 |
| ENSRNOG00000011511.7 | Stk24 | 15:106629311-106678918 | 1.11E-15 | 3.65 | 3.26E-13 |
| ENSRNOG00000028103.5 | Psmd3 | 10:86599219-86611188 | 1.63E-15 | 2.46 | 4.74E-13 |
| ENSRNOG00000006331.4 | Elovl5 | 8:85259981-85285983 | 1.69E-15 | 3.56 | 4.87E-13 |
| ENSRNOG00000020552.2 | Fosl1 | 1:220826559-220835066 | 1.84E-15 | 26.04 | 5.26E-13 |
| ENSRNOG00000013712.7 | Tex261 | 4:115448707-115453659 | 1.95E-15 | 4.53 | 5.52E-13 |
| ENSRNOG00000034242.3 | Vcp | 5:58426548-58445953 | 2.95E-15 | 2.12 | 8.30E-13 |
| ENSRNOG00000019090.6 | Cct3 | 2:187668795-187693610 | 6.33E-15 | 2.13 | 1.76E-12 |
| ENSRNOG00000000503.7 | Ppard | 20:7818288-7885333 | 6.41E-15 | 3.57 | 1.77E-12 |
| ENSRNOG00000021719.7 | Slfn5 | 10:70298518-70312070 | 6.59E-15 | -5.64 | 1.81E-12 |
| ENSRNOG00000015421.6 | Slc27a3 | 2:189760746-189765415 | 8.02E-15 | -2.44 | 2.18E-12 |
| ENSRNOG00000020897.6 | Prpf19 | 1:226947104-226958144 | 8.42E-15 | -2.26 | 2.27E-12 |
| ENSRNOG00000005811.2 | LOC688655 | 6:34553366-34555306 | 8.75E-15 | 6.06 | 2.34E-12 |
| ENSRNOG00000007393.7 | Ndrg1 | 7:107734322-107775714 | 9.86E-15 | 5.09 | 2.61E-12 |
| ENSRNOG00000046484.1 | Ccdc160 | X:140175860-140176829 | 9.93E-15 | -6.43 | 2.61E-12 |
| ENSRNOG00000021131.6 | Mindy1 | 2:196462553-196470130 | 1.05E-14 | -5.22 | 2.73E-12 |
| ENSRNOG00000004791.5 | Arf6 | 6:91697108-91698255 | 1.07E-14 | 2.47 | 2.77E-12 |
| ENSRNOG00000010833.5 | Mthfd2 | 4:115004447-115015965 | 1.21E-14 | 2.90 | 3.08E-12 |
| ENSRNOG00000019253.8 | Bcar1 | 19:43932553-43955783 | 1.27E-14 | 3.06 | 3.21E-12 |
| ENSRNOG00000017250.4 | Gmpr | 17:19543274-19580929 | 1.43E-14 | 3.73 | 3.59E-12 |
| ENSRNOG00000013090.7 | Gadd45g | 17:13391466-13393243 | 1.8E-14 | 7.37 | 4.47E-12 |
| ENSRNOG00000037580.2 | Psmd8 | 1:88116811-88123643 | 2E-14 | 2.26 | 4.94E-12 |
| ENSRNOG00000019070.4 | St13 | 7:122585770-122635731 | 2.26E-14 | 2.30 | 5.55E-12 |
| ENSRNOG00000018393.4 | Uts2 | 5:168078747-168084145 | 2.44E-14 | 38.27 | 5.94E-12 |
| ENSRNOG00000021198.4 | Hist1h2bq | 17:43679924-43689311 | 2.59E-14 | 2.42 | 6.26E-12 |
| ENSRNOG00000050800.2 | LOC100911576 | 14:88543629-88574203 | 3.17E-14 | 2.57 | 7.62E-12 |
| ENSRNOG00000058882.1 | Emd | X:156452817-156456035 | 3.64E-14 | 3.70 | 8.67E-12 |
| ENSRNOG00000017309.7 | Rsrp1 | 5:153260929-153264669 | 5.22E-14 | 2.52 | 1.23E-11 |
| ENSRNOG00000009358.6 | Osgin2 | 5:29663684-29682005 | 5.32E-14 | 5.65 | 1.25E-11 |
| ENSRNOG00000046005.1 | Scd2 | 1:264059373-264072433 | 5.93E-14 | -2.69 | 1.38E-11 |
| ENSRNOG00000000981.8 | Scarb1 | 12:36694959-36761455 | 6.01E-14 | 3.79 | 1.39E-11 |
| ENSRNOG00000018818.6 | Txnl1 | 18:59059374-59086256 | 6.36E-14 | 2.06 | 1.46E-11 |
| ENSRNOG00000001172.7 | Rnf10 | 12:47103313-47138128 | 7.42E-14 | 2.55 | 1.7E-11 |
| ENSRNOG00000058601.1 | AABR07036542.1 | 12:47842420-47847165 | 7.82E-14 | 8.13 | 1.77E-11 |
| ENSRNOG00000019459.5 | Oaz1 | 7:11752411-11754508 | 7.99E-14 | 2.58 | 1.80E-11 |
| ENSRNOG00000014204.7 | Pacsin3 | 3:80072488-80081047 | 1.08E-13 | -3.71 | 2.41E-11 |
| ENSRNOG00000059660.1 | AABR07065531.26 | 6:133667974-133676907 | 1.1E-13 | 4.70 | 2.45E-11 |
| ENSRNOG00000020298.6 | Bag3 | 1:199941160-199965191 | 1.24E-13 | 13.50 | 2.75E-11 |
| ENSRNOG00000020717.4 | Bod1 | 10:16259727-16266061 | 1.25E-13 | 3.00 | 2.75E-11 |
| ENSRNOG00000055564.1 | RGD1564664 | 3:82854776-82856171 | 1.28E-13 | 6.52 | 2.79E-11 |
| ENSRNOG00000011927.7 | Sdc3 | 5:148923097-148956404 | 1.48E-13 | -3.71 | 3.21E-11 |
| ENSRNOG00000047250.2 | Gmfb | 15:23601367-23606634 | 1.98E-13 | 3.20 | 4.25E-11 |
| ENSRNOG00000061040.1 | Herc4 | 20:26755910-26830931 | 2.23E-13 | 3.05 | 4.77E-11 |
| ENSRNOG00000014727.6 | Fahd1 | 10:14214518-14215957 | 2.92E-13 | 3.37 | 6.21E-11 |
| ENSRNOG00000059268.2 | Ppp1r10 | 20:3329676-3344286 | 3.13E-13 | 3.62 | 6.59E-11 |
| ENSRNOG00000006756.8 | Maged1 | X:63803188-63809861 | 5.03E-13 | 3.18 | 1.05E-10 |
| ENSRNOG00000019978.4 | Parg | 16:8350325-8457996 | 6.09E-13 | 3.48 | 1.27E-10 |
| ENSRNOG00000009850.5 | St3gal4 | 8:36264749-36314811 | 7.7E-13 | 2.03 | 1.59E-10 |
| ENSRNOG00000019069.4 | Nxf1 | 1:224957516-224970727 | 8.2E-13 | 2.33 | 1.69E-10 |
| ENSRNOG00000019536.5 | Smim3 | 18:55744809-55771730 | 8.52E-13 | 2.50 | 1.74E-10 |
| ENSRNOG00000010407.6 | LOC103690024 | 4:156403015-156427755 | 9.34E-13 | 3.52 | 1.90E-10 |
| ENSRNOG00000005615.6 | Gadd45a | 4:97782511-97784842 | 1.25E-12 | 7.85 | 2.52E-10 |
| ENSRNOG00000016374.8 | Fgfr2 | 1:200590952-200696928 | 1.43E-12 | 2.64 | 2.86E-10 |
| ENSRNOG00000007607.4 | Nr4a1 | 7:142905757-142920216 | 1.64E-12 | 2.40 | 3.26E-10 |
| ENSRNOG00000056596.1 | Alas1 | 8:114927721-114940177 | 1.86E-12 | 2.51 | 3.68E-10 |
| ENSRNOG00000000923.8 | Cct6a | 12:30491415-30501860 | 1.99E-12 | 2.10 | 3.92E-10 |
| ENSRNOG00000014525.5 | Hspd1 | 9:61680529-61690956 | 2.19E-12 | 2.04 | 4.29E-10 |
| ENSRNOG00000000304.7 | Cd164 | 20:46250362-46261994 | 2.27E-12 | 2.02 | 4.41E-10 |
| ENSRNOG00000056651.1 | LOC103690068 | 1:88192369-88193346 | 2.55E-12 | 9.55 | 4.92E-10 |
| ENSRNOG00000038999.4 | RT1-A1 | 20:5351604-5421098 | 3.82E-12 | 4.13 | 7.35E-10 |
| ENSRNOG00000011771.3 | Ptp4a1 | 9:37223728-37231291 | 3.99E-12 | 3.14 | 7.63E-10 |
| ENSRNOG00000002393.7 | Eprs | 13:103300931-103371577 | 4.12E-12 | 4.95 | 7.83E-10 |
| ENSRNOG00000000036.6 | Klhdc8a | 13:49074643-49082729 | 4.44E-12 | -2.10 | 8.39E-10 |
| ENSRNOG00000000250.6 | Jmjd6 | 10:105781752-105787803 | 6.28E-12 | 3.97 | 1.18E-09 |
| ENSRNOG00000048682.2 | Zwint | 20:17075770-17091766 | 6.95E-12 | 2.02 | 1.30E-09 |
| ENSRNOG00000002461.7 | Nid1 | 17:90553393-90627101 | 7.94E-12 | 3.54 | 1.47E-09 |
| ENSRNOG00000021262.5 | Slc23a2 | 3:124632491-124724252 | 7.9E-12 | 3.23 | 1.47E-09 |
| ENSRNOG00000019622.3 | ACTr3 | 9:97355923-97367445 | 8.43E-12 | 5.69 | 1.55E-09 |
| ENSRNOG00000055984.1 | Adrm1 | 3:175548173-175552947 | 8.83E-12 | 2.23 | 1.61E-09 |
| ENSRNOG00000060728.1 | Tuba1a | 7:140637286-140640953 | 9.1E-12 | 3.73 | 1.65E-09 |
| ENSRNOG00000004870.5 | Dyrk3 | 13:47906230-47916877 | 1.15E-11 | 5.13 | 2.07E-09 |
| ENSRNOG00000012760.8 | Chtop | 2:189887856-189899325 | 1.5E-11 | 2.29 | 2.68E-09 |
| ENSRNOG00000019383.7 | Tef | 7:123043502-123058604 | 1.54E-11 | -5.14 | 2.75E-09 |
| ENSRNOG00000010716.3 | Atoh8 | 4:100067340-100099517 | 1.63E-11 | 7.10 | 2.87E-09 |
| ENSRNOG00000030478.3 | AY172581.9 | MT:67-1025 | 1.88E-11 | 2.40 | 3.30E-09 |
| ENSRNOG00000006766.6 | Laptm4b | 7:72924798-72968101 | 2.22E-11 | 2.12 | 3.87E-09 |
| ENSRNOG00000000827.6 | Ier3 | 20:3438797-3440769 | 2.38E-11 | 2.78 | 4.10E-09 |
| ENSRNOG00000020433.7 | Actn4 | 1:87078019-87147333 | 2.44E-11 | 2.02 | 4.19E-09 |
| ENSRNOG00000004284.4 | Btg1 | 7:37812830-37815088 | 2.51E-11 | 3.19 | 4.28E-09 |
| ENSRNOG00000016189.8 | Frmd5 | 3:113483268-113525864 | 2.68E-11 | 2.79 | 4.54E-09 |
| ENSRNOG00000018214.6 | Bok | 9:100829551-100840504 | 3.19E-11 | 2.63 | 5.34E-09 |
| ENSRNOG00000016356.7 | Got1 | 1:263246247-263269762 | 3.3E-11 | 2.45 | 5.50E-09 |
| ENSRNOG00000001201.4 | Cstb | 20:10966330-10968432 | 3.44E-11 | 2.49 | 5.72E-09 |
| ENSRNOG00000021812.4 | Scx | 7:117519074-117521093 | 3.73E-11 | 11.52 | 6.16E-09 |
| ENSRNOG00000046207.1 | Cbx4 | 10:108190994-108196217 | 3.98E-11 | 2.64 | 6.54E-09 |
| ENSRNOG00000053468.1 | Tuba1b | 7:140614751-140617721 | 4.11E-11 | 3.45 | 6.72E-09 |
| ENSRNOG00000025338.7 | Msi2 | 10:75681392-76039964 | 5.67E-11 | 2.95 | 9.24E-09 |
| ENSRNOG00000016460.7 | Clu | 15:42640145-42665857 | 5.85E-11 | 2.93 | 9.47E-09 |
| ENSRNOG00000003228.5 | Mid1ip1 | X:13114568-13116743 | 7.56E-11 | -2.87 | 1.21E-08 |
| ENSRNOG00000020340.7 | Brd8 | 18:27398687-27424090 | 7.89E-11 | 3.45 | 1.26E-08 |
| ENSRNOG00000040287.4 | Cyp1b1 | 6:2307807-2316722 | 1.13E-10 | 2.03 | 1.77E-08 |
| ENSRNOG00000009088.7 | Txnrd1 | 7:26946124-26984400 | 1.19E-10 | 2.63 | 1.86E-08 |
| ENSRNOG00000013413.7 | Rorb | 1:234252756-234435839 | 1.19E-10 | 25.57 | 1.86E-08 |
| ENSRNOG00000001843.5 | Bcl6 | 11:80255789-80279075 | 1.37E-10 | 12.40 | 2.12E-08 |
| ENSRNOG00000012872.7 | Carnmt1 | 1:234677691-234704792 | 1.4E-10 | 3.51 | 2.15E-08 |
| ENSRNOG00000014117.7 | Hmox1 | 19:14508615-14515456 | 1.43E-10 | 5.19 | 2.18E-08 |
| ENSRNOG00000000906.7 | Medag | 12:6711746-6740714 | 1.63E-10 | -6.24 | 2.48E-08 |
| ENSRNOG00000002911.5 | Alb | 14:19176276-19191863 | 1.71E-10 | 5.57 | 2.59E-08 |
| ENSRNOG00000007573.4 | Hoxb9 | 10:84119883-84123390 | 1.83E-10 | 3.22 | 2.76E-08 |
| ENSRNOG00000042838.3 | Junb | 19:26092973-26094756 | 1.85E-10 | 4.34 | 2.78E-08 |
| ENSRNOG00000018109.4 | Clic4 | 5:153568744-153625869 | 2.09E-10 | 5.08 | 3.13E-08 |
| ENSRNOG00000009437.7 | Ewsr1 | 14:85322295-85350948 | 2.21E-10 | 2.08 | 3.29E-08 |
| ENSRNOG00000002520.5 | Litaf | 10:4719712-4763510 | 2.45E-10 | 2.06 | 3.63E-08 |
| ENSRNOG00000057696.1 | Sbk1 | 1:197659186-197681006 | 2.61E-10 | -4.45 | 3.86E-08 |
| ENSRNOG00000054446.1 | Sltm | 8:76977821-77022837 | 2.85E-10 | 2.58 | 4.18E-08 |
| ENSRNOG00000025701.5 | Nifk | 13:34267470-34277530 | 4.11E-10 | 2.59 | 5.97E-08 |
| ENSRNOG00000047706.3 | LOC103690108 | 20:3791406-3794027 | 4.18E-10 | 3.80 | 6.05E-08 |
| ENSRNOG00000002413.6 | Gpc4 | X:139354154-139464798 | 4.84E-10 | 2.13 | 6.98E-08 |
| ENSRNOG00000023509.6 | Irs2 | 16:83824429-83848684 | 5.52E-10 | 3.26 | 7.90E-08 |
| ENSRNOG00000011704.5 | Fbxo34 | 15:24254041-24334890 | 5.61E-10 | 4.45 | 7.99E-08 |
| ENSRNOG00000002408.6 | Rbm47 | 14:43810118-43837662 | 6.24E-10 | 3.60 | 8.77E-08 |
| ENSRNOG00000017558.6 | Tubb2a | 17:31493106-31498651 | 6.24E-10 | 2.71 | 8.77E-08 |
| ENSRNOG00000047896.2 | Kctd10 | 12:47992090-48010583 | 7.13E-10 | 2.47 | 9.97E-08 |
| ENSRNOG00000002470.8 | Ifi47 | 10:34277992-34285623 | 8.04E-10 | -2.69 | 1.12E-07 |
| ENSRNOG00000016595.5 | Hhex | 1:256101902-256107649 | 8.29E-10 | 3.05 | 1.15E-07 |
| ENSRNOG00000018107.5 | Zfand5 | 1:238843337-238852566 | 8.61E-10 | 2.22 | 1.19E-07 |
| ENSRNOG00000003538.7 | Adamts4 | 13:89622631-89634419 | 8.86E-10 | 79.29 | 1.22E-07 |
| ENSRNOG00000015434.7 | Midn | 7:12414732-12424367 | 9.07E-10 | 2.73 | 1.24E-07 |
| ENSRNOG00000018003.6 | F2rl1 | 2:25222327-25235275 | 9.14E-10 | 27.45 | 1.25E-07 |
| ENSRNOG00000023257.5 | Adamts9 | 4:124660261-124858250 | 9.76E-10 | 5.25 | 1.33E-07 |
| ENSRNOG00000059770.1 | AABR07044383.1 | 20:3791628-3796275 | 1.02E-09 | 10.11 | 1.38E-07 |
| ENSRNOG00000057078.1 | Ddit4 | 20:29509288-29511382 | 1.12E-09 | 5.87 | 1.51E-07 |
| ENSRNOG00000025443.4 | Map1lc3a | 3:150801288-150802935 | 1.23E-09 | 3.26 | 1.66E-07 |
| ENSRNOG00000015999.6 | Cirbp | 7:12401206-12405022 | 1.3E-09 | -3.49 | 1.74E-07 |
| ENSRNOG00000019996.5 | Slc16a1 | 2:207108551-207128554 | 1.36E-09 | 3.15 | 1.80E-07 |
| ENSRNOG00000031662.4 | Slc6a5 | 1:105284752-105336363 | 1.36E-09 | 2.39 | 1.80E-07 |
| ENSRNOG00000020929.5 | Lin37 | 1:89010256-89014189 | 1.5E-09 | 2.54 | 1.98E-07 |
| ENSRNOG00000009811.6 | Cnih1 | 15:23568554-23580395 | 1.59E-09 | 2.19 | 2.08E-07 |
| ENSRNOG00000030447.5 | AC105662.1 | 5:173148883-173149276 | 1.73E-09 | 9.72 | 2.25E-07 |
| ENSRNOG00000021118.5 | Nomo1 | 1:102017262-102068870 | 2.28E-09 | 2.67 | 2.95E-07 |
| ENSRNOG00000000700.4 | Tmem119 | 12:48598646-48605704 | 2.85E-09 | 3.53 | 3.68E-07 |
| ENSRNOG00000032669.5 | Serpina1 | 6:127610242-127632265 | 3.4E-09 | 4.96 | 4.35E-07 |
| ENSRNOG00000007763.7 | Plod1 | 5:164720586-164747083 | 3.77E-09 | 2.80 | 4.79E-07 |
| ENSRNOG00000058461.1 | Sfpq | 5:145079802-145088498 | 4.32E-09 | 2.52 | 5.44E-07 |
| ENSRNOG00000023376.4 | Riok3 | 18:3565165-3590739 | 4.42E-09 | 2.37 | 5.55E-07 |
| ENSRNOG00000042592.2 | Rgs10 | 1:199782077-199823386 | 4.46E-09 | 2.94 | 5.58E-07 |
| ENSRNOG00000048308.2 | Dr1 | 14:2463642-2474780 | 4.58E-09 | -7.60 | 5.70E-07 |
| ENSRNOG00000020938.7 | Ppp1r15a | 1:101511900-101514974 | 4.73E-09 | 2.76 | 5.86E-07 |
| ENSRNOG00000032487.4 | Sts | X:45420595-45428750 | 5.1E-09 | 2.40 | 6.30E-07 |
| ENSRNOG00000009700.5 | Dync1i1 | 4:30807878-31121090 | 5.61E-09 | 2.56 | 6.91E-07 |
| ENSRNOG00000023465.4 | Depp1 | 4:148782478-148784562 | 5.86E-09 | 3.66 | 7.19E-07 |
| ENSRNOG00000009946.7 | Ldlr | 8:22750335-22774903 | 6.77E-09 | 2.10 | 8.24E-07 |
| ENSRNOG00000016989.3 | Dolk | 3:8850153-8852192 | 7.08E-09 | -2.93 | 8.60E-07 |
| ENSRNOG00000000576.6 | Anapc16 | 20:29544628-29558321 | 7.18E-09 | 2.73 | 8.68E-07 |
| ENSRNOG00000009205.6 | Lmo4 | 2:250218640-250241673 | 8.46E-09 | 2.98 | 1.02E-06 |
| ENSRNOG00000008297.5 | Oser1 | 3:159784127-159802952 | 1.02E-08 | 2.56 | 1.21E-06 |
| ENSRNOG00000013408.5 | Npas2 | 9:45901740-46081880 | 1.07E-08 | 8.64 | 1.27E-06 |
| ENSRNOG00000021824.5 | Dnajb1 | 19:24747177-24750919 | 1.1E-08 | 18.90 | 1.29E-06 |
| ENSRNOG00000036697.3 | Mafg | 10:109806158-109811323 | 1.1E-08 | 2.71 | 1.29E-06 |
| ENSRNOG00000052730.1 | Psmb3 | 10:85646632-85653729 | 1.13E-08 | 2.03 | 1.32E-06 |
| ENSRNOG00000013179.5 | Tinagl1 | 5:148382705-148392697 | 1.2E-08 | 2.23 | 1.39E-06 |
| ENSRNOG00000019050.4 | Ifit1 | 1:252944102-252946170 | 1.29E-08 | -14.73 | 1.49E-06 |
| ENSRNOG00000010524.6 | Cryab | 8:55178288-55182545 | 1.4E-08 | 3.80 | 1.61E-06 |
| ENSRNOG00000046171.3 | AABR07035839.1 | 12:24590644-24619639 | 1.4E-08 | 6.53 | 1.61E-06 |
| ENSRNOG00000005292.7 | Trip11 | 6:125742795-125812517 | 1.45E-08 | 6.09 | 1.66E-06 |
| ENSRNOG00000015699.7 | Lman2l | 9:42999228-43022999 | 1.51E-08 | 2.47 | 1.72E-06 |
| ENSRNOG00000007817.5 | Kctd6 | 15:18484509-18493366 | 1.6E-08 | 4.60 | 1.81E-06 |
| ENSRNOG00000018994.5 | Psmc4 | 1:84978205-84986581 | 1.73E-08 | 2.01 | 1.95E-06 |
| ENSRNOG00000058646.1 | Zfp36l1 | 6:103308044-103313074 | 1.87E-08 | 2.15 | 2.08E-06 |
| ENSRNOG00000032922.7 | Dclk1 | 2:144646307-144936927 | 1.88E-08 | 6.04 | 2.09E-06 |
| ENSRNOG00000003177.7 | Mat2b | 10:25832071-25847994 | 1.97E-08 | -2.64 | 2.18E-06 |
| ENSRNOG00000003551.5 | B4galt3 | 13:89643880-89649906 | 1.99E-08 | 3.98 | 2.19E-06 |
| ENSRNOG00000010872.5 | CTb | 6:136142955-136145837 | 2.22E-08 | 2.26 | 2.45E-06 |
| ENSRNOG00000000521.4 | Cdkn1a | 20:6351457-6358864 | 2.36E-08 | 5.95 | 2.58E-06 |
| ENSRNOG00000019202.8 | Pvr | 1:80820049-80835701 | 2.48E-08 | 4.97 | 2.7E-06 |
| ENSRNOG00000032902.3 | Ybx1-ps3 | 10:40380387-40381886 | 2.95E-08 | 2.06 | 3.18E-06 |
| ENSRNOG00000059589.1 | Lmbr1 | 4:2116093-2201749 | 3.26E-08 | 7.65 | 3.50E-06 |
| ENSRNOG00000019831.5 | Samd4b | 1:85324042-85362241 | 3.62E-08 | 2.84 | 3.88E-06 |
| ENSRNOG00000020975.7 | Mrpl49 | 1:221417335-221420115 | 3.66E-08 | 2.13 | 3.89E-06 |
| ENSRNOG00000027145.6 | Rora | 8:75516903-75607212 | 3.66E-08 | 4.52 | 3.89E-06 |
| ENSRNOG00000017748.6 | Nkx6-2 | 1:211922388-211923929 | 3.8E-08 | 9.00 | 4.03E-06 |
| ENSRNOG00000009639.5 | Zrsr1 | 14:107750161-107751892 | 3.91E-08 | 3.15 | 4.12E-06 |
| ENSRNOG00000015568.6 | Plpp5 | 16:71121174-71125316 | 4.85E-08 | 2.55 | 5.05E-06 |
| ENSRNOG00000003515.6 | Ephx1 | 13:99271365-99300579 | 4.96E-08 | -2.73 | 5.12E-06 |
| ENSRNOG00000019949.4 | Mrps12 | 1:86920679-86923575 | 4.96E-08 | 2.28 | 5.12E-06 |
| ENSRNOG00000016275.4 | Ttr | 18:15532962-15540177 | 5.87E-08 | 6.41 | 6.02E-06 |
| ENSRNOG00000031090.6 | RT1-CE7 | 20:4694471-4896970 | 5.94E-08 | 2.02 | 6.07E-06 |
| ENSRNOG00000019822.4 | Gadd45b | 7:11646268-11648322 | 6.32E-08 | 3.63 | 6.44E-06 |
| ENSRNOG00000000909.8 | Uspl1 | 12:6929977-6956984 | 6.99E-08 | 3.01 | 7.10E-06 |
| ENSRNOG00000016326.5 | Cx3cl1 | 19:10644243-10653800 | 7.09E-08 | -3.69 | 7.18E-06 |
| ENSRNOG00000031053.3 | Mt-nd4l | MT:9869-10166 | 8.07E-08 | -2.09 | 8.11E-06 |
| ENSRNOG00000047396.2 | Rmnd5b | 10:34979922-34990943 | 8.06E-08 | -8.04 | 8.11E-06 |
| ENSRNOG00000049402.2 | Nbl1 | 5:157524512-157535664 | 8.86E-08 | 2.18 | 8.85E-06 |
| ENSRNOG00000004139.8 | Ndel1 | 10:55411900-55444861 | 9E-08 | 2.29 | 8.97E-06 |
| ENSRNOG00000018783.6 | Bcas2 | 2:205662587-205670511 | 9.15E-08 | -2.36 | 9.09E-06 |
| ENSRNOG00000001130.8 | Nos1 | 12:44213942-44520341 | 9.67E-08 | 4.89 | 9.58E-06 |
| ENSRNOG00000031612.3 | Gls2 | 7:2605718-2621852 | 9.75E-08 | 3.63 | 9.63E-06 |
| ENSRNOG00000012582.5 | Eif4ebp1 | 16:68954874-68968248 | 1.08E-07 | -2.41 | 1.06E-05 |
| ENSRNOG00000002387.5 | Tceal1 | X:107370430-107372338 | 1.08E-07 | -2.62 | 1.06E-05 |
| ENSRNOG00000024428.4 | Kif20a | 18:27424327-27432814 | 1.09E-07 | 7.20 | 1.07E-05 |
| ENSRNOG00000032708.5 | RT1-Bb | 20:4039412-4049711 | 1.1E-07 | 2.64 | 1.07E-05 |
| ENSRNOG00000005670.5 | Art4 | 4:170830850-170841187 | 1.1E-07 | -5.01 | 1.07E-05 |
| ENSRNOG00000008274.3 | Xpc | 4:123134456-123161985 | 1.12E-07 | -4.26 | 1.08E-05 |
| ENSRNOG00000054945.1 | AABR07015081.2 | 14:46653039-46657975 | 1.14E-07 | 12.18 | 1.10E-05 |
| ENSRNOG00000036696.4 | Hgs | 10:109638766-109657456 | 1.14E-07 | 2.32 | 1.10E-05 |
| ENSRNOG00000016483.6 | Myo16 | 16:84575149-84939323 | 1.33E-07 | 10.54 | 1.27E-05 |
| ENSRNOG00000017506.7 | Cltb | 17:10537364-10554989 | 1.33E-07 | 2.02 | 1.28E-05 |
| ENSRNOG00000049814.2 | LOC100910882 | 3:152226709-152259156 | 1.34E-07 | 13.02 | 1.28E-05 |
| ENSRNOG00000006420.6 | Rbm38 | 3:171037956-171050475 | 1.38E-07 | 5.23 | 1.31E-05 |
| ENSRNOG00000021314.4 | Fdft1 | 15:46339248-46367302 | 1.41E-07 | -2.76 | 1.33E-05 |
| ENSRNOG00000000506.7 | Tead3 | 20:7908225-7930929 | 1.5E-07 | 3.79 | 1.41E-05 |
| ENSRNOG00000017911.8 | Tcaf1 | 4:72124233-72143748 | 1.61E-07 | -2.96 | 1.50E-05 |
| ENSRNOG00000033803.5 | LOC100360841 | 9:69790830-69791196 | 1.62E-07 | -2.52 | 1.50E-05 |
| ENSRNOG00000018183.7 | Ubr4 | 5:157848205-157956132 | 1.69E-07 | 2.74 | 1.56E-05 |
| ENSRNOG00000010096.7 | Azi2 | 8:126411828-126434579 | 1.71E-07 | 2.49 | 1.57E-05 |
| ENSRNOG00000052498.1 | Grb14 | 3:50937668-51054378 | 1.78E-07 | -3.25 | 1.63E-05 |
| ENSRNOG00000000891.5 | AABR07035107.1 | 12:4737816-4741997 | 1.79E-07 | -4.19 | 1.63E-05 |
| ENSRNOG00000001700.7 | Whrn | 5:79234949-79317206 | 1.86E-07 | 2.35 | 1.69E-05 |
| ENSRNOG00000021984.6 | Rgs7 | 13:93095199-93307199 | 1.89E-07 | 6.62 | 1.72E-05 |
| ENSRNOG00000005538.7 | Psmd11 | 10:67810654-67857223 | 1.92E-07 | 2.34 | 1.73E-05 |
| ENSRNOG00000004693.8 | Pbx1 | 13:86390740-86451002 | 1.96E-07 | 2.60 | 1.76E-05 |
| ENSRNOG00000030091.6 | Dusp14 | 10:71363687-71383602 | 1.96E-07 | 3.02 | 1.76E-05 |
| ENSRNOG00000001525.7 | Igsf11 | 11:64286693-64421248 | 2.02E-07 | -2.47 | 1.81E-05 |
| ENSRNOG00000008015.5 | Fos | 6:109300432-109303299 | 2.28E-07 | 4.65 | 2.03E-05 |
| ENSRNOG00000004608.5 | Pam16 | 10:11146358-11153936 | 2.49E-07 | 2.20 | 2.20E-05 |
| ENSRNOG00000005248.6 | Slc1a4 | 14:104581189-104612597 | 2.49E-07 | 8.01 | 2.21E-05 |
| ENSRNOG00000049828.1 | Crlf2 | 14:1456843-1461543 | 2.52E-07 | 2.11 | 2.23E-05 |
| ENSRNOG00000018812.3 | Rpp25 | 8:62283335-62284716 | 2.64E-07 | -5.22 | 2.32E-05 |
| ENSRNOG00000003104.7 | Trpv2 | 10:48903539-48925030 | 2.65E-07 | -3.02 | 2.33E-05 |
| ENSRNOG00000018484.5 | Plk3 | 5:135997724-136002900 | 2.78E-07 | 2.64 | 2.43E-05 |
| ENSRNOG00000009549.4 | Fbxo3 | 3:93968854-94000863 | 2.82E-07 | -3.20 | 2.45E-05 |
| ENSRNOG00000020608.5 | Ppan | 8:21905864-21909842 | 2.9E-07 | 2.21 | 2.51E-05 |
| ENSRNOG00000031965.4 | Impdh2 | 8:117347028-117351610 | 2.99E-07 | 2.01 | 2.58E-05 |
| ENSRNOG00000001277.4 | Mafk | 12:16923989-16934706 | 3.08E-07 | 2.66 | 2.65E-05 |
| ENSRNOG00000003977.5 | Dusp1 | 10:16970625-16973418 | 3.08E-07 | 4.29 | 2.65E-05 |
| ENSRNOG00000033663.5 | P4ha2 | 10:39435226-39464188 | 3.16E-07 | 2.31 | 2.70E-05 |
| ENSRNOG00000013541.5 | Sh2d4a | 16:23083953-23156962 | 3.28E-07 | -3.12 | 2.80E-05 |
| ENSRNOG00000010984.6 | Anxa11 | 16:3867207-3912148 | 3.5E-07 | 2.16 | 2.97E-05 |
| ENSRNOG00000028368.7 | Etnk2 | 13:50481162-50499140 | 3.61E-07 | -2.46 | 3.05E-05 |
| ENSRNOG00000052894.1 | Epg5 | 18:74299930-74395547 | 3.78E-07 | 3.81 | 3.17E-05 |
| ENSRNOG00000015496.6 | Tpm4 | 16:19385735-19399903 | 3.79E-07 | 2.11 | 3.18E-05 |
| ENSRNOG00000019559.5 | Eaf1 | 16:7588216-7602966 | 3.88E-07 | 2.83 | 3.23E-05 |
| ENSRNOG00000012100.6 | Ssbp1 | 4:68634928-68645172 | 4.23E-07 | -2.58 | 3.51E-05 |
| ENSRNOG00000008510.6 | Abtb2 | 3:93495105-93647721 | 4.26E-07 | 7.00 | 3.53E-05 |
| ENSRNOG00000014625.4 | Atp5f1d | 7:12426808-12432130 | 4.44E-07 | 2.10 | 3.67E-05 |
| ENSRNOG00000050869.2 | Cebpd | 11:89008007-89009146 | 4.67E-07 | 3.32 | 3.85E-05 |
| ENSRNOG00000010624.6 | Ap3b1 | 2:24024790-24226224 | 4.81E-07 | 2.28 | 3.95E-05 |
| ENSRNOG00000013948.5 | Zc3hav1 | 4:66011914-66062089 | 4.81E-07 | 2.15 | 3.95E-05 |
| ENSRNOG00000019189.7 | Acat2 | 1:47972398-47992653 | 5.46E-07 | 2.05 | 4.45E-05 |
| ENSRNOG00000007544.5 | Il23r | 4:98203957-98305173 | 6.14E-07 | 6.58 | 4.98E-05 |
| ENSRNOG00000013663.6 | Tmem86a | 1:103172986-103177417 | 6.46E-07 | -3.77 | 5.21E-05 |
| ENSRNOG00000004500.5 | Myc | 7:102586312-102591240 | 6.6E-07 | 2.62 | 5.30E-05 |
| ENSRNOG00000011544.7 | Zfp219 | 15:28402414-28406046 | 6.74E-07 | 2.28 | 5.40E-05 |
| ENSRNOG00000048152.2 | Myo1b | 9:54558201-54766054 | 7.14E-07 | 2.89 | 5.71E-05 |
| ENSRNOG00000042728.2 | Fbrs | 1:198932869-198937750 | 7.77E-07 | 2.37 | 6.17E-05 |
| ENSRNOG00000002232.6 | Aff1 | 14:7226585-7384876 | 8.05E-07 | 8.17 | 6.37E-05 |
| ENSRNOG00000030667.6 | Ppm1b | 6:8218695-8280124 | 8.06E-07 | 2.21 | 6.37E-05 |
| ENSRNOG00000020225.5 | Dlgap4 | 3:152752090-152846118 | 8.14E-07 | 2.72 | 6.40E-05 |
| ENSRNOG00000062039.1 | AABR07044420.2 | 20:5347665-5347939 | 9.29E-07 | 3.85 | 7.28E-05 |
| ENSRNOG00000028616.6 | PCT1 | 3:171213935-171219871 | 9.37E-07 | 2.96 | 7.32E-05 |
| ENSRNOG00000009887.7 | Arih1 | 8:64166359-64268555 | 9.44E-07 | 2.57 | 7.35E-05 |
| ENSRNOG00000010819.6 | Hspa4l | 2:127625682-127677503 | 9.64E-07 | 4.42 | 7.49E-05 |
| ENSRNOG00000016281.5 | Col4a1 | 16:83522161-83632153 | 9.66E-07 | 2.21 | 7.50E-05 |
| ENSRNOG00000036698.4 | Nploc4 | 10:109552648-109610030 | 1.03E-06 | 2.21 | 8.00E-05 |
| ENSRNOG00000014155.7 | Spart | 2:144522071-144548917 | 1.07E-06 | -3.15 | 8.28E-05 |
| ENSRNOG00000001203.7 | Rrp1 | 20:10981997-10993280 | 1.08E-06 | 2.04 | 8.30E-05 |
| ENSRNOG00000010896.5 | Tprn | 3:2480231-2487710 | 1.11E-06 | 3.32 | 8.49E-05 |
| ENSRNOG00000056457.1 | Gpd1 | 7:141370490-141377928 | 1.23E-06 | -2.53 | 9.40E-05 |
| ENSRNOG00000006086.7 | Lynx1 | 7:115982875-115988090 | 1.25E-06 | 2.32 | 9.57E-05 |
| ENSRNOG00000013777.3 | Rnf166 | 19:55290562-55300403 | 1.26E-06 | -2.72 | 9.60E-05 |
| ENSRNOG00000033169.5 | Cpeb4 | 10:15987920-16046033 | 1.26E-06 | 3.88 | 9.60E-05 |
| ENSRNOG00000048174.2 | Uqcrq | 10:38779131-38782419 | 1.27E-06 | 2.22 | 9.62E-05 |
| ENSRNOG00000013707.7 | Spata13 | 15:40937707-41066769 | 1.38E-06 | 3.40 | 0.0001 |
| ENSRNOG00000043201.2 | Coq8a | 13:98451636-98480419 | 1.4E-06 | -2.11 | 0.0001 |
| ENSRNOG00000000158.5 | Cdo1 | 18:40701983-40716686 | 1.42E-06 | -2.79 | 0.0001 |
| ENSRNOG00000018944.5 | Pank1 | 1:253119111-253185533 | 1.51E-06 | -2.82 | 0.0001 |
| ENSRNOG00000050741.2 | Rbm4 | 1:220039733-220048966 | 1.68E-06 | 2.18 | 0.0001 |
| ENSRNOG00000053790.1 | AABR07036007.1 | 12:30441054-30444565 | 1.72E-06 | 3.05 | 0.0001 |
| ENSRNOG00000006698.6 | Rab33a | X:135348435-135360203 | 1.86E-06 | -2.40 | 0.0001 |
| ENSRNOG00000062155.1 | AC134224.2 | 1:221151826-221153278 | 1.89E-06 | 2.71 | 0.0001 |
| ENSRNOG00000002218.5 | Stbd1 | 14:16976355-16979760 | 1.92E-06 | 2.52 | 0.0001 |
| ENSRNOG00000008748.4 | Pex2 | 2:98252924-98269184 | 2.01E-06 | -2.50 | 0.0001 |
| ENSRNOG00000014021.6 | Matn4 | 3:160838631-160853650 | 2E-06 | 4.76 | 0.0001 |
| ENSRNOG00000025484.3 | Tmem177 | 13:35929818-35933243 | 2E-06 | -5.42 | 0.0001 |
| ENSRNOG00000003334.5 | Klhl21 | 5:169181417-169190073 | 2.07E-06 | 2.36 | 0.0001 |
| ENSRNOG00000016552.6 | Hmgcs1 | 2:52431600-52445055 | 2.07E-06 | -3.39 | 0.0001 |
| ENSRNOG00000020703.7 | Sipa1l3 | 1:87260834-87468288 | 2.2E-06 | 3.32 | 0.0002 |
| ENSRNOG00000014310.7 | Tmtc4 | 15:109339563-109394927 | 2.38E-06 | -2.55 | 0.0002 |
| ENSRNOG00000018257.5 | Hpx | 1:170423482-170431073 | 2.54E-06 | 2.29 | 0.0002 |
| ENSRNOG00000005275.6 | Shmt1 | 10:47031049-47059216 | 2.56E-06 | -2.43 | 0.0002 |
| ENSRNOG00000015415.6 | Rhoq | 6:10533150-10568581 | 2.68E-06 | 3.09 | 0.0002 |
| ENSRNOG00000045683.2 | LOC102553715 | 10:49000972-49003931 | 2.73E-06 | 3.56 | 0.0002 |
| ENSRNOG00000033433.4 | Csrnp1 | 8:128659867-128672284 | 2.81E-06 | 2.83 | 0.0002 |
| ENSRNOG00000012561.8 | Arhgef10 | 16:79725642-79793619 | 2.84E-06 | 3.15 | 0.0002 |
| ENSRNOG00000011778.6 | Blvra | 3:119561289-119577806 | 2.91E-06 | 2.27 | 0.0002 |
| ENSRNOG00000016573.6 | Dgat2 | 1:164113458-164143818 | 2.98E-06 | -3.23 | 0.0002 |
| ENSRNOG00000004255.4 | Usf1 | 13:89797799-89805558 | 3.02E-06 | -2.05 | 0.0002 |
| ENSRNOG00000046112.2 | Dedd2 | 1:82072543-82088275 | 3.07E-06 | 3.81 | 0.0002 |
| ENSRNOG00000053210.1 | Zc3h11a | 13:50196041-50234862 | 3.15E-06 | 2.06 | 0.0002 |
| ENSRNOG00000024533.3 | Eogt | 4:129477806-129515435 | 3.34E-06 | -3.20 | 0.0002 |
| ENSRNOG00000004535.6 | Kcng3 | 6:6794807-6842758 | 3.35E-06 | 9.82 | 0.0002 |
| ENSRNOG00000022054.2 | Paqr7 | 5:152708774-152720229 | 3.38E-06 | -2.70 | 0.0002 |
| ENSRNOG00000021916.5 | Slc16a12 | 1:252976643-253000760 | 3.89E-06 | -3.59 | 0.0003 |
| ENSRNOG00000029810.3 | LOC100911766 | 1:214454089-214473741 | 3.9E-06 | -2.29 | 0.0003 |
| ENSRNOG00000027008.6 | Igtp | 10:43613930-43653390 | 3.98E-06 | -4.07 | 0.0003 |
| ENSRNOG00000022702.5 | Fbxo22 | 8:59769993-59786065 | 3.99E-06 | -2.43 | 0.0003 |
| ENSRNOG00000003463.8 | Srebf1 | 10:46570995-46593009 | 4.02E-06 | -2.32 | 0.0003 |
| ENSRNOG00000004049.7 | Baiap2 | 10:109107388-109187115 | 4.19E-06 | 2.98 | 0.0003 |
| ENSRNOG00000013928.6 | Dsp | 17:27286795-27334379 | 4.38E-06 | 2.06 | 0.0003 |
| ENSRNOG00000010121.5 | Lef1 | 2:236233238-236345056 | 4.4E-06 | -2.82 | 0.0003 |
| ENSRNOG00000045568.3 | Rbm14 | 1:220048872-220067124 | 4.53E-06 | 2.60 | 0.0003 |
| ENSRNOG00000050560.1 | Polr2i | 1:91041213-91042230 | 4.62E-06 | -2.98 | 0.0003 |
| ENSRNOG00000020392.7 | Stxbp3 | 2:211595658-211638788 | 4.73E-06 | -3.00 | 0.0003 |
| ENSRNOG00000057284.1 | Cenpb | 3:123721485-123723524 | 4.92E-06 | -2.26 | 0.0003 |
| ENSRNOG00000015285.6 | Lrp4 | 3:80362857-80416679 | 4.95E-06 | 5.17 | 0.0003 |
| ENSRNOG00000021056.6 | Kcnj14 | 1:101805530-101809544 | 5.66E-06 | 3.93 | 0.0004 |
| ENSRNOG00000026128.4 | Cpne8 | 7:131623180-131798236 | 5.66E-06 | 3.83 | 0.0004 |
| ENSRNOG00000004373.6 | Ddx39a | 19:24846937-24854953 | 5.68E-06 | 2.78 | 0.0004 |
| ENSRNOG00000002196.7 | Ociad2 | 14:37435653-37444775 | 5.9E-06 | 2.28 | 0.0004 |
| ENSRNOG00000009910.6 | Swap70 | 1:174862697-174924078 | 6.08E-06 | 2.52 | 0.0004 |
| ENSRNOG00000016924.8 | Acly | 10:88392247-88442845 | 6.19E-06 | -2.02 | 0.0004 |
| ENSRNOG00000051831.1 | 5_8S_rRNA | 1:11906440-11906593 | 6.89E-06 | -11.57 | 0.0004 |
| ENSRNOG00000050792.2 | Tnfaip6 | 3:37545237-37564699 | 7.17E-06 | 3.22 | 0.0005 |
| ENSRNOG00000000796.8 | Ranbp2 | 20:28027053-28076664 | 7.23E-06 | 2.82 | 0.0005 |
| ENSRNOG00000017037.7 | Otud3 | 5:157346285-157368472 | 7.29E-06 | 3.58 | 0.0005 |
| ENSRNOG00000014648.6 | Efnb2 | 16:86631063-86675049 | 7.35E-06 | 2.93 | 0.0005 |
| ENSRNOG00000037658.3 | Gprasp2 | X:106360392-106363300 | 7.46E-06 | 3.21 | 0.0005 |
| ENSRNOG00000028895.4 | Rtp4 | 11:80638944-80650802 | 7.96E-06 | -4.68 | 0.0005 |
| ENSRNOG00000002511.8 | Flt4 | 10:35078725-35121599 | 8.08E-06 | 2.30 | 0.0005 |
| ENSRNOG00000006727.5 | Dtd2 | 6:72780542-72786830 | 8.4E-06 | -3.83 | 0.0005 |
| ENSRNOG00000030639.5 | Usp13 | 2:119197238-119317500 | 8.4E-06 | 3.58 | 0.0005 |
| ENSRNOG00000030721.6 | Fbrsl1 | 12:52356831-52377475 | 8.39E-06 | 2.35 | 0.0005 |
| ENSRNOG00000019422.4 | Egr1 | 18:27657627-27661429 | 8.55E-06 | 4.09 | 0.0005 |
| ENSRNOG00000046912.2 | Nr1d2 | 15:8730870-8757165 | 8.57E-06 | -2.58 | 0.0005 |
| ENSRNOG00000002254.6 | Tmem33 | 14:42535700-42560174 | 9.32E-06 | 2.57 | 0.0006 |
| ENSRNOG00000043069.2 | Sec22a | 11:68633159-68686548 | 9.59E-06 | -2.20 | 0.0006 |
| ENSRNOG00000000648.6 | Jmjd1c | 20:22751742-22882672 | 1E-05 | 2.89 | 0.0006 |
| ENSRNOG00000005359.7 | Csrnp3 | 3:51883558-52120290 | 1.06E-05 | 8.10 | 0.0006 |
| ENSRNOG00000058908.1 | Hmgb1 | 16:37500016-37502237 | 1.11E-05 | -2.11 | 0.0007 |
| ENSRNOG00000048848.2 | Ccdc9 | 1:78213222-78224319 | 1.13E-05 | -2.00 | 0.0007 |
| ENSRNOG00000027032.5 | Rhbdd3 | 14:85351357-85357023 | 1.2E-05 | 2.26 | 0.0007 |
| ENSRNOG00000004466.4 | Fam210b | 3:170354140-170362513 | 1.21E-05 | -2.05 | 0.0007 |
| ENSRNOG00000043189.3 | Trub2 | 3:8338483-8348746 | 1.23E-05 | -2.52 | 0.0007 |
| ENSRNOG00000018477.6 | Otud4 | 19:31899086-31942180 | 1.23E-05 | 2.92 | 0.0007 |
| ENSRNOG00000048248.2 | LOC100910446 | 1:23543425-23556241 | 1.34E-05 | 13.57 | 0.0008 |
| ENSRNOG00000029614.5 | Robo1 | 11:9642364-10143317 | 1.35E-05 | 2.35 | 0.0008 |
| ENSRNOG00000009570.7 | Pdxp | 7:120140459-120145908 | 1.39E-05 | 3.00 | 0.0008 |
| ENSRNOG00000014254.3 | Cpt1a | 1:218569509-218629678 | 1.45E-05 | -2.70 | 0.0009 |
| ENSRNOG00000005291.6 | Slc38a1 | 7:137975548-138039984 | 1.48E-05 | 2.68 | 0.0009 |
| ENSRNOG00000006539.7 | Pex16 | 3:81283136-81292573 | 1.5E-05 | -2.19 | 0.0009 |
| ENSRNOG00000005849.7 | Aco1 | 5:56425023-56481218 | 1.55E-05 | -2.40 | 0.0009 |
| ENSRNOG00000062127.1 | AC134224.1 | 1:221154560-221156910 | 1.55E-05 | 2.28 | 0.0009 |
| ENSRNOG00000051372.1 | Mycn | 6:38222554-38228379 | 1.56E-05 | 4.09 | 0.0009 |
| ENSRNOG00000047005.2 | Kcnk5 | 15:4554602-4597000 | 1.7E-05 | 2.61 | 0.0010 |
| ENSRNOG00000021062.5 | Fxyd5 | 1:89464859-89474252 | 1.9E-05 | 2.06 | 0.0011 |
| ENSRNOG00000006178.8 | Dync1h1 | 6:134958853-135085769 | 1.9E-05 | 2.05 | 0.0011 |
| ENSRNOG00000025076.7 | Ecpas | 5:75931292-76039760 | 1.98E-05 | 2.02 | 0.0011 |
| ENSRNOG00000016791.8 | Chka | 1:219077770-219126220 | 2E-05 | 2.13 | 0.0011 |
| ENSRNOG00000045636.2 | Fasn | 10:109987734-110005901 | 2.06E-05 | -3.03 | 0.0012 |
| ENSRNOG00000056427.1 | AABR07007717.1 | 2:25564883-25565757 | 2.29E-05 | 3.43 | 0.0013 |
| ENSRNOG00000010633.3 | Acsl1 | 16:48937455-49003246 | 2.34E-05 | -2.11 | 0.0013 |
| ENSRNOG00000015796.7 | Msh2 | 6:11215962-11274932 | 2.4E-05 | -3.01 | 0.0014 |
| ENSRNOG00000014426.7 | Lox | 18:47500329-47577819 | 2.51E-05 | 2.14 | 0.0014 |
| ENSRNOG00000015242.5 | Fbxl8 | 19:37221131-37226065 | 2.57E-05 | 4.29 | 0.0014 |
| ENSRNOG00000010658.6 | LOC103691744 | 2:264266983-264293046 | 2.58E-05 | 3.38 | 0.0014 |
| ENSRNOG00000018317.6 | Aak1 | 4:118655727-118795774 | 2.58E-05 | 4.10 | 0.0014 |
| ENSRNOG00000015644.5 | Ugcg | 5:76386835-76419560 | 2.6E-05 | -2.28 | 0.0015 |
| ENSRNOG00000001113.6 | Mmd2 | 12:14021726-14078134 | 2.75E-05 | -2.09 | 0.0015 |
| ENSRNOG00000019542.5 | MGC108823 | 18:55576238-55584957 | 2.9E-05 | -3.52 | 0.0016 |
| ENSRNOG00000004660.5 | Fzd6 | 7:77899321-77931029 | 2.94E-05 | -3.24 | 0.0016 |
| ENSRNOG00000053040.1 | AC094643.2 | 7:11181385-11182279 | 3E-05 | -2.69 | 0.0016 |
| ENSRNOG00000062130.1 | AC108578.1 | 8:59539059-59539602 | 2.99E-05 | 3.59 | 0.0016 |
| ENSRNOG00000004891.4 | Arl14ep | 3:97983685-97993684 | 3.07E-05 | -3.66 | 0.0017 |
| ENSRNOG00000014187.6 | Igf1r | 1:128924965-129206516 | 3.07E-05 | 3.16 | 0.0017 |
| ENSRNOG00000025509.7 | Apbb2 | 14:43512900-43523080 | 3.07E-05 | 3.28 | 0.0017 |
| ENSRNOG00000005371.8 | Klhl29 | 6:29178857-29483460 | 3.14E-05 | 2.75 | 0.0017 |
| ENSRNOG00000047657.2 | C4a | 20:2651598-2678141 | 3.14E-05 | 2.46 | 0.0017 |
| ENSRNOG00000012386.6 | Zbtb38 | 8:104589620-104593625 | 3.2E-05 | 2.66 | 0.0017 |
| ENSRNOG00000010947.8 | Mmp14 | 15:33074440-33083655 | 3.23E-05 | -2.41 | 0.0017 |
| ENSRNOG00000005802.7 | Usp24 | 5:125896724-126030401 | 3.25E-05 | 2.54 | 0.0018 |
| ENSRNOG00000012847.5 | Scgb1c1 | 1:213595239-213596459 | 3.37E-05 | 2.92 | 0.0018 |
| ENSRNOG00000003927.7 | Cd55 | 13:47126740-47154292 | 3.43E-05 | 5.43 | 0.0018 |
| ENSRNOG00000018243.7 | Ubfd1 | 1:192025356-192037025 | 3.59E-05 | 2.92 | 0.0019 |
| ENSRNOG00000020254.7 | Per2 | 9:98555168-98597359 | 3.94E-05 | -3.00 | 0.0021 |
| ENSRNOG00000042126.1 | Lsm5 | 4:87016002-87019219 | 3.94E-05 | -2.13 | 0.0021 |
| ENSRNOG00000006048.6 | Ezh2 | 4:77284403-77347011 | 4.03E-05 | 5.08 | 0.0021 |
| ENSRNOG00000060735.1 | AABR07053136.1 | 3:93648342-93648657 | 4.11E-05 | 3.77 | 0.0022 |
| ENSRNOG00000018413.7 | Per3 | 5:168086997-168123395 | 4.17E-05 | -4.99 | 0.0022 |
| ENSRNOG00000017833.7 | Actn2 | 17:66304500-66397653 | 4.2E-05 | 3.04 | 0.0022 |
| ENSRNOG00000015179.6 | Tradd | 19:37214470-37216572 | 4.28E-05 | -2.92 | 0.0022 |
| ENSRNOG00000022256.6 | Cxcl10 | 14:17210732-17212930 | 4.64E-05 | -4.27 | 0.0024 |
| ENSRNOG00000013934.6 | Dennd2b | 1:174137119-174289647 | 4.67E-05 | -3.32 | 0.0024 |
| ENSRNOG00000022582.2 | Ccnq | 10:66019519-66020682 | 4.71E-05 | -2.94 | 0.0024 |
| ENSRNOG00000012641.5 | Sfr1 | 1:267483144-267509350 | 4.73E-05 | -2.15 | 0.0025 |
| ENSRNOG00000033215.6 | RT1-Db1 | 20:4087617-4097190 | 4.95E-05 | 2.23 | 0.0026 |
| ENSRNOG00000019390.5 | Klhl40 | 8:130416354-130421871 | 4.96E-05 | -8.67 | 0.0026 |
| ENSRNOG00000057556.1 | Pdzrn3 | 4:133717138-133951264 | 5E-05 | -2.91 | 0.0026 |
| ENSRNOG00000020379.7 | Cog8 | 19:39246624-39257451 | 5.19E-05 | -2.67 | 0.0026 |
| ENSRNOG00000018371.4 | Tubb6 | 18:63130541-63140181 | 5.29E-05 | 3.45 | 0.0027 |
| ENSRNOG00000009035.4 | Malsu1 | 4:78735278-78744102 | 5.36E-05 | -2.31 | 0.0027 |
| ENSRNOG00000009846.7 | Frg1 | 16:53957857-53978865 | 5.64E-05 | -2.43 | 0.0028 |
| ENSRNOG00000043098.1 | Mt2A | 19:11307966-11308740 | 5.79E-05 | -3.01 | 0.0029 |
| ENSRNOG00000019068.4 | Ctrb1 | 19:43906291-43911057 | 6.13E-05 | 21.90 | 0.0030 |
| ENSRNOG00000049866.2 | Plcxd1 | 12:52641221-52676608 | 6.13E-05 | 16.23 | 0.0030 |
| ENSRNOG00000000443.7 | LOC103689965 | 20:4302346-4508214 | 6.23E-05 | 2.20 | 0.0031 |
| ENSRNOG00000061099.1 | AABR07033324.1 | 11:17336443-17340373 | 6.26E-05 | 3.41 | 0.0031 |
| ENSRNOG00000010381.7 | Mknk1 | 5:134691880-134730893 | 6.45E-05 | -2.10 | 0.0032 |
| ENSRNOG00000024578.7 | Ttyh2 | 10:103206013-103248862 | 6.66E-05 | -2.22 | 0.0033 |
| ENSRNOG00000055567.1 | Fmnl2 | 3:38559913-38675878 | 6.77E-05 | 2.31 | 0.0033 |
| ENSRNOG00000057125.1 | Ddr1 | 20:3552928-3575780 | 6.77E-05 | -3.09 | 0.0033 |
| ENSRNOG00000009311.6 | Fstl3 | 7:12804918-12810570 | 6.8E-05 | 2.66 | 0.0033 |
| ENSRNOG00000007582.6 | Zswim4 | 19:25320682-25345790 | 6.85E-05 | 2.66 | 0.0033 |
| ENSRNOG00000018927.2 | Sprn | 1:212622093-212622537 | 6.88E-05 | -2.88 | 0.0033 |
| ENSRNOG00000008224.6 | Jdp2 | 6:109466059-109505161 | 7.46E-05 | 3.14 | 0.0036 |
| ENSRNOG00000018242.7 | Camkk1 | 10:59585071-59608165 | 7.46E-05 | -4.97 | 0.0036 |
| ENSRNOG00000017382.4 | Snx33 | 8:61584655-61595032 | 7.49E-05 | -2.55 | 0.0036 |
| ENSRNOG00000031939.5 | AABR07066416.1 | 9:8629215-8632017 | 7.54E-05 | -3.16 | 0.0036 |
| ENSRNOG00000009881.7 | Fam161a | 14:107785028-107798115 | 7.67E-05 | 8.83 | 0.0037 |
| ENSRNOG00000027654.4 | Appbp2 | 10:72503346-72545141 | 7.75E-05 | -2.34 | 0.0037 |
| ENSRNOG00000003075.6 | Tmem39a | 11:64723185-64752544 | 7.92E-05 | 2.71 | 0.0038 |
| ENSRNOG00000043451.4 | Spp1 | 14:6673685-6679901 | 8.16E-05 | 4.81 | 0.0039 |
| ENSRNOG00000019236.8 | Rnf220 | 5:136129424-136166786 | 8.4E-05 | -2.03 | 0.0040 |
| ENSRNOG00000017601.7 | Srd5a1 | 1:36320460-36354756 | 8.53E-05 | -2.01 | 0.0040 |
| ENSRNOG00000005623.7 | Ankmy2 | 6:55648020-55689218 | 8.83E-05 | -2.83 | 0.0042 |
| ENSRNOG00000007829.4 | Lactb2 | 5:4982347-5005109 | 8.87E-05 | -2.14 | 0.0042 |
| ENSRNOG00000019361.5 | Inpp5j | 14:83730861-83741969 | 8.86E-05 | -8.01 | 0.0042 |
| ENSRNOG00000016152.4 | Dek | 17:17965880-17987834 | 9.31E-05 | -2.35 | 0.0044 |
| ENSRNOG00000045840.1 | Hist2h4a | 4:170763604-170763916 | 9.39E-05 | 2.24 | 0.0044 |
| ENSRNOG00000002722.5 | Sec14l1 | 10:106065711-106112857 | 9.81E-05 | -2.12 | 0.0046 |
| ENSRNOG00000001773.7 | Senp2 | 11:82630435-82664630 | 9.98E-05 | 2.49 | 0.0046 |
| ENSRNOG00000027747.7 | Ninl | 3:146719910-146764015 | 9.98E-05 | 3.48 | 0.0046 |
| ENSRNOG00000019080.4 | Hsd3b7 | 1:199248469-199251740 | 0.0001 | -2.50 | 0.0047 |
| ENSRNOG00000019215.6 | LOC100911730 | 1:214009783-214013765 | 0.0001 | 2.54 | 0.0047 |
| ENSRNOG00000016156.6 | Nptxr | 7:121011679-121029754 | 0.0001 | 3.50 | 0.0047 |
| ENSRNOG00000005749.5 | Foxred2 | 7:119170180-119185238 | 0.0001 | 5.05 | 0.0048 |
| ENSRNOG00000017496.4 | Cnp | 10:88490797-88497356 | 0.0001 | -2.34 | 0.0048 |
| ENSRNOG00000058457.1 | Chtopl1 | 4:163118451-163119391 | 0.0001 | 2.53 | 0.0048 |
| ENSRNOG00000012565.4 | Cln8 | 16:79828323-79838212 | 0.0001 | 2.81 | 0.0051 |
| ENSRNOG00000006670.3 | Rai2 | X:34731890-34794589 | 0.0001 | -4.28 | 0.0051 |
| ENSRNOG00000001880.6 | Dgcr6 | 11:87076380-87081950 | 0.0001 | 2.06 | 0.0052 |
| ENSRNOG00000014903.7 | Zfyve27 | 1:261415190-261438539 | 0.0001 | 2.39 | 0.0052 |
| ENSRNOG00000013111.7 | Mettl3 | 15:28710299-28721127 | 0.0001 | -2.08 | 0.0052 |
| ENSRNOG00000008533.4 | Ago2 | 7:114339433-114380613 | 0.0001 | 4.02 | 0.0053 |
| ENSRNOG00000010017.6 | Wee1 | 1:174767959-174786361 | 0.0001 | -4.27 | 0.0053 |
| ENSRNOG00000003897.6 | Col1a1 | 10:82745800-82762789 | 0.0001 | 2.23 | 0.0053 |
| ENSRNOG00000010099.7 | Asb8 | 7:139724345-139734568 | 0.0001 | -2.46 | 0.0054 |
| ENSRNOG00000001465.7 | Ids | 8:69449800-69466618 | 0.0001 | 3.23 | 0.0055 |
| ENSRNOG00000011461.4 | Ap3m1 | 15:3436360-3454279 | 0.0001 | -2.34 | 0.0056 |
| ENSRNOG00000002129.7 | Lrrc8b | 14:5418740-5428757 | 0.0001 | 2.85 | 0.0057 |
| ENSRNOG00000009983.6 | Lrrc42 | 5:126835154-126856292 | 0.0001 | -2.61 | 0.0061 |
| ENSRNOG00000047783.2 | Tmem200a | 1:20331975-20412381 | 0.0001 | -2.04 | 0.0063 |
| ENSRNOG00000021817.5 | Irak2 | 4:145594574-145651053 | 0.0001 | 2.29 | 0.0063 |
| ENSRNOG00000019082.5 | LOC103690016 | 1:67368383-67390141 | 0.0001 | -3.97 | 0.0063 |
| ENSRNOG00000008837.6 | Ass1 | 3:10327413-10375826 | 0.0001 | 4.05 | 0.0065 |
| ENSRNOG00000052357.1 | Fosl2 | 6:25598935-25616995 | 0.0002 | 2.18 | 0.0066 |
| ENSRNOG00000051158.3 | Cfb | 20:4536211-4542073 | 0.0002 | 8.06 | 0.0066 |
| ENSRNOG00000008607.8 | U2surp | 8:103246557-103298927 | 0.0002 | 2.00 | 0.0067 |
| ENSRNOG00000006700.7 | Wdyhv1 | 7:97957002-97994586 | 0.0002 | 2.48 | 0.0068 |
| ENSRNOG00000010642.5 | Lysmd2 | 8:82560760-82577019 | 0.0002 | -2.68 | 0.0068 |
| ENSRNOG00000031391.4 | Ceacam16 | 1:80773818-80783898 | 0.0002 | 2.03 | 0.0068 |
| ENSRNOG00000050193.2 | LOC100912262 | 12:29012694-29080011 | 0.0002 | 5.33 | 0.0068 |
| ENSRNOG00000050343.3 | Jmy | 2:23042188-23097610 | 0.0002 | 5.88 | 0.0068 |
| ENSRNOG00000058589.1 | AABR07046778.1 | 5:6373582-6373849 | 0.0002 | -3.49 | 0.0068 |
| ENSRNOG00000032398.4 | RGD1562136 | 9:111327401-111347430 | 0.0002 | -2.25 | 0.0069 |
| ENSRNOG00000025608.4 | Lrat | 2:181896304-181905300 | 0.0002 | 2.25 | 0.0071 |
| ENSRNOG00000018246.6 | Tdp2 | 17:42229641-42241066 | 0.0002 | -2.45 | 0.0073 |
| ENSRNOG00000010161.8 | Myo10 | 2:77868411-78071309 | 0.0002 | 2.26 | 0.0074 |
| ENSRNOG00000060568.1 | Ptpn21 | 6:122656499-122721496 | 0.0002 | -3.27 | 0.0075 |
| ENSRNOG00000006206.7 | Tmem106b | 4:39517547-39535169 | 0.0002 | -2.16 | 0.0077 |
| ENSRNOG00000013751.6 | Plpbp | 16:69163576-69176036 | 0.0002 | -2.38 | 0.0077 |
| ENSRNOG00000001469.8 | Eln | 12:24978482-25021863 | 0.0002 | 3.61 | 0.0078 |
| ENSRNOG00000036802.4 | Snhg11 | 3:154863071-154869460 | 0.0002 | 2.01 | 0.0079 |
| ENSRNOG00000046560.1 | AC109096.1 | 1:220836091-220836504 | 0.0002 | -2.62 | 0.0079 |
| ENSRNOG00000015124.6 | Gpam | 1:275846818-275906686 | 0.0002 | -4.30 | 0.0081 |
| ENSRNOG00000027357.4 | Sox17 | 5:14890407-14895907 | 0.0002 | 3.71 | 0.0081 |
| ENSRNOG00000047503.2 | Apoc3 | 8:50529317-50531498 | 0.0002 | 3.85 | 0.0081 |
| ENSRNOG00000001851.7 | Far2 | 4:182483193-182565436 | 0.0002 | 2.82 | 0.0082 |
| ENSRNOG00000033776.3 | LOC100359668 | 6:76652205-76652757 | 0.0002 | 4.30 | 0.0082 |
| ENSRNOG00000008309.5 | Rpa3 | 4:34279319-34282351 | 0.0002 | -2.44 | 0.0089 |
| ENSRNOG00000014367.6 | Ephb6 | 4:70903252-70918514 | 0.0002 | -2.51 | 0.0090 |
| ENSRNOG00000010888.7 | Ankrd33b | 2:84361060-84437084 | 0.0002 | 6.10 | 0.0092 |
| ENSRNOG00000045918.3 | Jarid2 | 17:20184244-20364714 | 0.0002 | 2.74 | 0.0093 |
| ENSRNOG00000018408.7 | RGD1307554 | 1:84361735-84386751 | 0.0002 | 2.62 | 0.0093 |
| ENSRNOG00000031315.2 | Rpl36al | 10:57251252-57251573 | 0.0002 | -9.06 | 0.0094 |
| ENSRNOG00000003745.3 | Atf3 | 13:109817727-109849632 | 0.0003 | 6.12 | 0.0102 |
| ENSRNOG00000013269.5 | Tnfsf10 | 2:113007548-113026899 | 0.0003 | -2.16 | 0.0109 |
| ENSRNOG00000025394.6 | Tanc1 | 3:45683992-45917745 | 0.0003 | 2.62 | 0.0110 |
| ENSRNOG00000047118.2 | LOC683897 | 20:48275761-48294948 | 0.0003 | -2.88 | 0.0110 |
| ENSRNOG00000047924.1 | Zbtb7c | 18:72005580-72462292 | 0.0003 | 2.70 | 0.0110 |
| ENSRNOG00000012756.6 | Slc25a43 | X:123350345-123384846 | 0.0003 | -3.84 | 0.0110 |
| ENSRNOG00000011238.6 | Tiparp | 2:157316265-157341469 | 0.0003 | 3.48 | 0.0111 |
| ENSRNOG00000029330.4 | Ca5b | X:32232141-32292072 | 0.0003 | -3.64 | 0.0111 |
| ENSRNOG00000050786.2 | AC103024.2 | 10:34316836-34317161 | 0.0003 | -5.70 | 0.0111 |
| ENSRNOG00000013256.5 | Ric8a | 1:213606598-213612904 | 0.0003 | -2.01 | 0.0114 |
| ENSRNOG00000002040.8 | Bmp2k | 14:14129724-14192048 | 0.0003 | 3.56 | 0.0117 |
| ENSRNOG00000021726.5 | Tlr3 | 16:50016856-50031214 | 0.0003 | -3.92 | 0.0117 |
| ENSRNOG00000052421.1 | AABR07012475.2 | 2:196587205-196591324 | 0.0003 | -4.13 | 0.0117 |
| ENSRNOG00000049128.2 | Nktr | 8:130366774-130393178 | 0.0003 | 2.19 | 0.0119 |
| ENSRNOG00000054080.1 | Cgnl1 | 8:78126674-78233430 | 0.0003 | -2.13 | 0.0120 |
| ENSRNOG00000019657.3 | Mk1 | 16:20531719-20534209 | 0.0003 | -3.19 | 0.0122 |
| ENSRNOG00000002342.6 | Aldh3a2 | 10:47525492-47546345 | 0.0003 | -2.35 | 0.0122 |
| ENSRNOG00000008892.6 | Parp2 | 15:27739250-27749648 | 0.0003 | -2.93 | 0.0122 |
| ENSRNOG00000053201.1 | Gpcpd1 | 3:125169134-125213607 | 0.0003 | -2.82 | 0.0122 |
| ENSRNOG00000061425.1 | Cox14 | 7:141380321-141380495 | 0.0003 | 2.27 | 0.0123 |
| ENSRNOG00000026880.6 | Usp38 | 19:30668601-30699859 | 0.0003 | 2.54 | 0.0123 |
| ENSRNOG00000022082.6 | LOC103690006 | 4:117800627-117814658 | 0.0003 | 2.74 | 0.0124 |
| ENSRNOG00000012343.5 | Pdp2 | 19:597426-601469 | 0.0003 | 2.02 | 0.0125 |
| ENSRNOG00000004217.6 | Stk10 | 10:17421074-17519706 | 0.0003 | 3.97 | 0.0126 |
| ENSRNOG00000000662.7 | Srrd | 12:50315932-50321144 | 0.0003 | 2.28 | 0.0131 |
| ENSRNOG00000020881.7 | Frmd8 | 1:221213311-221233962 | 0.0004 | -2.35 | 0.0135 |
| ENSRNOG00000028185.5 | Tstd3 | 5:35892736-35902262 | 0.0004 | -2.03 | 0.0135 |
| ENSRNOG00000030410.3 | Zfp958 | 12:5485902-5490935 | 0.0004 | -2.63 | 0.0135 |
| ENSRNOG00000025372.5 | Glce | 8:67006591-67040005 | 0.0004 | -2.94 | 0.0137 |
| ENSRNOG00000027742.6 | Adamtsl2 | 3:5624505-5654910 | 0.0004 | 2.79 | 0.0137 |
| ENSRNOG00000050453.2 | AABR07002848.1 | 1:86873252-86888958 | 0.0004 | 2.76 | 0.0137 |
| ENSRNOG00000010706.4 | Ccdc117 | 14:85763396-85772762 | 0.0004 | 2.36 | 0.0140 |
| ENSRNOG00000017728.7 | Edrf1 | 1:205706467-205743653 | 0.0004 | 2.67 | 0.0141 |
| ENSRNOG00000016167.5 | Spata2L | 19:56032609-56037077 | 0.0004 | 3.24 | 0.0146 |
| ENSRNOG00000012772.5 | Nqo1 | 19:38422163-38437180 | 0.0004 | 2.18 | 0.0148 |
| ENSRNOG00000003784.7 | Rilp | 10:63659084-63662050 | 0.0004 | -2.55 | 0.0148 |
| ENSRNOG00000020110.4 | B4gat1 | 1:220416017-220418240 | 0.0004 | -2.73 | 0.0148 |
| ENSRNOG00000025558.8 | Palm2 | 5:74649764-74788497 | 0.0004 | 7.12 | 0.0150 |
| ENSRNOG00000010643.7 | Kank2 | 8:22791994-22821397 | 0.0004 | -2.01 | 0.0150 |
| ENSRNOG00000022268.6 | Pnpla3 | 7:125034763-125055976 | 0.0004 | -4.95 | 0.0151 |
| ENSRNOG00000042556.1 | Bmyc | 3:2916661-2917421 | 0.0004 | -2.48 | 0.0157 |
| ENSRNOG00000001859.5 | Sdf2l1 | 11:88122270-88124513 | 0.0004 | 2.45 | 0.0160 |
| ENSRNOG00000007329.8 | Frmd6 | 6:93281407-93358840 | 0.0004 | -2.33 | 0.0160 |
| ENSRNOG00000015455.6 | Spr | 4:116912350-116916236 | 0.0004 | -2.51 | 0.0164 |
| ENSRNOG00000017290.8 | Zfp335 | 3:161357277-161376119 | 0.0005 | 2.32 | 0.0168 |
| ENSRNOG00000018788.6 | Btbd2 | 7:11930134-11942799 | 0.0005 | -2.09 | 0.0171 |
| ENSRNOG00000013279.7 | LOC681458 | 1:263968133-264004008 | 0.0005 | 3.30 | 0.0172 |
| ENSRNOG00000016013.7 | Gprc5b | 1:188688742-188713280 | 0.0005 | 3.30 | 0.0172 |
| ENSRNOG00000014613.7 | Ddah1 | 2:251634430-251766014 | 0.0005 | 2.03 | 0.0176 |
| ENSRNOG00000060235.1 | AABR07044837.2 | 20:23073500-23083780 | 0.0005 | 17.87 | 0.0176 |
| ENSRNOG00000016998.4 | Atxn1 | 17:19249951-19533814 | 0.0005 | 4.92 | 0.0177 |
| ENSRNOG00000001052.7 | Slc25a30 | 15:57813355-57834173 | 0.0005 | 2.91 | 0.0179 |
| ENSRNOG00000014142.6 | Ogfrl1 | 9:29634178-29647903 | 0.0005 | 2.38 | 0.0183 |
| ENSRNOG00000053230.1 | Fopnl | 10:730246-751061 | 0.0005 | -2.70 | 0.0183 |
| ENSRNOG00000001153.3 | Pla2g1b | 12:46879342-46889082 | 0.0005 | 4.60 | 0.0190 |
| ENSRNOG00000026133.5 | Ly6d | 7:115993300-115994783 | 0.0005 | 4.31 | 0.0190 |
| ENSRNOG00000049215.1 | Cbx2 | 10:108132104-108140935 | 0.0005 | 5.02 | 0.0190 |
| ENSRNOG00000019441.5 | Hsd3b2 | 2:202341421-202350929 | 0.0005 | 3.13 | 0.0192 |
| ENSRNOG00000052134.1 | Pigy | 8:23167973-23170209 | 0.0005 | -2.83 | 0.0192 |
| ENSRNOG00000002373.8 | Akap1 | 10:76151037-76166276 | 0.0006 | -2.51 | 0.0194 |
| ENSRNOG00000005515.4 | Rhbdl3 | 10:67677070-67731670 | 0.0006 | -2.67 | 0.0200 |
| ENSRNOG00000055524.1 | Trip10 | 9:9689474-9702306 | 0.0006 | 2.09 | 0.0202 |
| ENSRNOG00000002926.5 | Uap1 | 13:88463531-88497901 | 0.0006 | 2.31 | 0.0206 |
| ENSRNOG00000014674.7 | Exosc9 | 2:123264693-123294880 | 0.0006 | -2.47 | 0.0206 |
| ENSRNOG00000004135.6 | RGD1311745 | 8:132029620-132036472 | 0.0006 | -2.51 | 0.0208 |
| ENSRNOG00000051253.1 | LOC102553828 | 1:133713280-133717580 | 0.0006 | 7.95 | 0.0209 |
| ENSRNOG00000008292.6 | Hif1a | 6:96810906-96856052 | 0.0006 | -2.06 | 0.0211 |
| ENSRNOG00000019869.8 | Lrfn1 | 1:85291461-85300825 | 0.0007 | -2.46 | 0.0225 |
| ENSRNOG00000046132.1 | AABR07024439.1 | 16:43813-45929 | 0.0007 | -5.25 | 0.0231 |
| ENSRNOG00000006320.7 | Ptges | 3:9727407-9738752 | 0.0007 | 3.15 | 0.0233 |
| ENSRNOG00000010630.6 | Prcp | 1:157701088-157750169 | 0.0007 | 3.14 | 0.0233 |
| ENSRNOG00000020185.7 | Wdr6 | 8:117358786-117366096 | 0.0007 | -2.44 | 0.0233 |
| ENSRNOG00000023334.6 | Parp14 | 11:68105368-68137535 | 0.0007 | -2.13 | 0.0233 |
| ENSRNOG00000025587.4 | Plagl1 | 1:7252348-7259035 | 0.0007 | 3.02 | 0.0233 |
| ENSRNOG00000047581.3 | AABR07066700.1 | 9:12472371-12588253 | 0.0007 | -3.44 | 0.0234 |
| ENSRNOG00000000525.8 | Pi16 | 20:6923488-6932786 | 0.0007 | 2.35 | 0.0242 |
| ENSRNOG00000003526.8 | Sytl4 | X:104765293-104814145 | 0.0007 | -2.45 | 0.0242 |
| ENSRNOG00000016678.3 | Angptl2 | 3:12262821-12292663 | 0.0008 | -2.11 | 0.0253 |
| ENSRNOG00000002474.7 | Tom1l1 | 10:78173846-78219793 | 0.0008 | -3.90 | 0.0253 |
| ENSRNOG00000018467.7 | Mitd1 | 9:44472364-44560837 | 0.0008 | -3.48 | 0.0253 |
| ENSRNOG00000000275.8 | Fam13c | 20:19360229-19479325 | 0.0008 | 2.55 | 0.0258 |
| ENSRNOG00000027260.4 | Adprh | 11:64861767-64868108 | 0.0008 | -2.07 | 0.0265 |
| ENSRNOG00000012404.6 | Thrsp | 1:162381252-162385575 | 0.0008 | -3.62 | 0.0266 |
| ENSRNOG00000010078.5 | Spag1 | 7:74994604-75054293 | 0.0008 | -2.86 | 0.0268 |
| ENSRNOG00000022771.6 | Arhgap23 | 10:85384880-85444556 | 0.0008 | 2.55 | 0.0268 |
| ENSRNOG00000059492.1 | LOC100365363 | 1:60884760-60894948 | 0.0008 | -2.92 | 0.0268 |
| ENSRNOG00000060899.1 | AABR07040840.1 | X:111942101-111942749 | 0.0008 | -2.42 | 0.0268 |
| ENSRNOG00000020325.6 | Calhm2 | 1:266908368-266914093 | 0.0008 | 3.38 | 0.0272 |
| ENSRNOG00000029394.5 | Dusp8 | 1:215030428-215033460 | 0.0008 | 3.43 | 0.0272 |
| ENSRNOG00000028975.2 | Hist2h3c2 | 2:198380425-198380836 | 0.0009 | 6.21 | 0.0284 |
| ENSRNOG00000026295.4 | Rbpjl | 3:160852163-160864534 | 0.0009 | 2.53 | 0.0284 |
| ENSRNOG00000058039.1 | Acta2 | 1:252537614-252550394 | 0.0009 | 2.15 | 0.0287 |
| ENSRNOG00000009754.6 | Nampt | 6:52122084-52156472 | 0.0009 | 2.03 | 0.0288 |
| ENSRNOG00000005426.4 | Snupn | 8:61615649-61648390 | 0.0009 | -2.51 | 0.0290 |
| ENSRNOG00000020726.6 | Sipa1 | 1:221006339-221015929 | 0.0009 | 2.11 | 0.0290 |
| ENSRNOG00000016103.5 | Nkd2 | 1:32058043-32084877 | 0.0009 | 3.80 | 0.0296 |
| ENSRNOG00000054757.1 | Adcy6 | 7:140270691-140291620 | 0.0009 | 2.27 | 0.0298 |
| ENSRNOG00000008781.5 | Erg28 | 6:109683252-109692218 | 0.0009 | -2.81 | 0.0305 |
| ENSRNOG00000003119.4 | Gc | 14:20266890-20302581 | 0.0010 | 6.91 | 0.0315 |
| ENSRNOG00000003893.6 | Ccnjl | 10:29123272-29182568 | 0.0010 | 9.43 | 0.0315 |
| ENSRNOG00000001861.6 | Ydjc | 11:88090920-88093516 | 0.0010 | -2.36 | 0.0327 |
| ENSRNOG00000008572.6 | Ppcs | 5:138466292-138470096 | 0.0010 | -2.61 | 0.0328 |
| ENSRNOG00000018533.5 | Iffo1 | 4:157659146-157676331 | 0.0010 | -2.36 | 0.0328 |
| ENSRNOG00000016032.6 | Cnnm3 | 9:43093137-43108486 | 0.0011 | -2.49 | 0.0348 |
| ENSRNOG00000007972.5 | Rbbp9 | 3:138701585-138708332 | 0.0011 | -2.46 | 0.0355 |
| ENSRNOG00000032394.5 | Tymp | 7:130342482-130347587 | 0.0012 | -2.29 | 0.0369 |
| ENSRNOG00000016987.8 | Pstpip2 | 18:74174935-74292921 | 0.0012 | 2.29 | 0.0370 |
| ENSRNOG00000028254.4 | Tmem141 | 3:2843634-2845593 | 0.0012 | -2.72 | 0.0376 |
| ENSRNOG00000011111.6 | Cipc | 6:110968060-110990912 | 0.0012 | -2.52 | 0.0378 |
| ENSRNOG00000018295.7 | Ccar2 | 15:51818896-51834030 | 0.0013 | -2.03 | 0.0388 |
| ENSRNOG00000018567.7 | Slc20a1 | 3:121725858-121739173 | 0.0013 | 2.26 | 0.0389 |
| ENSRNOG00000008314.5 | Orc3 | 5:50020814-50075454 | 0.0013 | -3.12 | 0.0392 |
| ENSRNOG00000011015.6 | Hivep2 | 1:8310576-8333885 | 0.0013 | 2.67 | 0.0392 |
| ENSRNOG00000008619.6 | Agtrap | 5:164886812-164898420 | 0.0013 | -2.64 | 0.0392 |
| ENSRNOG00000033133.4 | Nae1 | 19:653509-680378 | 0.0013 | -2.64 | 0.0392 |
| ENSRNOG00000014946.7 | AABR07005588.1 | 1:189457582-189460844 | 0.0013 | -2.22 | 0.0393 |
| ENSRNOG00000020622.5 | Cilp2 | 16:21288875-21295869 | 0.0013 | -2.80 | 0.0394 |
| ENSRNOG00000012185.7 | Zfyve21 | 6:136380989-136400810 | 0.0013 | -2.18 | 0.0396 |
| ENSRNOG00000005059.5 | LOC100911917 | 3:4861752-4866833 | 0.0013 | -6.67 | 0.0401 |
| ENSRNOG00000028208.6 | Mief2 | 10:46972037-46974806 | 0.0013 | -3.60 | 0.0403 |
| ENSRNOG00000061031.1 | Fzd8 | 17:62262128-62265113 | 0.0013 | -3.76 | 0.0403 |
| ENSRNOG00000008758.5 | Tspan18 | 3:82097067-82236664 | 0.0013 | 2.49 | 0.0404 |
| ENSRNOG00000019382.6 | Zbtb47 | 8:130401469-130406991 | 0.0013 | -2.43 | 0.0406 |
| ENSRNOG00000061348.1 | Fam53b | 1:204714472-204817080 | 0.0014 | -2.55 | 0.0413 |
| ENSRNOG00000001374.6 | Rasal1 | 12:41416217-41448668 | 0.0014 | -2.34 | 0.0427 |
| ENSRNOG00000011398.4 | LOC103692719 | 6:136150784-136250752 | 0.0014 | 2.23 | 0.0432 |
| ENSRNOG00000055236.1 | Gemin4 | 10:64368808-64375816 | 0.0014 | -4.18 | 0.0432 |
| ENSRNOG00000015271.7 | Zfp418 | 1:69615433-69629913 | 0.0015 | 3.02 | 0.0437 |
| ENSRNOG00000058677.1 | Lsm8 | 4:45555076-45560943 | 0.0015 | -2.11 | 0.0437 |
| ENSRNOG00000028733.6 | Prkar1b | 12:17614631-17712373 | 0.0015 | 4.58 | 0.0446 |
| ENSRNOG00000013515.7 | Ptpru | 5:149922351-149996334 | 0.0015 | 2.50 | 0.0450 |
| ENSRNOG00000031138.3 | Irgm | 10:34213935-34221928 | 0.0015 | -2.72 | 0.0450 |
| ENSRNOG00000046382.2 | Gatd1 | 1:214389254-214394411 | 0.0015 | -3.76 | 0.0452 |
| ENSRNOG00000029698.5 | Pim3 | 7:129860113-129863441 | 0.0015 | 2.15 | 0.0458 |
| ENSRNOG00000004479.7 | Aurka | 3:170364180-170378210 | 0.0016 | 2.27 | 0.0476 |
| ENSRNOG00000000303.7 | Cep57l1 | 20:46610145-46666830 | 0.0017 | -2.81 | 0.0487 |
| ENSRNOG00000007412.4 | Dok1 | 4:113864214-113866674 | 0.0017 | -3.42 | 0.0489 |
| ENSRNOG00000012928.7 | Prmt9 | 19:34070631-34105610 | 0.0017 | -3.18 | 0.0489 |
| ENSRNOG00000028403.5 | Ptcd2 | 2:29570537-29598277 | 0.0017 | -3.02 | 0.0489 |
| ENSRNOG00000031081.4 | Stat2 | 7:2691368-2707741 | 0.0017 | -2.15 | 0.0494 |

* FDR: false discovery rate. The FDR-adjusted P value was sorted from lowest to highest.
